# A murine model of sepsis induces age- and sex-specific chromatin remodeling in myeloid-derived suppressor cells

**DOI:** 10.3389/fimmu.2026.1750174

**Published:** 2026-03-24

**Authors:** Angel M. Charles, Christine E. Rodhouse, Dijoia B. Darden, Marie-Pierre L. Gauthier, Mingqi Zhou, Miguel Hernandez-Rios, Dayuan Wang, Gemma Casadesus, Letitia Bible, Alicia M. Mohr, Feifei Xiao, Guoshuai Cai, Jason O. Brant, Shannon M. Wallet, Clayton E. Mathews, Lyle L. Moldawer, Paramita Chakrabarty, Rhonda L. Bacher, Philip A. Efron, Robert Maile, Michael P. Kladde

**Affiliations:** 1 Sepsis and Critical Illness Research Center, Department of Surgery, College of Medicine, University of Florida, Gainesville, FL, United States; 2 Department of Biochemistry and Molecular Biology, College of Medicine, University of Florida, Gainesville, FL, United States; 3 Department of Biostatistics, College of Public Health & Health Professions, University of Florida, Gainesville, FL, United States; 4 Department of Pharmacy and Therapeutics, University of Florida College of Medicine, Gainesville, FL, United States; 5 Department of Oral Biology, College of Dentistry, University of Florida, Gainesville, FL, United States; 6 Department of Pathology, Immunology and Laboratory Medicine, College of Medicine, University of Florida, Gainesville, FL, United States; 7 Department of Neuroscience, University of Florida College of Medicine, Gainesville, FL, United States

**Keywords:** aging, chromatin accessibility, DNA methylation, epigenetics, myeloid-derived suppressor cells, precision medicine, sepsis, sex

## Abstract

**Introduction:**

Sepsis survivors frequently develop long-term immune dysfunction, but the epigenetic mechanisms underlying persistent myeloid suppression remain unclear. Myeloid-derived suppressor cells (MDSCs), whose function is shaped by host age and sex, are key contributors to post-sepsis immune dysregulation.

**Methods:**

Here, we present a high-resolution epigenetic map targeting gene promoters of MDSCs after sepsis and daily chronic stress using MAPit-FENGC, a single-molecule assay that simultaneously profiles DNA methylation and chromatin accessibility. In a clinically relevant murine model, including young and older adult male and female mice, splenic MDSCs were isolated for MAPit-FENGC and single-cell RNA sequencing.

**Results:**

Unsupervised clustering identified nine promoter classes reflecting chromatin dynamics: age- and sex-dependent sepsis-induced opening (Classes 1-4), persistent closure with varying levels of DNA methylation (Classes 5-7), and constitutive openness post-sepsis (Classes 8, 9). Transcriptomic profiling corroborated these promoter states, linking accessibility with gene expression.

**Conclusions:**

These findings define promoter-level epigenetic classes across a targeted locus panel in splenic CD11b^+^Gr1^+^ cells within this murine sepsis model and generate mechanistic hypotheses regarding age- and sex-associated chromatin states.

## Introduction

Sepsis, when the host has organ insufficiency/failure in response to infection (1), affects in excess of 1.7 million adults in the United States, and results in the death of more than 350,000 of these individuals (2). In addition, sepsis is estimated to cost greater than $38 billion per year in the US alone (3). Although acute hospital mortality has improved due to measures such as the Surviving Sepsis campaign (4), long-term patient outcomes remain dismal (5, 6). Despite the significance and impact of all the former, few to no therapeutics are available for this patient population (7).

Myeloid-derived suppressor cells (MDSCs), delineated almost two decades ago (8), are defined as pathologically activated myeloid cells with potent immunosuppressive activity, yet they also possess some pro-inflammatory properties (9). Although these leukocytes have been investigated in the cancer field for some time, research focused on MDSCs in sepsis is relatively recent (10). MDSCs have been demonstrated to expand in number, remain circulating in the sepsis survivor, and are associated with poor outcomes in these patients (11, 12). Whereas these sepsis-derived MDSCs meet the key functional definition of MDSCs (immunosuppressive activity against lymphocytes, etc.), data indicate that MDSCs from septic mice are not identical to oncologic-derived MDSCs in both mice and humans (13–15). Modification of MDSCs in the sepsis survivor represents a potential approach to improve these patients’ outcomes (16), but still requires a better disease-specific understanding of these cells.

In addition to the need for more disease-specific precision/personalized therapeutics for sepsis, there is a need to delineate how sepsis engenders unique effects in certain cohorts, e.g., age and sex (16–25). Work from our group and others has illustrated that both sex and age have significant effects on the sepsis response, as well as on the post-infection MDSCs generated by that host (17, 18, 20–22). Thus, investigating these cohorts will be key to any successful future interventions for sepsis survivors.

Epigenetics is the study of changes in gene regulation/phenotype that occur without alteration of DNA sequence, mediated by DNA methylation, chromatin accessibility, and histone modifications. These mechanisms are considered a potential means of successfully modifying MDSCs to alleviate their pathology in the host (26). One method for studying the epigenetics of sepsis-induced MDSCs is Methyltransferase Accessibility Protocol for Individual Templates (27) combined with Flap-Enabled Next-Generation Capture (MAPit-FENGC), a validated method that allows for simultaneous single-molecular-level analysis of endogenous CpG methylation and chromatin accessibility at pre-determined target regions (28, 29). Using a preclinical surgical sepsis animal model that better parallels the human condition (17, 21), we utilized MAPit to assess the epigenetic uniqueness of isolated murine splenic Cd11b^+^Gr1^+^ MDSCs across several cohorts: sepsis versus control, male versus female, and young adult versus older adult.

## Materials and methods

### Animals

Young (3–5 mo) and old (18–22 mo) C57BL/6J (B6) mice were purchased from Jackson Laboratory (Bar Harbor, ME). Mice were cared for by the University of Florida Animal Care Services (Gainesville, FL) and housed in transparent cages (four animals of same sex/age per cage) within specific pathogen-free facilities at ambient room temperatures (23°C). The animals were provided standard rodent chow and water *ad libitum* for the duration of the experiment.

Young and older adult female (50%) and male (50%) mice underwent our intra-abdominal sepsis model of cecal ligation and puncture (CLP) followed by daily chronic stress (DCS). Eight mouse cohorts (n = 3 for each) were used: young female naïve, young female sepsis, young male naïve, young male sepsis, old female naïve, old female sepsis, old male naïve, and old male sepsis. Mice were euthanized seven days post CLP + DCS in compliance with predetermined IACUC Body Condition Score (BCS) criteria. A modification to this protocol included euthanasia if any one of the following occurred: 1) ≥ 20% weight loss from baseline, 2) ≥ 20% weight loss relative to age-matched controls if the animals were still growing during the study, 3) ≥ 20% weight loss from baseline in older mice that maintained a BCS > 2 during the first five days after CLP, or 4) > 40% weight loss from baseline in older mice with a BCS > 2 after the first five days following CLP. Based on these criteria, no mice had to be euthanized for excessive weight loss during the course of the study. Therefore, animals were euthanized within their home cages whenever possible to avoid co-mingling, or by placement in an appropriate enclosure. Carbon dioxide gas (100% medical grade) was introduced into the enclosure at a displacement rate of 30-70% of chamber volume per minute, monitored by a flow meter and maintained for at least 1 minute after respiration ceased.

### Intra-abdominal sepsis model

CLP was conducted under isoflurane anesthesia as we have previously described (30). The cecum was ligated 1 cm from its tip and a 25-gauge needle was used to puncture the cecum. Buprenorphine analgesia was provided for 48 hours post-surgery. Antibiotics (imipenem monohydrate; 25 mg/kg diluted in 1 mL 0.9% (w/v) NaCl) were administered 2 hours post-CLP, then continued for 72 hours. DCS was conducted as previously described (31). Briefly, this involved placing mice in weighted plexiglass animal restraint holders (Kent Scientific, Torrington, CT) for two hours daily starting the day after CLP. The purpose was to simulate the ICU environment, where patients are often bedbound with limited mobility. MDSCs were isolated using a CD11b^+^Gr1^+^ positive selection (EasySep™ Mouse MDSC (CD11b+Gr1+) Isolation Kit, StemCell, Cambridge, MA, USA) according to manufacturer’s instructions and as we have described previously (21). Our rationale for using CLP + DCS is that it is an established model designed to better recapitulate the prolonged physiologic and psychologic stressors of the ICU environment and to induce durable post−sepsis immune dysregulation (31). For clarity, we refer to this CLP + DCS model throughout as ‘sepsis’; however, all inferences are limited to the CLP + DCS condition and the loci assayed.

### Single-cell RNA sequencing

Splenocytes were obtained and counts were quantified using a Cellometer™ Auto 2000 Cell Viability Counter (Nexcelom Bioscience, Lawrence, MA). One million cells with a minimum of 85% viability were obtained for scRNA-seq using 10x Genomics chemistry (Chromium X instrument, Pleasonton, CA) as described in the companion paper (Rodhouse et al.).

### Methyltransferase accessibility protocol for individual templates combined with flap-enabled next-generation capture epigenetic analysis

MAPit-FENGC is a validated method that allows for simultaneous single-molecular level analysis of methylation and accessibility at target promoter and enhancer regions (28, 29). One million cells from each mouse were washed with ice-cold PBS, pH 7.2. Cells were centrifuged at 1,000 *x g* for 5 minutes then washed in ice-cold cell resuspension buffer (20 mM HEPES, pH 7.5, 70 mM NaCl, 0.25 mM ethylenediaminetetraacetic acid (EDTA) pH 8.0, 0.5 mM ethylene glycol tetraacetic acid (EGTA), pH 8.0, 0.5% (w/v) glycerol, with freshly supplemented 10 mM dithiothreitol and 0.25 mM phenylmethylsulfonyl fluoride). Cells were centrifuged at 1,000 *x g* for 5 minutes before being resuspended with 92 μL of resuspension buffer containing 0.5% (w/v) digitonin, followed by staining with trypan blue to ascertain 100% permeabilization. Cells were then treated with 100 U M.CviPI GpC methyltransferase (32) (100 U/million cells; New England Biolabs, M0227B-HI) with freshly added 160 µM *S*-adenosyl-*L*-methionine for 15 minutes at 37 °C. The reaction was terminated using an equal volume of 10 mM EDTA, 100 mM NaCl, and 1% (w/v) SDS followed by a quick vortex at medium speed. The nuclei were treated with 100 µg/ml RNase A for 30 min at 37 °C followed with 100 µg/ml proteinase K treatment at 50 °C overnight. Extraction of genomic DNA was conducted using phenol-chloroform-isoamyl alcohol (25:24:1, v/v) phase separation, which was followed by ethanol precipitation, then resuspension in molecular-grade H_2_O.

The full FENGC targeted enrichment methodology for concurrent profiling of chromatin accessibility and CpG methylation can be found at Zhou et al. (29). Briefly, a total of 98 target promoters from immune-related genes were located using the transcription start site (TSS) and ENCODE annotations from the mm10 genome assembly. Capture and enrichment of the 98 target sequences was conducted with a total of 294 oligonucleotides (flap oligos 1 and 2, as well as nested oligos 3). The gene promoters, sequences of the targeted regions, and oligos used for FENGC sequence enrichment are catalogued in **Supplementary Table 1**. All three oligos for each target (4 nmole each) were ordered as 100 μM salt-free solutions in 96-well format (Eurofins Genomics, KY, USA). After FENGC sequence enrichment, enzymatic deamination of cytosines to uracil, and PCR amplification, the amplicons were purified and submitted to the University of Florida Interdisciplinary Center for Biotechnology Research (UF-ICBR, RRID: SCR_019152) for SMRTbell library construction (Pacific Biosciences, Menlo Park, CA). Libraries were sequencing on a PacBio SEQUEL IIe instrument by UF-ICBR. The library pool was loaded at 120 pM, using diffusion loading and 20- to 30-hour movies with high-fidelity (HiFi) generation and demultiplexing. Sequencing Kit 2.0 (PacBio, 101-389-001) and Instrument Chemistry Bundle Version 11.0 were used with all other steps being performed using recommended protocol by the PacBio sequencing calculator.

For epigenetic analysis, HiFi circular consensus sequences (CCS) were generated using default parameters, except for a setting of ≥ 5 single polymerase read passes. CCS reads were aligned to the mm10 reference sequences using the Python reAminator pipeline (33). Cut-offs of 95% C-to-U conversion and 95% length of reference sequences alignments were applied. To unambiguously distinguish endogenous CpG methylation from M.CviPI-probed GpC methylation, GCG sites were removed from calculation of HCG and GCH methylation (where H = A, C, or T). Methylscaper (34) was used to generate tri-colored single-molecule heatmaps and averaged percent methylated HCG and GCH.

### Statistical analyses

To assess epigenetic differences across the assayed promoters in CD11b^+^Gr1^+^ MDSCs isolated from murine spleens, we used two types of data matrices (1): endogenous CpG methylation (HCG sites) and (2) GpC methylation representing chromatin accessibility (GCH sites). After filtering read quality for ≥ 95% conversion rate and ≥ 95% length of reference, each promoter target in each sample was further filtered for > 25 reads. For each sample, separate matrices of percent methylation across covered HCG and GCH sites were generated and filtered to retain loci with ≥ 25 informative reads. Principal component analysis (PCA) was conducted using base R’s prcomp() function without imputation. Targets were not used in the PCAs if < 2 samples per cohort had data passing the filters. PCA analysis of target reads exceeding all quality measures revealed a far outlier data point, corresponding to one young naïve mouse, reducing this group from n = 3 to n = 2. The data for this sample was removed for generation of the PCA plots shown in **Supplementary Figures 1**, **2** and all male methylscaper images in **Supplementary File 2**.

Single-molecule accessibility and methylation patterns were visualized and summarized for each sample and target using the Methylscaper v1.13.3 R package (34). Line plots of group-averaged chromatin accessibility and methylation across genomic positions were separated by sex, disease, and age groups using averages calculated by the methyl_average_status function with a 50-bp window size.

## Results

Exposure of mice to CLP + DCS induced widespread reorganization of chromatin accessibility and CpG methylation across a targeted panel of inflammatory, metabolic, and differentiation-associated promoters in splenic CD11b^+^Gr1^+^ cells, with additional age- and sex-dependent stratification of chromatin states. More specifically, multiple gene promoters showed increased accessibility paired with reduced methylation, a configuration associated with transcriptional activation. Other loci demonstrated persistent closure and hypermethylation, consistent with durable repression. This dichotomy defined nine distinct promoter classes, of which Classes 1–4 reflected sepsis-induced opening, whereas Classes 5–7 and Classes 8, 9 captured states with persistently closed (likely repressed or silenced) and constitutively open (likely expressed) chromatin, respectively. Importantly, these patterns (across the assayed promoters) were evident regardless of host age or sex, underscoring sepsis itself as the primary driver of epigenetic reprogramming.

### Epigenetic structure separates MDSCs by sepsis, age, and sex

Principal Component Analysis (PCA) of MAPit-FENGC single-molecule data across the assayed loci revealed clear distinctions (out to four PCs) in the epigenetic landscape of CD11b^+^Gr1^+^ splenic MDSCs based on sepsis exposure, age, and sex (**Supplementary Figures 1**, **2**). In PCA of endogenous CpG methylation (**Supplementary Figure 1**), septic mice formed distinct clusters compared to naïve controls. Notably, after sepsis, the greatest divergence in CpG methylation patterns was observed in older adult females relative to younger females and males of both ages, suggesting age- and sex-dependent remodeling of the methylome. When PCA was restricted to GpC methylation (chromatin accessibility) data (**Supplementary Figure 2**), sepsis again drove marked separation, particularly among female samples. Septic females (both young and older adult) clustered distinctly from their naïve counterparts, whereas MDSCs derived from all adult males displayed a more intermediate shift. These data suggest that accessibility at the profiled promoters is altered after CLP + DCS, with larger shifts observed in female samples in this cohort.

### Distinct promoter accessibility classes exist in MDSCs following sepsis

Unsupervised clustering analysis (34) of individual MAPit-FENGC promoter copies revealed nine promoter classes (Classes 1-9) in splenic CD11b^+^Gr1^+^ MDSCs, each with characteristic chromatin accessibility and DNA methylation patterns across conditions (**Table 1**). These classes captured a range of promoter architectures across this targeted dataset and demonstrated age- and sex-associated differences in endogenous CpG methylation, accessibility, or both at specific loci. Here, we describe qualitative trends for each class, highlighting how sepsis-induced chromatin accessibility (nucleosome-free regions, NFRs) and DNA methylation changes vary by promoter class, age, and sex.

**Table 1:** Gene promoters grouped by epigenetic class.

Epigenetic class	Representative gene promoters	Accessibility
1	*S100a9*	*Opened in all sepsis groups, but greatest in older females* *Partial DNA demethylation (of low levels of methylation)*
2	*Ccl3, Cxcr2, Lcn2, Lgals9, Nos2, Ptgs2, Rnase2a*	*Opened only in septic old females and septic young and old males*
3	*Mmp8, S100a8*	*Opened in all sepsis groups*
4	*Fyb*	*Opened more in septic females (young and older)* *Baseline levels of endogenous DNA methylation*
5	*Dab2, Emp1, F7, Lyz1, Mmp19, Pmp22, Retn, Serpina1a, Vnn1, Vsig4*	*Closed in all conditions but more closed in septic old female and septic males (young and old)* *Highest levels of endogenous DNA methylation*
6	*Ambp, Ccl5, Cd9, Cxcl3, Fabp7, Fgr, Gpnmb, Kdm6b, Lgals2, Mmp9, Serpine1*	*Lose accessibility in older sepsis* *Opened in young sepsis and naïve as well as older naïve* *Intermediate levels of endogenous DNA methylation*
7	*Car4, Ccl17, Gprc5b, Htr2a, Igkv12-44, Igkv4-69, Il10, Mt2, Plac8, S100a10, Stab1, Tmem176a, Vcan*	*Lose accessibility in older sepsis* *Opened in young sepsis and naïve as well as older naïve* *Low levels of endogenous DNA methylation*
8	*Atf6, Cd274, Hdac3, Il1rl2*, *Il4ra, Nfkbiz, Vdr*	*Open in all conditions, with age-dependent partial closure*
9	*Tet2*	*Opened in all conditions, sepsis and naïve*

**TABLE 1** Class behaviors reflect patterns within the assayed promoter panel and the CLP + DCS model.

### Sepsis induces NFRs at many promoters (Classes 1-4)

**Figure 1:**
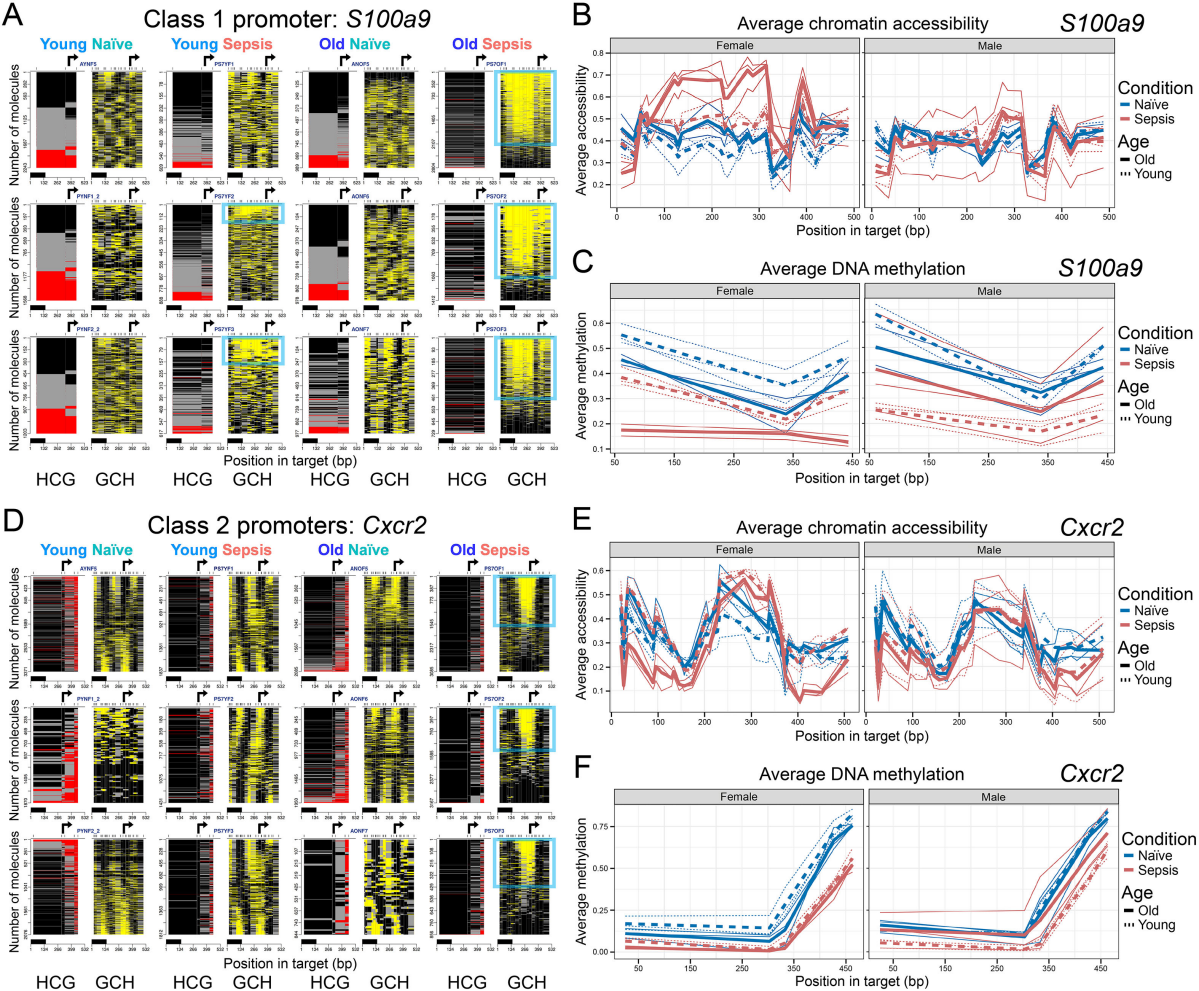
Class 1 and 2 promoters exhibit robust sepsis-induced chromatin accessibility, with age- and sex-specific differences and partial demethylation across MDSC subsets. **(A)** Representative single-molecule MAPit-FENGC plots for selected Class 1 promoters, showing simultaneous profiling of endogenous CpG methylation (HCG, left of each pair of panels, red) and *in vitro* GpC methylation (GCH, right of each pair, yellow) in Gr1^+^ MDSCs from four experimental groups: Young Naïve, Young Sepsis, Older Naïve, and Older Sepsis, all from female mice. Each horizontal line represents a unique DNA molecule, with the total number indicated on the y-axis. Black and gray marks denote methylated cytosines, while yellow marks indicate unmethylated (open or accessible) cytosines. Promoters are organized by gene and condition; Class 1 promoters were defined by consistent nucleosome-free region (NFR) formation (highlighted by blue boxes) in all septic groups, which co-localizes with the TSS (arrow). The black bar on top of the base pair scale is the size of a nucleosome particle (147 bp). Particularly robust NFRs are observed in *Older Sepsis* conditions, especially in females. **(B)** Average chromatin accessibility (GpC methylation) across the same *S100a9* region, plotted separately for females (left) and males (right). **(C)** Average endogenous CpG methylation levels across the *S100a9* promoter. In **(B)** and **(C)**, thin lines represent the average methylation at each GCH and HCG site in individual mice, respectively, whereas thicker lines represent the mean across the n = 2 or 3 samples in each cohort that passed filters. **(D–F)** Equivalent plots for the Class 2 promoter genes *Cxcr2* in Gr1^+^ MDSCs.

Several promoter classes exhibited marked chromatin opening due to nucleosome sliding/eviction in response to our sepsis model. A single Class 1 promoter, *S100a9* (encoding the calcium-binding protein A9, a key MDSC protein that regulates MDSC trafficking, expansion, and activation, as well as acting as an alarmin) (35, 36), showed striking, sepsis-driven increases in accessibility (at GCH sites; each mouse, right within each pair of panels; yellow color; **Figure 1A**). Large NFRs, with peak accessibility upstream of the transcription start site (TSS), formed on *S100a9* promoter copies in all six total mice in both the septic female (**Figure 1A**, **Supplementary File 1**; promoter copies with NFRs enclosed by blue rectangles) and male (**Supplementary File 2**) cohorts, except for one young septic mouse of both sexes (PS7YF1 and PS7YM3). These NFRs were characterized by promoter copies with continuous spans of accessible GCH of variable length—some extending across the entire 523 bp assayed region. This contrasts with the more random or diffuse accessibility over the *S100a9* promoter in naïve mice (and mice PS7YF1 and PS7YM3 that did not mount a sepsis response at this promoter) characteristic of linkers in randomly positioned nucleosome arrays.

The magnitude of *S100a9* NFR formation in Class 1 was modulated by sex and age. In MDSCs isolated from females, older septic mice had significantly more extensive *S100a9* promoter opening than young septic females (**Figure 1A**), whereas male MDSCs showed robust *S100a9* NFR formation in sepsis regardless of age (though slightly less pronounced than in females) (**Supplementary File 2**). In septic mice, *S100a9* demonstrated increased average chromatin accessibility in older adult females compared to young adult females and older adult males, with older females showing the largest NFRs and greatest fraction of molecules with NFRs (indicating a higher proportion of cells with an open promoter; **Figure 1B**). This Class 1 promoter had moderate levels of CpG methylation in naïve mice that underwent partial demethylation in septic mice, in line with an activated chromatin state (**Figure 1C**).

Class 2 promoters also gained accessibility in septic conditions but in a more context-dependent manner. Specifically, Class 2 chromatin opened (NFRs appeared) in older adult female sepsis and in male sepsis (both young and older), but not in young female sepsis. This pattern indicates that young females failed to open these promoters unless aged, whereas males of either age mounted an increase in accessibility. When present, the overall degree of NFR formation (length and fraction of promoter copies) for Class 2 genes was similar in females but more variable in males. A representative Class 2 gene is *Cxcr2* (**Figure 1D**, **Supplementary Files 1, 2**; encodes C-X-C chemokine receptor type 2, trafficking and facilitating the suppressive function of MDSCs within inflamed tissue) (37). In MDSCs from septic hosts, the *Cxcr2* promoter remained nucleosome-occupied (by randomly positioned nucleosomes) in young females but showed clear NFR formation at the TSS in older females (and in males of both ages). Concordantly, *Cxcr2* and other Class 2 promoters (e.g., *Nos2*, *Ptgs2*) exhibited low-to-moderate DNA methylation (at HCGs; left; red color) that either remained stable or slightly decreased with sepsis in the responsive groups (**Figure 1D**, **Supplementary Files 1, 2**). The requirement of advanced age for female MDSCs to demonstrate open Class 2 loci, *versus* no such requirement in males (**Supplementary File 2**), highlights a female-specific age dependency in epigenetic activation of this promoter set. Average chromatin accessibility at the *Cxcr2* locus was significantly increased in septic older adult mice, particularly females, consistent with an epigenetic state favoring enhanced expression (**Figure 1E**). This accessibility gain was not accompanied by substantial changes in DNA methylation (**Figure 1F**), which remained uniformly low upstream of the TSS across groups, suggesting a permissive chromatin environment at baseline, with further inducibility following septic challenge.

**Figure 2:**
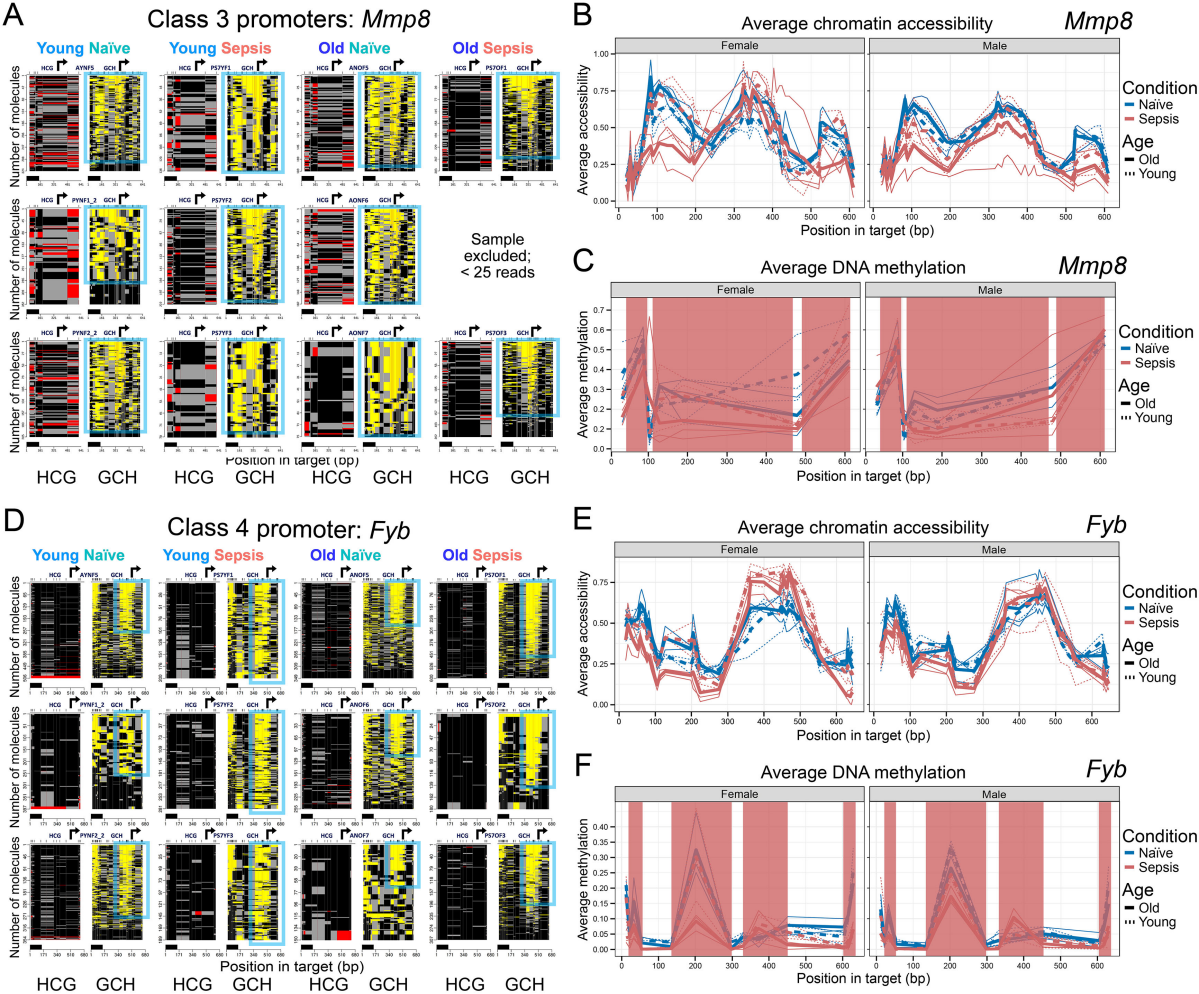
Class 3 promoters are consistently accessible in sepsis across all age and sex groups, and Class 4 promoters are activated by sepsis in females only. **(A)** Single-molecule MAPit-FENGC plots for Class 3 genes (e.g., *Mmp8*), showing HCG (left) and GCH (right) signals across Young/Older Female MDSCs. Sepsis induces NFRs in all septic conditions, regardless of age. The figure is annotated as in **Figure 1**. **(B)** Average chromatin accessibility (GpC methylation) across the same region, plotted separately for females (left) and males (right). **(C)** Average endogenous CpG methylation levels across the promoter. **(D-F)** Equivalent plots for the Class 4 promoter *Fyb.* Red shading in **(C)** and **(F)** marks segments of the curves that overlap CCG sites, where off-target methylation by the GpC chromatin-probing enzyme can elevate apparent methylation levels but likely reflects chromatin accessibility rather than genuine CpG methylation.

Class 3 promoters were broadly responsive to sepsis across all cohorts, showing no significant sex- or age-dependent differences in accessibility. These loci, e.g., *S100a8* (S100A8 acts similarly to S100A9) (35) and *Mmp8* (MMP8 facilitates MDSC mobilization and migration, as well as the immunosuppressive environment) (38), contained NFRs in some cells at baseline and formed NFRs in more cells and/or of increased length after septic shock. However, the NFRs formed to similar extents in MDSCs from septic hosts in males and females and in both young and older mice. Consequently, Class 3 represents a core set of promoters that are consistently opened by sepsis irrespective of host age or sex. Notably, this pattern was observed even in septic mice (PS7YF1 and PS7YM3) that did not exhibit chromatin remodeling at Class 1 and 2 promoters. DNA methylation of Class 3 promoters was generally low to intermediate and showed modest changes (often slight demethylation), if any, upon sepsis. The uniform NFR formation at these sites suggests they may drive fundamental sepsis-responsive genes in MDSCs that are required in all populations. For instance, *S100a8* (encoding S100 calcium-binding protein A8) promoter exhibited equivalent accessibility gains in septic males and females, young and older (**Supplementary Files 1, 2**), mirroring the behavior of its Class 1 counterpart *S100a9*, but without the older-female bias. Similarly, *Mmp8* (encoding matrix metalloproteinase-8) opened consistently in all septic groups, forming an NFR at the TSS (**Figure 2A**, **Supplementary Files 1, 2**). Concomitantly, compared to naïve mice, septic mice (older females in particular) showed increased inaccessibility upstream and downstream of the region of TSS accessibility at the *Mmp8* promoter (**Figure 2B**). Thus, Class 3 promoters highlight sepsis-induced epigenetic changes that are common to both sexes and ages, representing an invariant component of the MDSC response to sepsis. The sepsis-induced increase in chromatin accessibility at *Mmp8* promoter was not associated with true DNA demethylation (**Figure 2C**; apparent demethylation of two HCG sites between map positions 100–200 bp is due to off-target methylation by M.CviPI of the second cytosine in accessible CCG sites (regions highlighted red)). Open chromatin in naïve mice of both sexes and ages suggests enhanced transcriptional readiness.

In contrast, a single Class 4 promoter displayed a clear sex-specific accessibility pattern. This promoter, *Fyb*, drives expression of transcript encoding adhesion and degranulation adaptor protein (ADAP), a signaling adaptive protein hypothesized to support MDSC migration, T-cell suppressive activity, and survival (39). *Fyb* promoter demonstrated TSS-localized NFRs in older and young adult naïve mice (**Figure 2D**), which showed an increased number of NFR-containing MDSCs from septic female mice of both ages. As observed at *Mmp8*, regions upstream and downstream of the accessibility peak at the *Fyb* TSS in naïve mice became inaccessible after septic shock (**Figure 2D**). In contrast, septic males (older or young) showed little to no detectable increase in chromatin opening (and less concomitant protection) beyond the initial accessibility of the *Fyb* TSS region in naïve male mice of both ages. The average chromatin accessibility plot, particularly in female mice, reveals increased and decreased accessibility in MDSCs from septic mice compared to naïve controls over the TSS region and upstream regulatory elements of *Fyb*, respectively (**Figure 2E**). Considering average CpG methylation at ACG and TCG sites (i.e., excluding off-target methylation by M.CviPI of accessible CCG (shaded red)), *Fyb* promoter showed low background levels of methylation at most CpG sites in all cohorts, consistent with an epigenetic landscape that supports transcriptional activation in females (**Figure 2F**). The absence of any chromatin changes in males, despite an adequate sepsis insult (as verified by other classes), emphasizes a sex divergence in epigenetic regulation, with preferential activation of the Class 4 locus in MDSCs from female mice during sepsis.

### Persistently closed promoters remain refractory to induction in sepsis (Classes 5-7)

In contrast to the sepsis-inducible classes above, chromatin at Class 5–7 loci was closed with no formation of an NFR in any group (naïve or septic, male or female, young or older). Instead, accessibility was limited to relatively short linkers distributed across each promoter, indicating that their DNA was packaged into arrays of randomly positioned nucleosomes. Notably, linker accessibility in Class 5–7 promoters decreased in MDSCs isolated from septic mice (older females and males) and young septic male mice. Therefore, Classes 5–7 represent promoters that underwent sepsis-specific loss of linker accessibility in an age- and sex-driven manner, contrasting with the NFR formation observed in Class 1–4 promoters.

The distinction between Classes 5, 6, and 7 lies in their DNA methylation status: Class 5 promoters were generally characterized by high levels of endogenous DNA methylation at all CpGs (HCGs) (hypermethylation), which is consistent with a silenced state; Class 6 promoters exhibited moderate-to-high CpG methylation (typically showing little to no demethylation); and Class 7 promoters had low baseline methylation. Despite these differences, all three classes suggest a sepsis-specific loss of accessibility in older mice (male and female), and to a lesser extent, in young adult male mice.

**Figure 3:**
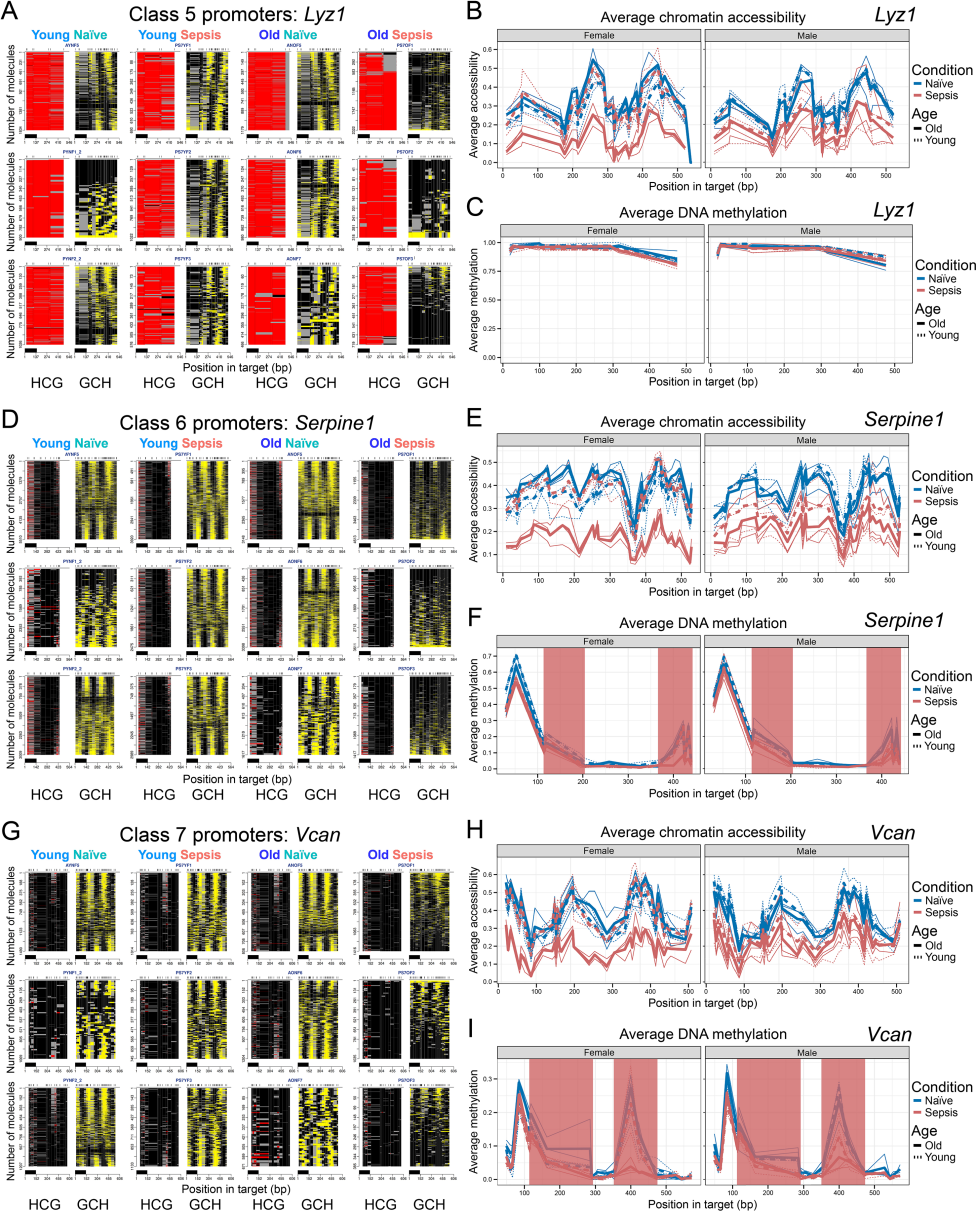
Class 5–7 promoters possess varying levels of DNA methylation but are all packaged into arrays of randomly positioned nucleosomes, where linkers lose accessibility in Older Sepsis only. **(A)** MAPit-FENGC plots of *Lyz1* showing no detectable NFRs in any condition across MDSCs from young/older female mice. HCG and GCH signals indicate high methylation and random nucleosome positioning. The figure is annotated as in **Figures 1**, **2**. **(B)** Average chromatin accessibility (GpC methylation) across the same region, plotted separately for females (left) and males (right). **(C)** Average endogenous CpG methylation levels across the promoter. **(D-F)** Equivalent plots for the Class 6 *Serpine1* promoter. **(G-I)** Equivalent plots for the Class 7 *Vcan* promoter. Red shading in **(F)** and **(I)** marks segments of the curves that overlap CCG sites, where off-target methylation by the GpC chromatin-probing enzyme can elevate apparent methylation levels but likely reflects chromatin accessibility rather than genuine CpG methylation.

Representative Class 5 genes include *Lyz1* (lysozyme 1), *Retn* (resistin), and *F7* (coagulation factor VII), among others (**Figures 3A–C**, **Supplementary Files 1, 2**). After sepsis, these promoters exhibited minimal changes in DNA methylation but showed decreased linker accessibility, specifically, in older adult mice (both sexes) and in young males. For example, *Lyz1* promoter remained highly methylated, with no visible formation of large NFRs capable of supporting active transcription in MDSCs from either naïve or septic mice. Similarly, *Retn* showed no NFR formation in any condition, although its methylation level declined slightly in older sepsis compared to older naïve animals (**Supplementary Files 1, 2**).

Exemplifying Class 6, the promoter of *Serpine1* (**Figures 3D–F**, **Supplementary Files 1, 2**), encoding the fibrinolytic serine protease inhibitor Pai-1 (a protein that influences MDSCs abundance and activity, often promoting immunosuppression and tumor growth) (40), was occupied by arrays of randomly positioned nucleosomes in naïve control mice (**Figure 3D**). Similar to Class 5 promoters, much of this characteristic linker accessibility was diminished in MDSCs from older septic mice. Average chromatin accessibility plots show that *Serpine1* is less accessible in septic groups, particularly in older mice, and to a lesser extent, young males, compared to their naïve counterparts (**Figure 3E**). No true DNA demethylation was detected (**Figure 3F**; the apparent demethylation of five CpG sites near map position 423 bp in MDSCs from some septic mice is due to off-target methylation by M.CviPI at accessible CCG sites (region highlighted red)). Despite baseline CpG methylation at the *Serpine1* TSS (map position 205–367 bp), the chromatin state of the *Serpine1* promoter is consistent with strong transcriptional silencing due to occupancy by randomly positioned nucleosomes and absence of sufficiently large NFRs capable of supporting active transcription. Given the role of *Serpine1* in inflammation, tissue remodeling, and coagulopathy, this decreased accessibility may reflect impaired inflammatory reprogramming of MDSCs during sepsis.

A Class 7 example is the *Vcan* gene (**Figures 3G–I**, **Supplementary Files 1, 2**) that encodes Versican, an extracellular matrix component that is upregulated during sepsis and thought so have some immune regulatory properties (41, 42). Similar to Class 5 and 6 genes, the *Vcan* promoter in MDSCs from young or naïve female mice had no large NFRs, only displaying linker accessibility in arrays of randomly positioned nucleosomes (**Figure 3G**). However, as was observed for Class 5 and 6 loci, in MDSCs from older septic females, this characteristic linker accessibility diminished. Average chromatin accessibility across the *Vcan* promoter revealed a modest but consistent decrease in septic conditions, particularly in older, but not young, adult female mice (**Figure 3H**). Male adult septic mice (young and older) showed reduced average accessibility across the *Vcan* promoter, although the effect was less pronounced than that observed in older adult septic females. Conversely, young female and naïve adult male mice showed relatively higher accessibility, suggesting age- and sex-specific regulation. Again, no true DNA demethylation was observed at this locus; instead, only decreased accessibility at CCG sites was detected (**Figure 3I**).

### Constitutively accessible promoters (Classes 8-9)

Within this class we have two sub-classes; promoters with constitutive NFRs that largely remain refractory to sepsis (Class 8) and a constitutively accessible promoter that maintains open chromatin in all conditions (Class 9). Class 8 gene promoters showed high accessibility, with prominent NFRs encompassing the TSSs in all four conditions. However, these promoters illustrated an age-dependent, relatively modest loss of NFR accessibility in MDSCs from only older septic female mice, where overall NFR architecture and presumably transcriptional activity was largely preserved. Class 8 promoters generally exhibited low, baseline DNA methylation levels, reflective of their active status.

**Figure 4:**
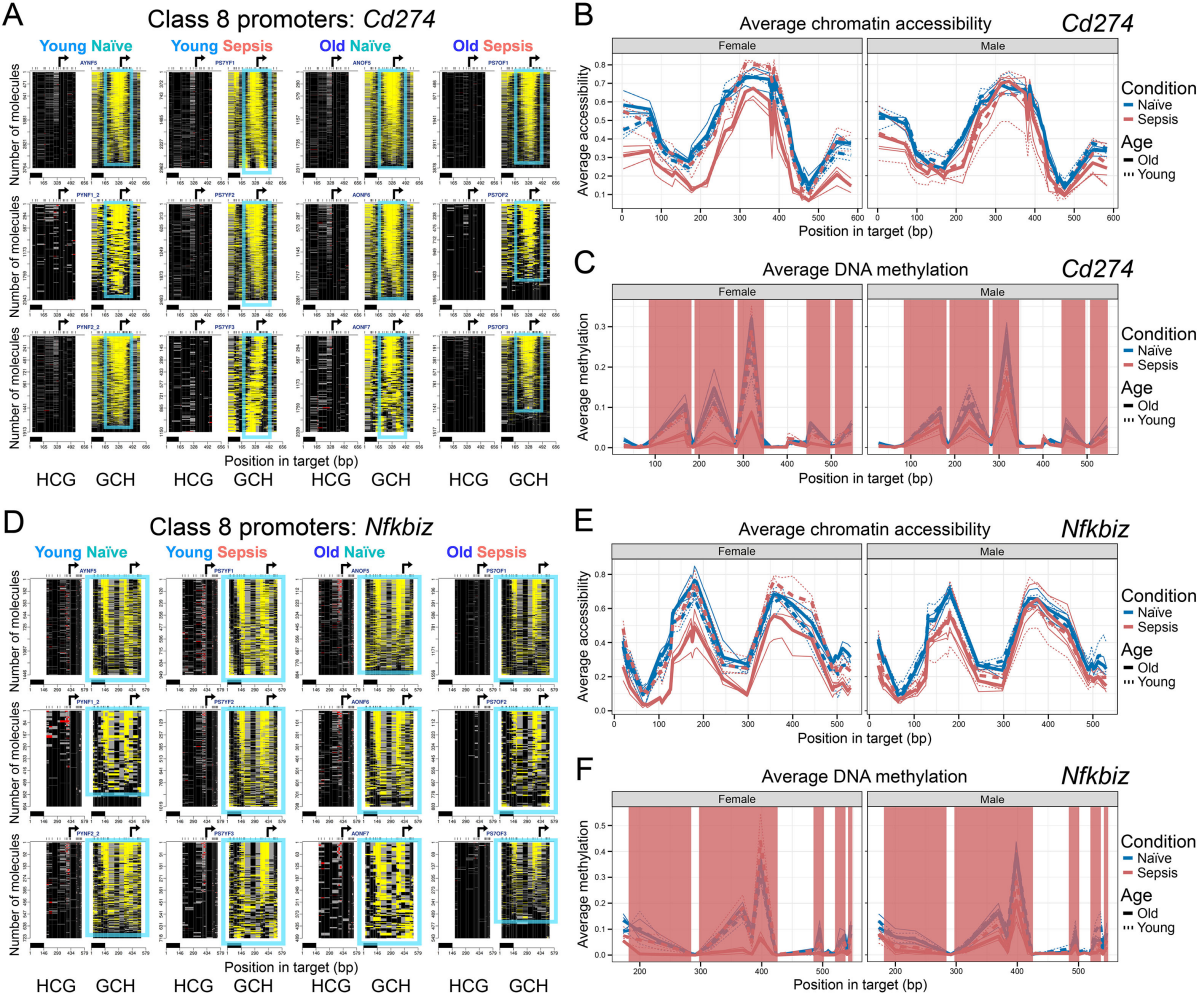
Class 8 promoters constitutively harbor well-defined NFRs across all conditions, which partially lose accessibility specifically in Female Older Sepsis. **(A)** HCG and GCH plots for *Cd274* promoter show prominent NFRs across all conditions. The figure is annotated as in **Figures 1**, **2**. **(B)** Average chromatin accessibility (GpC methylation) across the same region, plotted separately for females (left) and males (right). **(C)** Average endogenous CpG methylation levels across the promoter. Red shading in **(C)** and **(F)** marks segments of the curves that overlap CCG sites, where off-target methylation by the GpC chromatin-probing enzyme can elevate apparent methylation levels but likely reflects chromatin accessibility rather than genuine CpG methylation. **(D-F)** Equivalent plots for *Nfkbiz* promoter.

One Class 8 gene is *Cd274* (**Figures 4A–C**, **Supplementary Files 1, 2**), encoding the immune-checkpoint inhibitor PD-L1, which in MDSCs plays a direct role in suppressing T-cell responses (43). The *Cd274* promoter exhibited the highest levels of NFR accessibility in MDSCs from young septic mice and in naïve adult controls. MDSCs from older adult septic females showed a modest reduction in NFR accessibility but retained NFR structure, consistent with ongoing transcriptional activity. Average accessibility at the *Cd274* locus was modestly decreased in MDSCs from older adult septic female mice compared to naïve controls and young septic mice, with septic animals, particularly older individuals, showing the greatest reduction (**Figure 4B**). Excluding DNA methylation of accessible CCG sites, only basal CpG methylation levels were detected within *Cd274* regulatory sequences, consistent with permissive chromatin and potential gene expression (**Figure 4C**).

Other Class 8 genes are represented by *Nfkbiz* (encoding the NF-κB inhibitor IκBζ), *Vdr* (vitamin D receptor), *Il4ra* (IL-4 receptor α chain, which has been shown to be important to MDSC-suppressive activity) (44), and *Atf6* (ATF6, a transcription factor that activates target genes in the unfolded protein response) (**Figures 4D–F**, **Supplementary Files 2, 3**). These gene promoters all followed a similar pattern with NFRs present in all conditions, except in MDSCs from older septic female mice. In each case, MDSCs from the older female septic group showed baseline CpG methylation (omitting CCG) and a modest loss of promoter accessibility that is unlikely to impair transcriptional activity.

**Figure 5:**
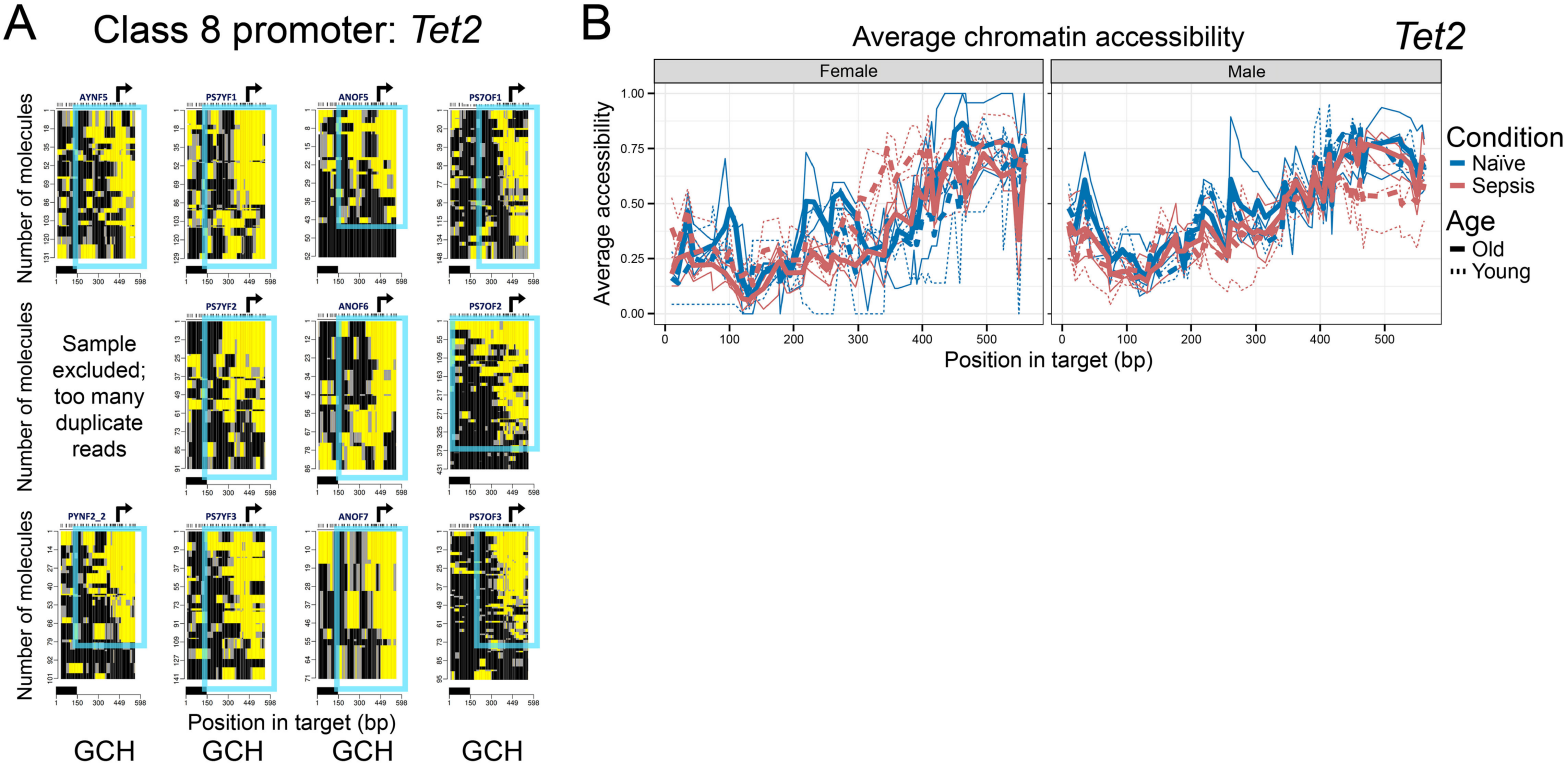
Class 9 promoter is constitutively open in sepsis. **(A)** GCH plots for the *Tet2* promoter show strong, stable NFRs in all female MDSCs. Otherwise, the figure is annotated as in **Figures 1**, **2**. **(B)** Average chromatin accessibility (GpC methylation) across the same region, plotted separately for females (left) and males (right). No plots of average endogenous CpG methylation are shown because all HCG sites are CCG.

Finally, a single promoter, *Tet2* (Class 9), encoding the DNA demethylating enzyme Tet2, remained highly accessible across all conditions, largely unaffected by sepsis, age, or sex (**Figure 5**). This constitutive chromatin accessibility, even after the stress of sepsis, is consistent with active transcription. CpG methylation could not be assessed because all 35 HGCs in the *Tet2* promoter amplicon are CCGs.

### Single-cell transcriptomics support epigenetic promoter class distinctions in MDSC subsets

**Figure 6:**
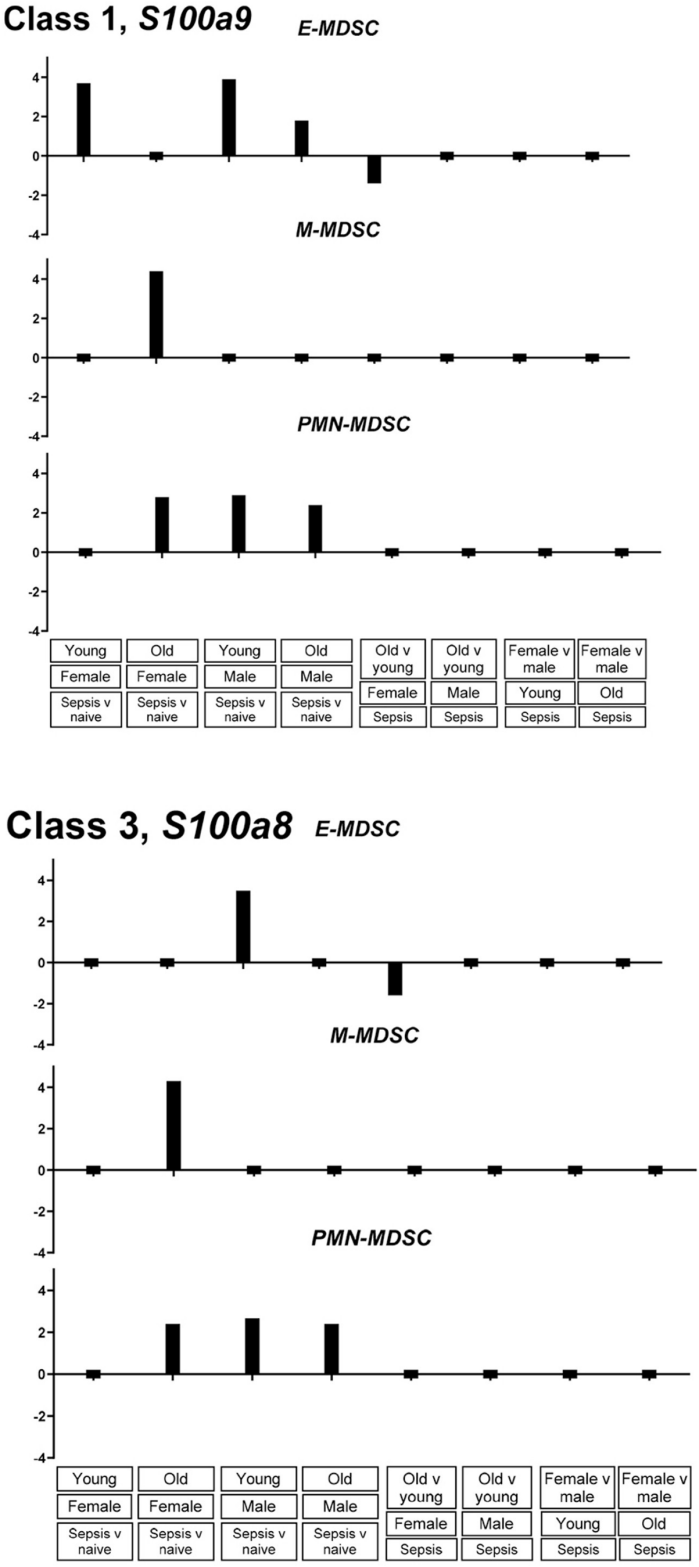
Class 1 and Class 3 genes show consistent sepsis-induced transcriptional activation across MDSC subsets. Bar plots log_2_ fold change in expression for *S100a9* (Class 1) and *S100a8* (Class 3) across E-MDSC, M-MDSC, and PMN-MDSC populations (conditions and comparisons as labeled) derived from single-cell RNA-sequencing analysis of splenic Cd11b^+^ Gr1^+^ cells.

**Figure 7:**
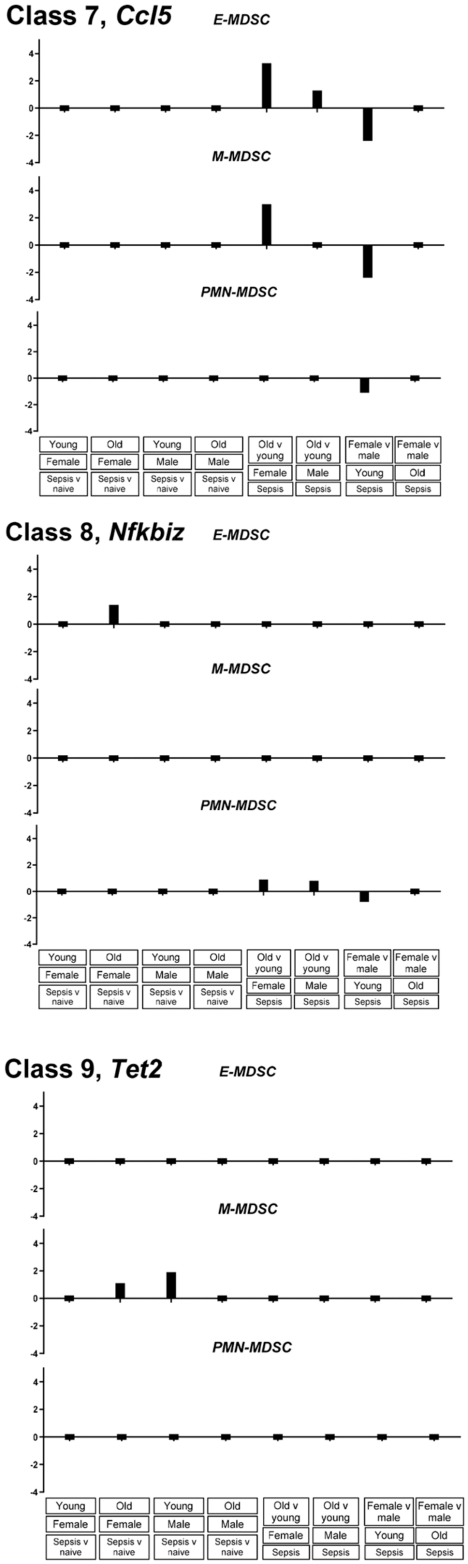
Expression of Class 7, Class 8, and Class 9 genes across MDSC subtypes reveals repression and age-dependent silencing. Bar plots depict log_2_ fold change in gene expression (conditions and comparisons as labeled) for *Ccl5* (Class 7), *Nfkbiz* (Class 8) and *Tet2* (Class 9), derived from single-cell RNA-seq analysis of splenic Cd11b^+^Gr1^+^ cells. Data are shown separately for early MDSCs (E-MDSC), monocytic MDSCs (M-MDSC), and polymorphonuclear MDSCs (PMN-MDSC).

To determine whether promoter accessibility classes defined by MAPit-FENGC corresponded to functional gene expression patterns, we analyzed single-cell RNA-seq data from CD11b^+^Gr1^+^ splenic leukocytes isolated from young and older, male and female adult mice under naïve or septic conditions, collected in the Companion Paper (Rodhouse et al.). Data were stratified into three MDSC subtypes based on gene expression patterns: early MDSCs (E-MDSC), monocytic MDSCs (M-MDSC), and granulocytic MDSCs (PMN-MDSC). The gene *S100a9*, assigned as a Class 1 promoter and characterized by widespread NFR formation in all septic groups, was induced across all three MDSC subsets in response to sepsis, regardless of age or sex (**Figure 6**). This transcriptional profile matches the broadly accessible chromatin configuration observed epigenetically. Similarly, the Class 3 promoter *S100a8*, which formed sepsis-induced NFRs in young and older mice, displayed upregulation in E-MDSCs and PMN-MDSCs during sepsis. In contrast, a Class 6 promoter *Ccl5*, which remained epigenetically inaccessible in all conditions, showed only modest and selective transcriptional induction in a subset of MDSCs, primarily E- and M-MDSCs, in young male septic mice (**Figure 7**). These observations are consistent with a transcriptionally repressed or enhancer-driven regulatory program.

In contrast, *Nfkbiz*, assigned to Class 8 based on selective modest promoter closure in “Aged Sepsis,” showed relatively low, but detectable, expression across MDSC subsets. While MAPit-FENGC revealed loss of promoter accessibility specifically in aged sepsis mice, scRNA-seq analysis did not show consistent transcriptional silencing in this group. Instead, modest expression changes were observed in PMN-MDSCs and E-MDSCs, particularly in young female septic mice. *Tet2*, the representative Class 9 gene whose promoter remained consistently accessible across all MAPit-FENGC conditions, exhibited modest transcriptional activation in scRNA-seq, primarily during sepsis in M-MDSCs. Despite the stable chromatin accessibility across all groups, expression was low or unchanged in E-MDSCs and PMN-MDSCs.

## Discussion

**Figure 8:**
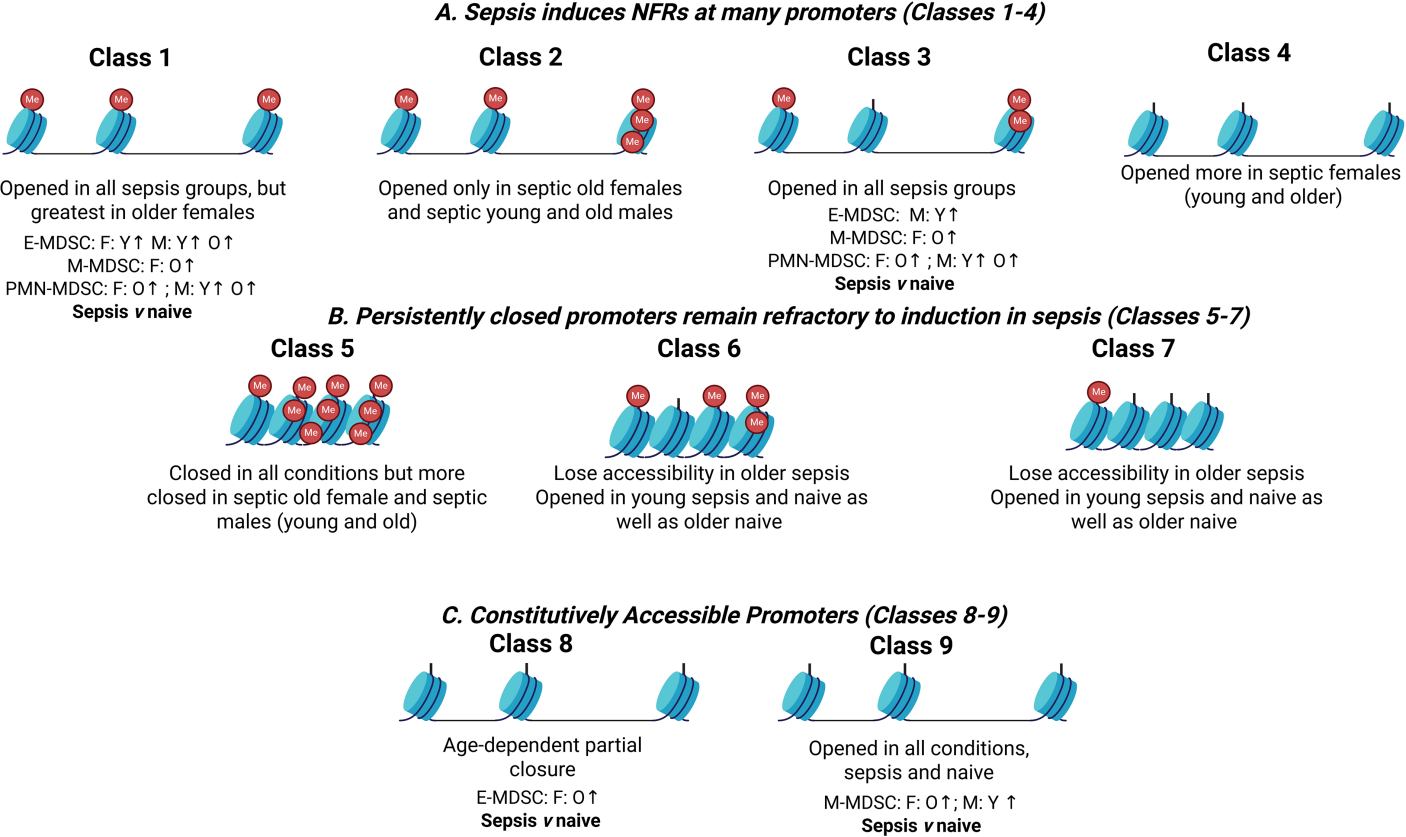
Hierarchy of responses of targeted promoters to naïve vs. CLP + DCS in young vs. older female and male mice.

Our study provides a high-resolution, promoter-targeted epigenetic map in a murine CLP + DCS model and suggests that host age and sex are associated with differences in chromatin accessibility and DNA methylation at specific loci in splenic CD11b^+^Gr1^+^ cells. Using MAPit-FENGC, we identified nine distinct promoter classes across multiple immune-relevant loci (**Figure 8**), encompassing a spectrum of response classes ranging from sepsis-induced chromatin opening to age-specific silencing (**Table 1**, **Supplementary Files 1, 2**). These epigenetic patterns were tightly linked to host sex, age, and sepsis exposure and corresponded to changes in transcription, as assessed by scRNA-seq.

We observed an age-dependent epigenetic repression of key immunoregulatory genes (e.g., *Cd274*, *Nfkbiz*, *Il4ra*) in older septic mice (Class 8). These promoters exhibited basal CpG methylation and robust promoter NFR accessibility in young or naïve mice. Older septic females showed a modest loss of accessibility but retention of NFR structure, consistent with ongoing transcriptional activity. Therefore, the observed accessibility patterns are most consistent with nucleosome repositioning, thereby emerging as a major contributor to reduced promoter accessibility in aged hosts. The selective, partial closure of immune checkpoint and anti-inflammatory loci (e.g., *Cd274*, *Il10*) is functionally relevant given established roles for PD-L1 and IL-10 pathways in T-cell suppression and myeloid-mediated regulation; however, in the present dataset these implications remain inferred from promoter architecture and transcript trends. We therefore interpret these Class 8 changes as a chromatin signature consistent with altered immunoregulatory potential in older hosts that requires direct functional testing. In aged septic hosts, epigenetic repression of these genes may render MDSCs less effective or dysregulated, contributing to the persistent immune dysfunction and chronic critical illness seen in this population (16, 21).

In contrast, sepsis-induced chromatin opening was observed in promoter classes associated with canonical MDSC effector genes (Classes 1-4). Promoters such as *S100a9*, *S100a8*, *Nos2*, and *Ptgs2* gained accessibility in septic MDSCs, often with concurrent demethylation, consistent with transcriptional activation (35, 45). The magnitude of these chromatin changes varied by sex and age. For example, Class 1 (*S100a9*) was accessible across all septic groups but showed particularly robust NFR formation in older females. These findings suggest that both age and sex can influence *S100a9* expression and chromatin accessibility, potentially contributing to the observed robust NFR formation at the *S100a9* promoter in older females during sepsis. Research has demonstrated that chromatin accessibility at the *S100a9* promoter is regulated during monocyte differentiation and is influenced by transcription factors such as C/EBPδ. In particular, C/EBPδ has been shown to bind within the *S100a9* promoter region, affecting its accessibility and expression levels (46). Age-related changes in immune function (“inflammaging”) have been associated with alterations in inflammatory markers, including S100a9. For example, a study investigating the impact of age and sex on inflammatory markers found that aging is associated with higher expression of *S100a9* and components of the NLRP3 inflammasome independent of sex (47). In the context of systemic lupus erythematosus (SLE), S100a9 has been identified as a molecule integral in determining sex-specific immune responses (36), which may be relevant to understanding its regulation in other inflammatory conditions like sepsis. The *S100a8* and *S100a9* promoters (Class 1 and 3 in MAPit-FENGC) exhibit universal sepsis-induced opening, particularly in aged females, and these genes are also among the top differentially expressed genes in our scRNA-seq dataset (in the accompanying manuscript, Rodhouse et al.). This gene pair is pivotal in MDSC activation, trafficking, and suppressive function. The robust accessibility and expression of *S100a8*/*a9* in both studies supports their role as central, sepsis-responsive effectors and potential biomarkers of MDSC reprogramming.

Class 2 promoters opened only in older females and males, but not young females highlighting a female-specific age threshold for epigenetic activation at specific loci. For example, CXCR2 is a chemokine receptor primarily expressed on neutrophils and plays a crucial role in mediating their recruitment to sites of infection, essential for survival in sepsis. Excessive or dysregulated CXCR2 signaling can contribute to tissue damage and organ failure (48), and the observed increased accessibility of the *CXCR2* gene in older females and males may suggest an age-related epigenetic regulation leading to enhanced expression.

The identification of stably closed promoters (Class 5) and constitutively open loci (Class 9) may further describe the epigenetic plasticity in MDSCs. Class 5 genes (e.g., *Lyz1*, *F7*) remained silenced through hypermethylation and packaging into arrays of randomly positioned nucleosomes regardless of condition. The refractoriness of Class 5 promoters to opening indicates they are epigenetically “locked down” in splenic MDSCs. These genes remain transcriptionally inactive even in sepsis, suggesting they are either irrelevant to the MDSC sepsis response or otherwise tightly repressed in this cell type.

Classes 6 and 7 highlight additional promoters shut down in MDSCs. Although these promoters had less DNA methylation than Class 5 promoters, they nevertheless remained epigenetically repressed as gauged by inability to form large NFRs following CLP sepsis + DCS challenge. Like Class 5, the chromatin of Class 6 and 7 promoters consists of randomly positioned nucleosomes with accessible linker DNA. Across Class 6 and 7 promoters, we frequently observed that all MDSC samples from older septic mice lacked NFRs, whereas one or more of the other conditions retained a relatively accessible configuration, suggesting age-specific chromatin condensation at these promoters. Across Class 6 and 7 promoters, all MDSC samples from older septic mice consistently lacked NFRs characteristic of active promoters, whereas one or more of the other conditions retained localized linker accessibility. This pattern indicates age-specific chromatin condensation within promoter regions that are normally permissive, rather than a loss of linker accessibility across the assayed promoters. As with all Class 5–7 promoters, these loci remain devoid of canonical NFRs, distinguishing them from the open promoter architectures observed in Classes 1-4.

Class 8 includes several immune-related genes (e.g., *Cd274*, *Il4ra*, *Nfkbiz*) whose partially diminished accessibility in older survivors of sepsis might contribute to impaired or altered immune regulation in this cohort. Notably, several Class 5–8 promoters, including *Cd274* (encoding PD-L1), *Il10*, and *Nfkbiz*, exhibited significant chromatin closure selectively in MDSCs from older septic mice. This pattern suggests a dominant role for nucleosome occupancy in regulating gene expression in aged hosts. Supporting this, our companion transcriptomic study (Rodhouse et al.) suggests that these genes were only modestly or minimally induced at the RNA level in older MDSC populations following sepsis, particularly in M-MDSCs. These findings support the concept that age-associated epigenetic silencing of immunoregulatory loci may cause persistent immune dysfunction in older sepsis survivors, and that transcriptional non-responsiveness may be epigenetically preconditioned in this cohort.

Class 9 promoter, *Tet2*, was persistently accessible across all groups, suggesting that it is a “core” MDSC housekeeping gene that remains transcriptionally poised or active even in the face of sepsis-induced stress. This suggests the *TET2* gene product, a DNA demethylating enzyme, is critical for core MDSC development and/or cell functions. TET2 is highly expressed in hematopoietic stem and progenitor cells and has been shown to be essential in regulating MDSC numbers in tumor models (49) and models of emergency myelopoiesis post-LPS-challenge (50). The constitutive accessibility underscores that some key promoters remain epigenetically unaltered and continuously active throughout sepsis, contrasting the dynamic changes seen in Classes 1-8. The absence of any observed loss of accessibility at *Tet2*, reinforces that such promoters are resilient to both inflammatory stress and host factors.

These context-specific activation patterns suggest that MDSC-mediated immunosuppression may arise *via* different regulatory programs in males versus females, and in young versus older hosts. This observation aligns with our prior data on age- and sex-specific differences in MDSC transcriptomes and function (17, 19), and may provide mechanistic insight into how such differences are epigenetically encoded.

Importantly, single-cell transcriptomics supported many of the epigenetic patterns described. Genes in Classes 1 and 3 showed increased expression in MDSC subsets concordant with sepsis-induced chromatin opening (**Figure 8**). In contrast, Class 8 genes (e.g., *Nfkbiz*, *Il4ra*) showed partial decoupling between accessibility and expression in older septic MDSC raising the possibility of additional regulatory checkpoints, such as enhancer silencing or post-transcriptional control, particularly in aged immune cells (13, 26). These findings underscore the importance of integrating multiple levels of gene regulation (chromatin state, methylation, and transcription) when evaluating MDSC behavior in critical illness. Collectively, these scRNA-seq results support the biological relevance of the MAPit-defined promoter classes, illustrating how chromatin accessibility states correspond to transcriptional outcomes across sepsis, sex, and age in MDSC subsets.

The epigenetic heterogeneity observed here helps explain the divergent immune phenotypes/endotypes that we (51, 52) have defined in sepsis survivors, and provides a foundation for understanding MDSC dysfunction in aging. In particular, the age-specific silencing of checkpoint and anti-inflammatory loci may promote persistent immune suppression, a hallmark of chronic critical illness and poor sepsis recovery (5, 16). Conversely, sex-biased promoter accessibility could underlie known disparities in sepsis outcomes between males and females (18). These findings may inform future strategies to test epigenetic reprogramming of MDSCs, but therapeutic implications remain hypothetical pending functional and genome-wide validation. Such therapies could include epigenetic modulators such as histone deacetylase (HDAC) inhibitors, bromodomain inhibitors, or DNA methyltransferase inhibitors, many of which are already being explored in oncologic and inflammatory contexts (7, 26).

Moreover, promoter classes defined here could serve as biomarkers of MDSC-state or targets for selective modulation. For instance, genes in Classes 1–3 may help stratify MDSC activation states post-sepsis and prioritize candidates for functional validation, while Class 8 promoters may highlight loci with age-associated changes in immunoregulatory chromatin. The stability of a Class 9 locus suggests that it may serve as an internal control or indicator of MDSC identity in future epigenetic studies.

Our findings provide mechanistic insight into how sepsis may drive divergent epigenetic fates in MDSCs that reflect hallmarks of both trained immunity and tolerance. The persistent chromatin accessibility observed at Class 1–3 promoters (e.g., *S100a9*, *Nos2*, *S100a8*), particularly in older or female hosts, suggests a form of innate immune training, where prior inflammatory exposure leaves a molecular imprint that primes these loci for rapid reactivation. This is consistent with studies showing that chromatin remodeling at promoters of inflammatory genes underlies the trained phenotype in monocytes and macrophages [reviewed in (53)]. Conversely, the selective, age-dependent silencing of promoters in Classes 6-8, including key regulatory genes such as *Cd274* and *Il10*, supports the emergence of an epigenetic tolerance program, particularly in aged MDSCs. These loci become refractory to activation despite hypomethylation, implicating nucleosome repositioning and chromatin compaction as dominant repressive mechanisms. The coexistence of both accessibility gains and repressive remodeling across promoter classes indicates that sepsis induces a divergent epigenetic landscape in MDSCs, poising some genes for increased responsiveness while silencing others. These dual trajectories may underlie the paradoxical state of immune hyperinflammation coupled with functional suppression seen in sepsis survivors and reflect an imprint of the inflammatory milieu on the myeloid epigenome that varies by age and sex.

### Limitations

We acknowledge that the present epigenetic dataset was generated in the context of the combined CLP + daily chronic stress (DCS) model and therefore cannot be used to attribute the observed promoter remodeling exclusively to CLP-induced sepsis. While this model was selected to better approximate the persistent physiologic stressors accompanying severe infection and critical illness, we cannot exclude the possibility that DCS independently contributes to, or modifies, the chromatin accessibility and methylation patterns observed in CD11b^+^Gr1^+^ cells. Accordingly, we have framed throughout the identified promoter classes as epigenetic signatures of the CLP + DCS post-sepsis state, and we note that inclusion of CLP-only and/or DCS-only cohorts profiled with the same MAPit-FENGC workflow will be required to resolve sepsis-specific effects and potential interaction terms. We also acknowledge antibiotic exposure as a potential confounder for sepsis *versus* naïve comparisons.

This study focused on a pre-selected set of immune and MDSC-relevant promoters, limiting discovery of unanticipated regulatory regions or enhancer-driven transcription. Additionally, we profiled Gr1^+^CD11b^+^ splenocytes, which include multiple MDSC subtypes and potentially other myeloid cells. Therefore, observed differences across age/sex/condition could reflect intrinsic chromatin remodeling within subsets, shifts in subset abundance, or both. Although scRNA-seq helped disaggregate subtype-specific effects, future studies using single-cell epigenomics (e.g., scATAC-seq or scCUT&Tag) could more finely resolve MDSC heterogeneity. Finally, our MAPit-FENGC dataset provides high-resolution data at selected promoters; the approach can be readily extended to capture distal regulatory elements, which may play significant roles in aging-related immune dysfunction.

The promoter “classes” were derived by unsupervised clustering of single−molecule promoter profiles, and in this type of analysis it is expected that some promoters can show distinct architecture (e.g., uniquely strong NFR formation, uniquely sex−restricted opening, or distinctive methylation/accessibility coupling) that does not align with broader clusters. In our dataset, these classes represent unique and reproducible promoter archetypes, and we retained them because merging them into adjacent clusters would obscure the defining chromatin behavior that motivated the classification. We report the clusters in this foundational study to allow characterization of sepsis responses when we perform the necessary future genome-wide epigenetic studies.

It is important to emphasize that the present study provides epigenetic and transcriptional evidence and does not directly test MDSC effector function. Prior murine work from our group has shown that polymicrobial sepsis can drive sustained expansion of Gr1^+^CD11b^+^ immature myeloid populations with T-cell suppressive activity and associated immune polarization (54, 55). However, the promoter-state signatures defined here should therefore be interpreted as hypothesis-generating, identifying locus-level chromatin configurations that are consistent with particular functional programs, which now motivate direct validation. In future work, we will pair promoter-class stratification with functional assays including *ex vivo* T-cell suppression, Arg1/iNOS activity, ROS production, cytokine secretion, PD-L1–dependent suppression, and metabolic profiling (e.g., Seahorse-based analyses) to test whether specific promoter architectures predict suppressive capacity and subset-specific effector states.

## Data Availability

The datasets presented in this study can be found in online repositories. The names of the repository/repositories and accession number(s) can be found below: PRJNA1423754 (NCBI Sequence Read Archive) and GSE311155 (GEO). Not publicly available (scheduled to be released on Apr 01, 2026).
