# Supplementary material for: A murine model of sepsis induces age- and sex-specific chromatin remodeling in myeloid-derived suppressor cells
Source: Front Immunol. 2026 Mar 24;17:1750174. doi: 10.3389/fimmu.2026.1750174 (PMC13053262; doi:10.3389/fimmu.2026.1750174)

Supplementary File 1 to accompany Charles et al.

Heatmaps of HCG and GCH methylation patterns of targeted promoters in **Female mice**

Class 1 promoter: *S100a9*

Strongest response to CLP + DCS of all profiled promoters:

- Opens in most mice of all septic cohorts, with sex- and age-specific differences
- Nucleosome-free region (NFR) formation consistent with activated transcription
- Moderate levels of CpG methylation, with sepsis-specific demethylation

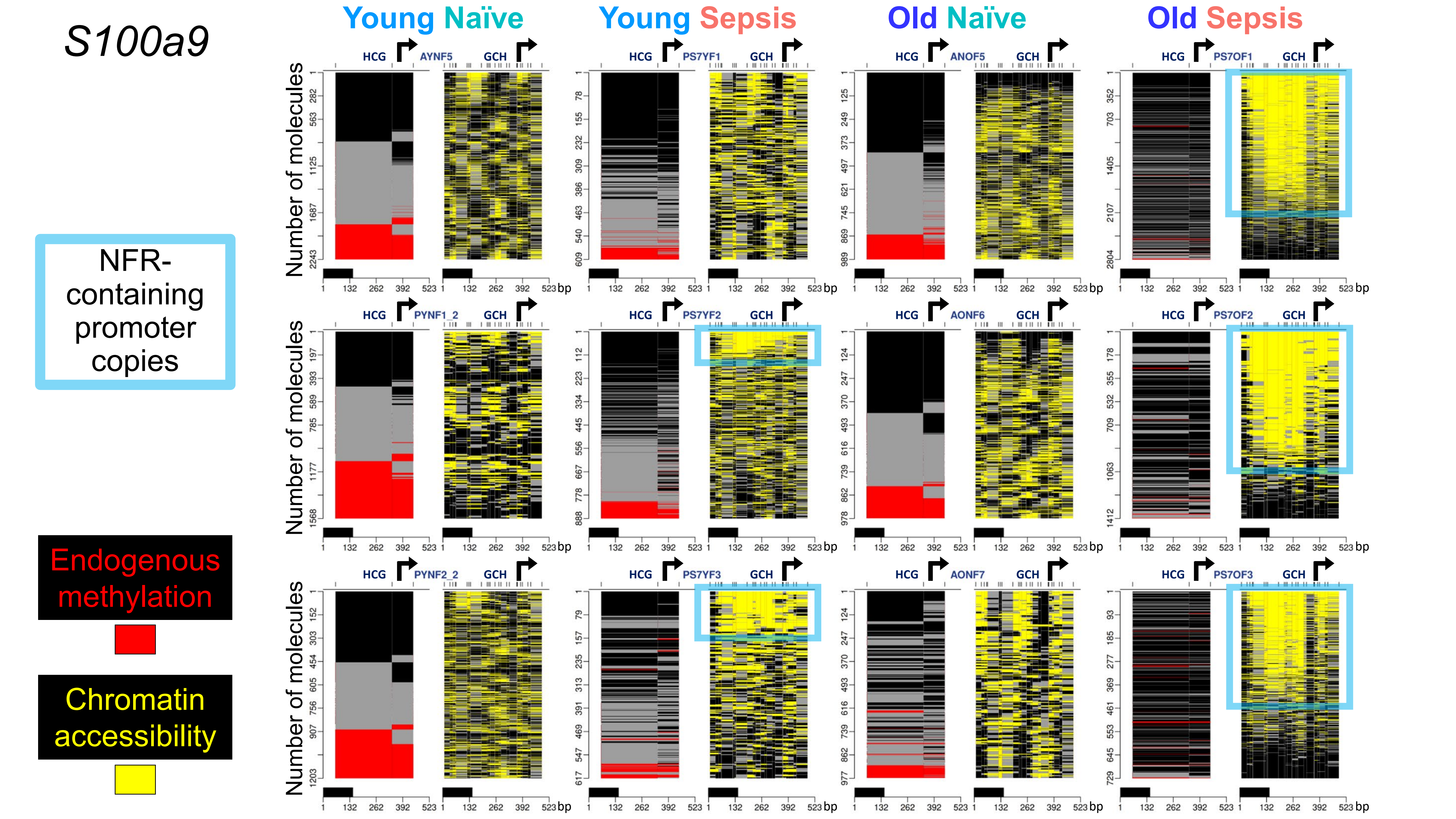

S100a9
Young Naïve
Young Sepsis
Old Naïve
Old Sepsis
HCG
GCH
AYNF5
PS7YF1
ANOF5
PS7OF1
PYNF1_2
PS7YF2
AONF6
PS7OF2
PYNF2_2
PS7YF3
AONF7
PS7OF3
Number of molecules
bp
NFR-containing promoter copies
Endogenous methylation
Chromatin accessibility

Class 2 promoters: *Cxcr2*, *Lcn2*, *Ccl3*, *Nos2*, *Ptgs2*, *Lgals9*, *Rnase2a*

Strong responses to CLP + DCS:

- NFR formation consistent with activated transcription
- Sex- and age-specific differences—promoter opening in Old Septic Female but in both Young and Old Septic Males
- Based on the fraction of NFR-bearing promoters, similar magnitude of sepsis response in both sexes
- CpG methylation levels ranging from background to fully methylated, with sepsis-specific demethylation at some loci

Note: Chromatin remodeling of Class 1 promoter and Class 3 promoters confirm CLP + DCS response in Female Young Sepsis mice, i.e., lack of accessibility is not due to a weak septic stimulus in some mice.

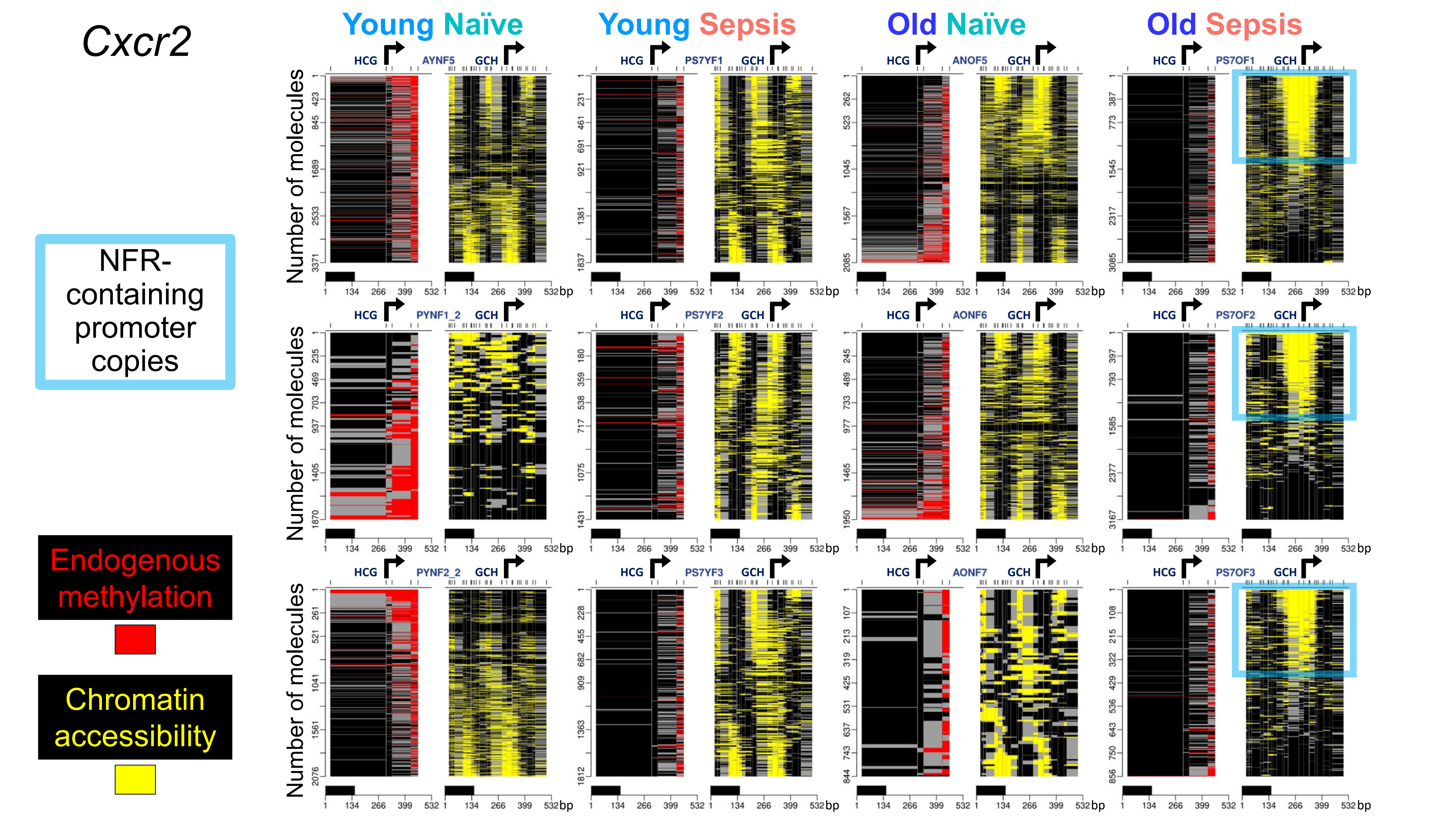

Cxcr2
Young Naïve
Young Sepsis
Old Naïve
Old Sepsis
NFR-containing promoter copies
Endogenous methylation
Chromatin accessibility
Number of molecules
HCG
GCH
AYNF5
PYNF1_2
PYNF2_2
PS7YF1
PS7YF2
PS7YF3
ANOF5
AONF6
AONF7
PS7OF1
PS7OF2
PS7OF3
1 134 266 399 532 bp

Lcn2

NFR-containing promoter copies

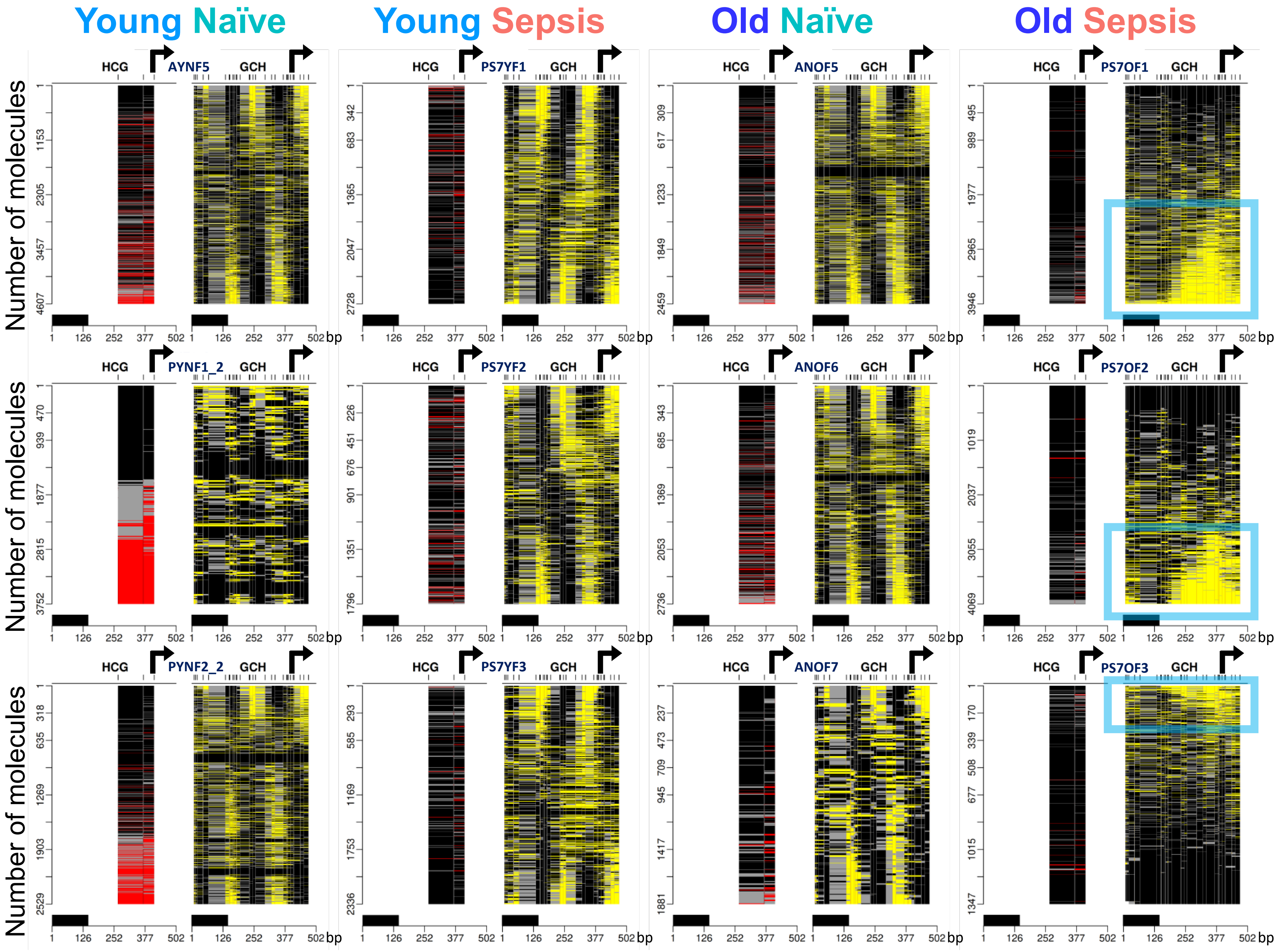

Endogenous methylation

Chromatin accessibility

Ccl3

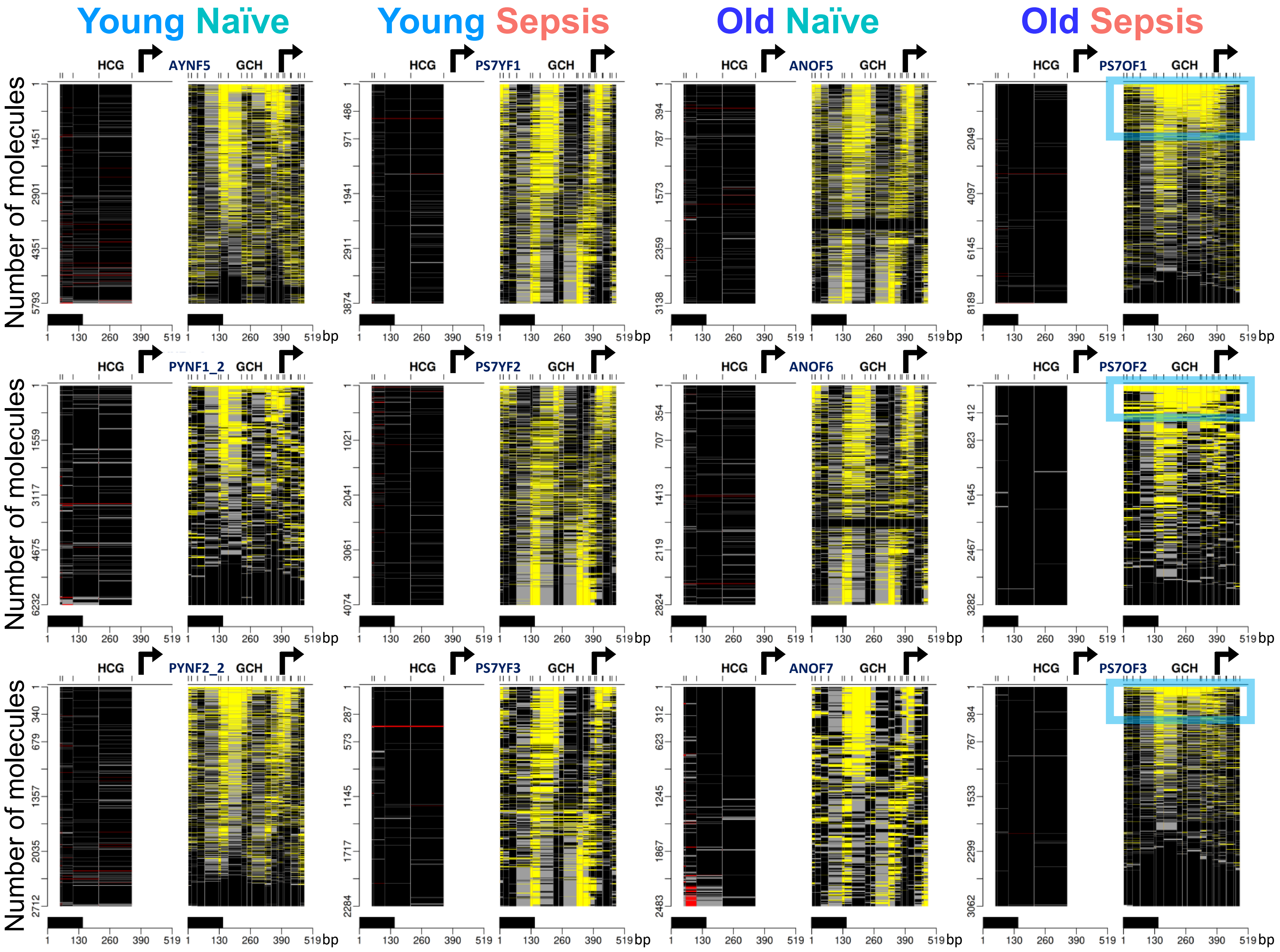

NFR-containing promoter copies

Endogenous methylation

Chromatin accessibility

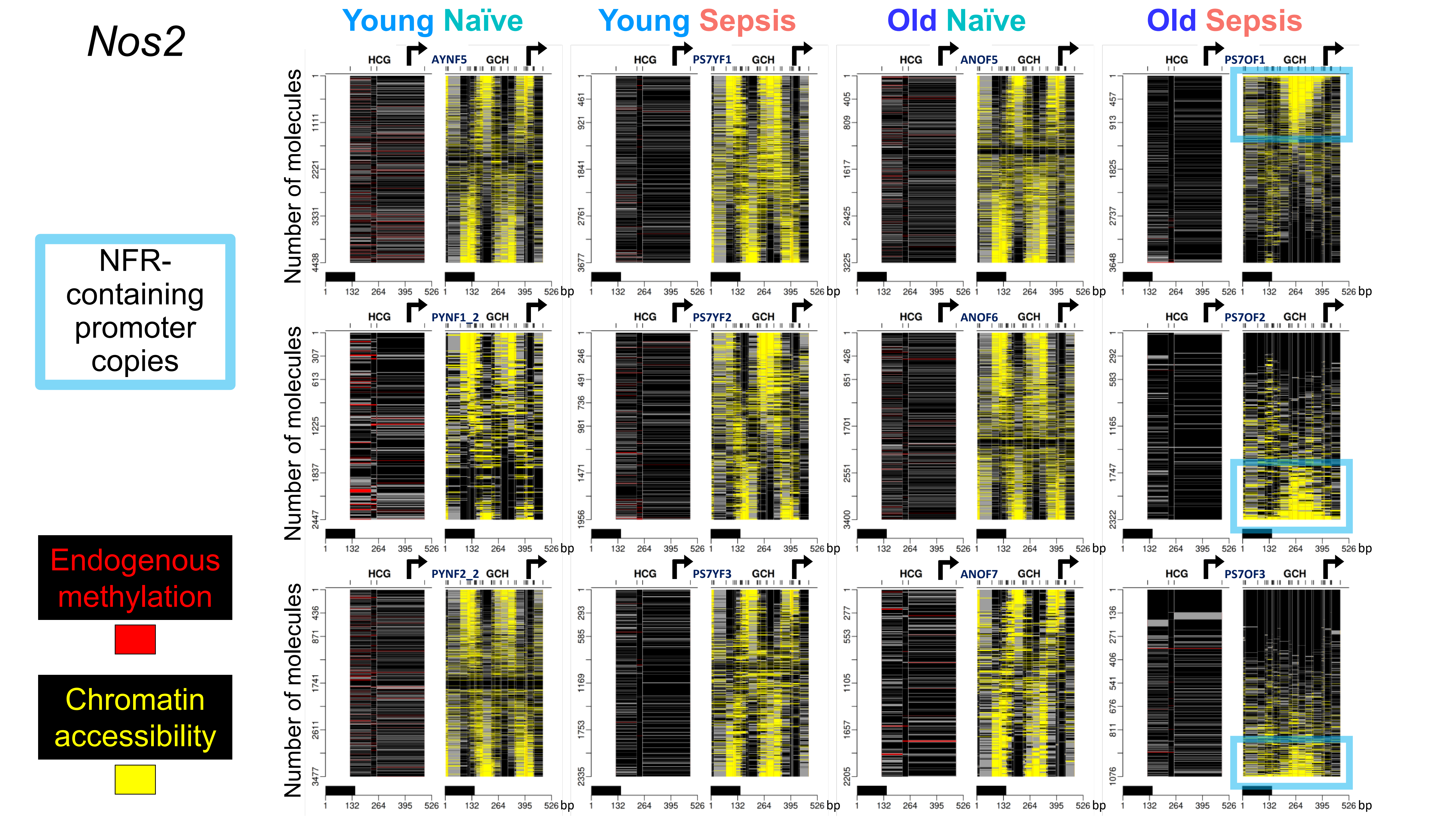
Nos2
Young Naïve
Young Sepsis
Old Naïve
Old Sepsis
NFR-containing promoter copies
Endogenous methylation
Chromatin accessibility
Number of molecules
HCG
GCH
AYNF5
PYNF1_2
PYNF2_2
PS7YF1
PS7YF2
PS7YF3
ANOF5
ANOF6
ANOF7
PS7OF1
PS7OF2
PS7OF3
1 132 264 395 526 bp

Ptgs2

NFR-containing promoter copies

Most HCG methylation likely arises from M.CviPI modification of accessible CCG sites

Endogenous methylation

Chromatin accessibility

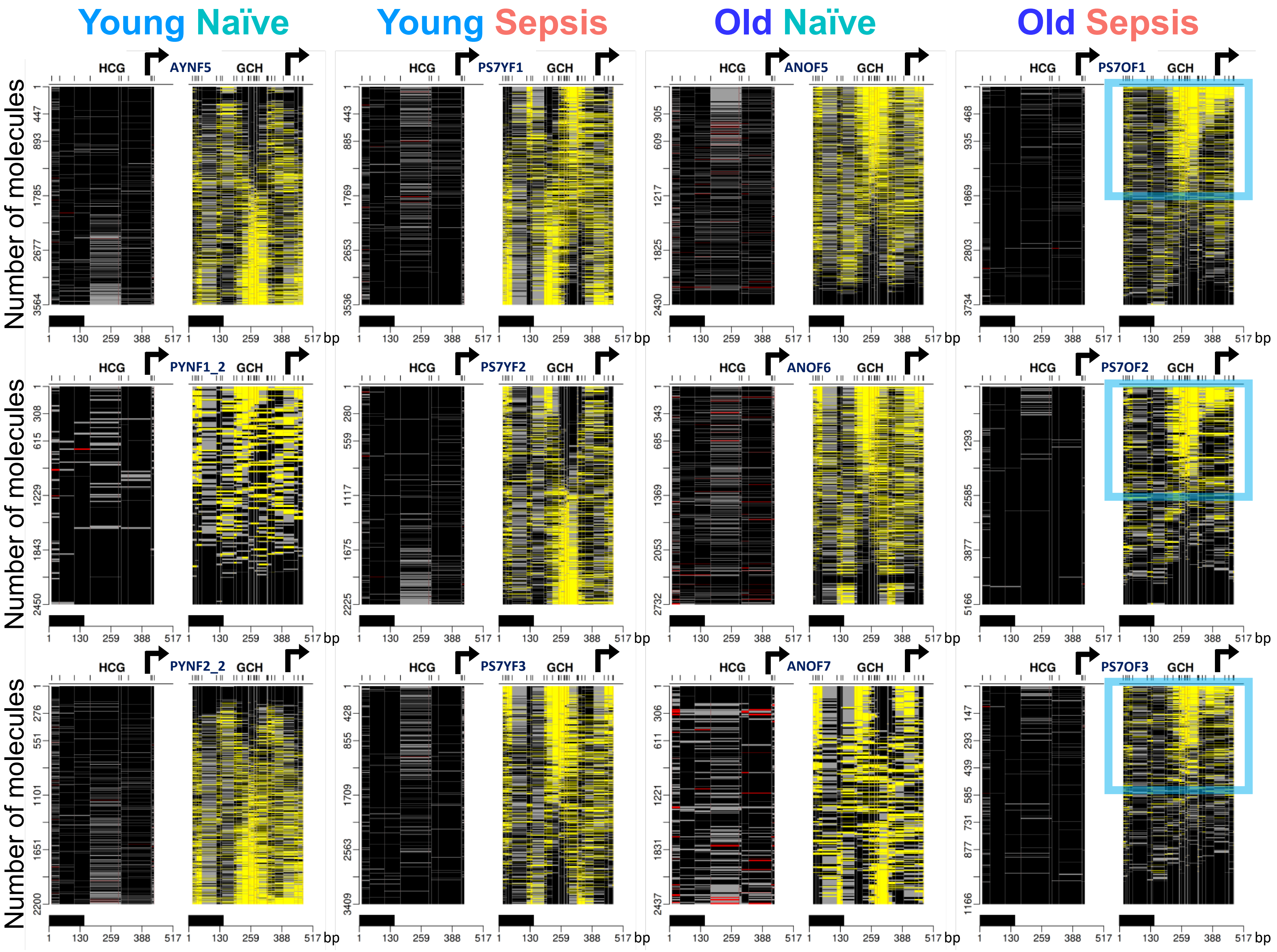

Lgals9

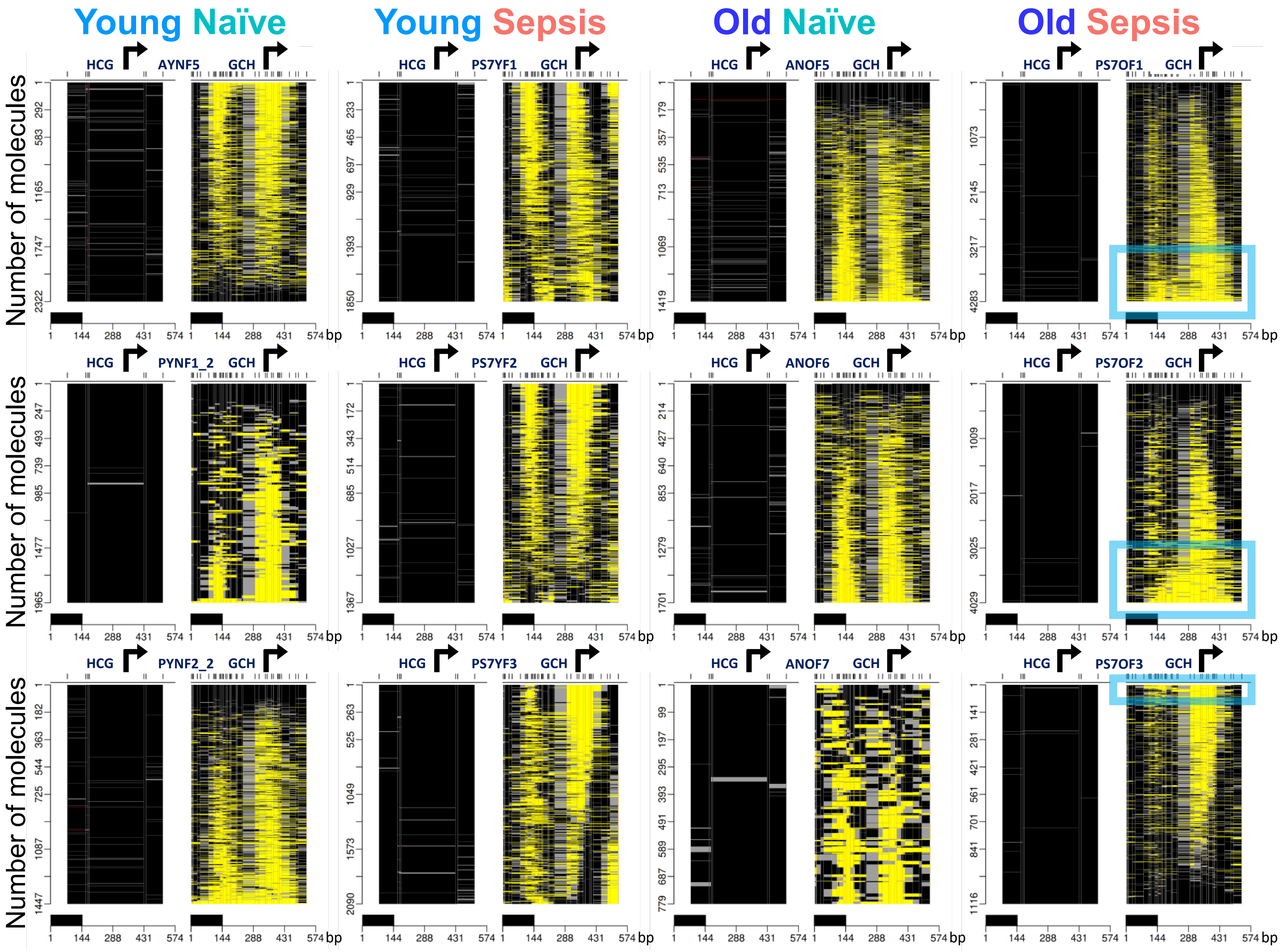

NFR-containing promoter copies

Endogenous methylation

Chromatin accessibility

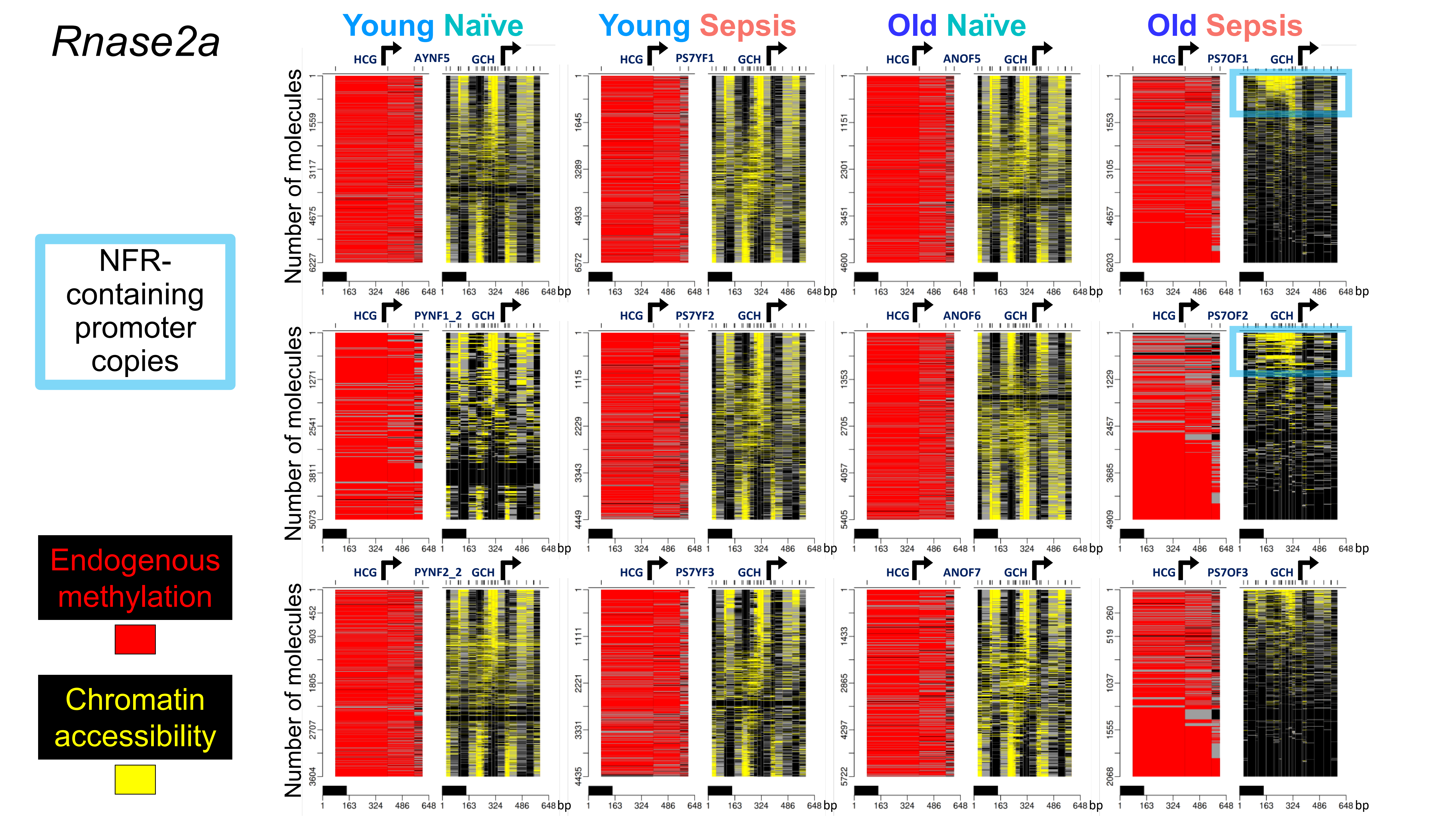
Rnase2a
Young Naïve
Young Sepsis
Old Naïve
Old Sepsis
NFR-containing promoter copies
Endogenous methylation
Chromatin accessibility
Number of molecules
HCG
GCH
AYNF5
PYNF1_2
PYNF2_2
PS7YF1
PS7YF2
PS7YF3
ANOF5
ANOF6
ANOF7
PS7OF1
PS7OF2
PS7OF3
1 163 324 486 648 bp

Class 3 promoters: *Mmp8*, *S100a8*

Response to CLP + DCS across <u>all</u> cohorts:

- No sex- or age-specific differences
- Nucleosome-free region (NFR) formation consistent with activated transcription
- Decreased accessibility upstream and downstream of NFRs at TSS
- Moderate levels of CpG methylation, with sepsis-specific demethylation

Note: Confirms chromatin remodeling in response to CLP + DCS in all male mice (even PS7YM3).

Mmp8

NFR-containing promoter copies

Most HCG methylation likely arises from M.CviPI modification of accessible CCG sites

Endogenous methylation

Chromatin accessibility

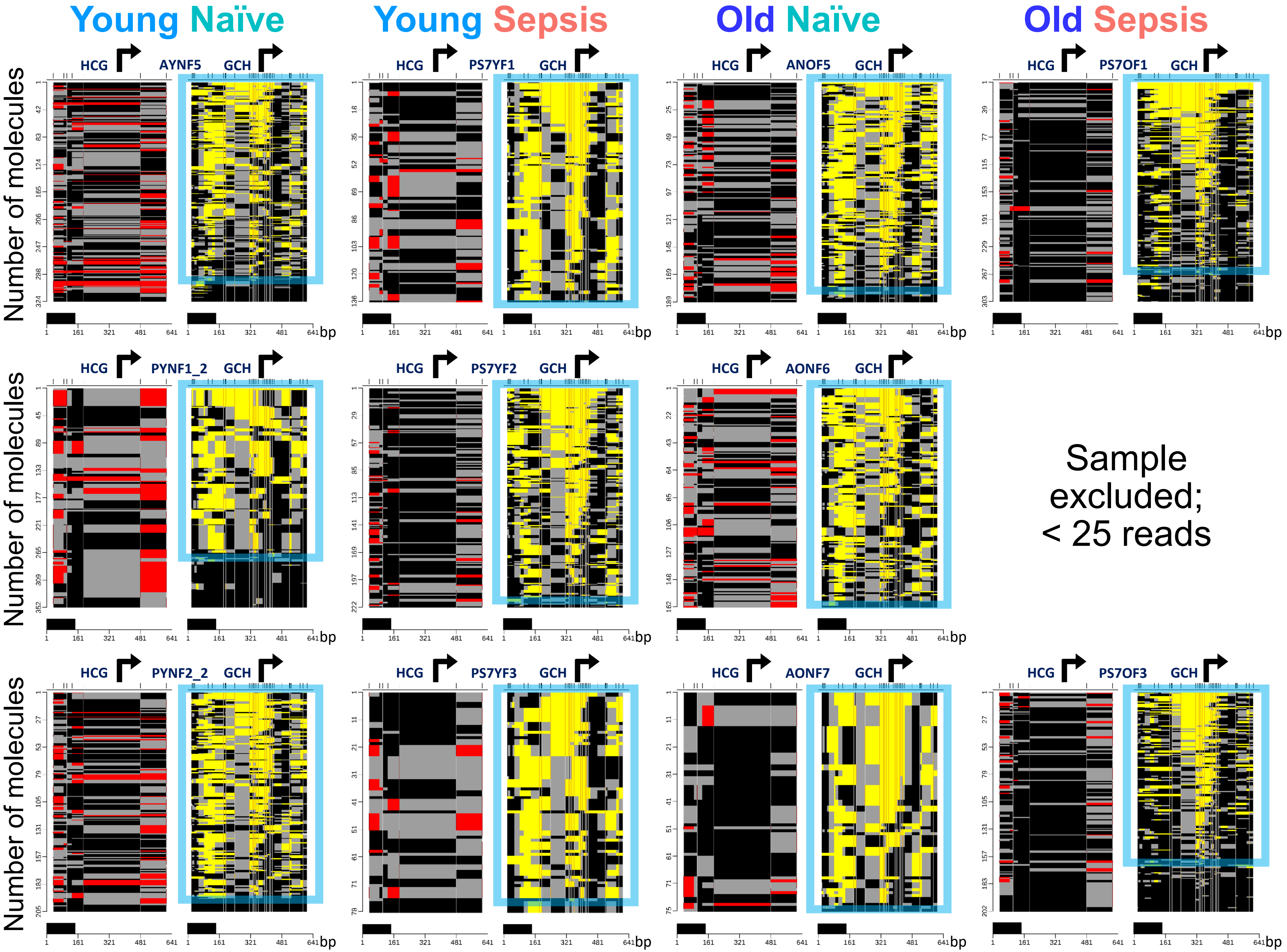

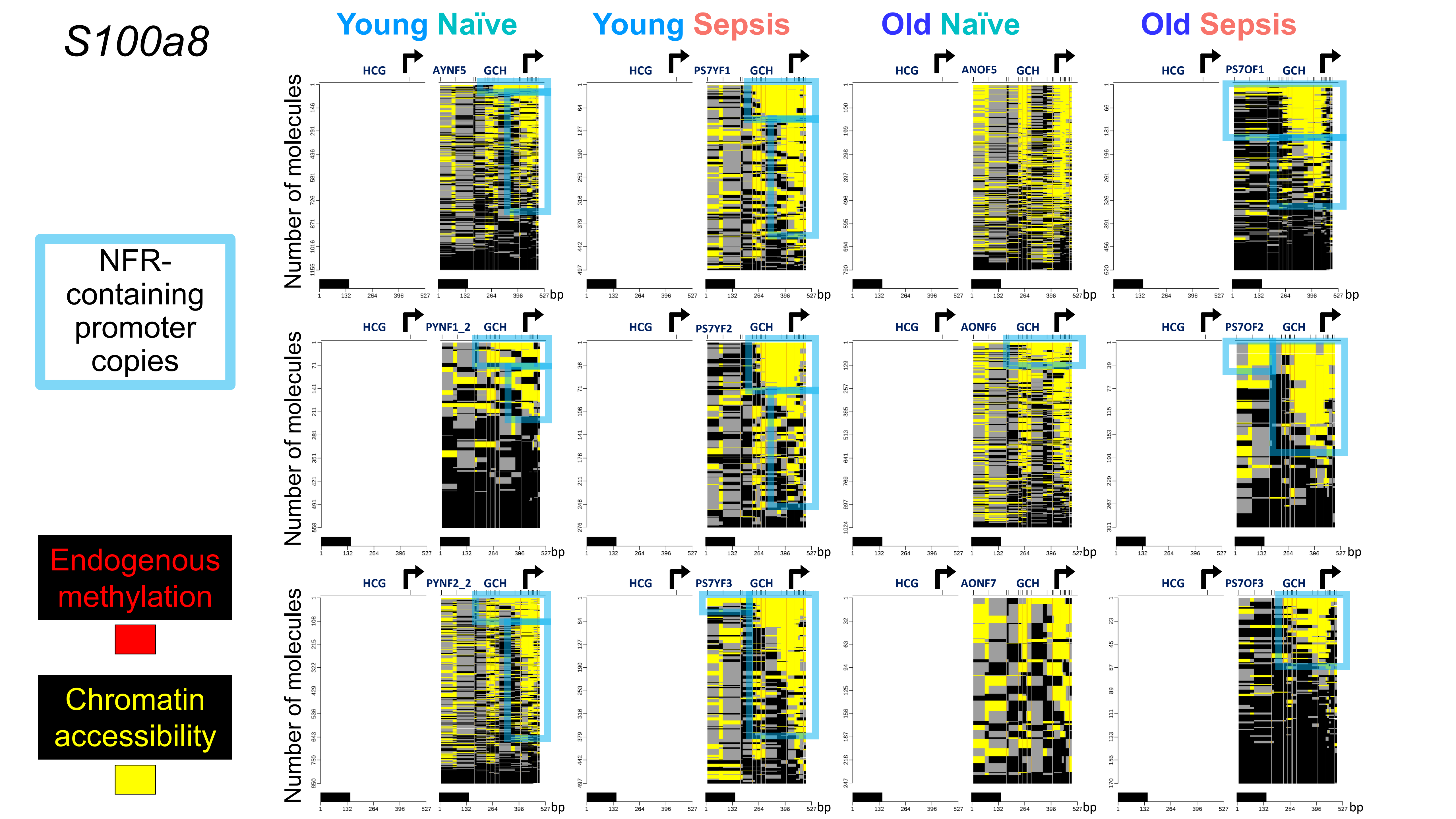
S100a8
Young Naïve
Young Sepsis
Old Naïve
Old Sepsis
HCG
GCH
AYNF5
PS7YF1
ANOF5
PS7OF1
PYNF1_2
PS7YF2
AONF6
PS7OF2
PYNF2_2
PS7YF3
AONF7
PS7OF3
Number of molecules
bp
NFR-containing promoter copies
Endogenous methylation
Chromatin accessibility

Class 4 promoter: *Fyb*

Response to CLP + DCS only in Female Septic mice (Young & Old):

- Strong sex divergence compared to Male Sepsis that showed no response
- Increased number of NFR-containing MDSCs in Female Septic mice, consistent with activated transcription
- Concomitant decrease in accessibility upstream and downstream of NFR at TSS
- Low, baseline levels of CpG methylation

Fyb

NFR-containing promoter copies

Most HCG methylation likely arises from M.CviPI modification of accessible CCG sites

Endogenous methylation

Chromatin accessibility

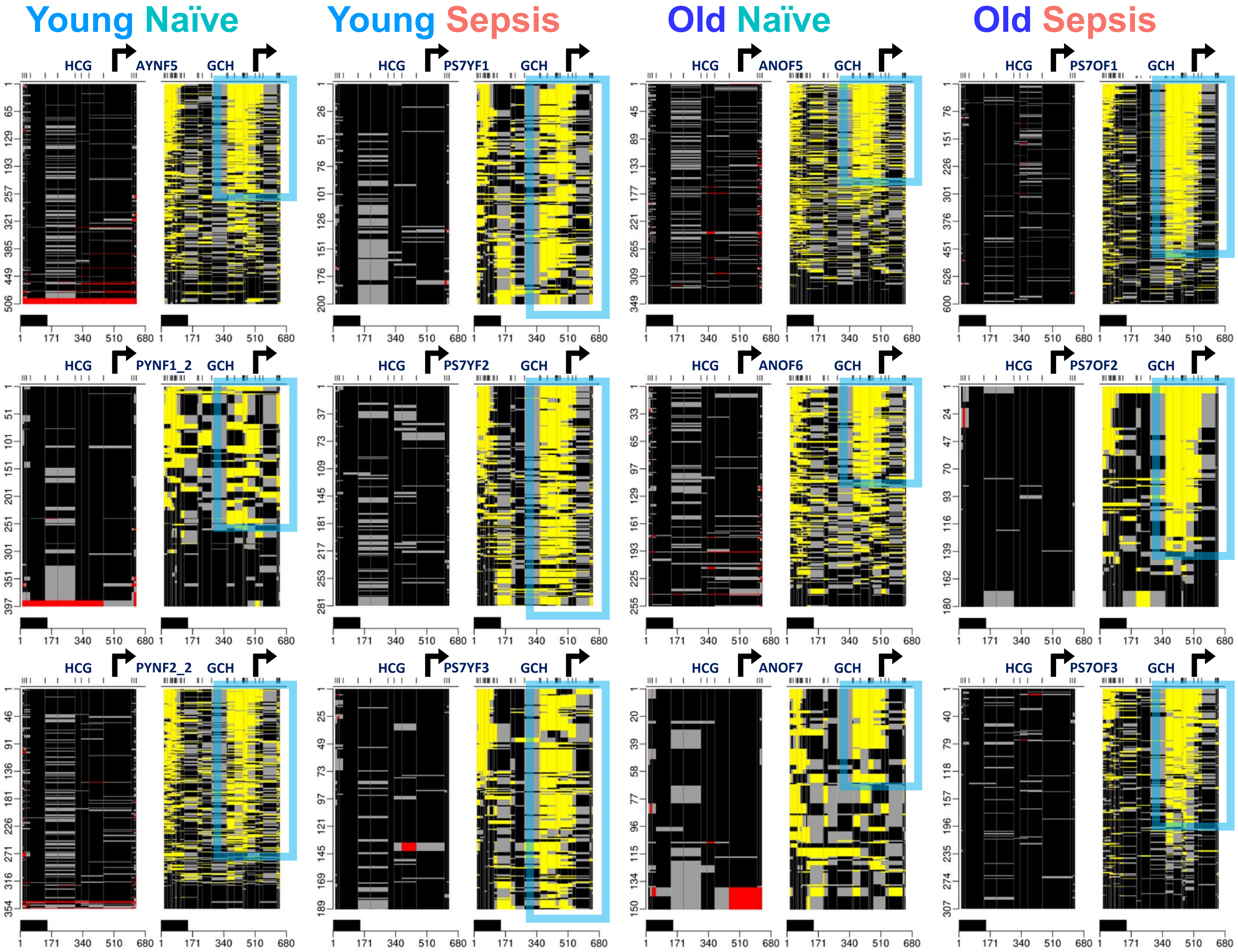

Class 5 promoters: *Lyz1*, *Retn*, *F7*, *Pmp22, Vnn1*, *Serpina1a*, *Emp1*, *Dab2, Vsig4*, *Mmp19*

<u>No</u> NRF formation in response to CLP + DCS across <u>all</u> cohorts:

- High levels of endogenous CpG methylation (at HCGs)
- Accessibility pattern consistent with disorganized or random nucleosome arrays
- Decreased accessibility in old sepsis samples

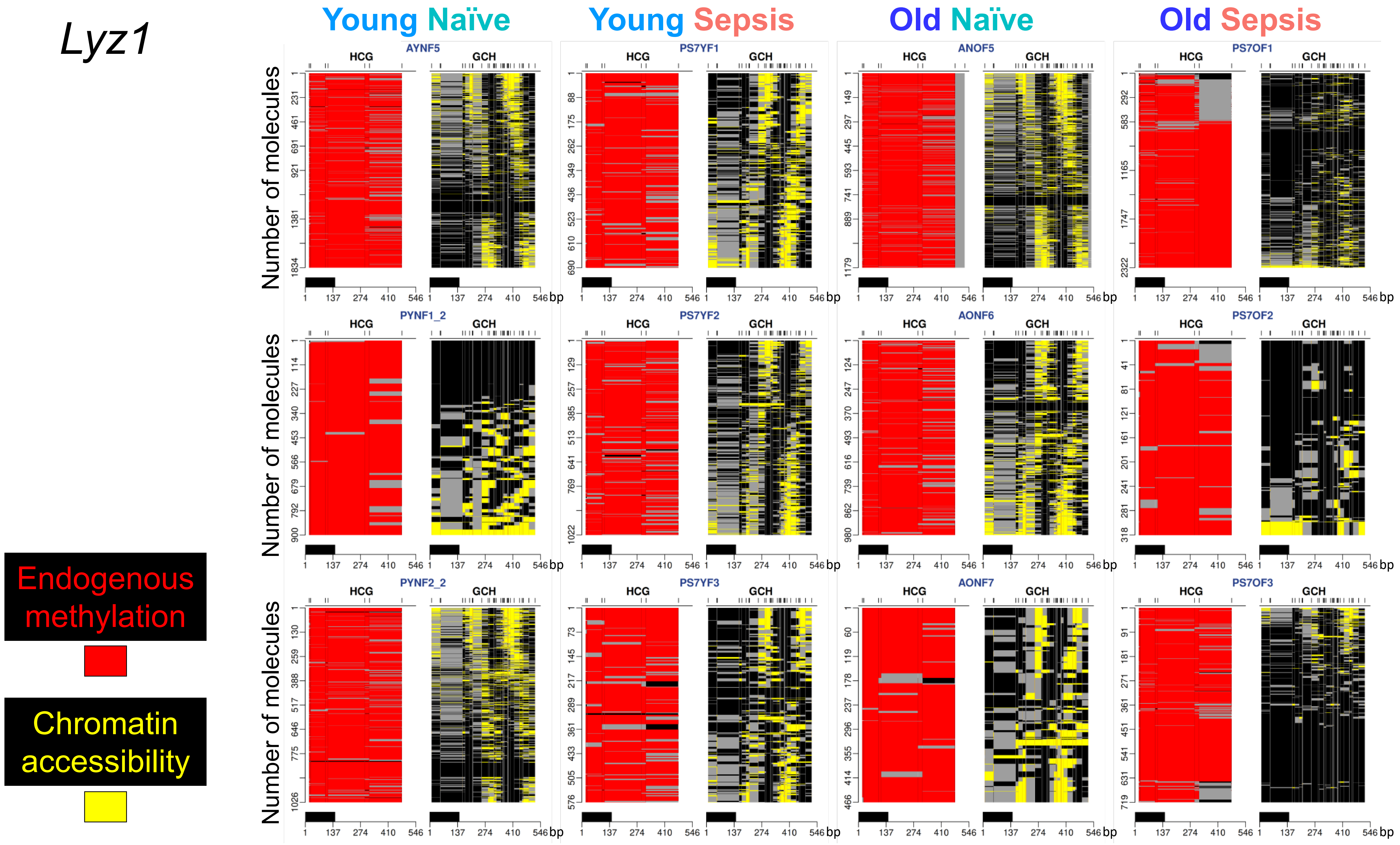
Lyz1
Young Naïve
Young Sepsis
Old Naïve
Old Sepsis
AYNF5
PYNF1_2
PYNF2_2
PS7YF1
PS7YF2
PS7YF3
ANOF5
AONF6
AONF7
PS7OF1
PS7OF2
PS7OF3
HCG
GCH
Number of molecules
bp
Endogenous methylation
Chromatin accessibility

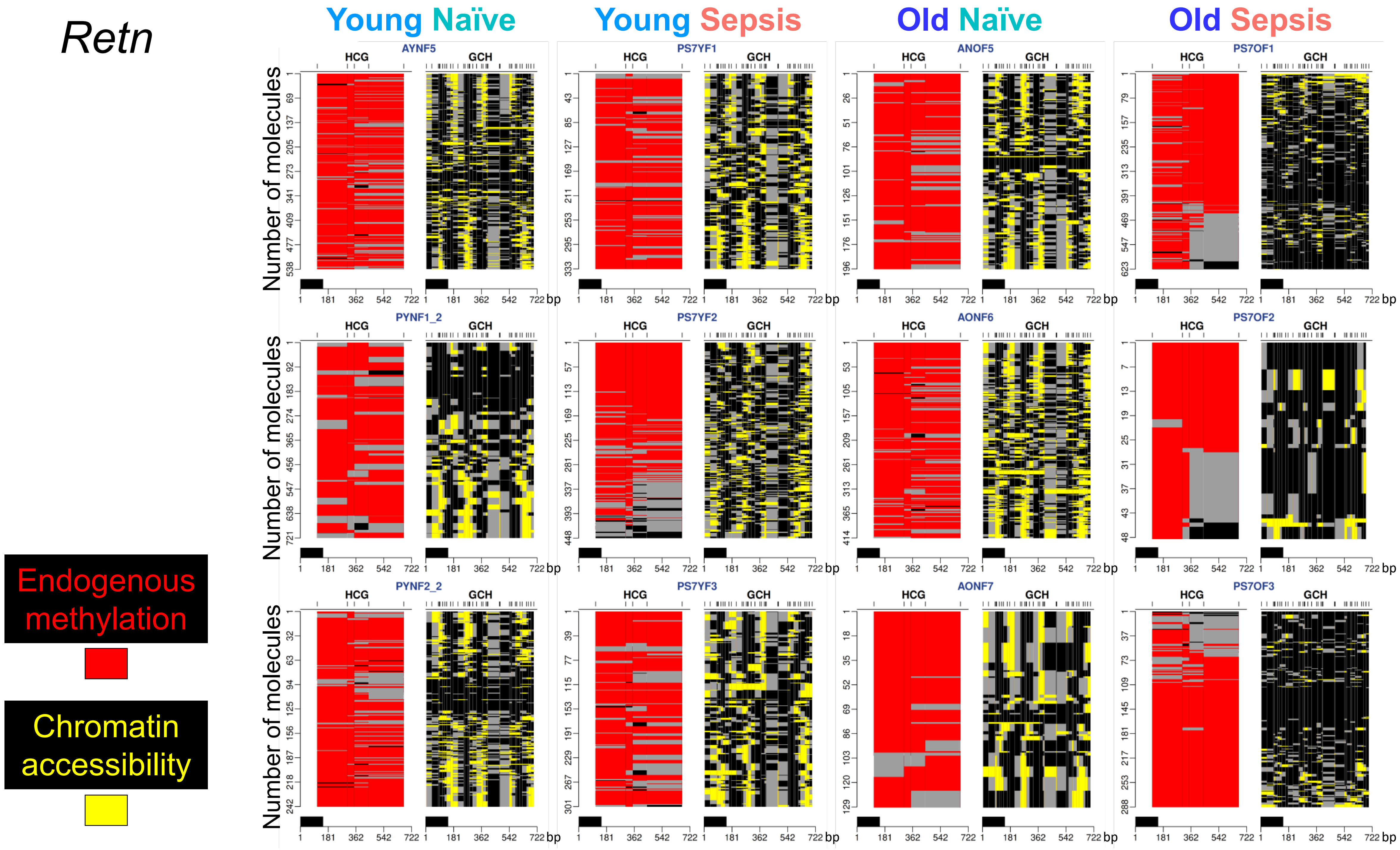
Retn
Young Naïve
Young Sepsis
Old Naïve
Old Sepsis
AYNF5
PS7YF1
ANOF5
PS7OF1
PYNF1_2
PS7YF2
AONF6
PS7OF2
PYNF2_2
PS7YF3
AONF7
PS7OF3
HCG
GCH
Number of molecules
1 181 362 542 722 bp
Endogenous methylation
Chromatin accessibility

F7

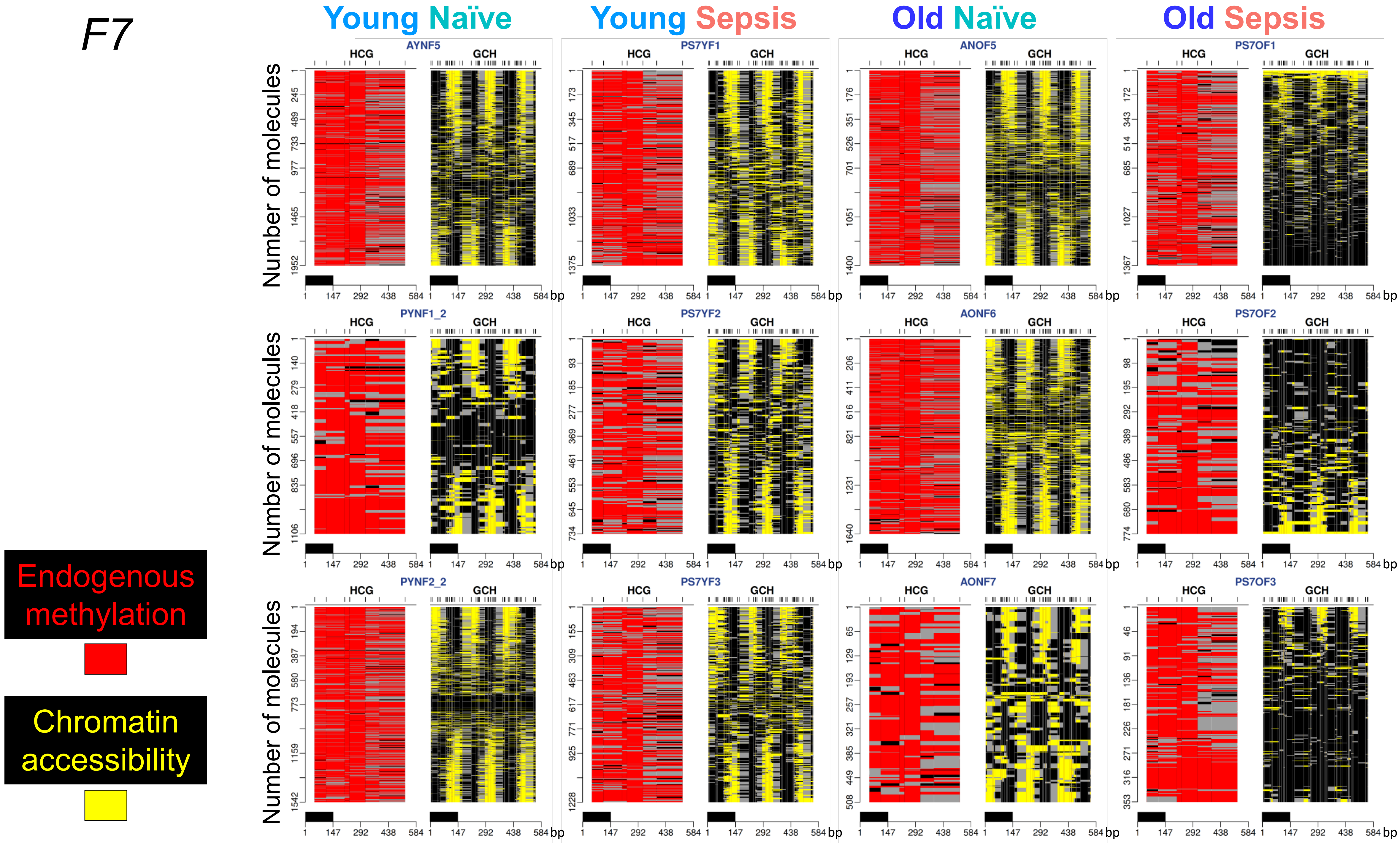
Young Naïve
Young Sepsis
Old Naïve
Old Sepsis
AYNF5
PS7YF1
ANOF5
PS7OF1
PYNF1_2
PS7YF2
AONF6
PS7OF2
PYNF2_2
PS7YF3
AONF7
PS7OF3
HCG
GCH
Number of molecules
1 147 292 438 584 bp
Endogenous methylation
Chromatin accessibility

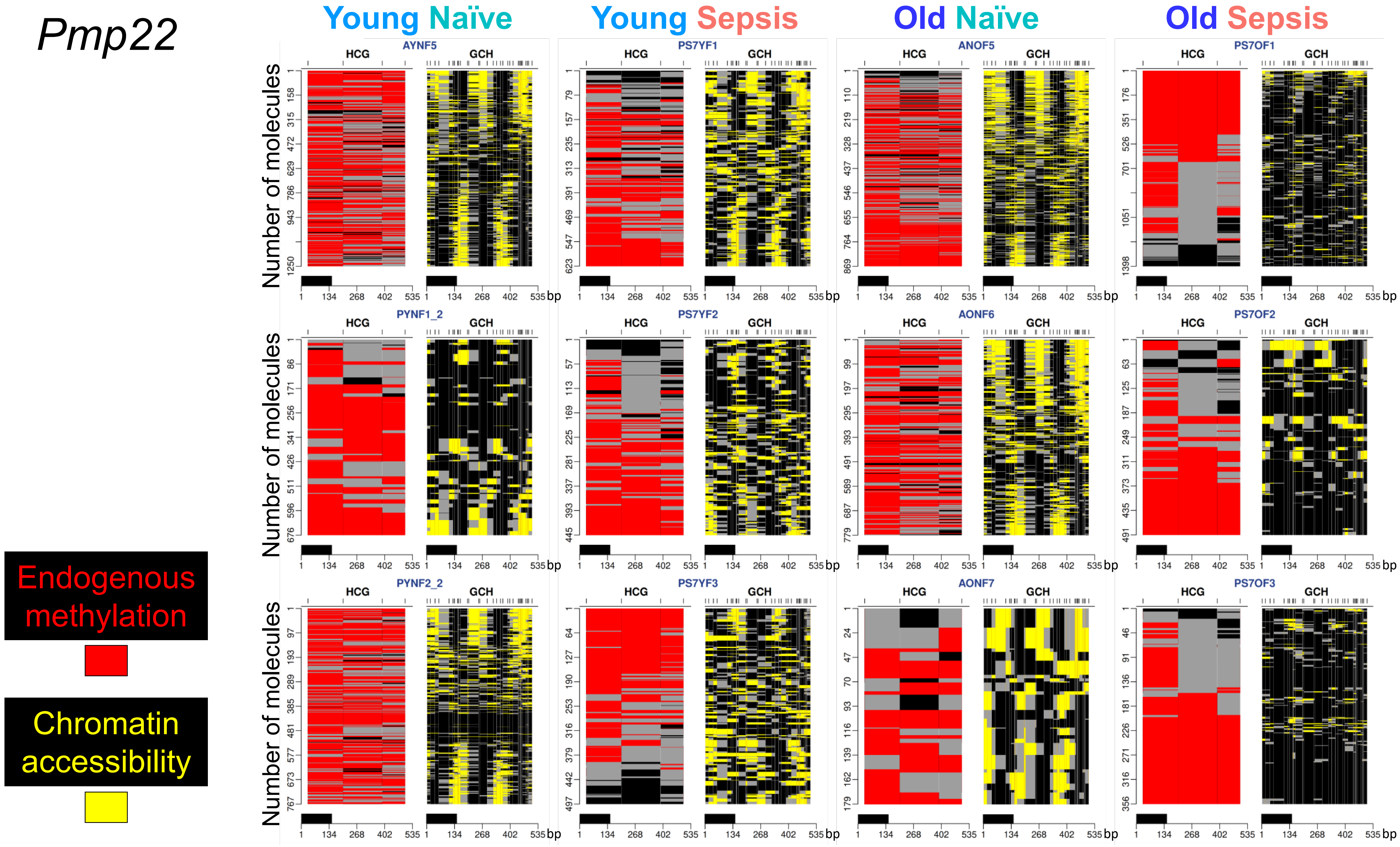
Pmp22
Young Naïve
Young Sepsis
Old Naïve
Old Sepsis
AYNF5
PS7YF1
ANOF5
PS7OF1
PYNF1_2
PS7YF2
AONF6
PS7OF2
PYNF2_2
PS7YF3
AONF7
PS7OF3
HCG
GCH
Number of molecules
1 134 268 402 535 bp
Endogenous methylation
Chromatin accessibility

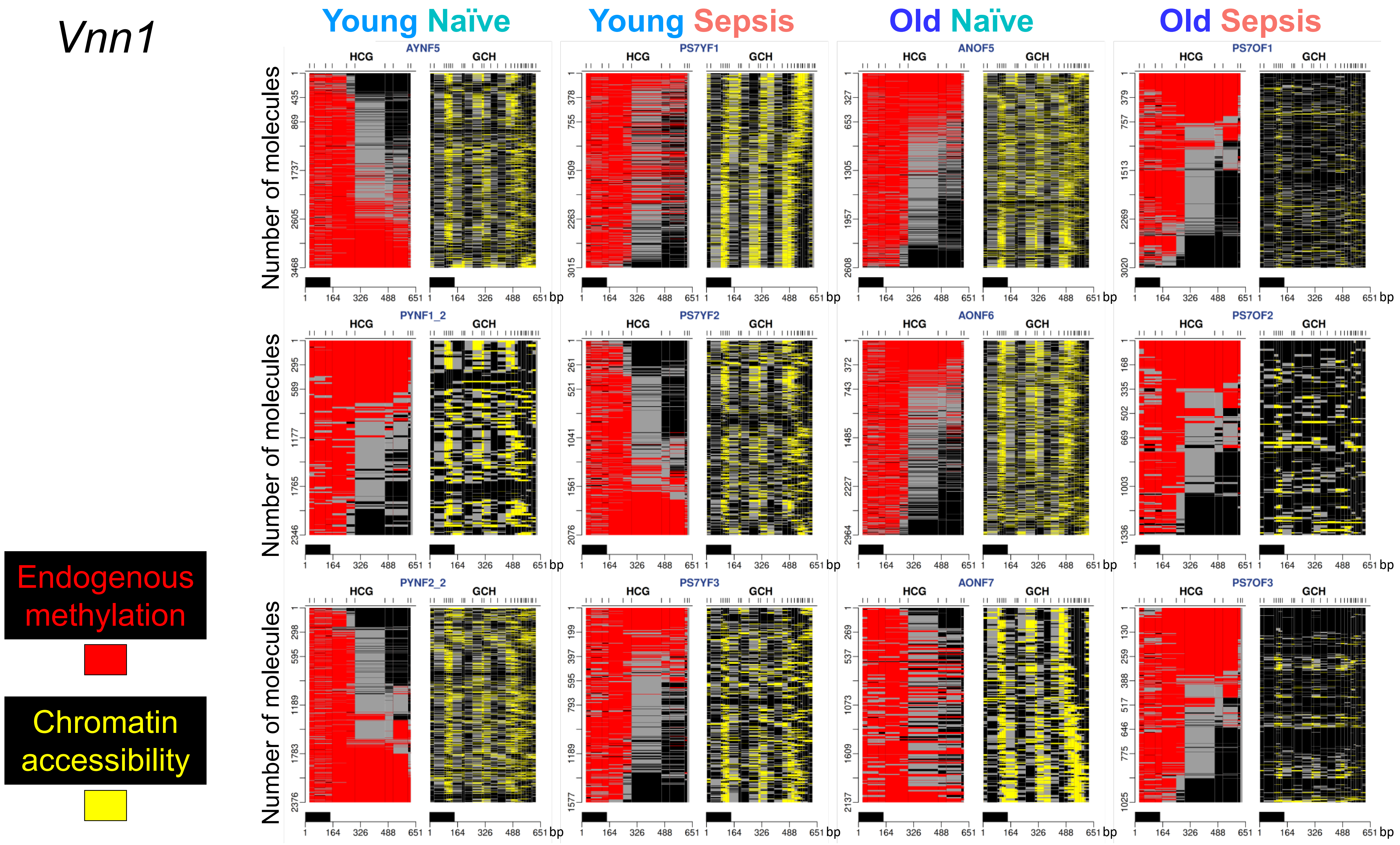
Vnn1
Young Naïve
Young Sepsis
Old Naïve
Old Sepsis
AYNF5
PS7YF1
ANOF5
PS7OF1
PYNF1_2
PS7YF2
AONF6
PS7OF2
PYNF2_2
PS7YF3
AONF7
PS7OF3
HCG
GCH
Number of molecules
bp
Endogenous methylation
Chromatin accessibility

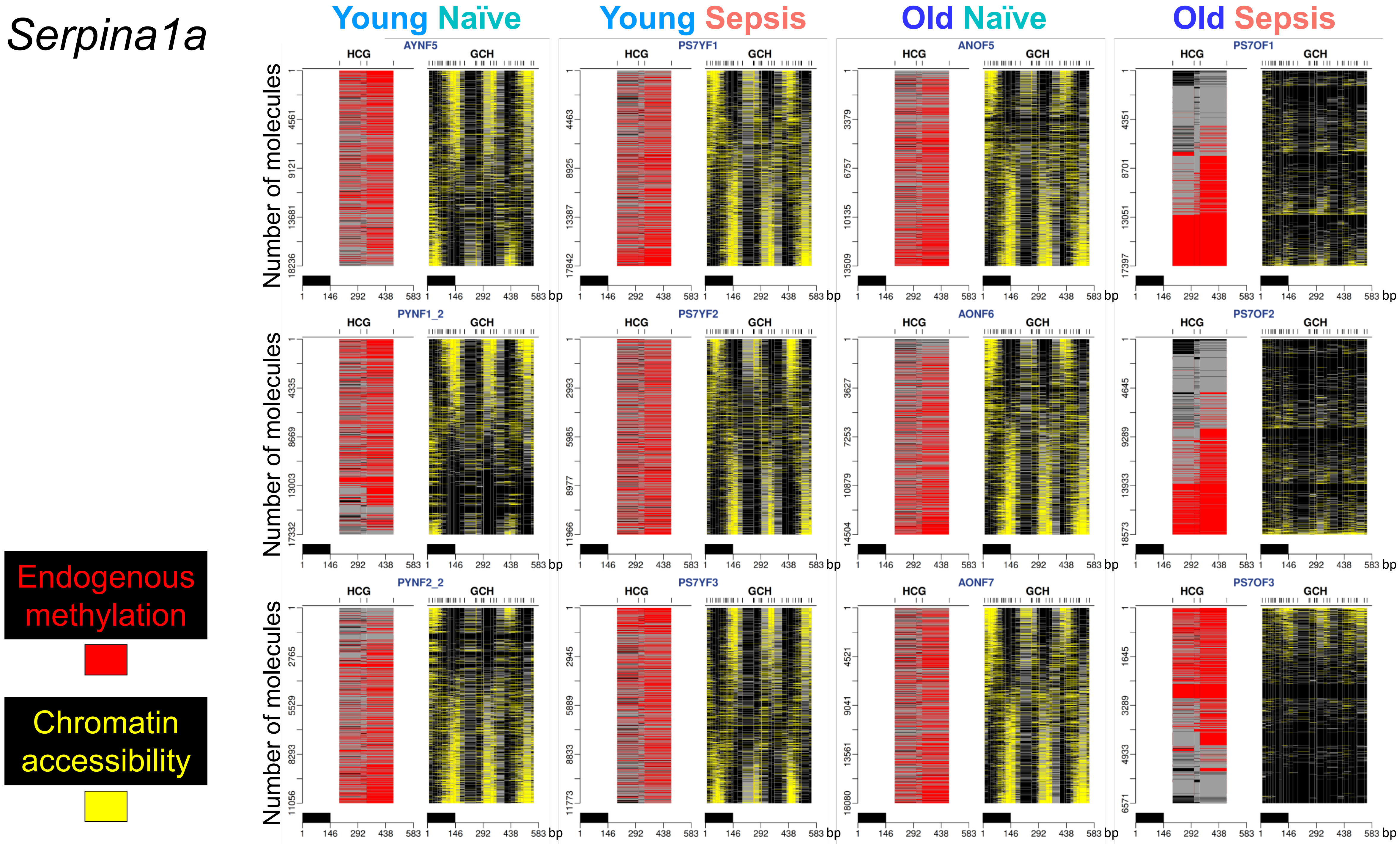

Serpina1a
Young Naïve
Young Sepsis
Old Naïve
Old Sepsis
AYNF5
PYNF1_2
PYNF2_2
PS7YF1
PS7YF2
PS7YF3
ANOF5
AONF6
AONF7
PS7OF1
PS7OF2
PS7OF3
HCG
GCH
Number of molecules
1
146
292
438
583
bp
Endogenous methylation
Chromatin accessibility

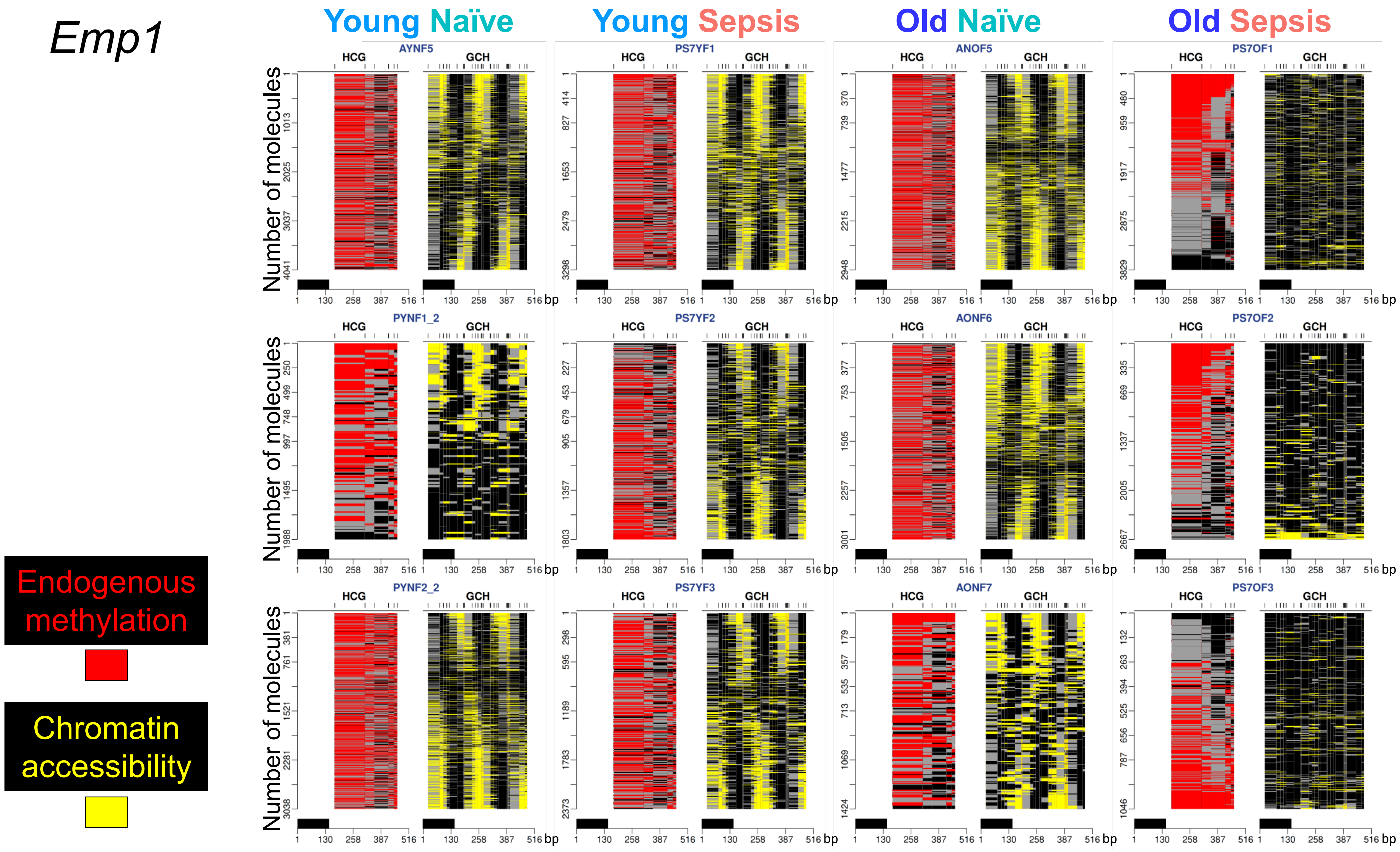

Emp1
Young Naïve
Young Sepsis
Old Naïve
Old Sepsis
AYNF5
PS7YF1
ANOF5
PS7OF1
PYNF1_2
PS7YF2
AONF6
PS7OF2
PYNF2_2
PS7YF3
AONF7
PS7OF3
HCG
GCH
Number of molecules
bp
Endogenous methylation
Chromatin accessibility

Dab2

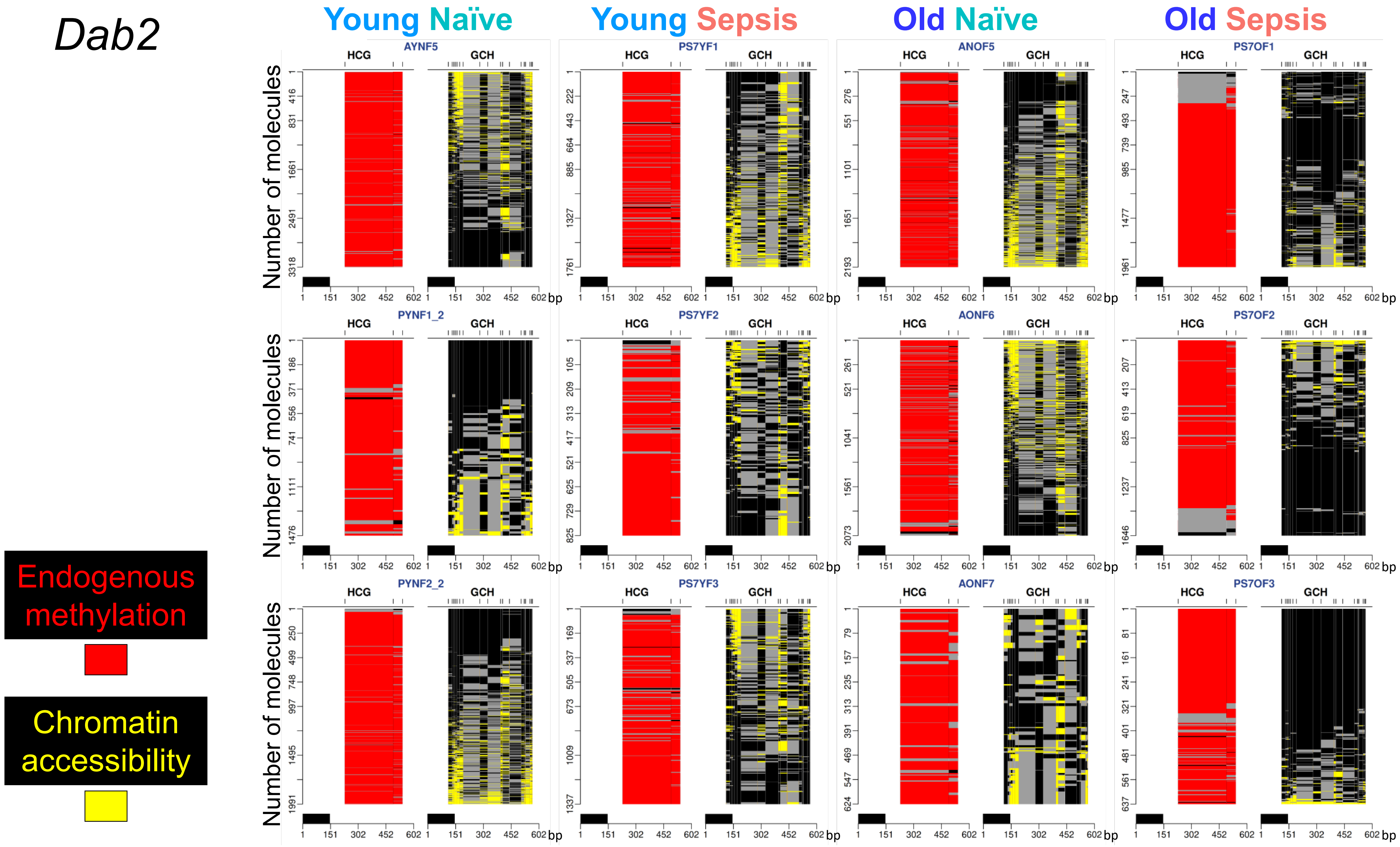
Young Naïve
Young Sepsis
Old Naïve
Old Sepsis
AYNF5
PS7YF1
ANOF5
PS7OF1
PYNF1_2
PS7YF2
ANOF6
PS7OF2
PYNF2_2
PS7YF3
AONF7
PS7OF3
HCG
GCH
Number of molecules
bp
Endogenous methylation
Chromatin accessibility

Vsig4

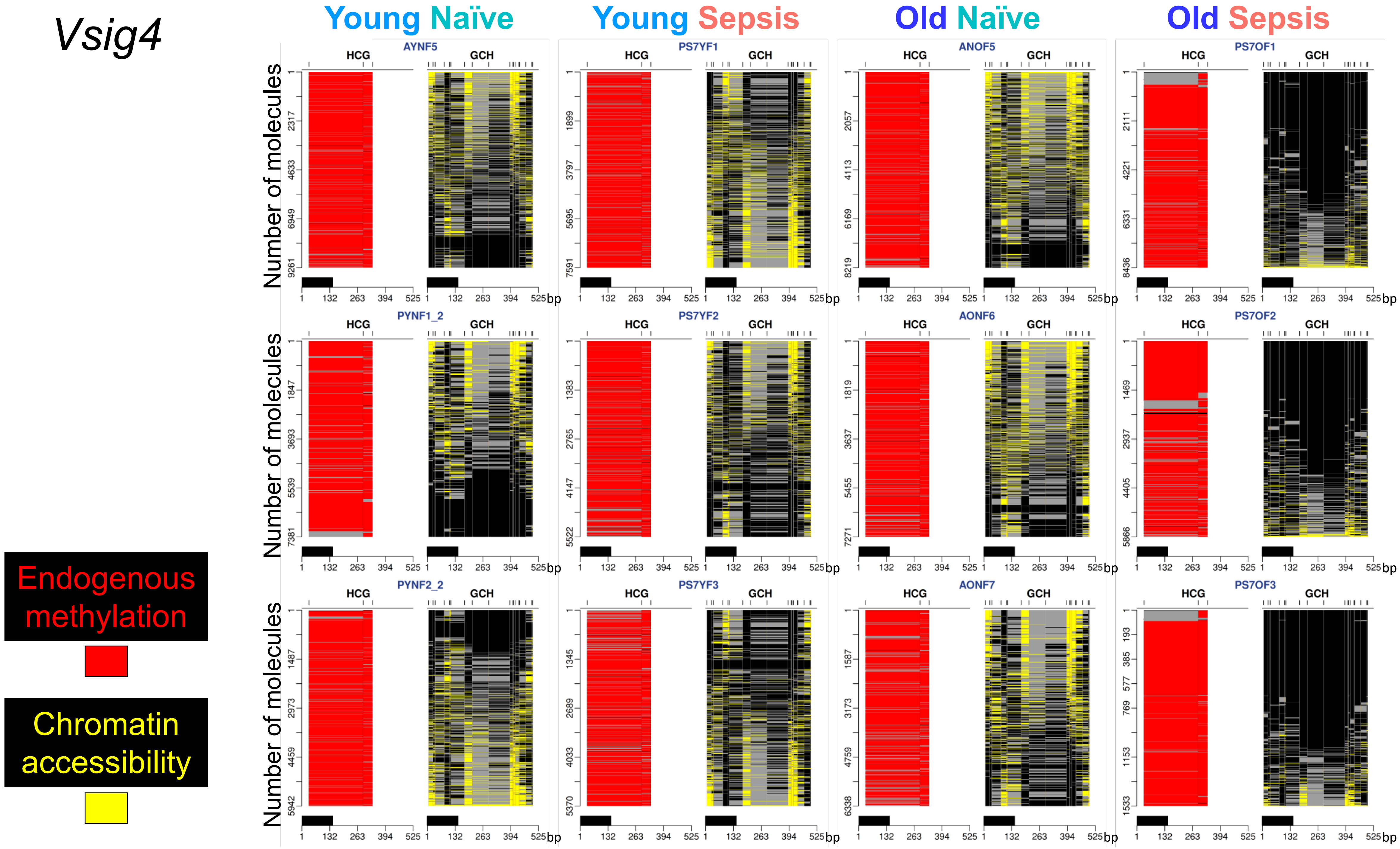

Young Naïve
Young Sepsis
Old Naïve
Old Sepsis
AYNF5
PS7YF1
ANOF5
PS7OF1
PYNF1_2
PS7YF2
AONF6
PS7OF2
PYNF2_2
PS7YF3
AONF7
PS7OF3
HCG
GCH
Number of molecules
1
132
263
394
525
bp
Endogenous methylation
Chromatin accessibility

Mmp19

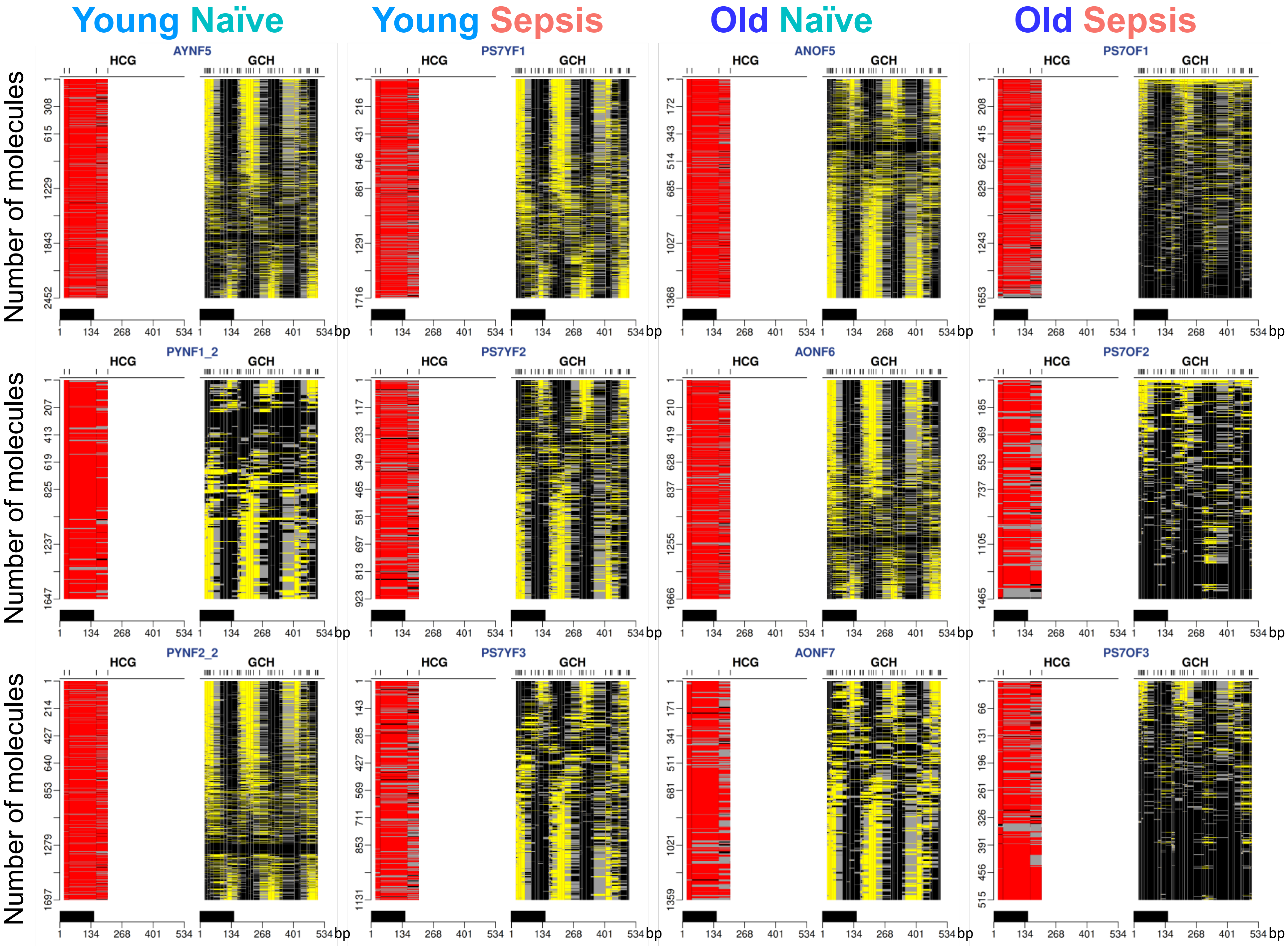
Young Naïve
Young Sepsis
Old Naïve
Old Sepsis
AYNF5
PYNF1_2
PYNF2_2
PS7YF1
PS7YF2
PS7YF3
ANOF5
AONF6
AONF7
PS7OF1
PS7OF2
PS7OF3
HCG
GCH
Number of molecules
1 134 268 401 534 bp

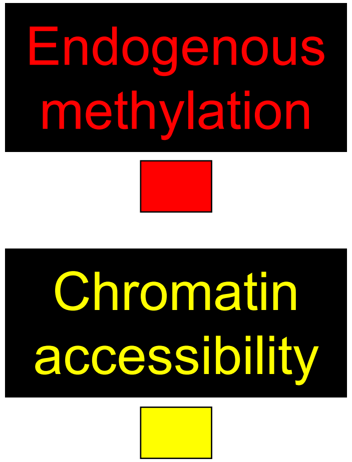
Endogenous methylation
Chromatin accessibility

Class 6 promoters: *Serpine1*, *Lgals2*, *Ccl5*, *Fabp7*, *Ambp*, *Kdm6b*, *Fgr*, *Gpnmb*, *Cd9*, *Cxcl3*, *Mmp9*

No NRF formation in response to CLP + DCS across all cohorts:

- Intermediate levels of endogenous CpG methylation (at HCGs)
- Accessibility pattern consistent with disorganized or random nucleosome arrays
- Decreased accessibility in old sepsis samples

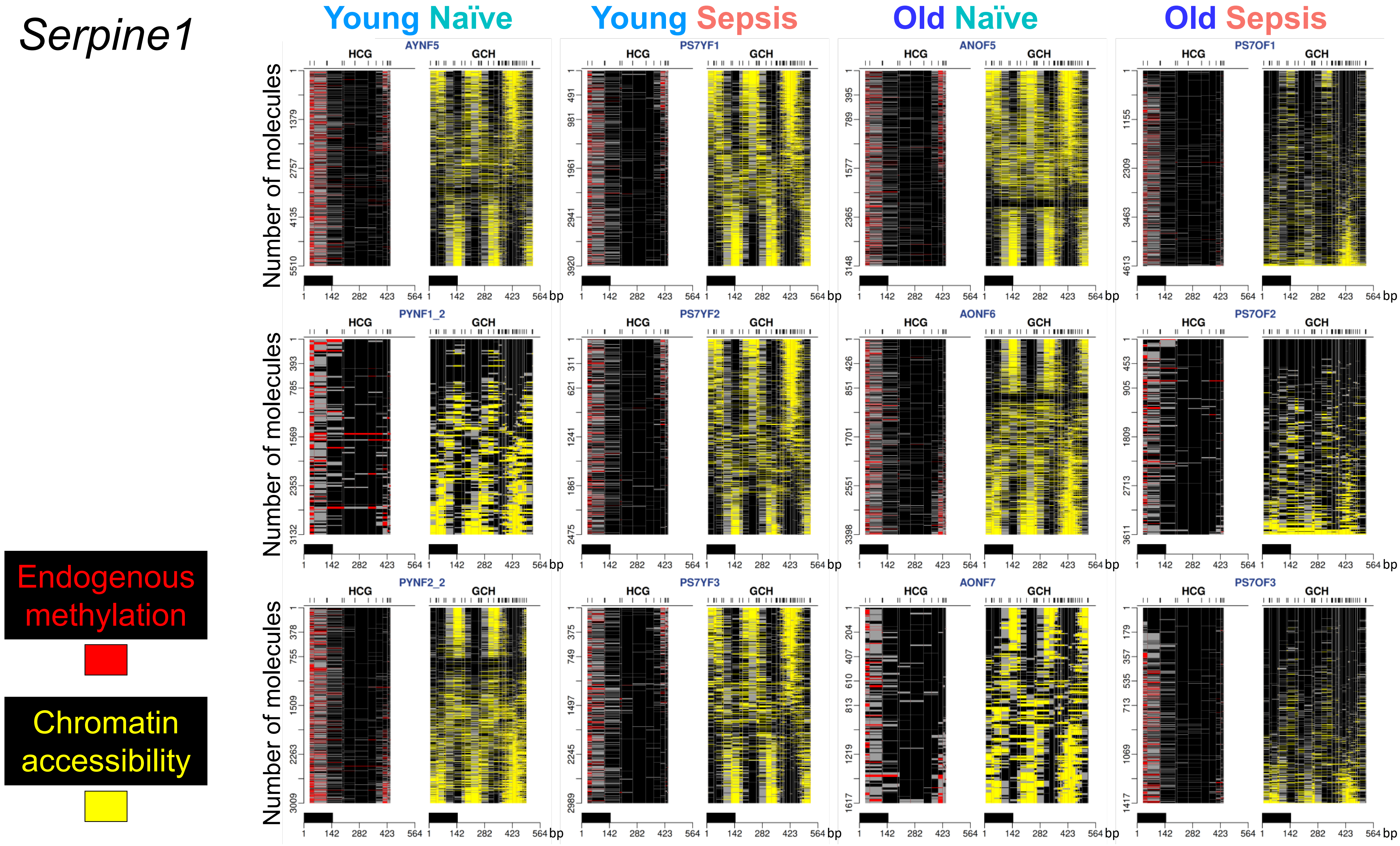

Serpine1
Young Naïve
Young Sepsis
Old Naïve
Old Sepsis
AYNF5
PS7YF1
ANOF5
PS7OF1
PYNF1_2
PS7YF2
AONF6
PS7OF2
PYNF2_2
PS7YF3
AONF7
PS7OF3
HCG
GCH
Number of molecules
1 142 282 423 564 bp
Endogenous methylation
Chromatin accessibility

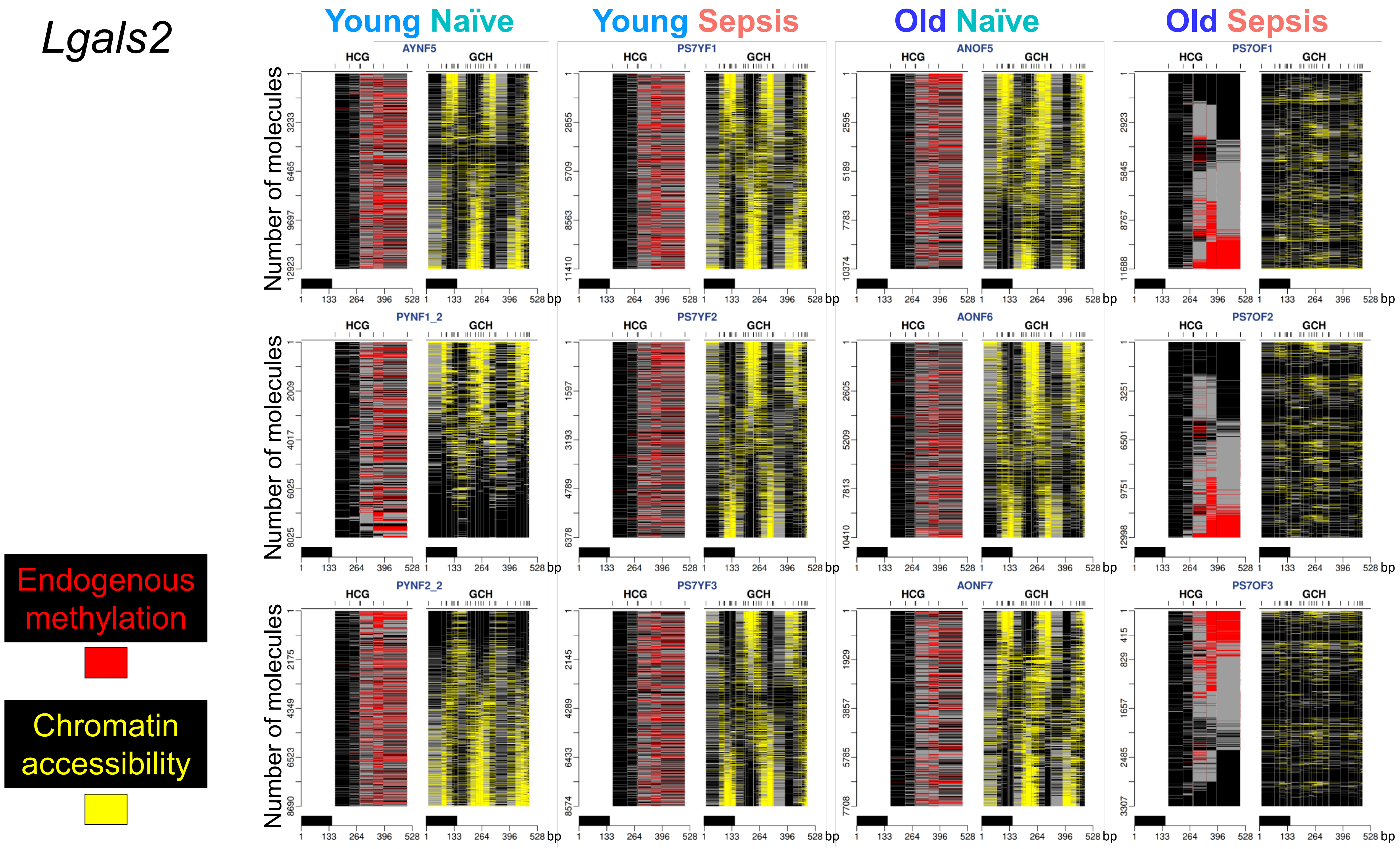

Lgals2
Young Naïve
Young Sepsis
Old Naïve
Old Sepsis
AYNF5
PYNF1_2
PYNF2_2
PS7YF1
PS7YF2
PS7YF3
ANOF5
AONF6
AONF7
PS7OF1
PS7OF2
PS7OF3
HCG
GCH
Number of molecules
bp
Endogenous methylation
Chromatin accessibility

Ccl5

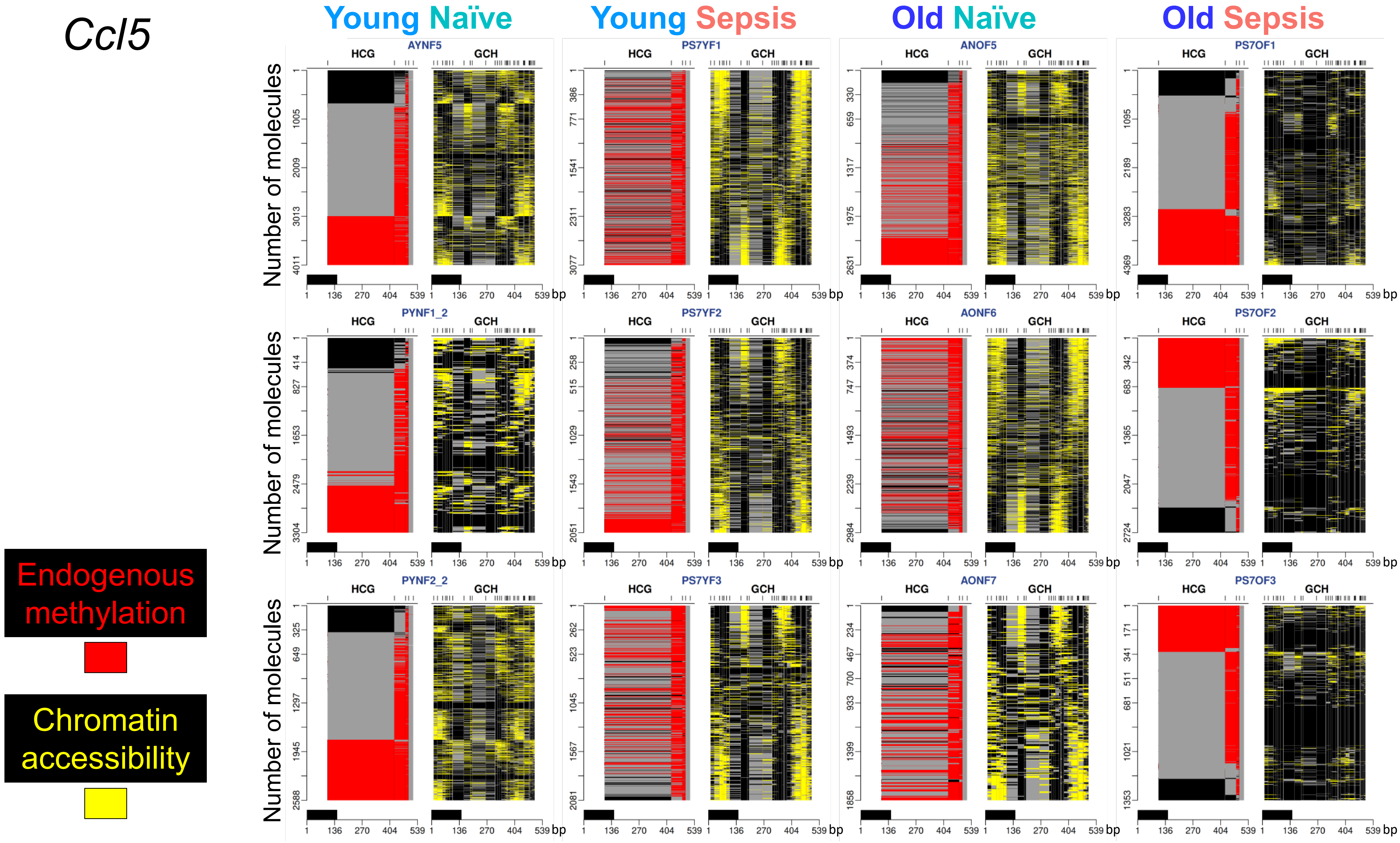

Young Naïve
Young Sepsis
Old Naïve
Old Sepsis
AYNF5
PYNF1_2
PYNF2_2
PS7YF1
PS7YF2
PS7YF3
ANOF5
AONF6
AONF7
PS7OF1
PS7OF2
PS7OF3
HCG
GCH
Number of molecules
1 136 270 404 539 bp
Endogenous methylation
Chromatin accessibility

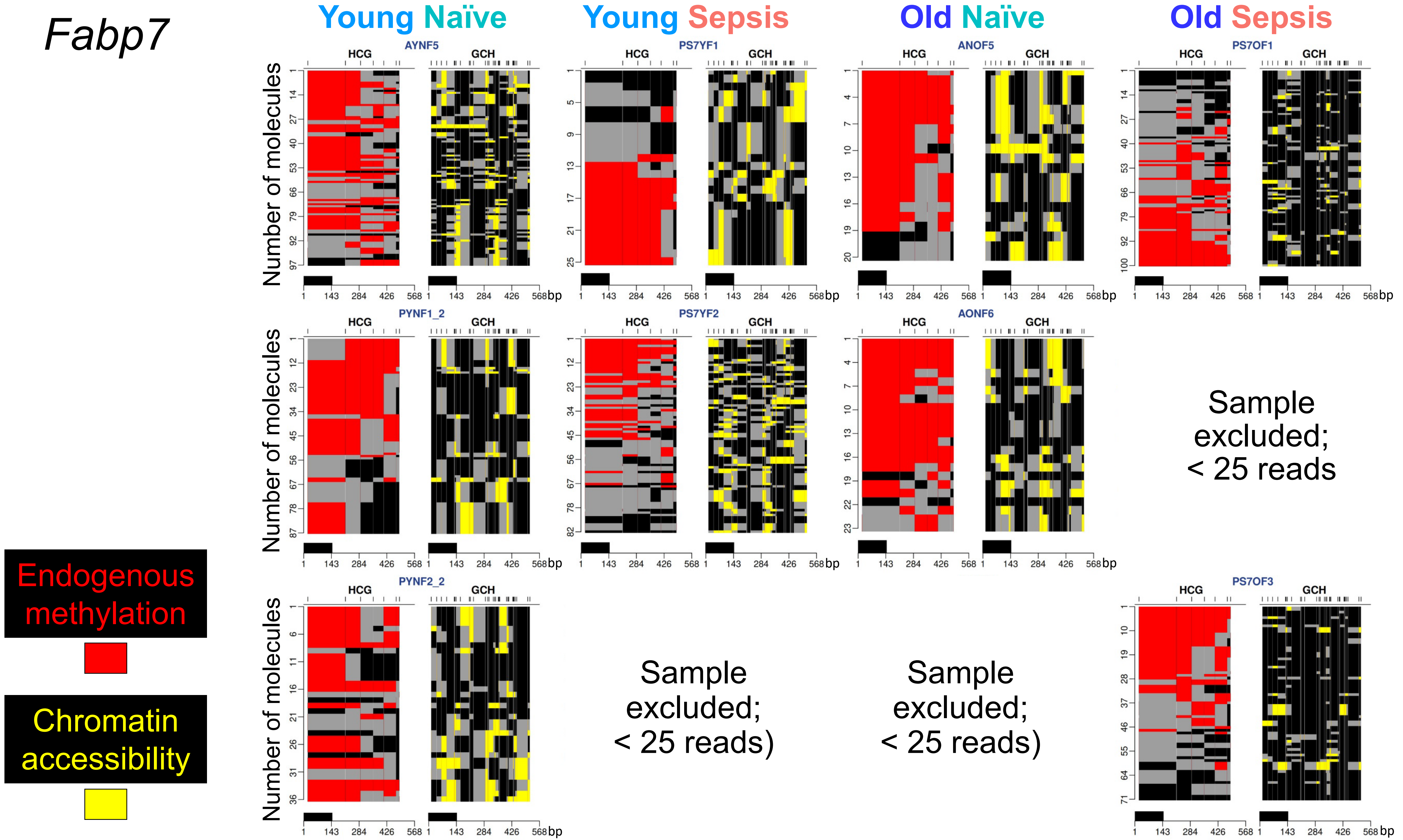
Fabp7
Young Naïve
Young Sepsis
Old Naïve
Old Sepsis
AYNF5
PS7YF1
ANOF5
PS7OF1
PYNF1_2
PS7YF2
AONF6
PYNF2_2
PS7OF3
HCG
GCH
Number of molecules
1 143 284 426 568 bp
Sample excluded; < 25 reads
Sample excluded; < 25 reads)
Sample excluded; < 25 reads)
Endogenous methylation
Chromatin accessibility

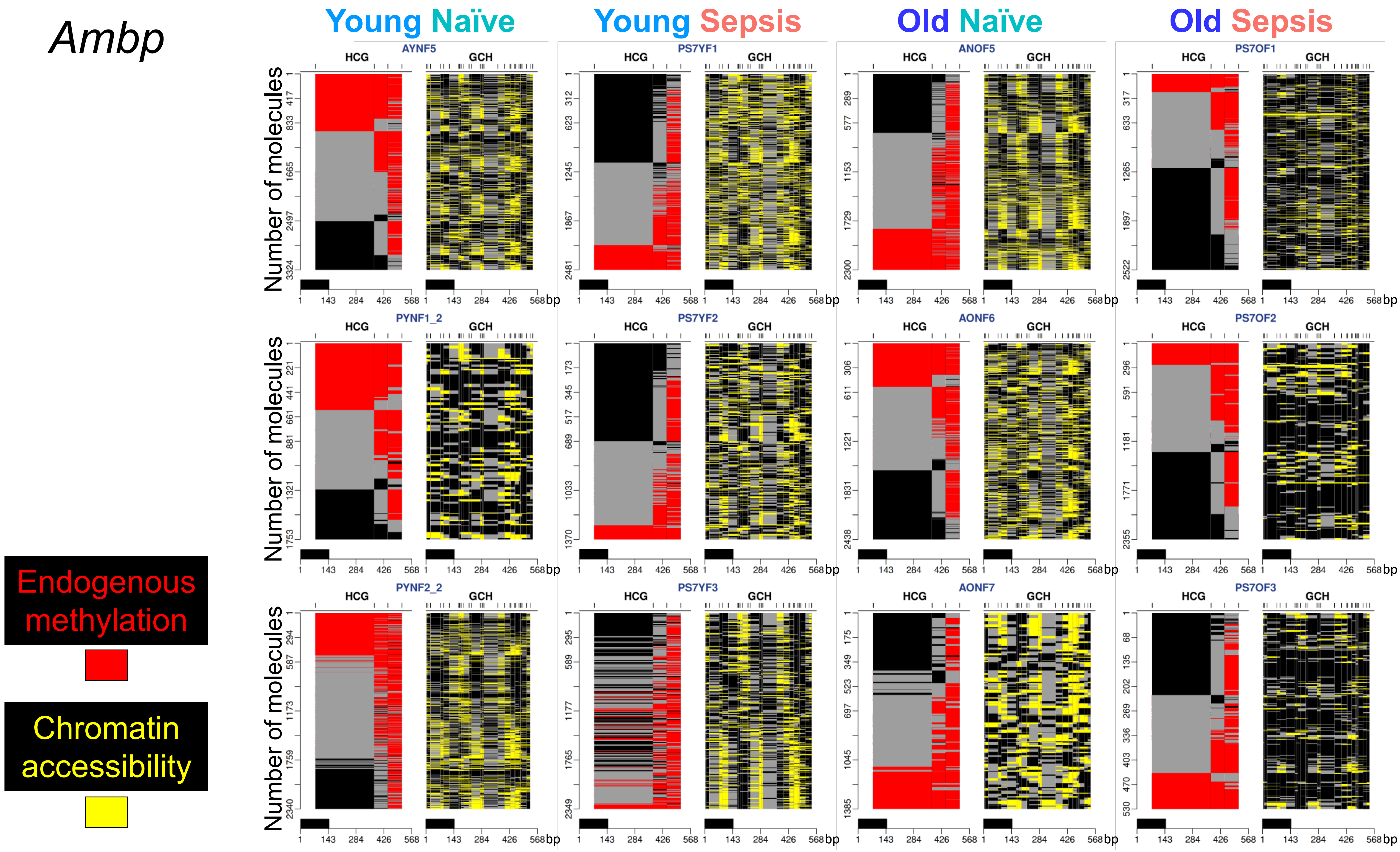

Ambp
Young Naïve
Young Sepsis
Old Naïve
Old Sepsis
AYNF5
PYNF1_2
PYNF2_2
PS7YF1
PS7YF2
PS7YF3
ANOF5
AONF6
AONF7
PS7OF1
PS7OF2
PS7OF3
HCG
GCH
Number of molecules
1
143
284
426
568
bp
Endogenous methylation
Chromatin accessibility

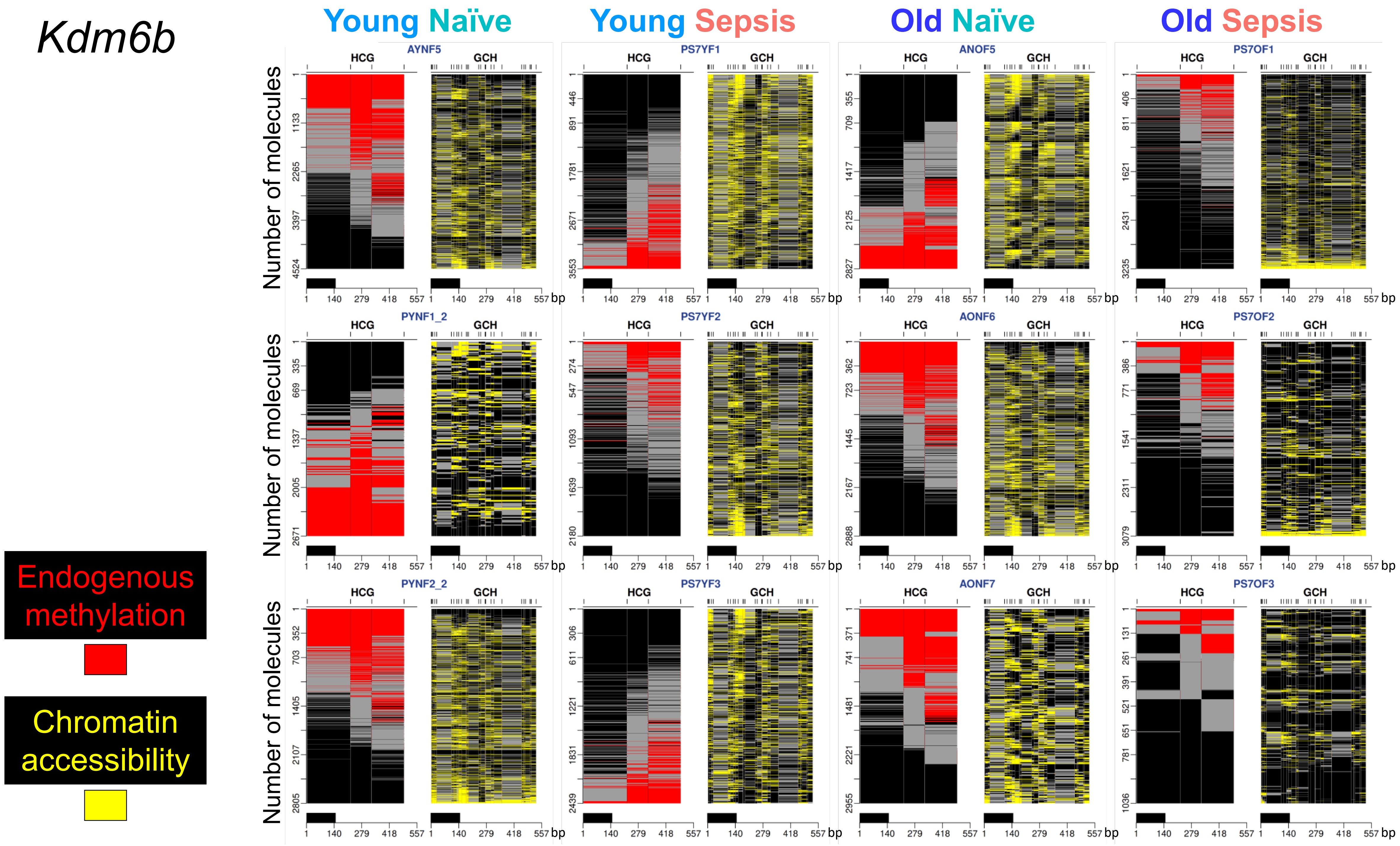

Kdm6b
Young Naïve
Young Sepsis
Old Naïve
Old Sepsis
Number of molecules
HCG
GCH
AYNF5
PYNF1_2
PYNF2_2
PS7YF1
PS7YF2
PS7YF3
ANOF5
AONF6
AONF7
PS7OF1
PS7OF2
PS7OF3
bp
Endogenous methylation
Chromatin accessibility

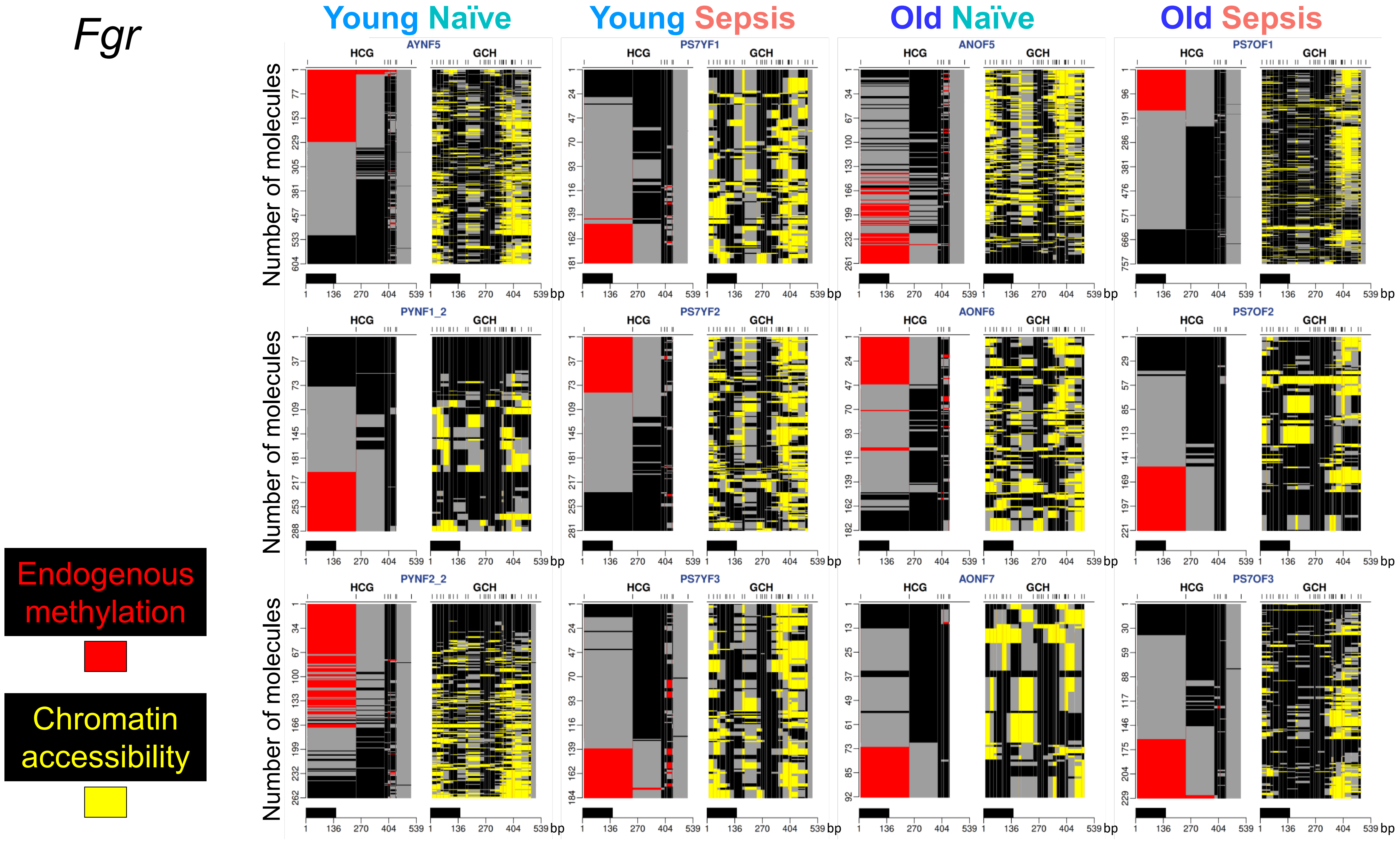

Fgr
Young Naïve
Young Sepsis
Old Naïve
Old Sepsis
AYNF5
PYNF1_2
PYNF2_2
PS7YF1
PS7YF2
PS7YF3
ANOF5
ANOF6
ANOF7
PS7OF1
PS7OF2
PS7OF3
HCG
GCH
Number of molecules
1 136 270 404 539 bp
Endogenous methylation
Chromatin accessibility

Gpnmb

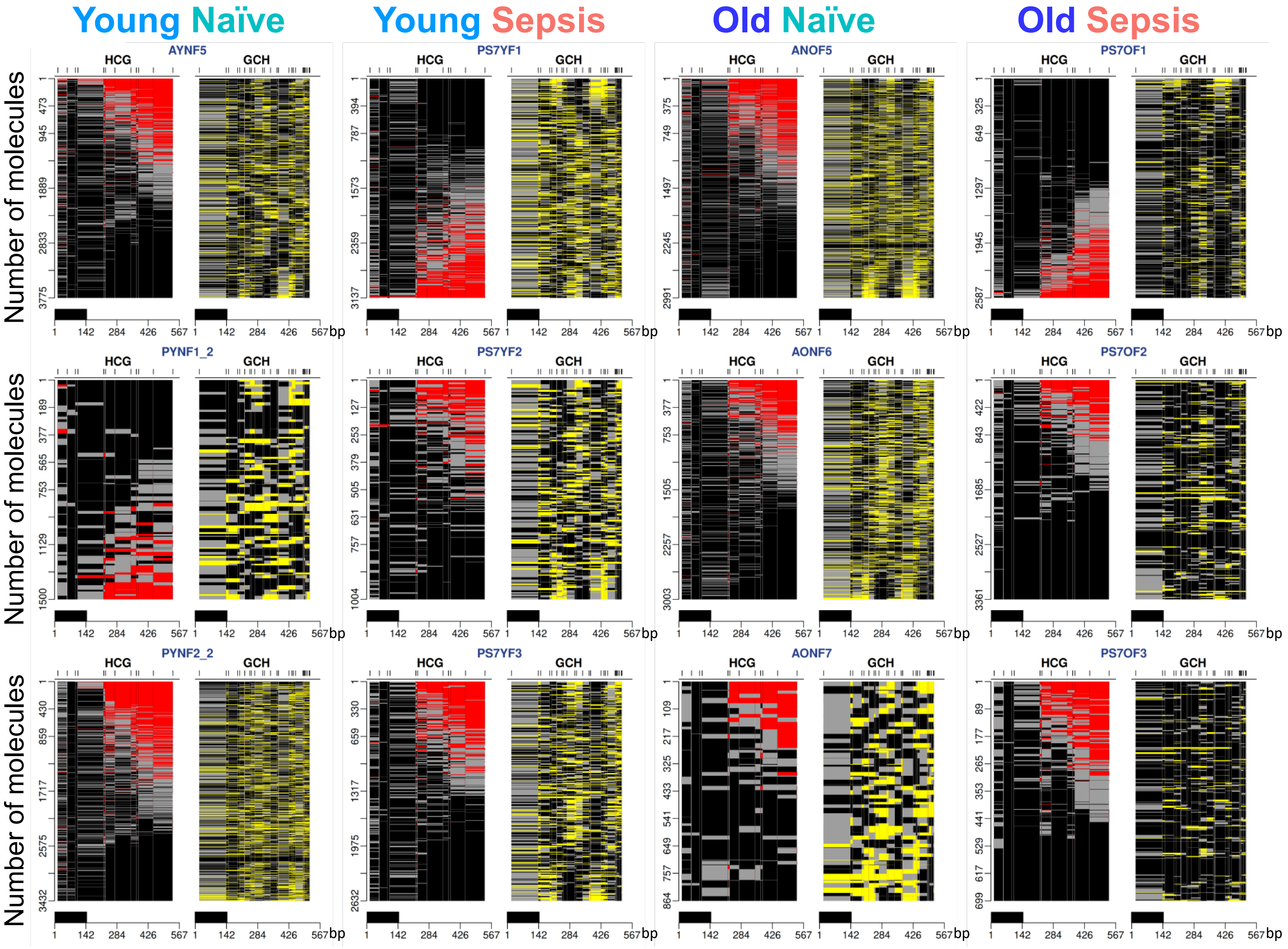

Endogenous methylation

Chromatin accessibility

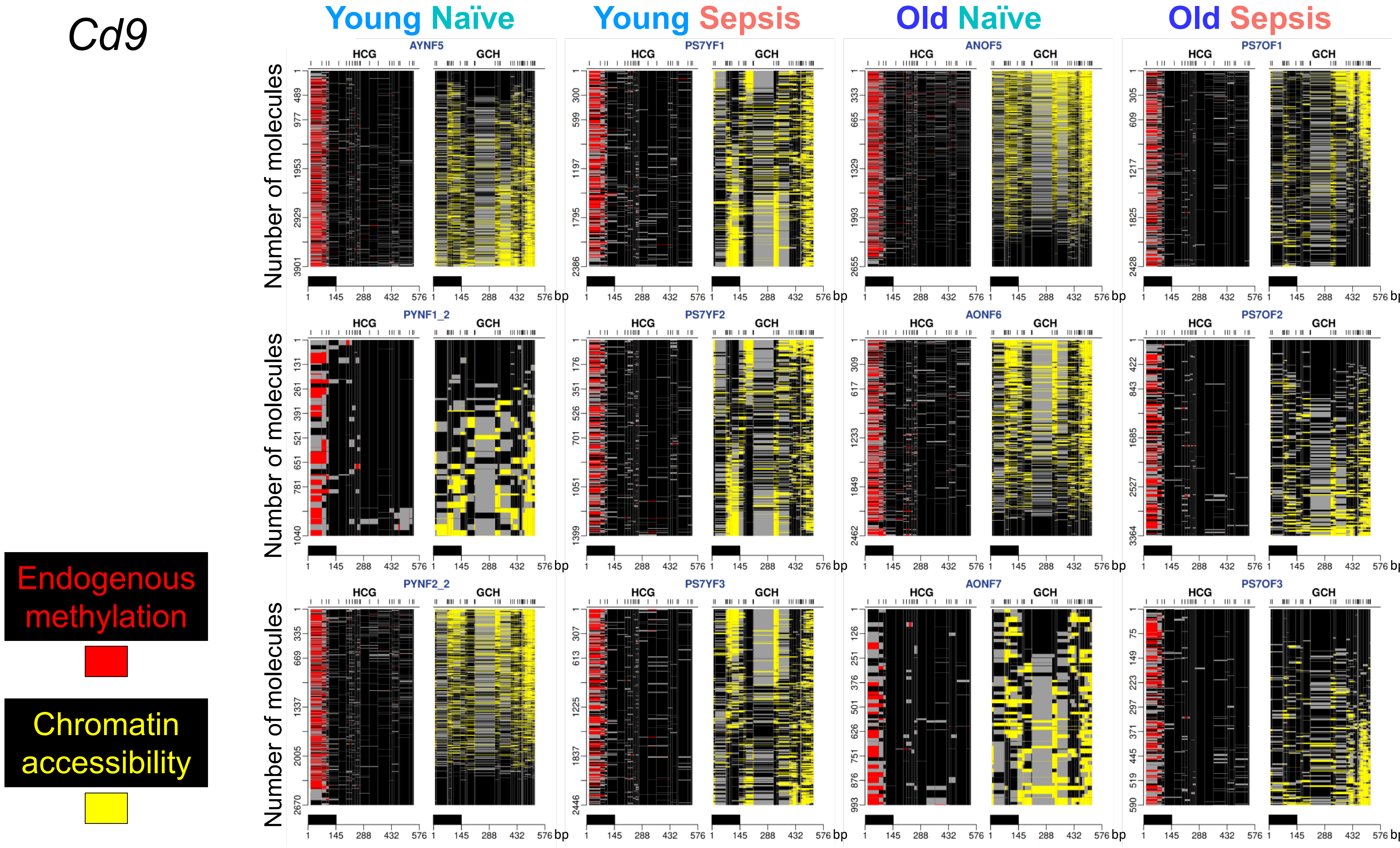
Cd9
Young Naïve
Young Sepsis
Old Naïve
Old Sepsis
AYNF5
PYNF1_2
PYNF2_2
PS7YF1
PS7YF2
PS7YF3
ANOF5
AONF6
AONF7
PS7OF1
PS7OF2
PS7OF3
HCG
GCH
Number of molecules
bp
Endogenous methylation
Chromatin accessibility

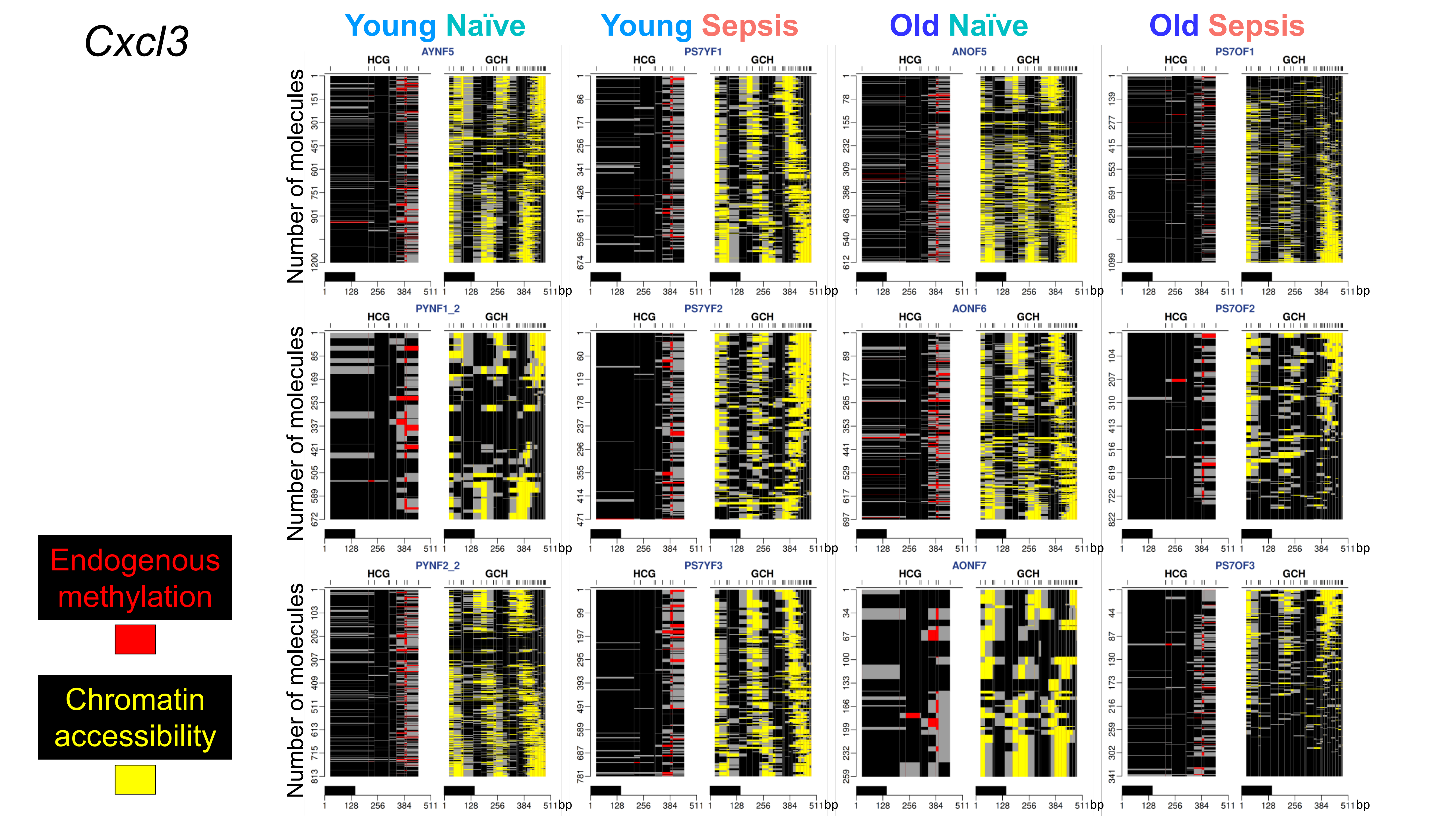

Cxcl3
Young Naïve
Young Sepsis
Old Naïve
Old Sepsis
AYNF5
PYNF1_2
PYNF2_2
PS7YF1
PS7YF2
PS7YF3
ANOF5
AONF6
AONF7
PS7OF1
PS7OF2
PS7OF3
HCG
GCH
Number of molecules
bp
Endogenous methylation
Chromatin accessibility

Mmp9

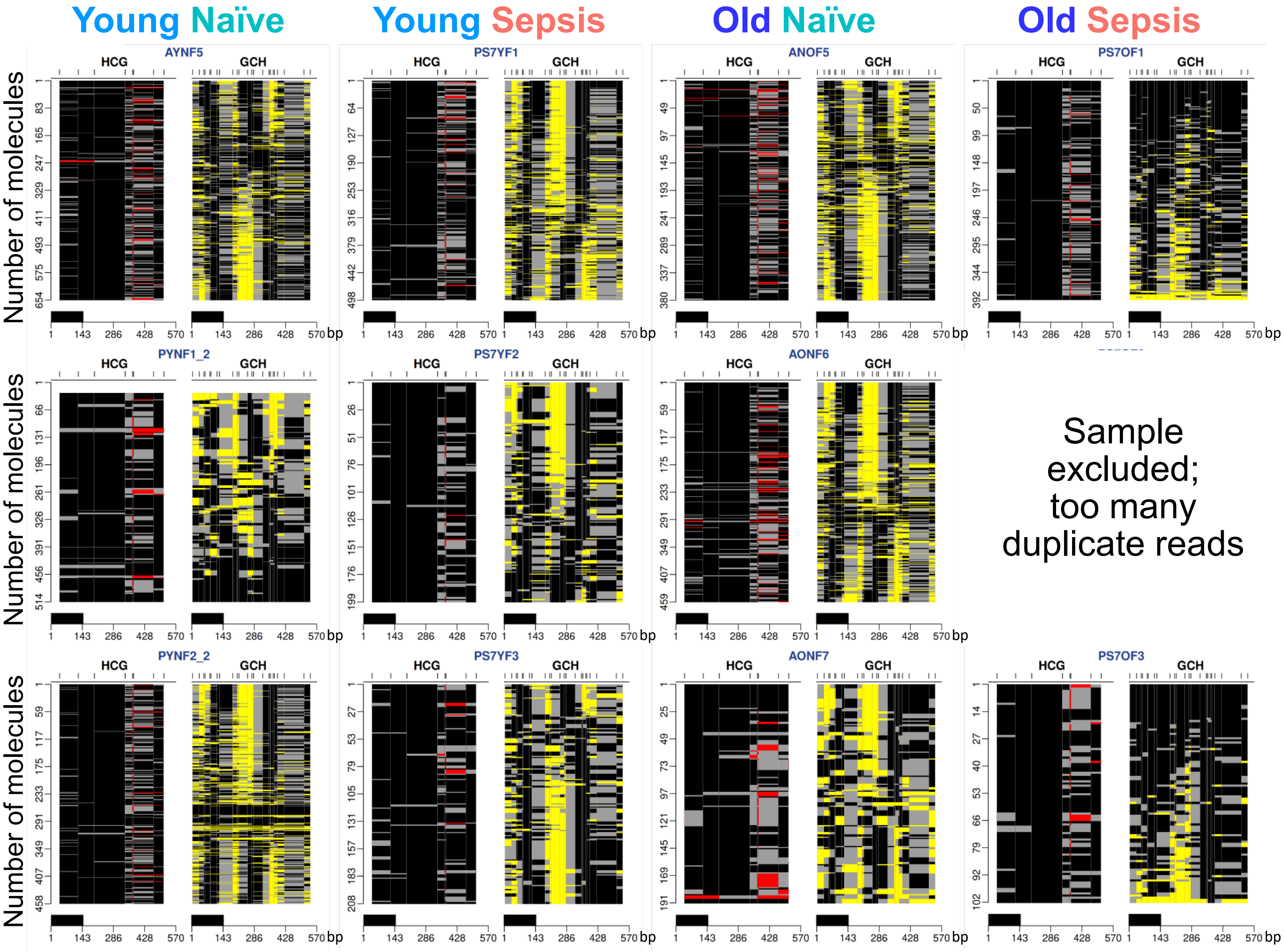

Young Naïve
Young Sepsis
Old Naïve
Old Sepsis
AYNF5
PS7YF1
ANOF5
PS7OF1
PYNF1_2
PS7YF2
AONF6
PYNF2_2
PS7YF3
AONF7
PS7OF3
HCG
GCH
Number of molecules
1 143 286 428 570 bp
Sample excluded; too many duplicate reads

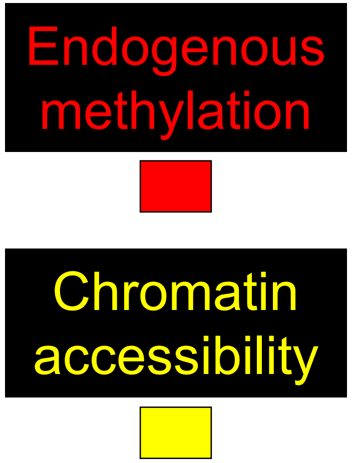

Endogenous methylation
Chromatin accessibility

Class 7 promoters: *Vcan*, *Tmem176*, *Stab1*, *Plac8*, *Il10*, *Gprc5b*, *Htr2a*, *S100a10*, *Car4*, *Mt2*, *Igkv4-69*, *Ccl17*, *Igkv12-44*, *Igkv12-46*

<u>No</u> NRF formation in response to CLP + DCS across <u>all</u> cohorts:

- <u>Low levels</u> of endogenous CpG methylation (at HCGs) or < 3 HCG sites
- Accessibility pattern consistent with disorganized or random nucleosome arrays
- Decreased accessibility in old sepsis cohort compared to other cohorts

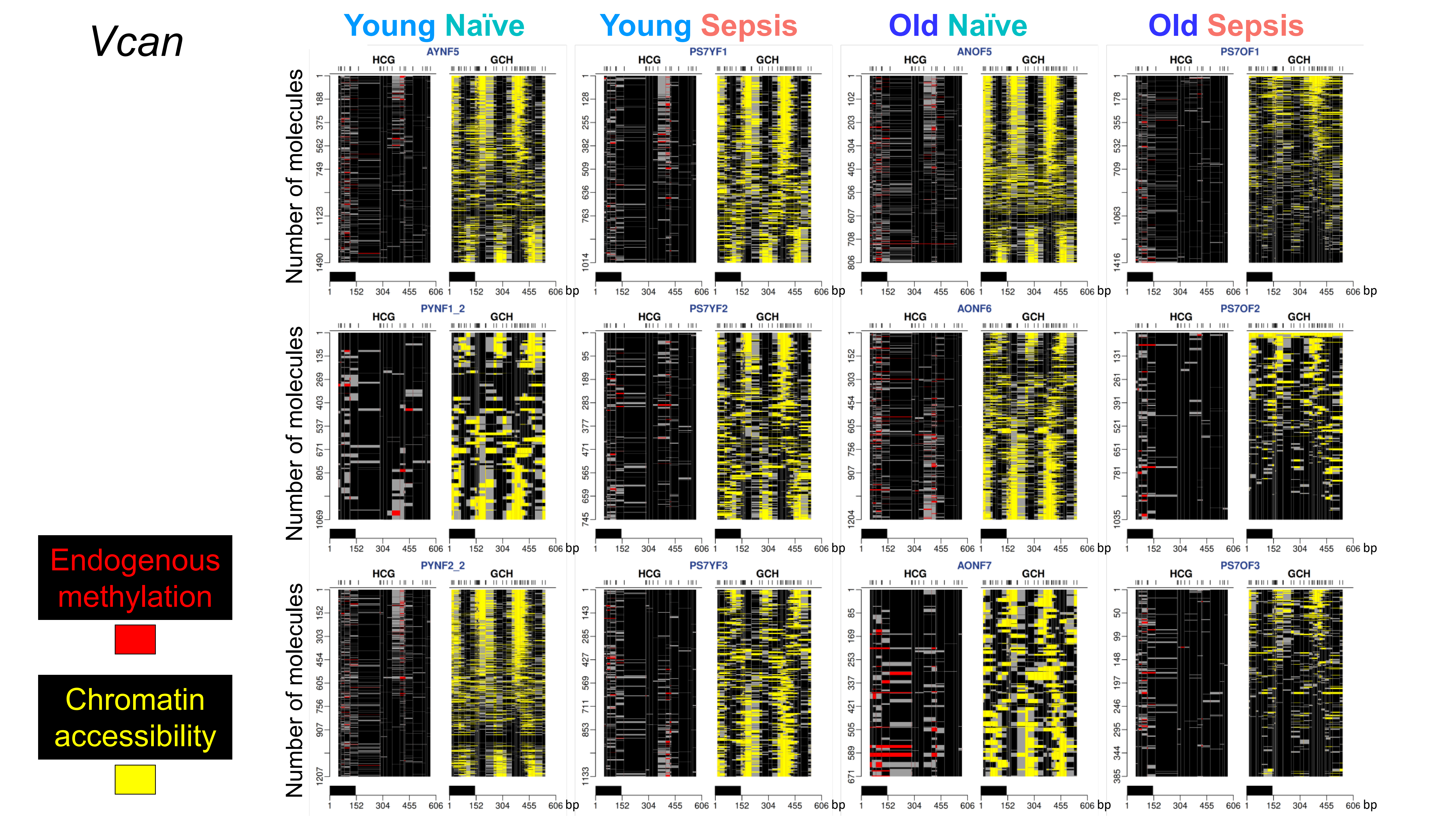
Vcan
Young Naïve
Young Sepsis
Old Naïve
Old Sepsis
AYNF5
PS7YF1
ANOF5
PS7OF1
PYNF1_2
PS7YF2
AONF6
PS7OF2
PYNF2_2
PS7YF3
AONF7
PS7OF3
HCG
GCH
Number of molecules
bp
Endogenous methylation
Chromatin accessibility

Tmem176

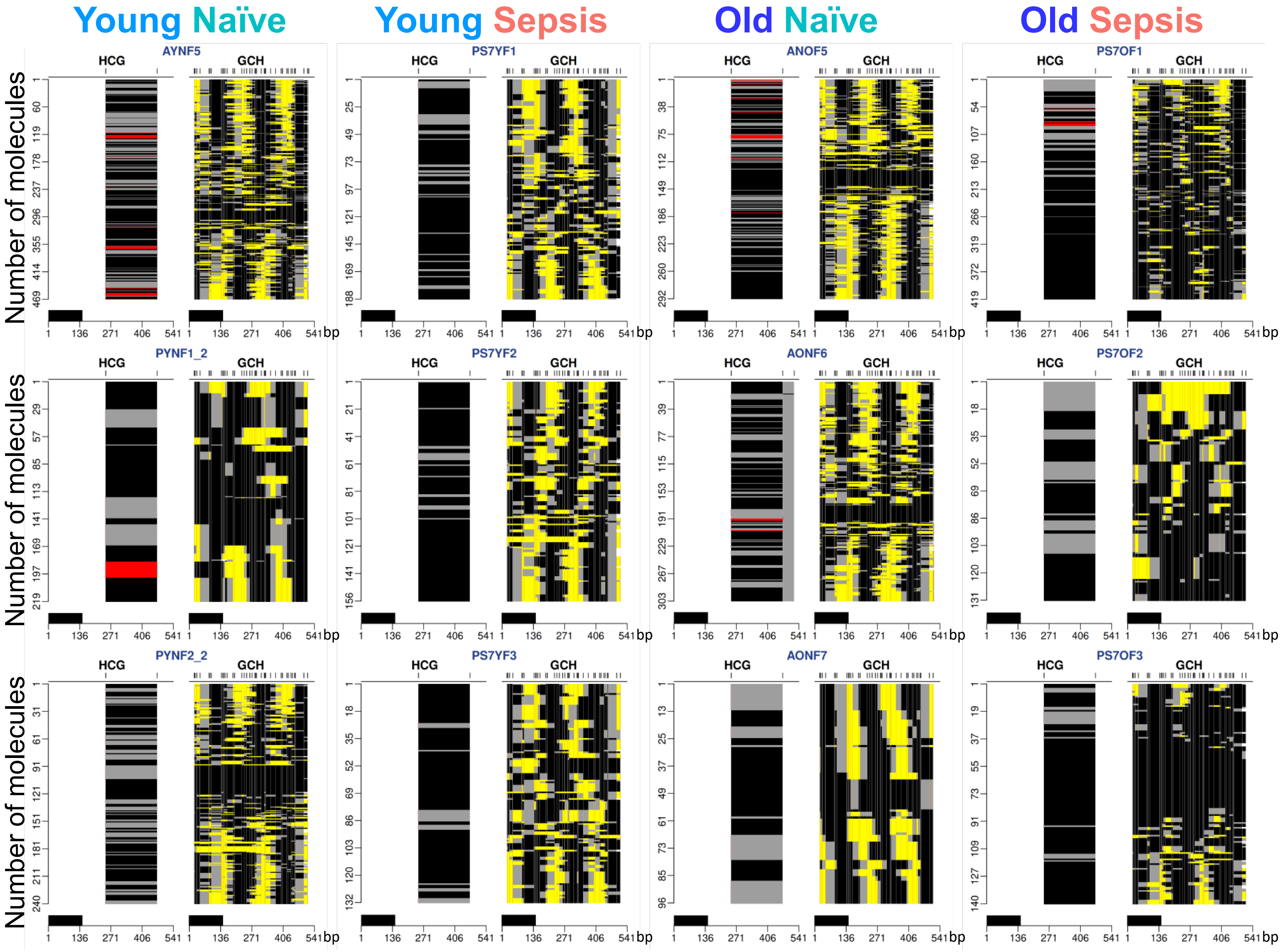
Young Naïve
Young Sepsis
Old Naïve
Old Sepsis
AYNF5
PS7YF1
ANOF5
PS7OF1
PYNF1_2
PS7YF2
AONF6
PS7OF2
PYNF2_2
PS7YF3
AONF7
PS7OF3
HCG
GCH
Number of molecules
1 136 271 406 541 bp

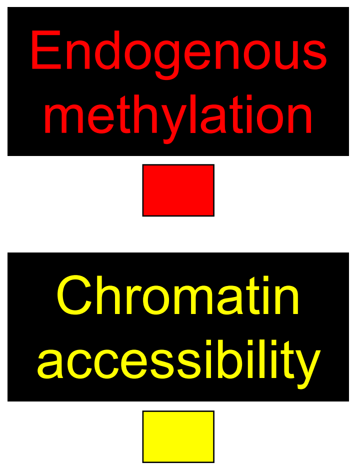
Endogenous methylation
Chromatin accessibility

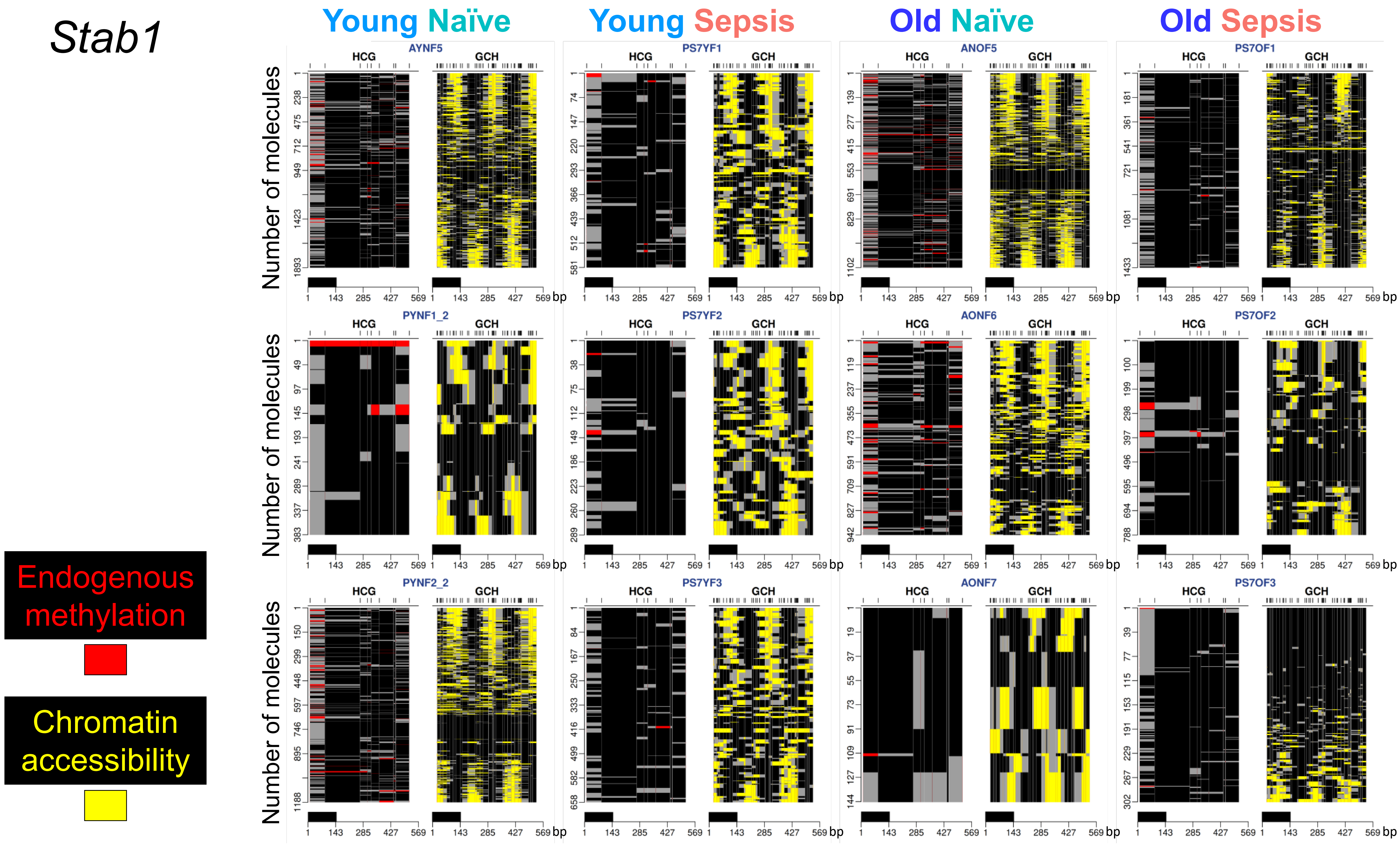

Stab1
Young Naïve
Young Sepsis
Old Naïve
Old Sepsis
AYNF5
PS7YF1
ANOF5
PS7OF1
PYNF1_2
PS7YF2
AONF6
PS7OF2
PYNF2_2
PS7YF3
AONF7
PS7OF3
HCG
GCH
Number of molecules
bp
Endogenous methylation
Chromatin accessibility

Plac8

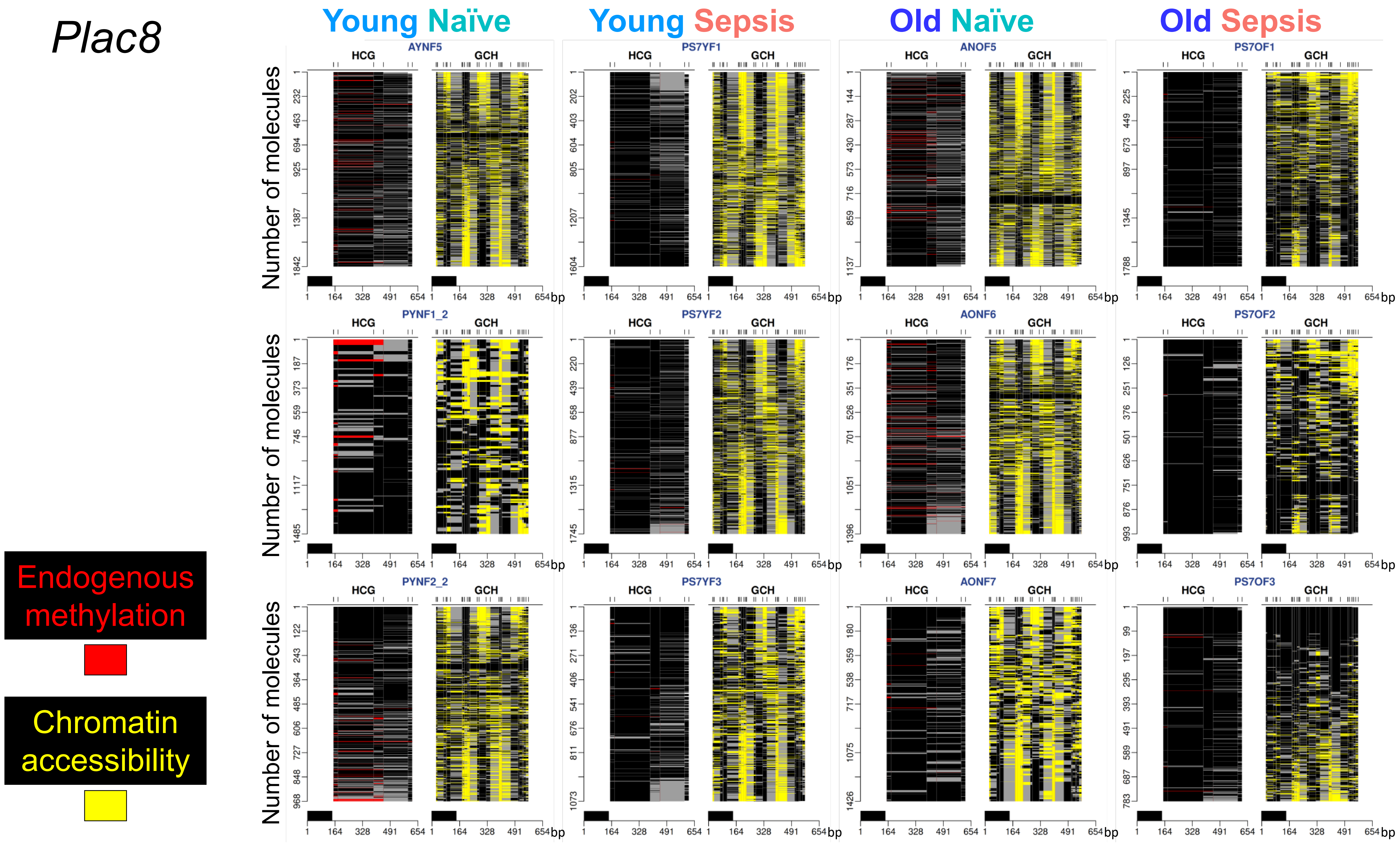
Young Naïve
Young Sepsis
Old Naïve
Old Sepsis
AYNF5
PS7YF1
ANOF5
PS7OF1
PYNF1_2
PS7YF2
ANOF6
PS7OF2
PYNF2_2
PS7YF3
AONF7
PS7OF3
HCG
GCH
Number of molecules
bp
Endogenous methylation
Chromatin accessibility

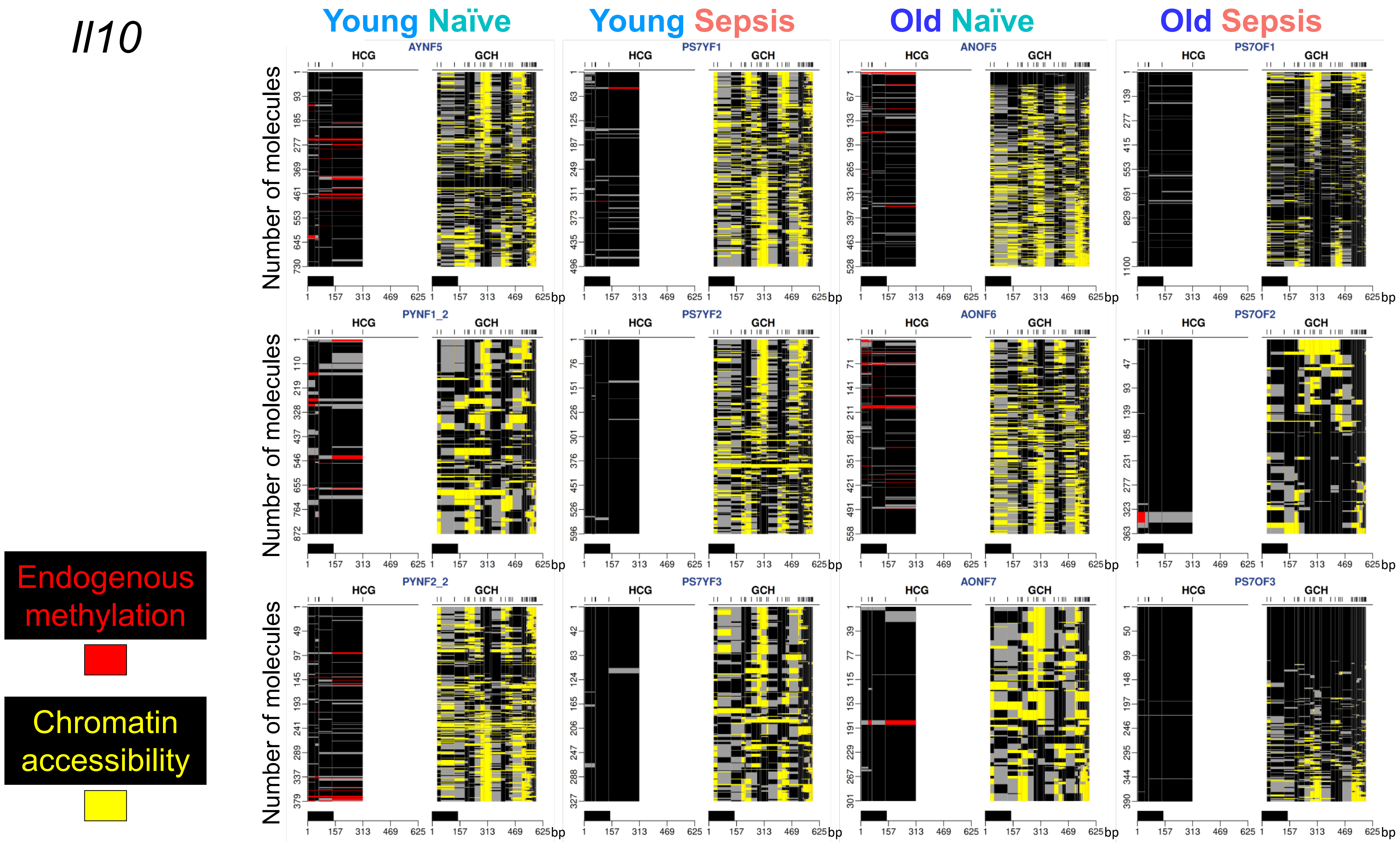
Il10
Young Naïve
Young Sepsis
Old Naïve
Old Sepsis
AYNF5
PYNF1_2
PYNF2_2
PS7YF1
PS7YF2
PS7YF3
ANOF5
AONF6
AONF7
PS7OF1
PS7OF2
PS7OF3
HCG
GCH
Number of molecules
1 157 313 469 625 bp
Endogenous methylation
Chromatin accessibility

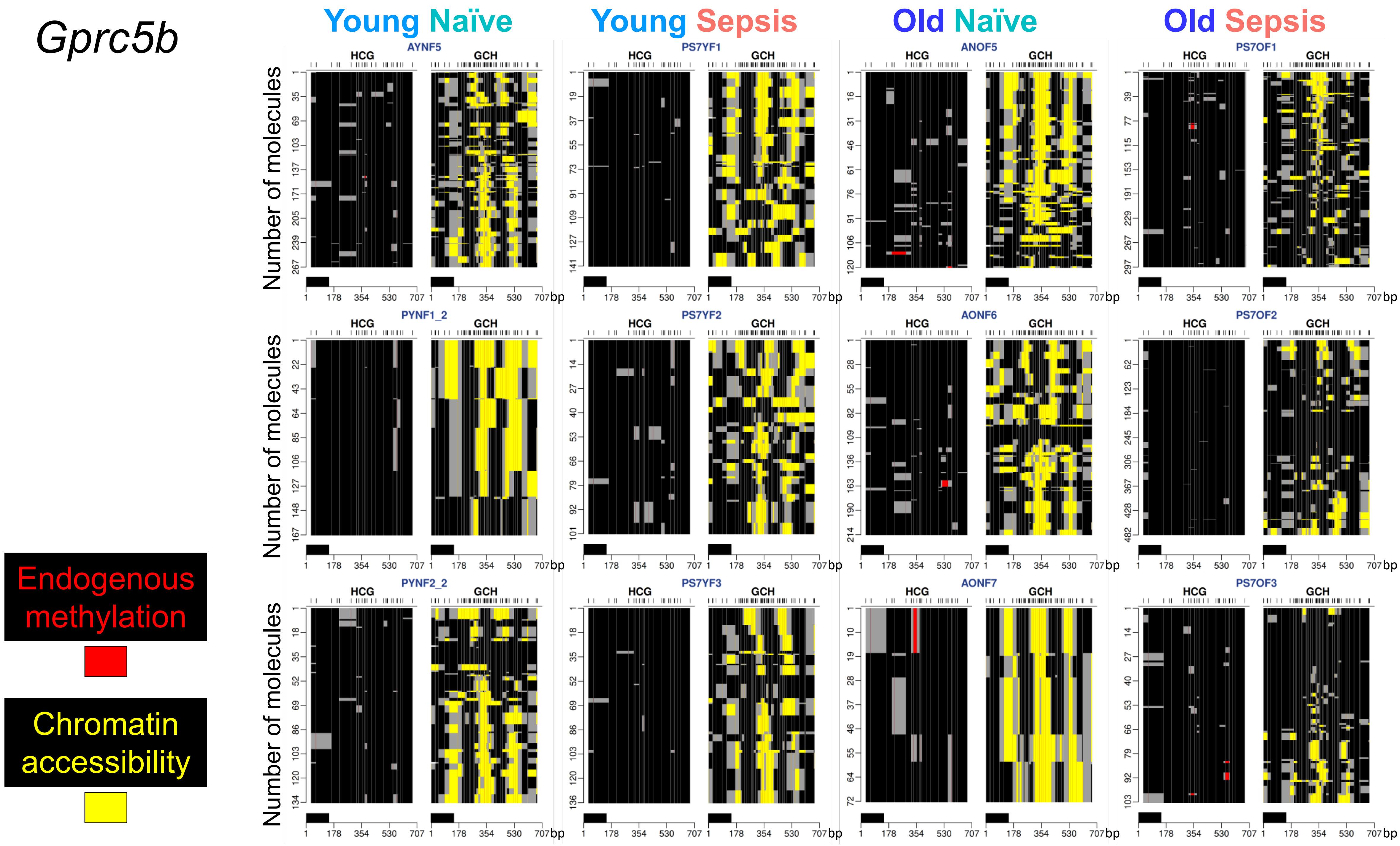

Gprc5b
Young Naïve
Young Sepsis
Old Naïve
Old Sepsis
AYNF5
PS7YF1
ANOF5
PS7OF1
PYNF1_2
PS7YF2
AONF6
PS7OF2
PYNF2_2
PS7YF3
AONF7
PS7OF3
HCG
GCH
Number of molecules
bp
Endogenous methylation
Chromatin accessibility

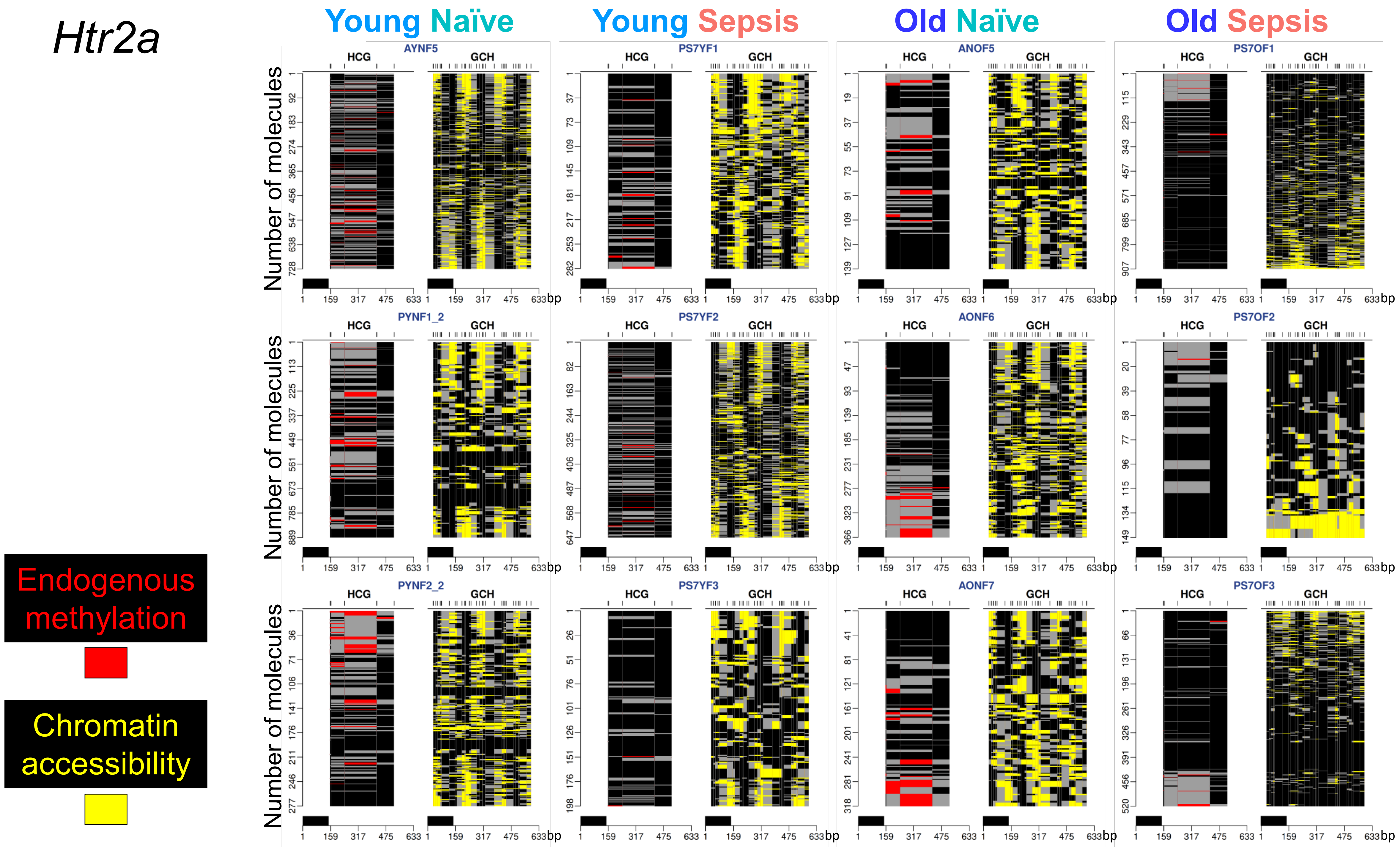

Htr2a
Young Naïve
Young Sepsis
Old Naïve
Old Sepsis
AYNF5
PS7YF1
ANOF5
PS7OF1
PYNF1_2
PS7YF2
AONF6
PS7OF2
PYNF2_2
PS7YF3
AONF7
PS7OF3
HCG
GCH
Number of molecules
1 159 317 475 633 bp
Endogenous methylation
Chromatin accessibility

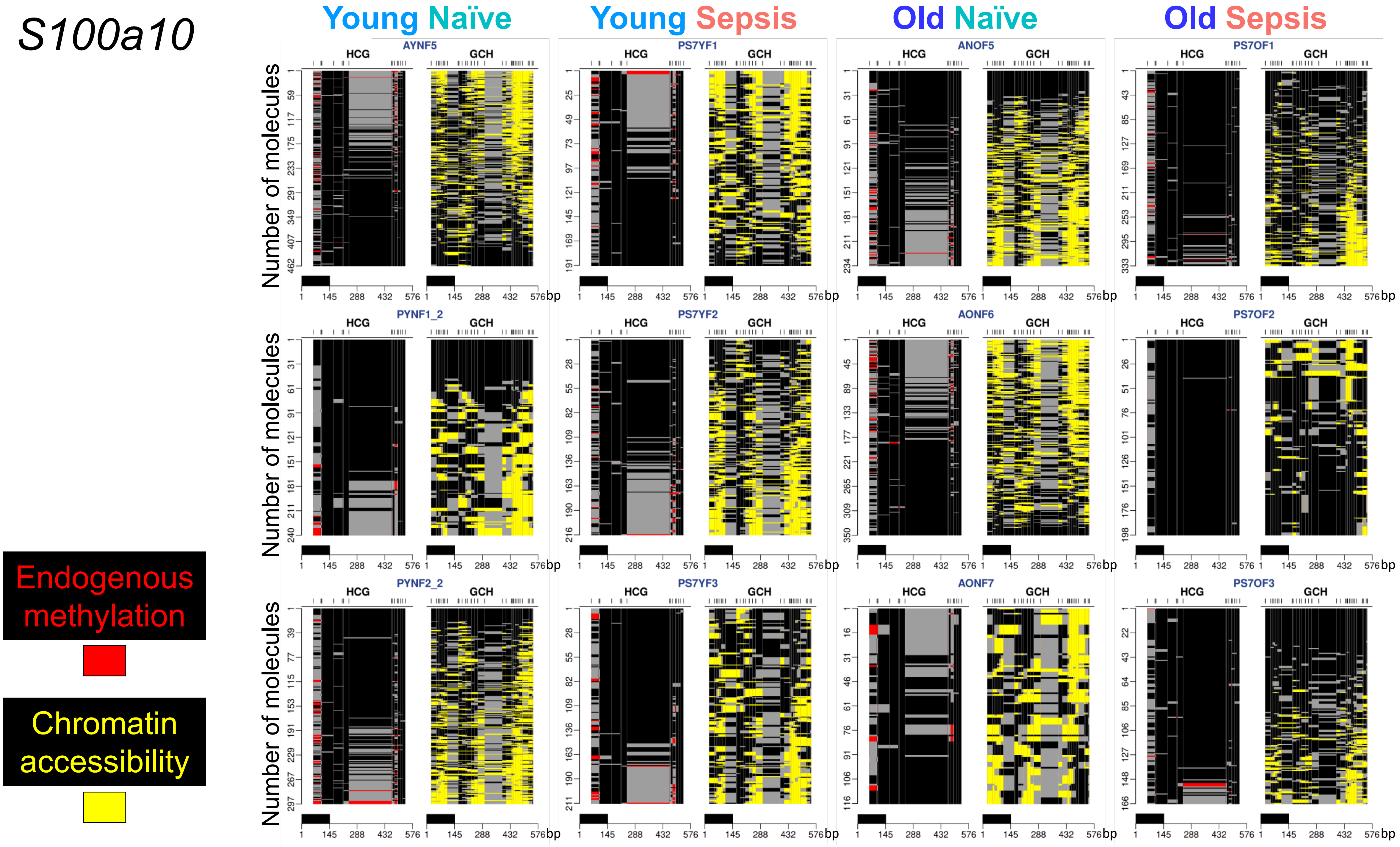
S100a10
Young Naïve
Young Sepsis
Old Naïve
Old Sepsis
AYNF5
PS7YF1
ANOF5
PS7OF1
PYNF1_2
PS7YF2
AONF6
PS7OF2
PYNF2_2
PS7YF3
AONF7
PS7OF3
HCG
GCH
Number of molecules
1 145 288 432 576 bp
Endogenous methylation
Chromatin accessibility

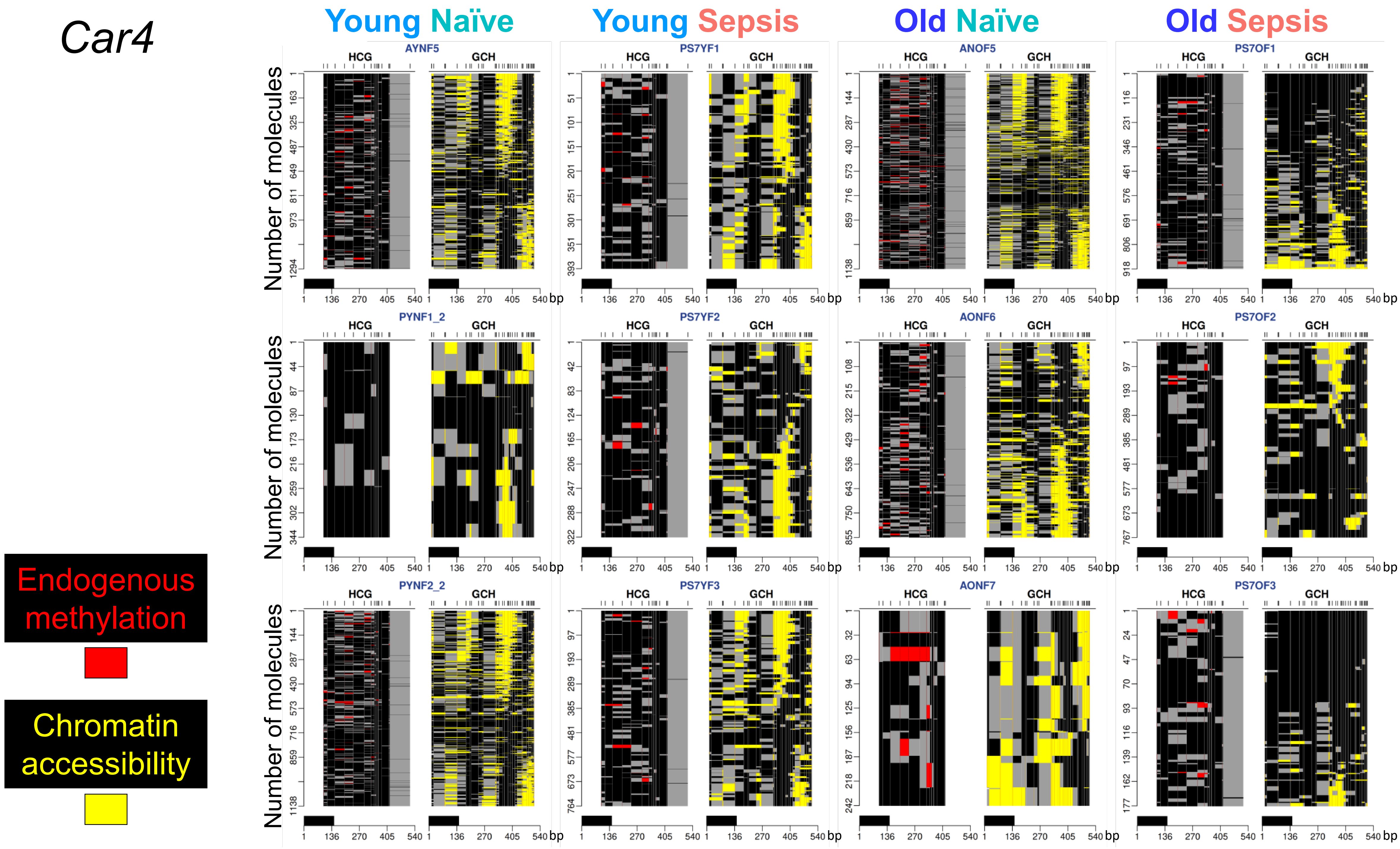

Car4
Young Naïve
Young Sepsis
Old Naïve
Old Sepsis
AYNF5
PYNF1_2
PYNF2_2
PS7YF1
PS7YF2
PS7YF3
ANOF5
AONF6
AONF7
PS7OF1
PS7OF2
PS7OF3
HCG
GCH
Number of molecules
1 136 270 405 540 bp
Endogenous methylation
Chromatin accessibility

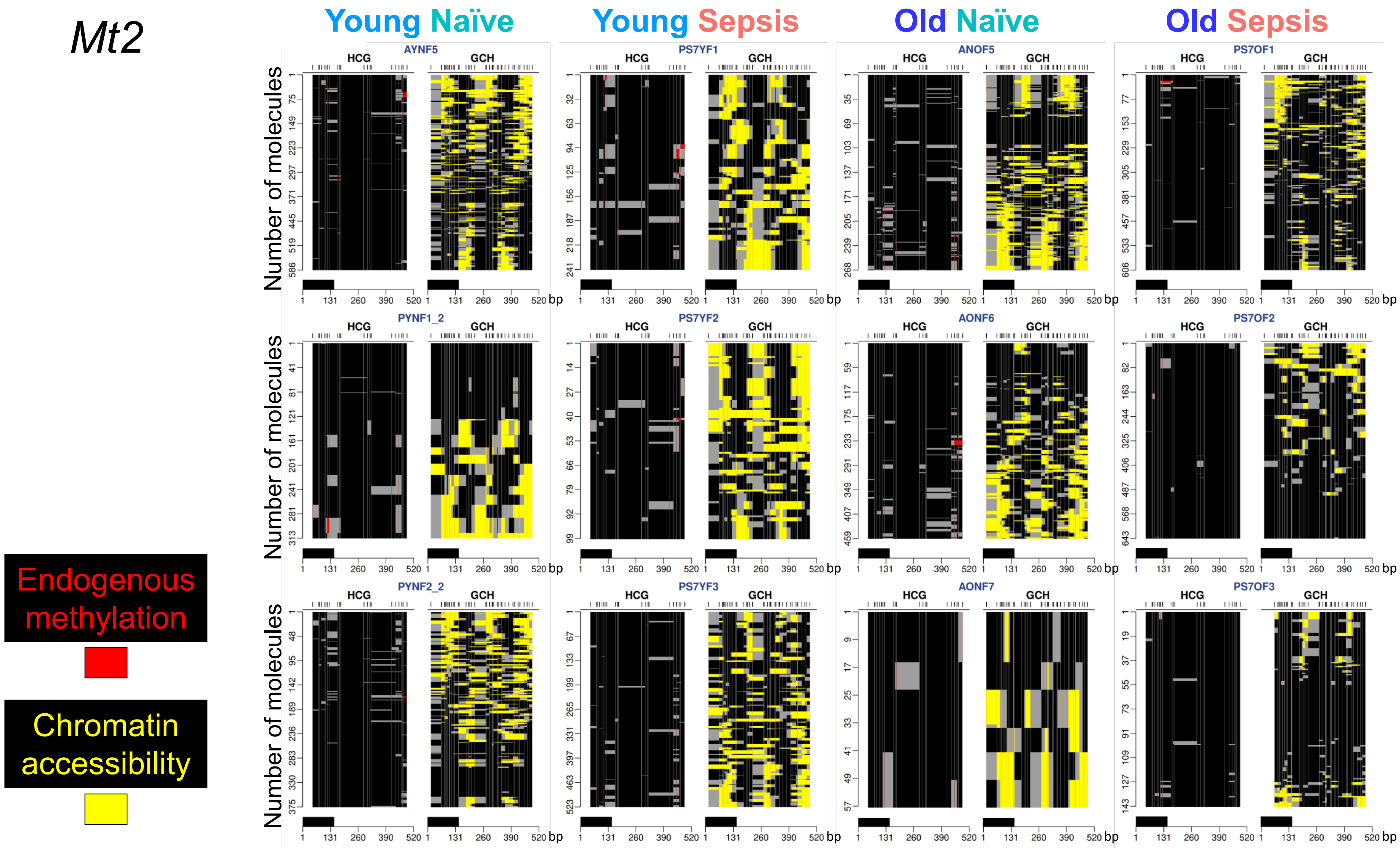
Mt2
Young Naïve
Young Sepsis
Old Naïve
Old Sepsis
AYNF5
PYNF1_2
PYNF2_2
PS7YF1
PS7YF2
PS7YF3
ANOF5
AONF6
AONF7
PS7OF1
PS7OF2
PS7OF3
HCG
GCH
Number of molecules
bp
Endogenous methylation
Chromatin accessibility

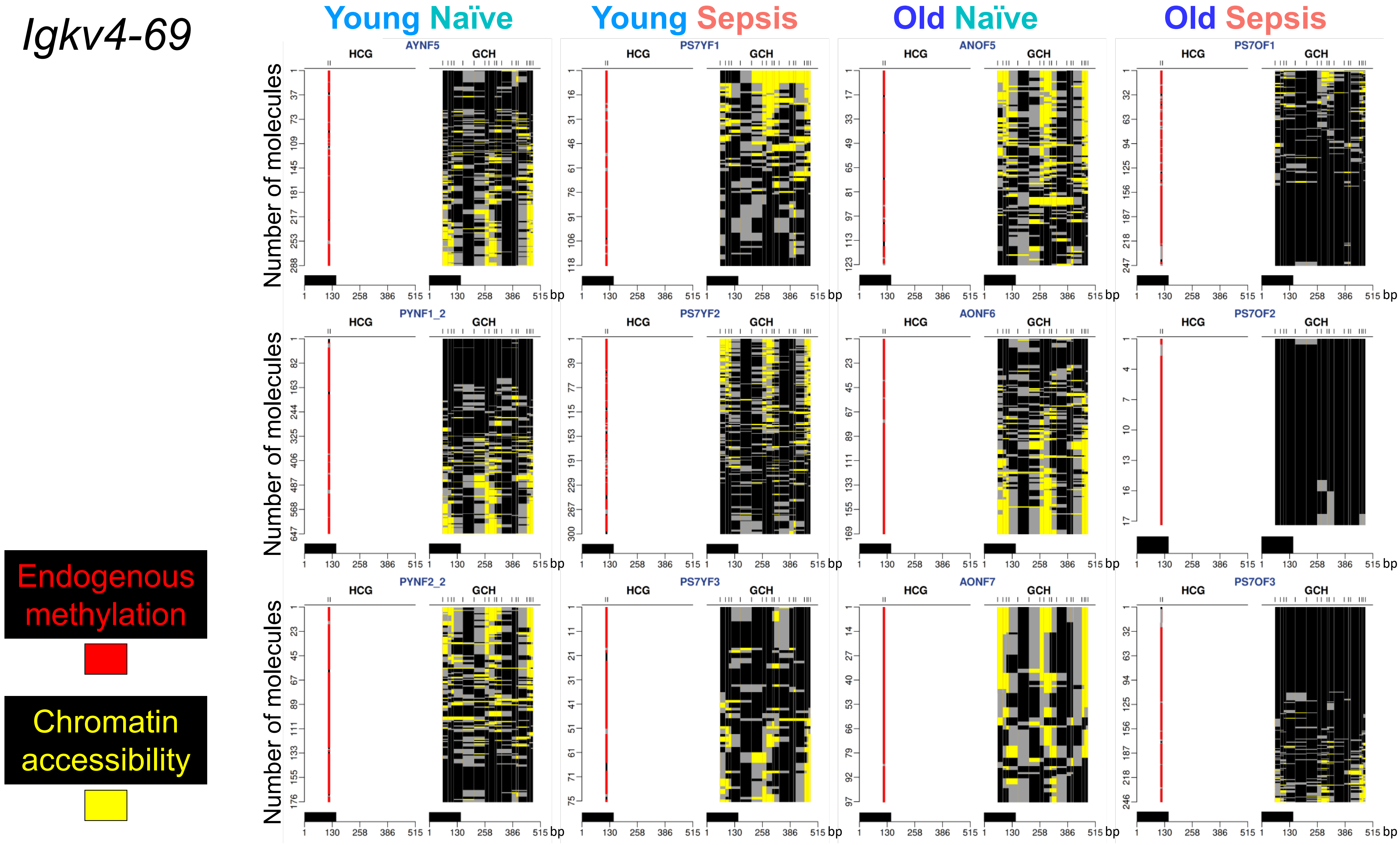
Igkv4-69
Young Naïve
Young Sepsis
Old Naïve
Old Sepsis
AYNF5
PYNF1_2
PYNF2_2
PS7YF1
PS7YF2
PS7YF3
ANOF5
AONF6
AONF7
PS7OF1
PS7OF2
PS7OF3
HCG
GCH
Number of molecules
1 130 258 386 515 bp
Endogenous methylation
Chromatin accessibility

Ccl17

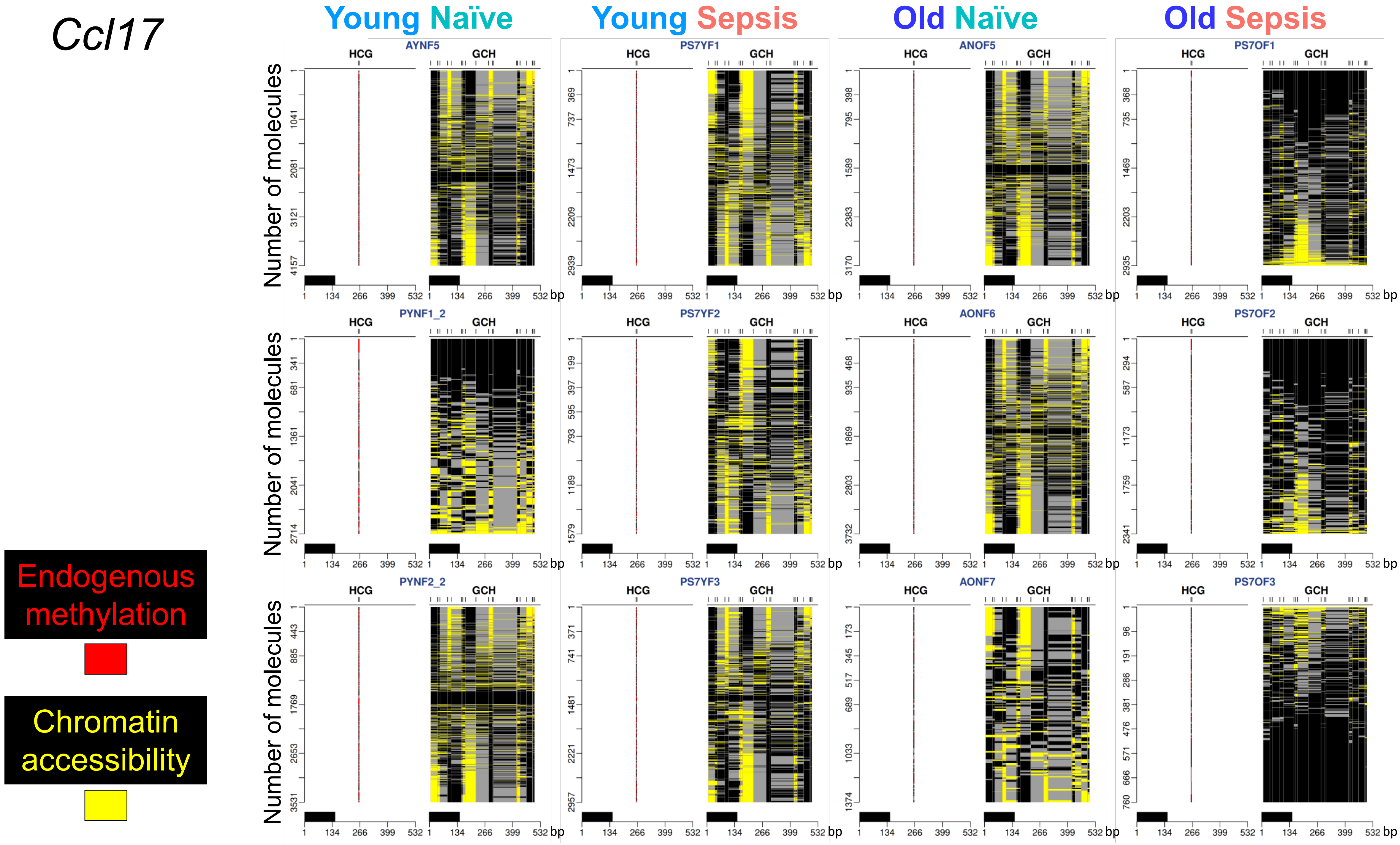
Young Naïve
Young Sepsis
Old Naïve
Old Sepsis
AYNF5
PS7YF1
ANOF5
PS7OF1
PYNF1_2
PS7YF2
AONF6
PS7OF2
PYNF2_2
PS7YF3
AONF7
PS7OF3
HCG
GCH
Number of molecules
1 134 266 399 532 bp
Endogenous methylation
Chromatin accessibility

Igkv12-44

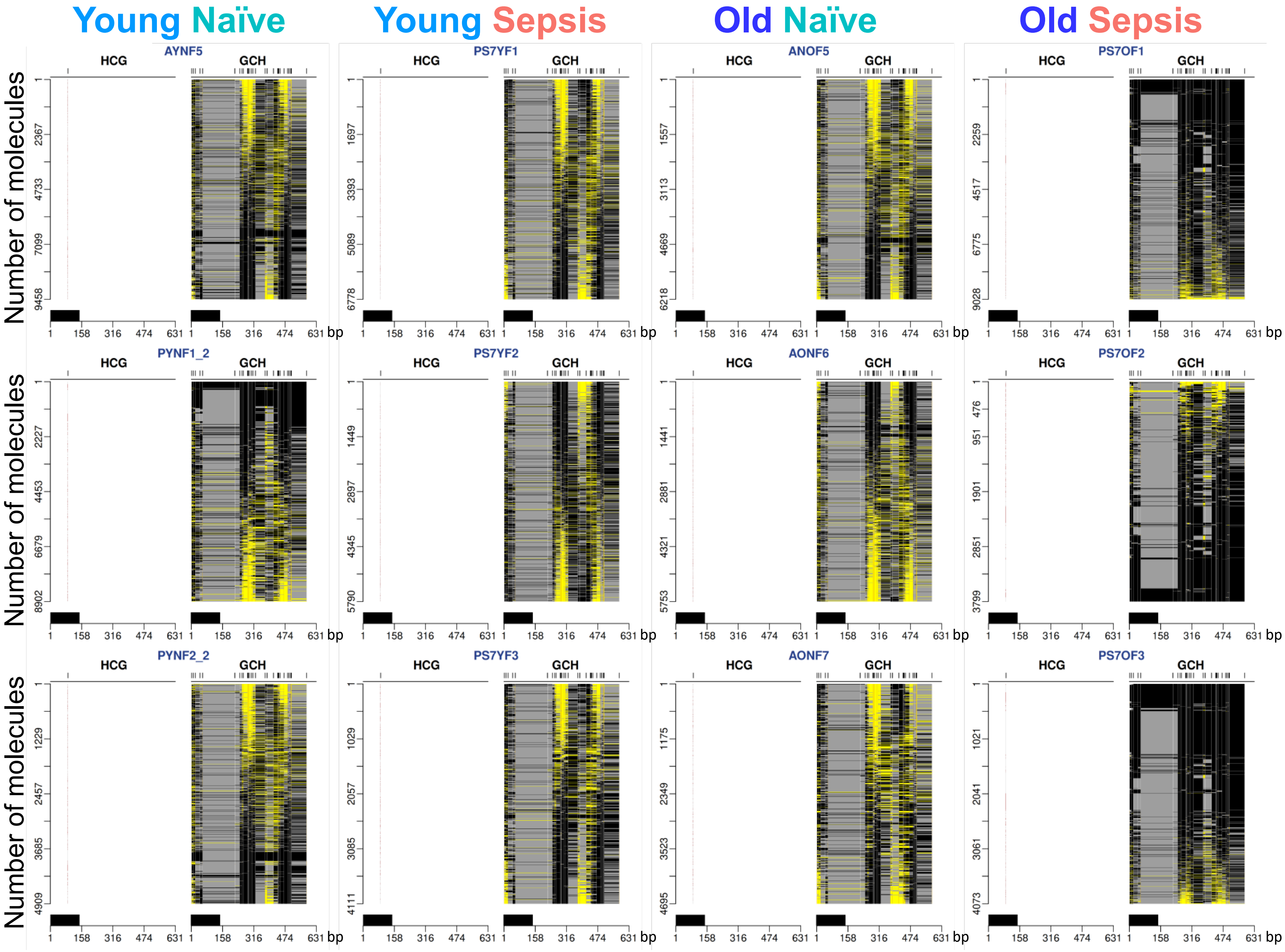

Endogenous methylation

Chromatin accessibility

Class 8 promoters: *Cd274*, *Nfkbiz*, *Hdac3*, *Vdr*, *Il4ra*, *Atf6*, *Il1rl2*

Constitutive NFRs that largely remain refractory to sepsis:

- Decrease in accessibility in old sepsis compared to all other cohorts
- Low, baseline levels of CpG methylation (any observed HCG methylation is likely attributable to M.CviPI methylating CCG sites in highly accessible regions)

Cd274

NFR-containing promoter copies

Most HCG methylation likely arises from M.CviPI modification of accessible CCG sites

Endogenous methylation

Chromatin accessibility

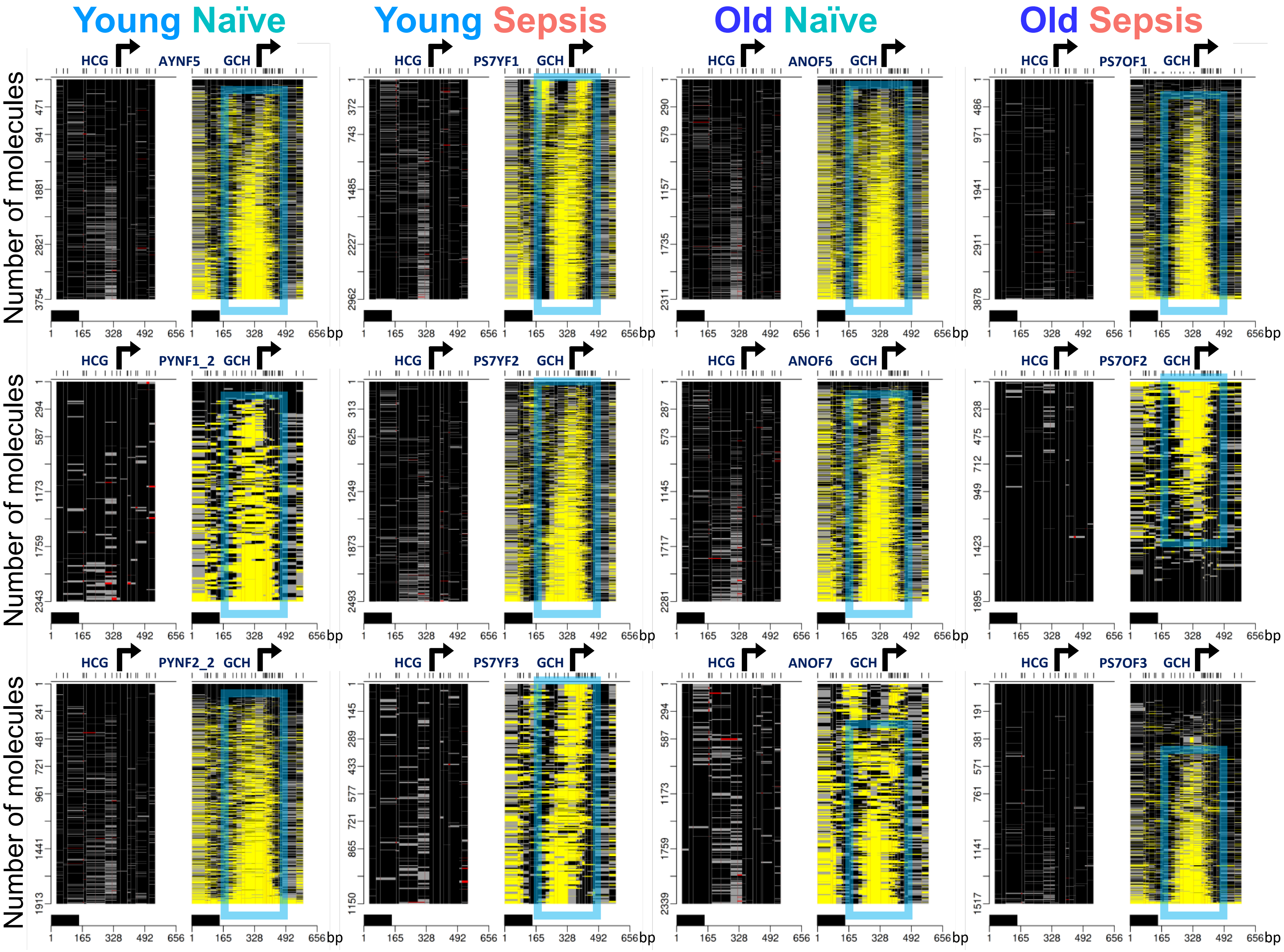

Nfkbiz

NFR-containing promoter copies

Most HCG methylation likely arises from M.CviPI modification of accessible CCG sites

Endogenous methylation

Chromatin accessibility

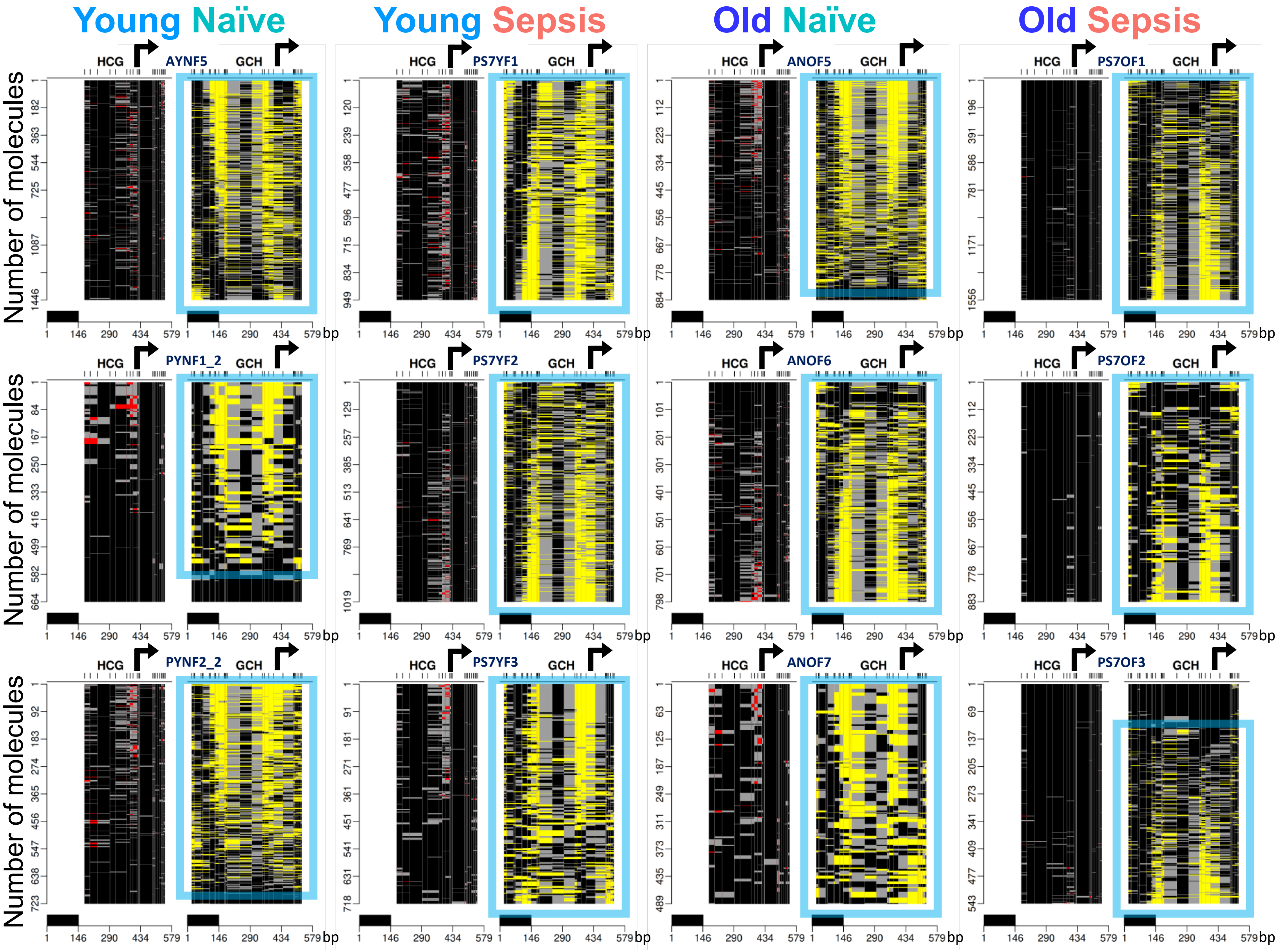

Hdac3

NFR-containing promoter copies

Most HCG methylation likely arises from M.CviPI modification of accessible CCG sites

Endogenous methylation

Chromatin accessibility

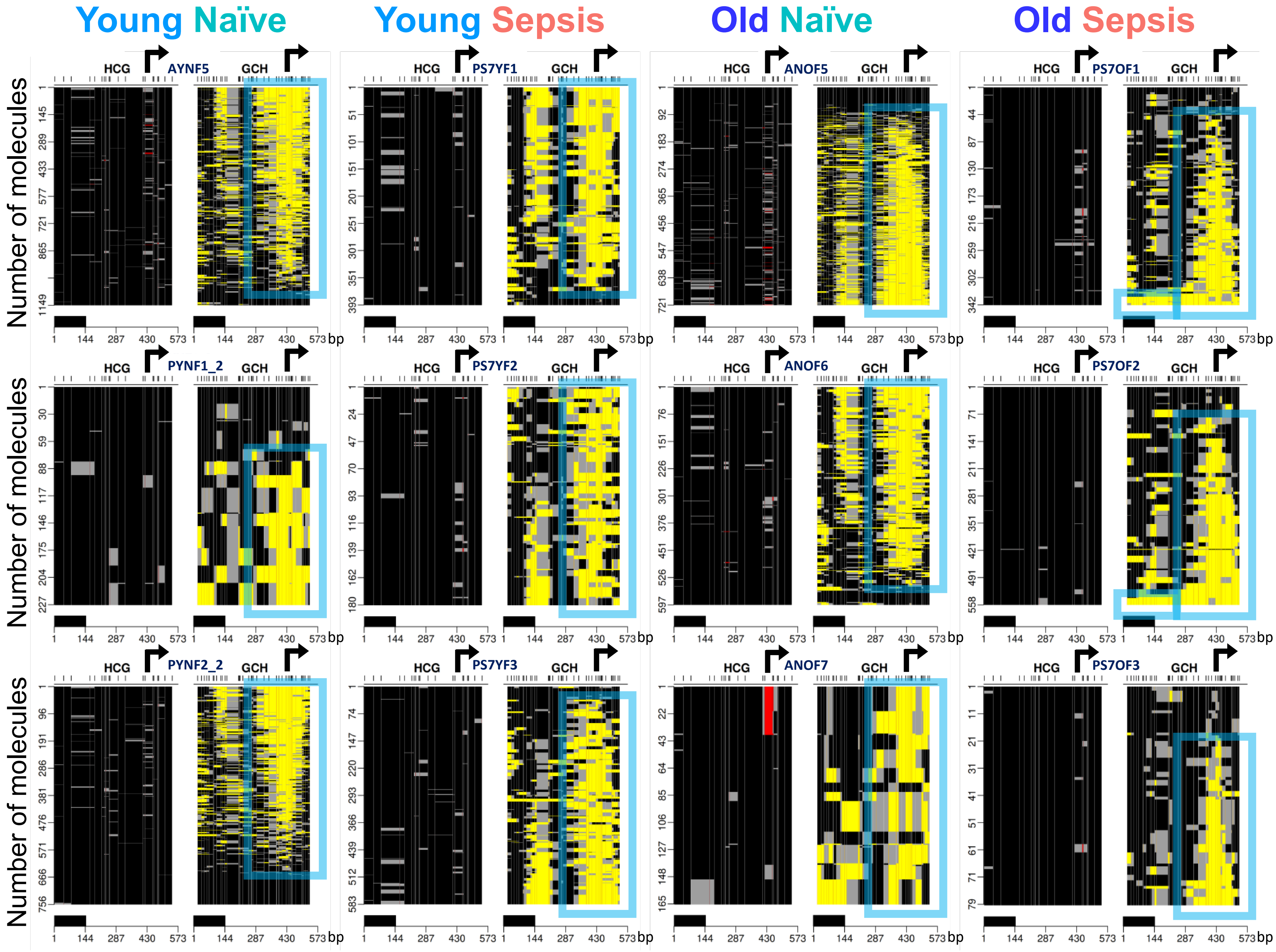

Vdr

NFR-containing promoter copies

Most HCG methylation likely arises from M.CviPI modification of accessible CCG sites

Endogenous methylation

Chromatin accessibility

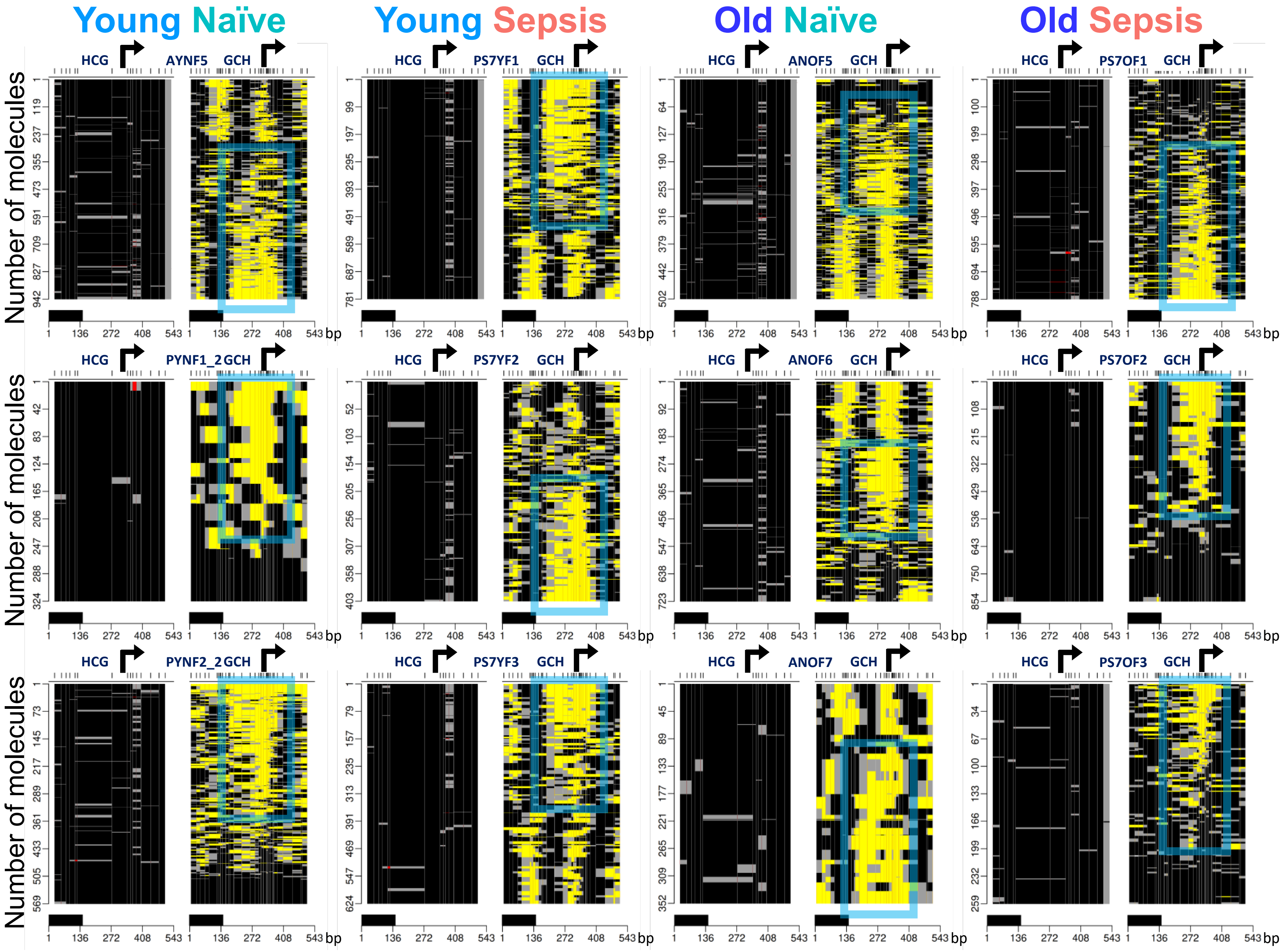

Il4ra

NFR-containing promoter copies

Most HCG methylation likely arises from M.CviPI modification of accessible CCG sites

Endogenous methylation

Chromatin accessibility

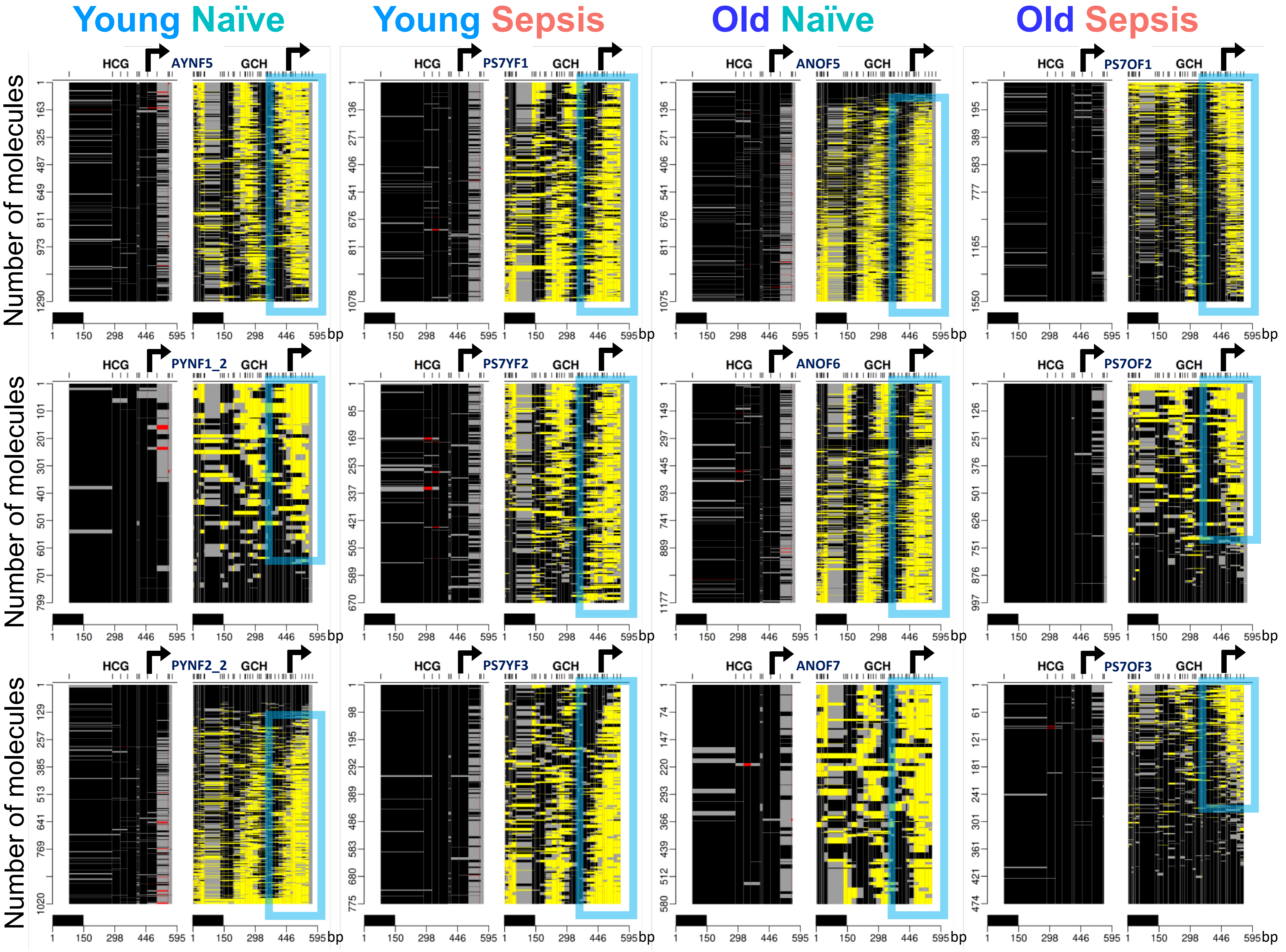

Atf6

NFR-containing promoter copies

Most HCG methylation likely arises from M.CviPI modification of accessible CCG sites

Endogenous methylation

Chromatin accessibility

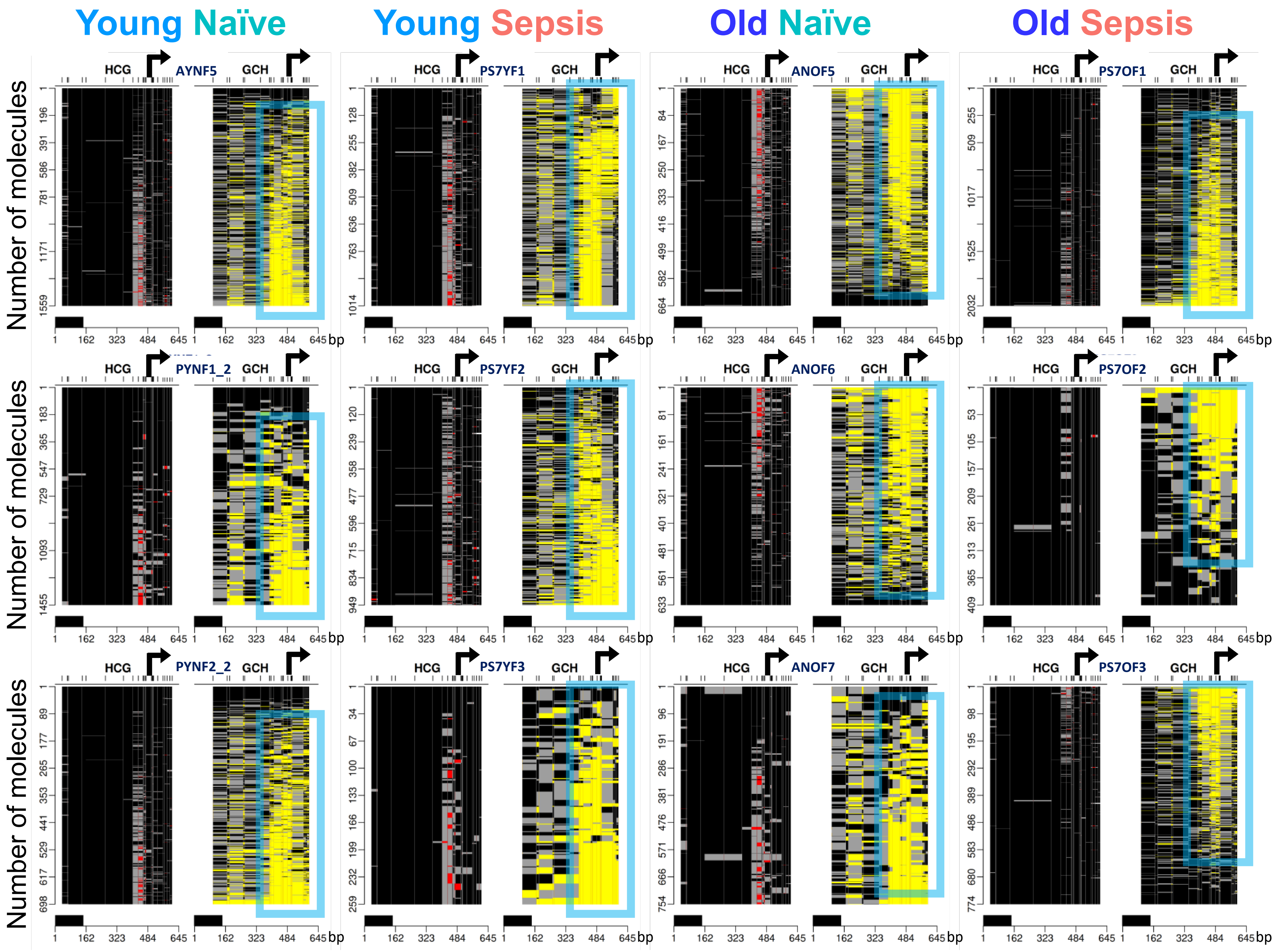

Il1rl2

NFR-containing promoter copies

Most HCG methylation likely arises from M.CviPI modification of accessible CCG sites

Endogenous methylation

Chromatin accessibility

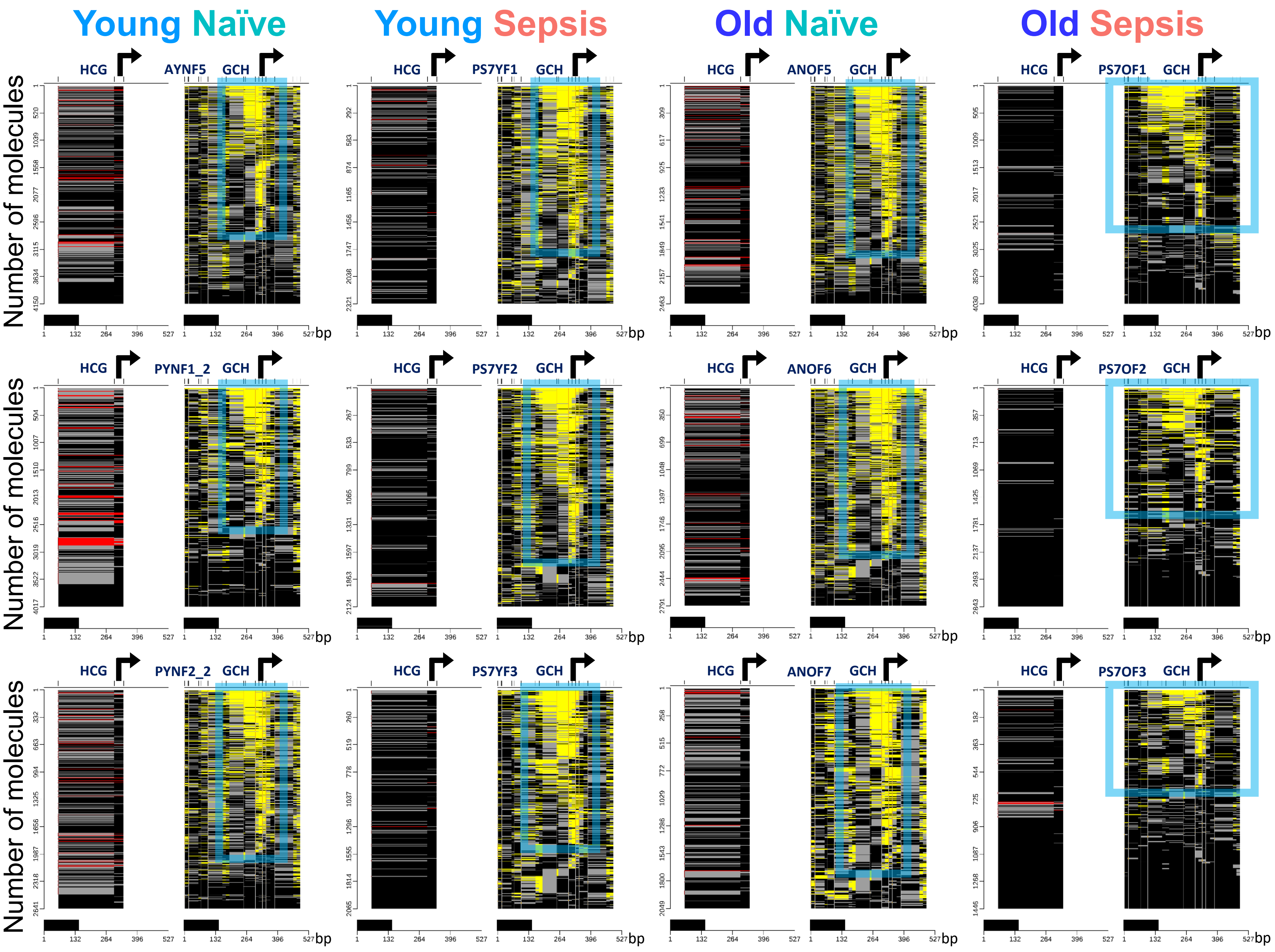

Class 9 promoter: *Tet2*

Constitutive NFR that is maintained under all conditions:

- Low, baseline levels of CpG methylation (any observed HCG methylation is likely attributable to M.CviPI methylating CCG sites in highly accessible regions)

Tet2

NFR-containing promoter copies

Most HCG methylation likely arises from M.CviPI modification of accessible CCG sites

Endogenous methylation

Chromatin accessibility

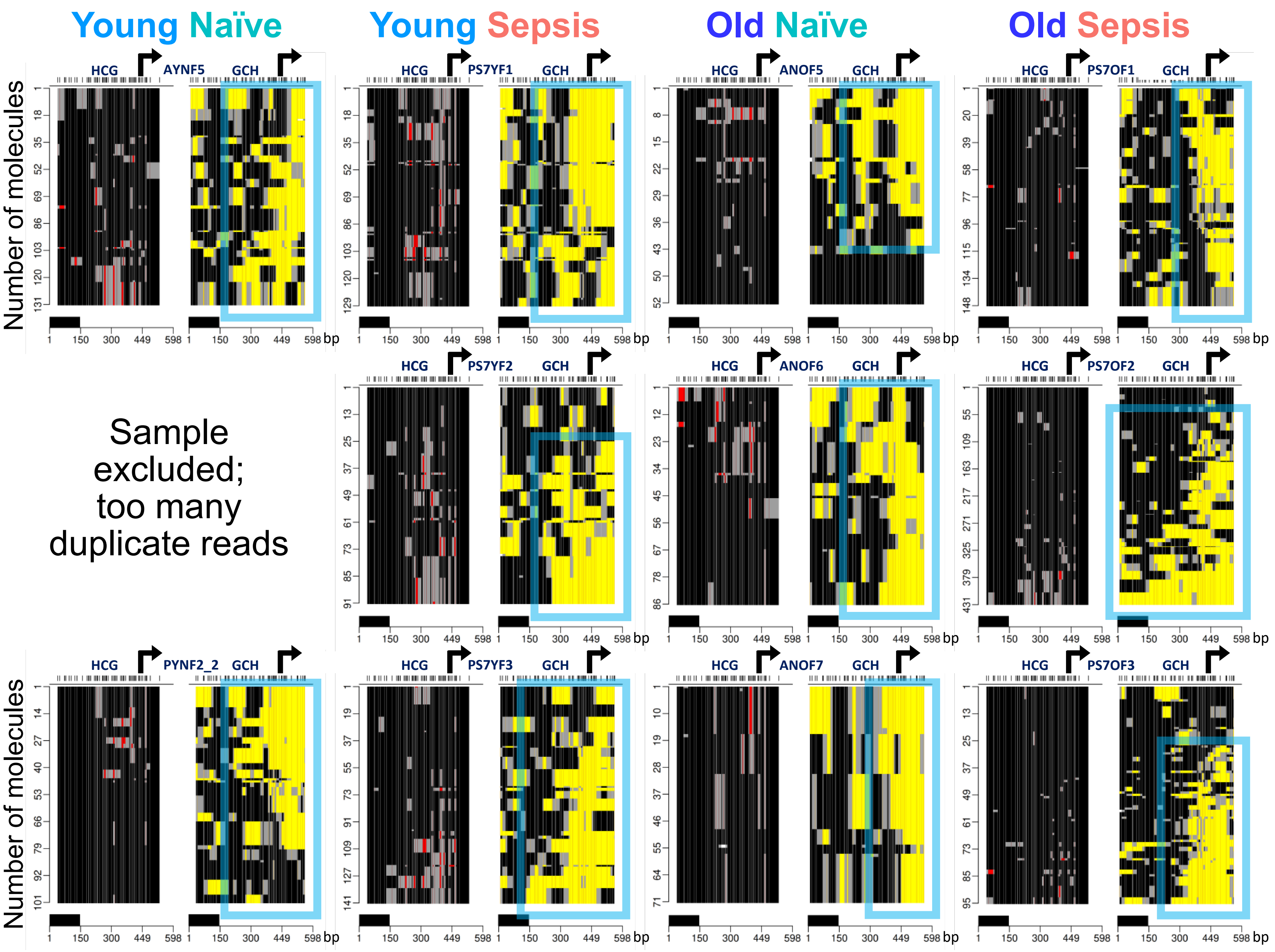

Supplement: Supplementary file 1 [file DataSheet1.pdf]
