# Supplementary material for: A murine model of sepsis induces age- and sex-specific chromatin remodeling in myeloid-derived suppressor cells
Source: Front Immunol. 2026 Mar 24;17:1750174. doi: 10.3389/fimmu.2026.1750174 (PMC13053262; doi:10.3389/fimmu.2026.1750174)
Supplement: Supplementary file 2 [file DataSheet2.pdf]

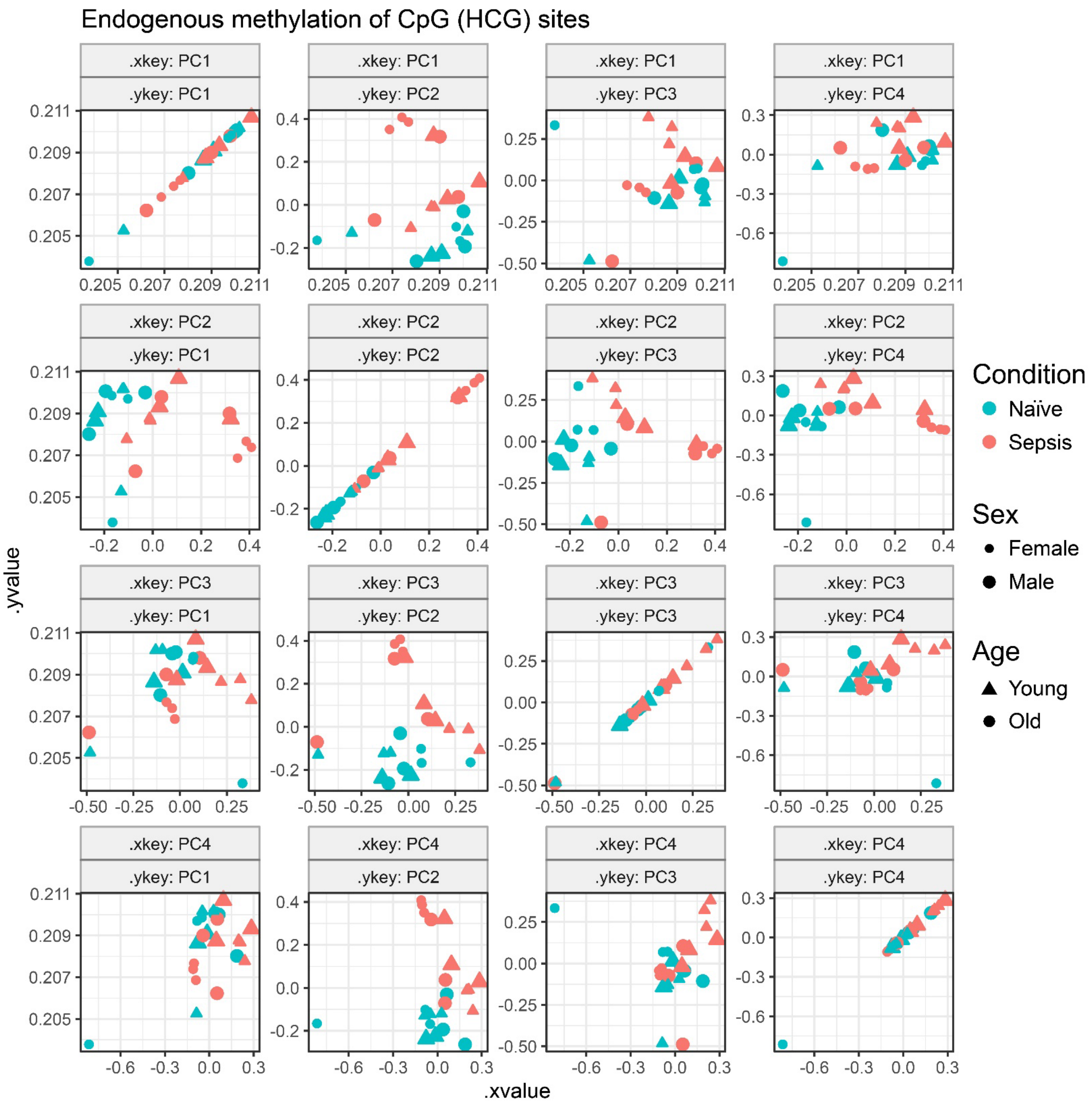

SUPPLEMENTARY FIGURE 1

Principal component analysis (PCA) of endogenous CpG methylation (HCG) in splenic $CD11b^{+}Gr1^{+}$ MDSCs. PCA was performed on the percent methylation of CpG (HCG) sites across all covered promoters passing quality filters and all MDSCs from male (excluding one naïve young male mouse) and female mice, young and old, with or without sepsis. Each point represents a single mouse. Color indicates condition (teal, naïve; red, sepsis), shape size denotes sex (small, female; large, male), and shape type differentiates age (circle, young; triangle, old). Principal components 1-4 are plotted in pairwise combinations to visualize clustering. Old septic females exhibit distinct separation, indicating strong CpG methylation alterations in response to sepsis.

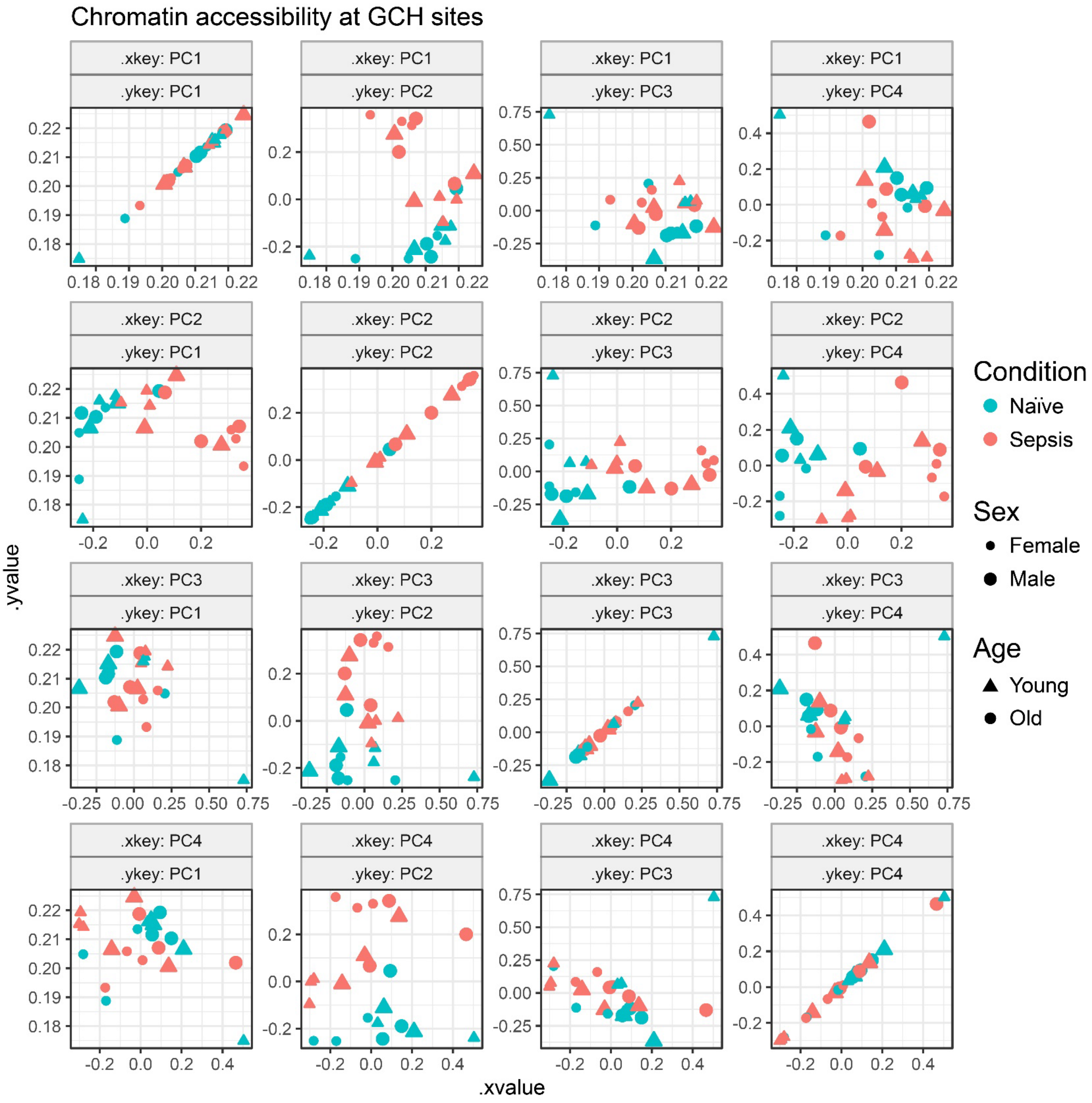

SUPPLEMENTARY FIGURE 2

PCA of GpC methylation (chromatin accessibility) in splenic CD11b^{+}Gr1^{+} MDSCs. PCA was performed using GCH methylation values from MAPit-FENGC to reflect promoter accessibility across all targeted promoters passing quality filters and all MDSC samples, excluding one naïve young male mouse. Each point represents a single mouse. Color indicates condition (teal naïve; red, sepsis), shape size denotes sex (small, female; large male), and shape type differentiates age (triangle, young; circle old). Separation by sepsis status is evident across multiple PCs, with the strongest divergence observed in female mice. In addition, these results suggest that sepsis induces distinct extents of chromatin remodeling, particularly between young and old females.
