# Supplementary material for: A murine model of sepsis induces age- and sex-specific chromatin remodeling in myeloid-derived suppressor cells
Source: Front Immunol. 2026 Mar 24;17:1750174. doi: 10.3389/fimmu.2026.1750174 (PMC13053262; doi:10.3389/fimmu.2026.1750174)

Supplementary File 1 to accompany Charles et al.

Heatmaps of HCG and GCH methylation patterns of targeted promoters in **Male mice**

Class 1 promoter: *S100a9*

Strongest response to CLP + DCS of all profiled promoters:

- Opens in most mice of all septic cohorts, with sex- and age-specific differences
- Nucleosome-free region (NFR) formation consistent with activated transcription
- Moderate levels of CpG methylation, with sepsis-specific demethylation

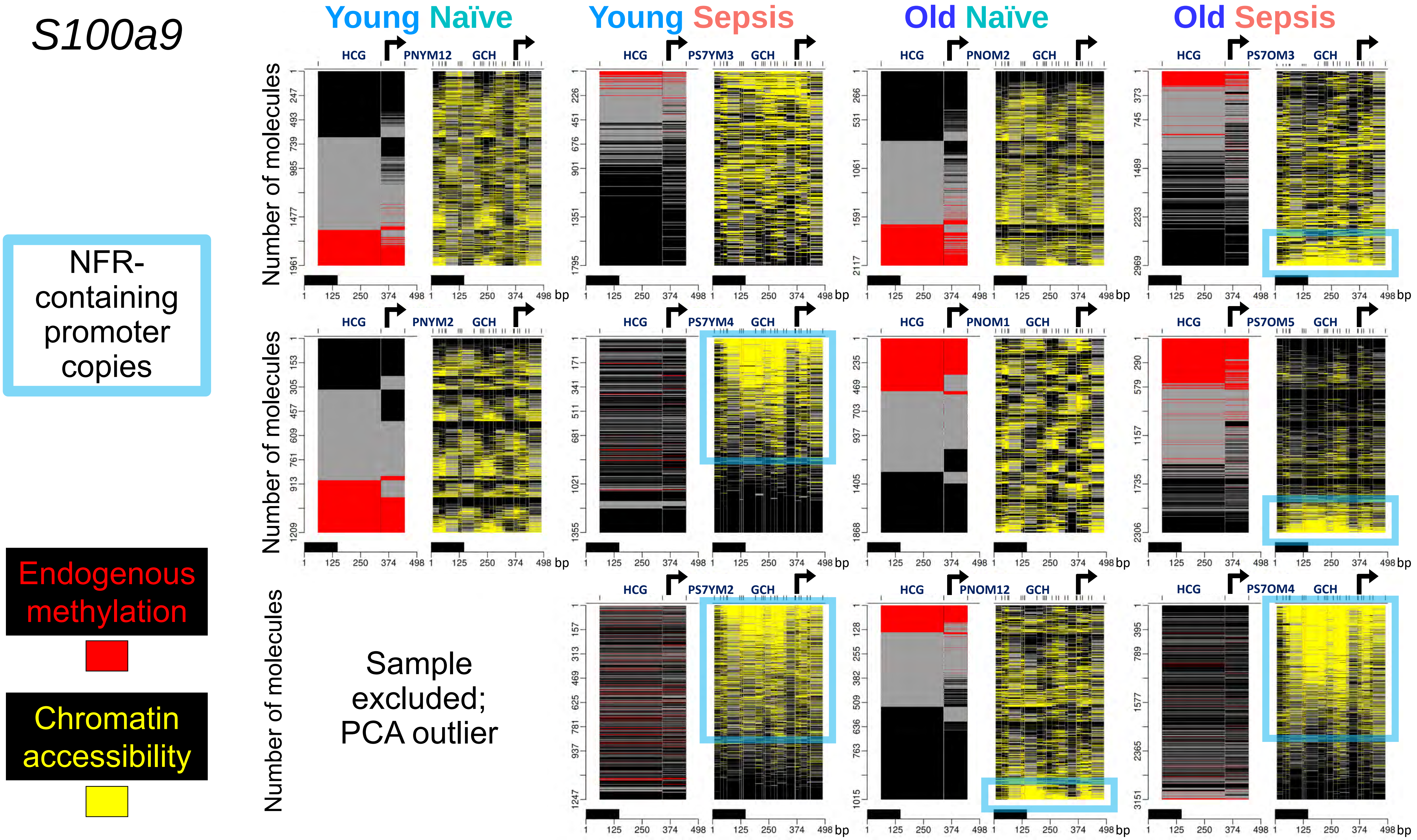
S100a9
Young Naïve
Young Sepsis
Old Naïve
Old Sepsis
NFR-containing promoter copies
Endogenous methylation
Chromatin accessibility
Number of molecules
HCG
GCH
PNYM12
PNYM2
PS7YM3
PS7YM4
PS7YM2
PNOM2
PNOM1
PNOM12
PS7OM3
PS7OM5
PS7OM4
bp
Sample excluded; PCA outlier

Class 2 promoters: *Cxcr2*, *Lcn2*, *Ccl3*, *Nos2*, *Ptgs2*, *Lgals9*, *Rnase2a*

Strong responses to CLP + DCS:

- NFR formation consistent with activated transcription
- Sex- and age-specific differences—promoter opening in Old Septic Female but in both Young and Old Septic Males
- Based on the fraction of NFR-bearing promoters, similar magnitude of sepsis response in both sexes
- CpG methylation levels ranging from background to fully methylated, with sepsis-specific demethylation at some loci

Note: Chromatin remodeling of Class 1 promoter and Class 3 promoters confirm CLP + DCS response in Female Young Sepsis mice, i.e., lack of accessibility is not due to a weak septic stimulus in some mice.

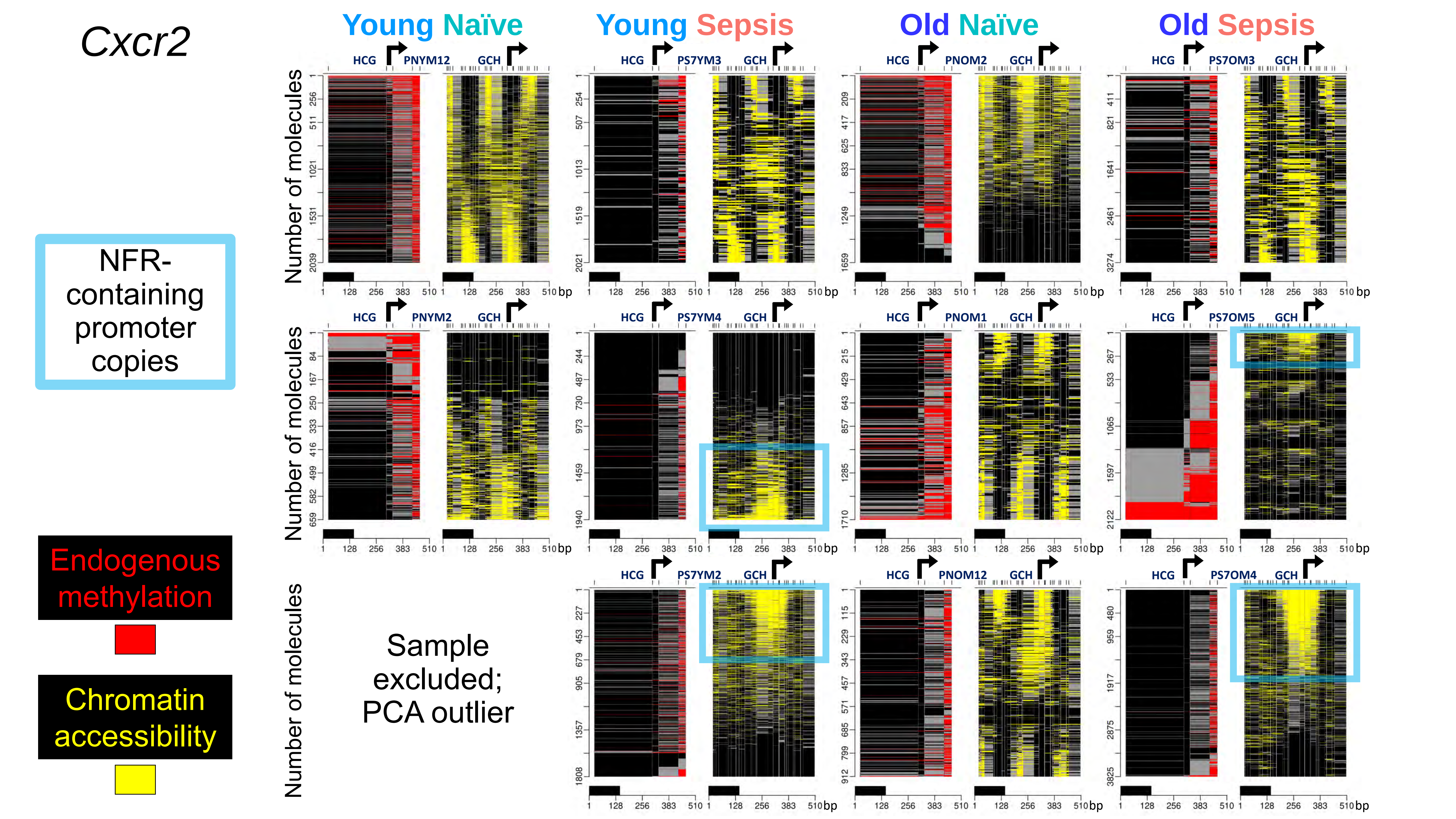

Cxcr2
Young Naïve
Young Sepsis
Old Naïve
Old Sepsis
HCG
GCH
PNYM12
PS7YM3
PNOM2
PS7OM3
PNYM2
PS7YM4
PNOM1
PS7OM5
PS7YM2
PNOM12
PS7OM4
Number of molecules
1 128 256 383 510 bp
NFR-containing promoter copies
Sample excluded; PCA outlier
Endogenous methylation
Chromatin accessibility

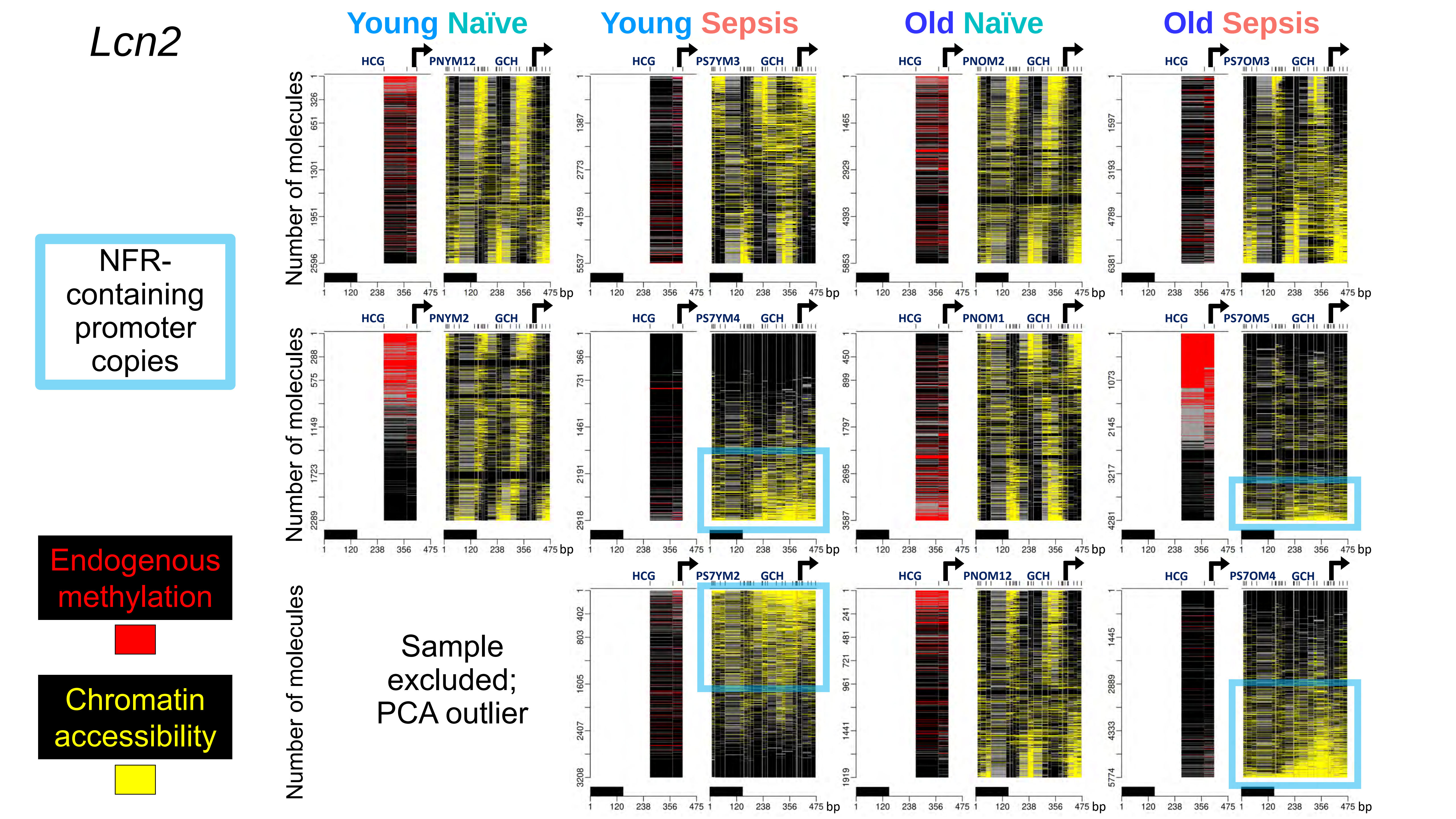

Lcn2
Young Naïve
Young Sepsis
Old Naïve
Old Sepsis
NFR-containing promoter copies
Endogenous methylation
Chromatin accessibility
Number of molecules
HCG
GCH
PNYM12
PNYM2
PS7YM3
PS7YM4
PS7YM2
PNOM2
PNOM1
PNOM12
PS7OM3
PS7OM5
PS7OM4
Sample excluded; PCA outlier
bp

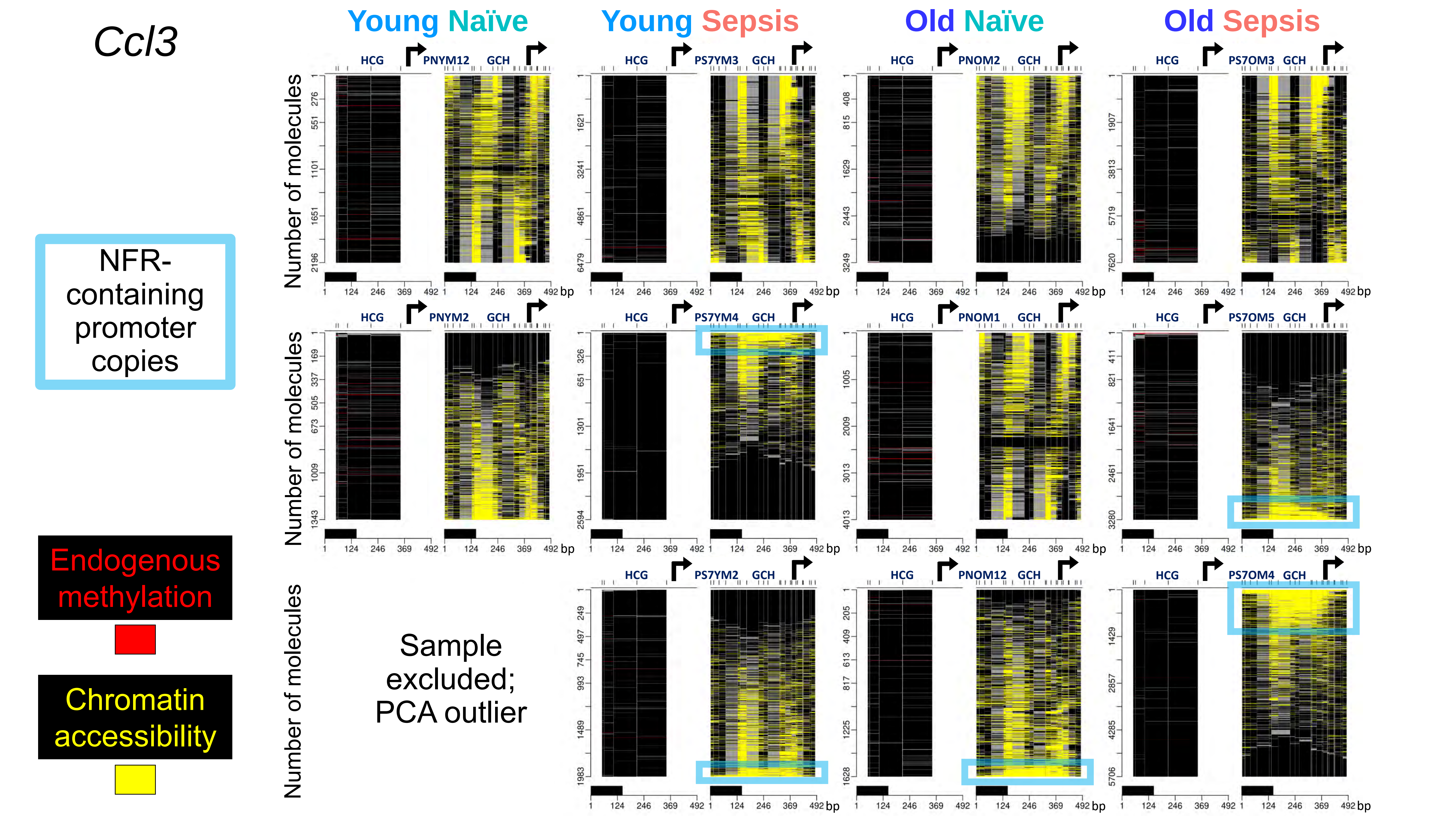
Ccl3
Young Naïve
Young Sepsis
Old Naïve
Old Sepsis
NFR-containing promoter copies
Endogenous methylation
Chromatin accessibility
Number of molecules
HCG
GCH
PNYM12
PNYM2
PS7YM3
PS7YM4
PS7YM2
PNOM2
PNOM1
PNOM12
PS7OM3
PS7OM5
PS7OM4
Sample excluded; PCA outlier
1 124 246 369 492 bp

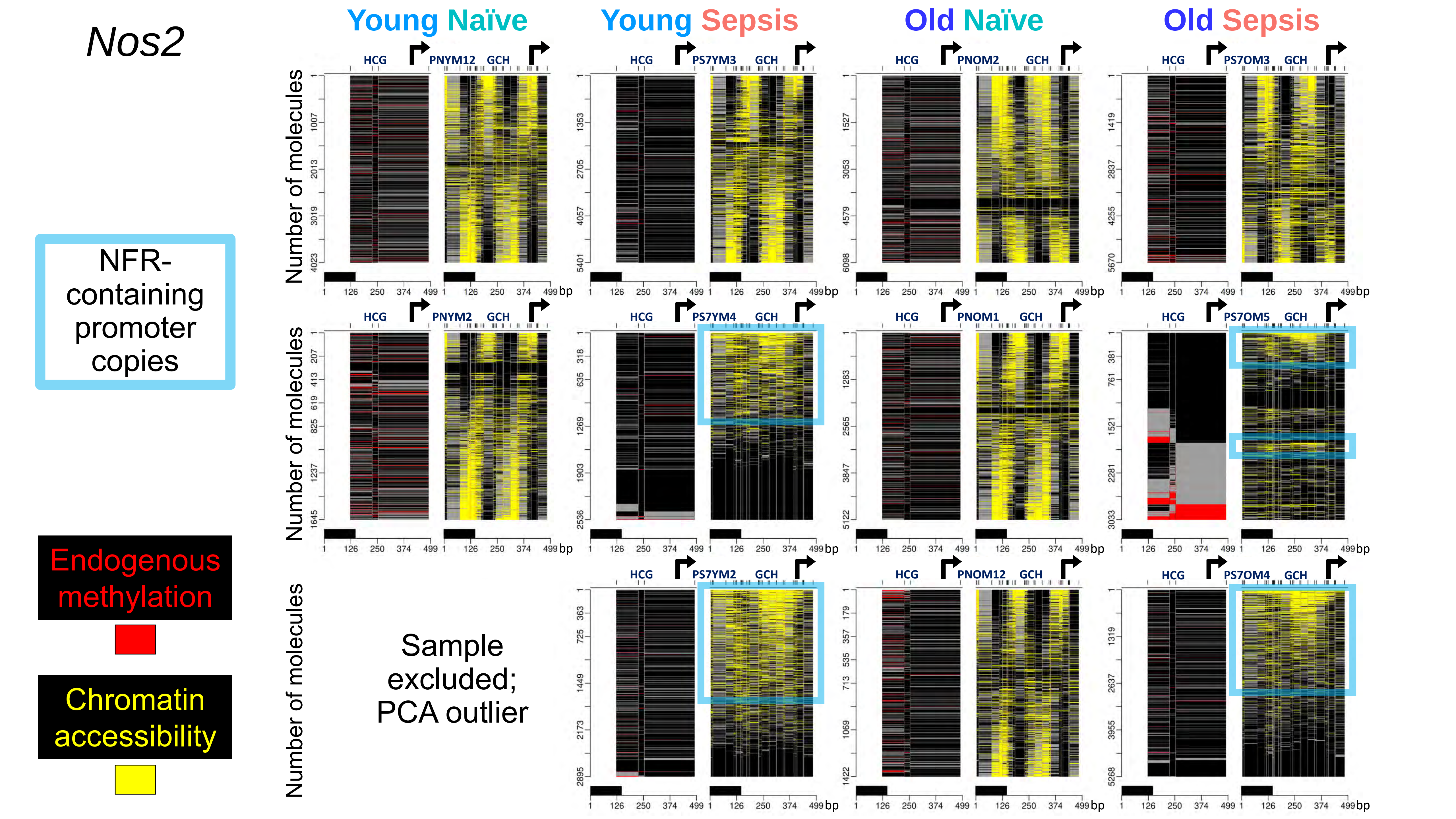

Nos2
Young Naïve
Young Sepsis
Old Naïve
Old Sepsis
NFR-containing promoter copies
Endogenous methylation
Chromatin accessibility
Sample excluded; PCA outlier
Number of molecules
HCG
GCH
PNYM12
PS7YM3
PNOM2
PS7OM3
PNYM2
PS7YM4
PNOM1
PS7OM5
PS7YM2
PNOM12
PS7OM4
bp

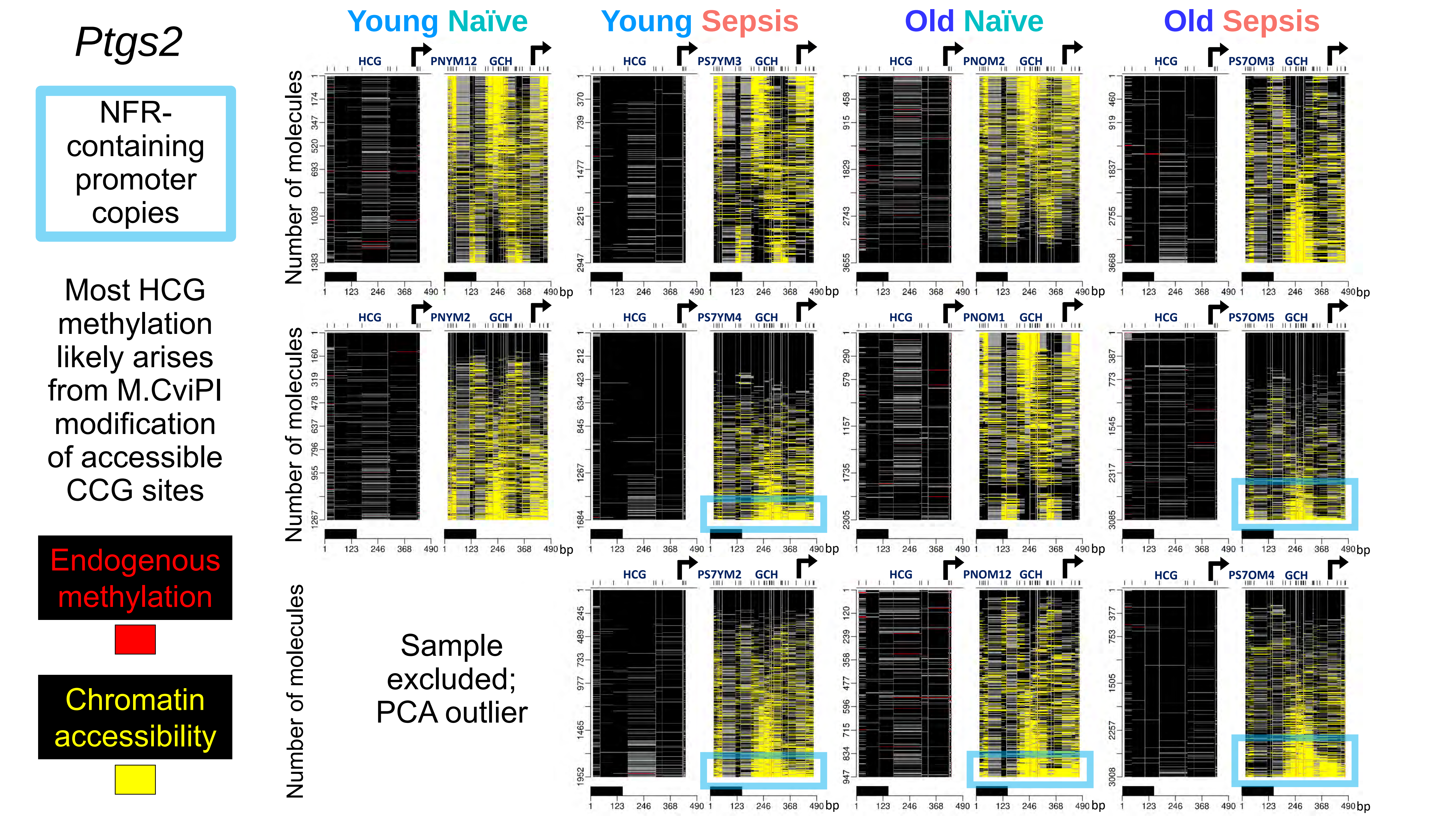

Ptgs2
NFR-containing promoter copies
Most HCG methylation likely arises from M.CviPI modification of accessible CCG sites
Endogenous methylation
Chromatin accessibility
Young Naïve
Young Sepsis
Old Naïve
Old Sepsis
Number of molecules
HCG
GCH
PNYM12
PNYM2
PS7YM3
PS7YM4
PS7YM2
PNOM2
PNOM1
PNOM12
PS7OM3
PS7OM5
PS7OM4
Sample excluded; PCA outlier
1 123 246 368 490 bp

Lgals9

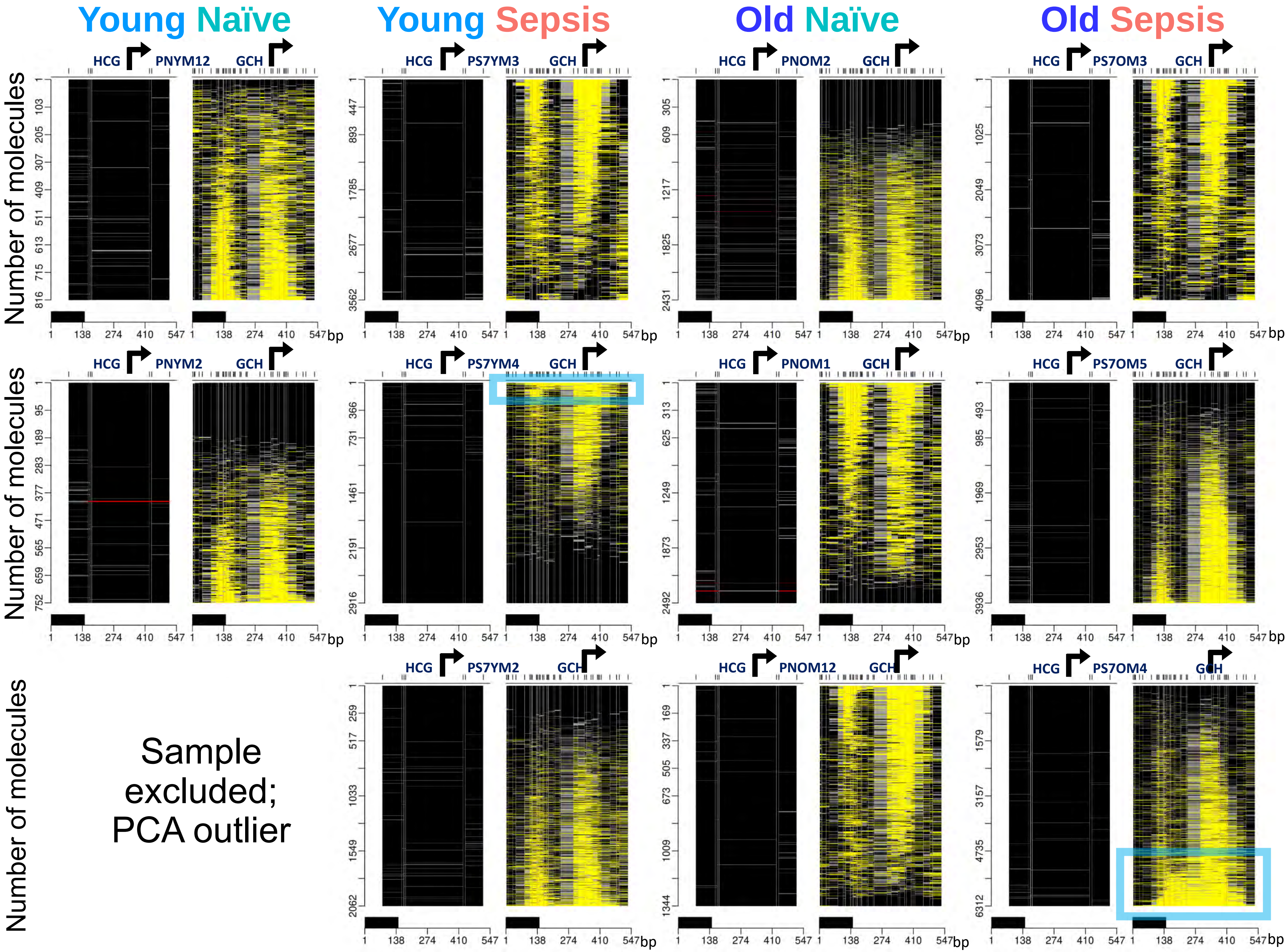

NFR-containing promoter copies

Endogenous methylation

Chromatin accessibility

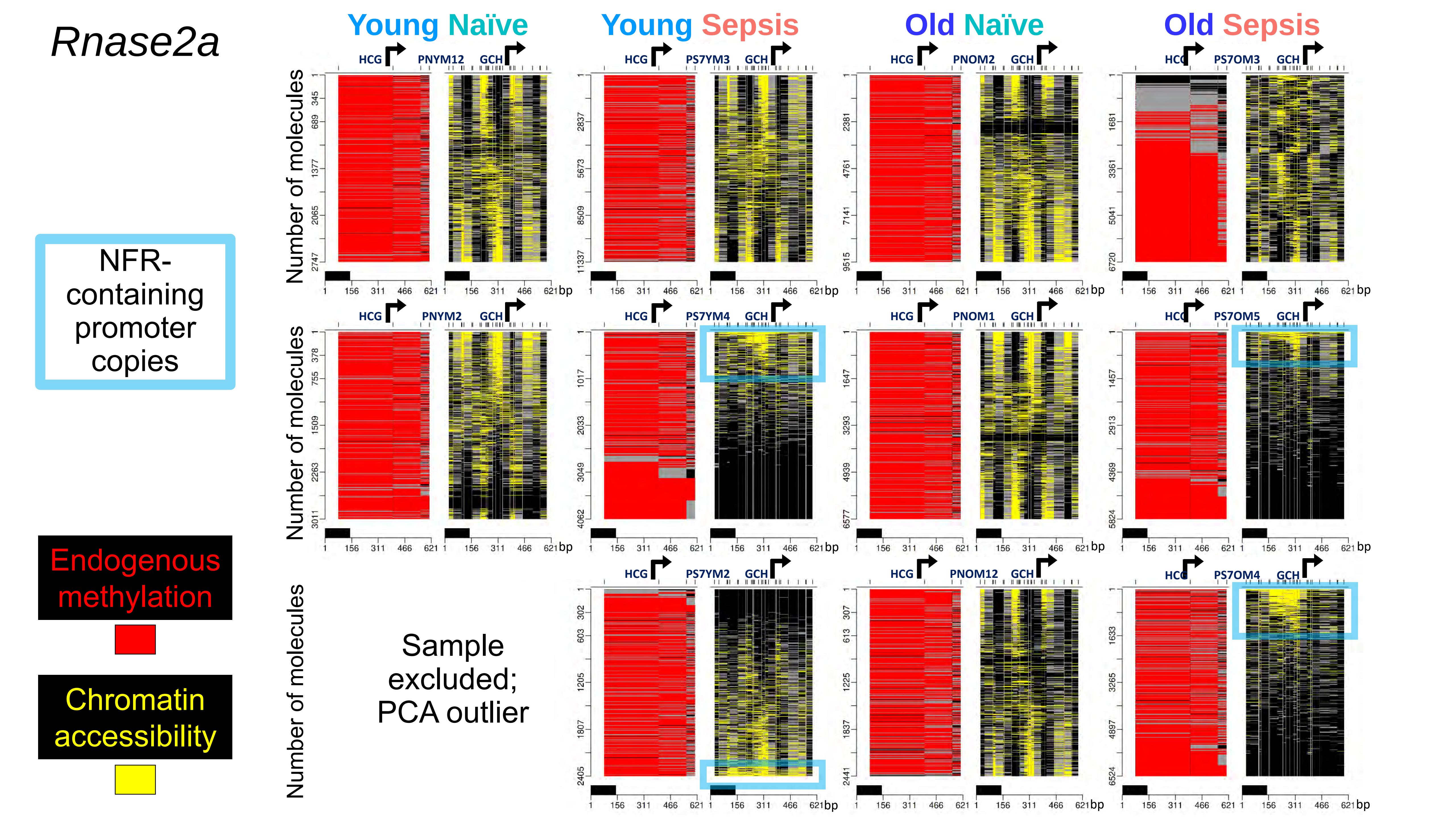

Rnase2a
Young Naïve
Young Sepsis
Old Naïve
Old Sepsis
HCG
GCH
PNYM12
PS7YM3
PNOM2
PS7OM3
PNYM2
PS7YM4
PNOM1
PS7OM5
PS7YM2
PNOM12
PS7OM4
Number of molecules
1 156 311 466 621 bp
NFR-containing promoter copies
Endogenous methylation
Chromatin accessibility
Sample excluded; PCA outlier

Class 3 promoters: *S100a8*, *Mmp8*

Response to CLP + DCS across all cohorts:

- No sex- or age-specific differences
- Nucleosome-free region (NFR) formation consistent with activated transcription
- Decreased accessibility upstream and downstream of NFRs at TSS
- Moderate levels of CpG methylation, with sepsis-specific demethylation

Note: Confirms chromatin remodeling in response to CLP + DCS in all male mice (even PS7YM3).

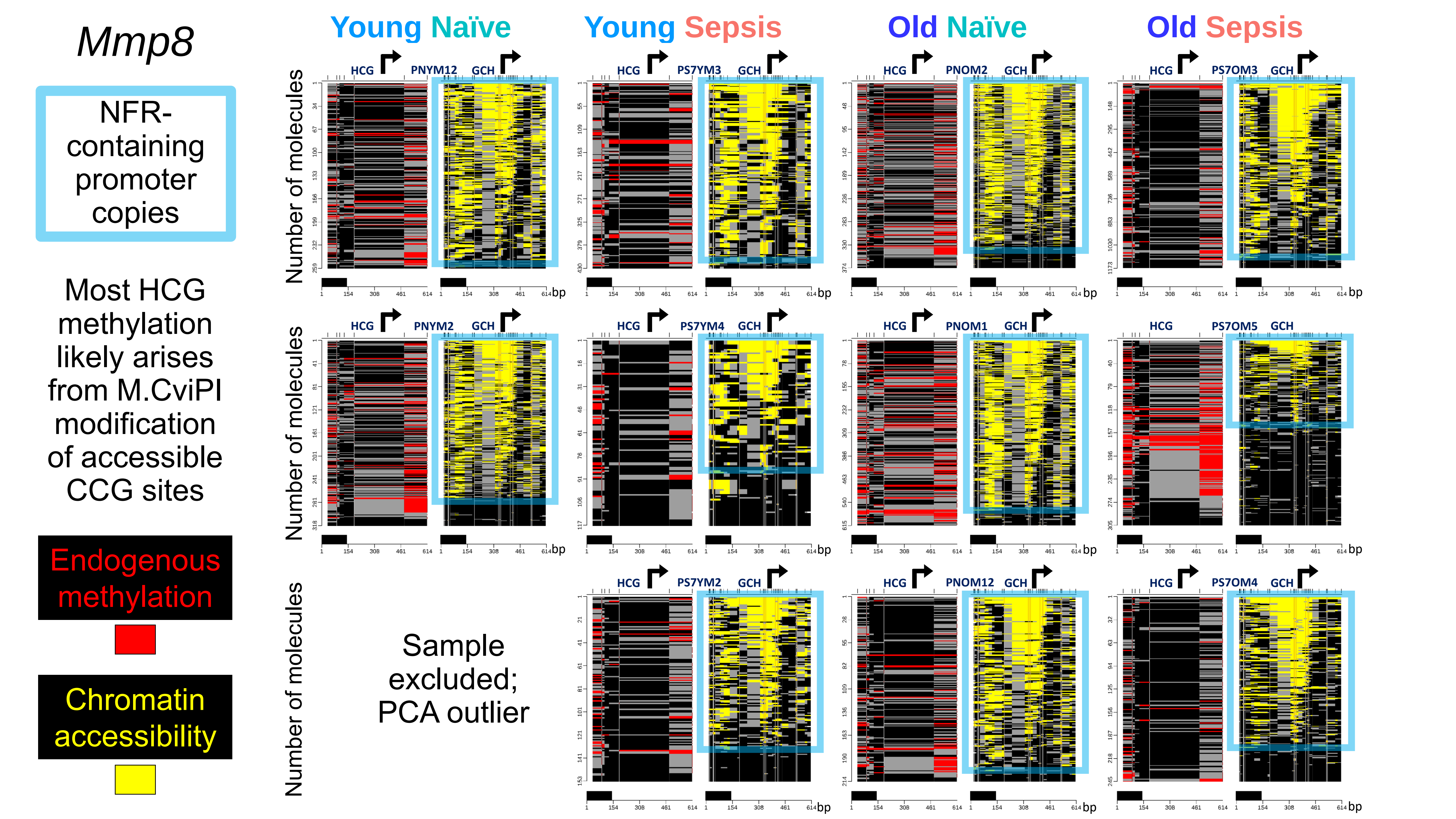

Mmp8
NFR-containing promoter copies
Most HCG methylation likely arises from M.CviPI modification of accessible CCG sites
Endogenous methylation
Chromatin accessibility
Young Naïve
Young Sepsis
Old Naïve
Old Sepsis
Number of molecules
HCG
GCH
PNYM12
PNYM2
PS7YM3
PS7YM4
PS7YM2
PNOM2
PNOM1
PNOM12
PS7OM3
PS7OM5
PS7OM4
1 154 308 461 614 bp
Sample excluded; PCA outlier

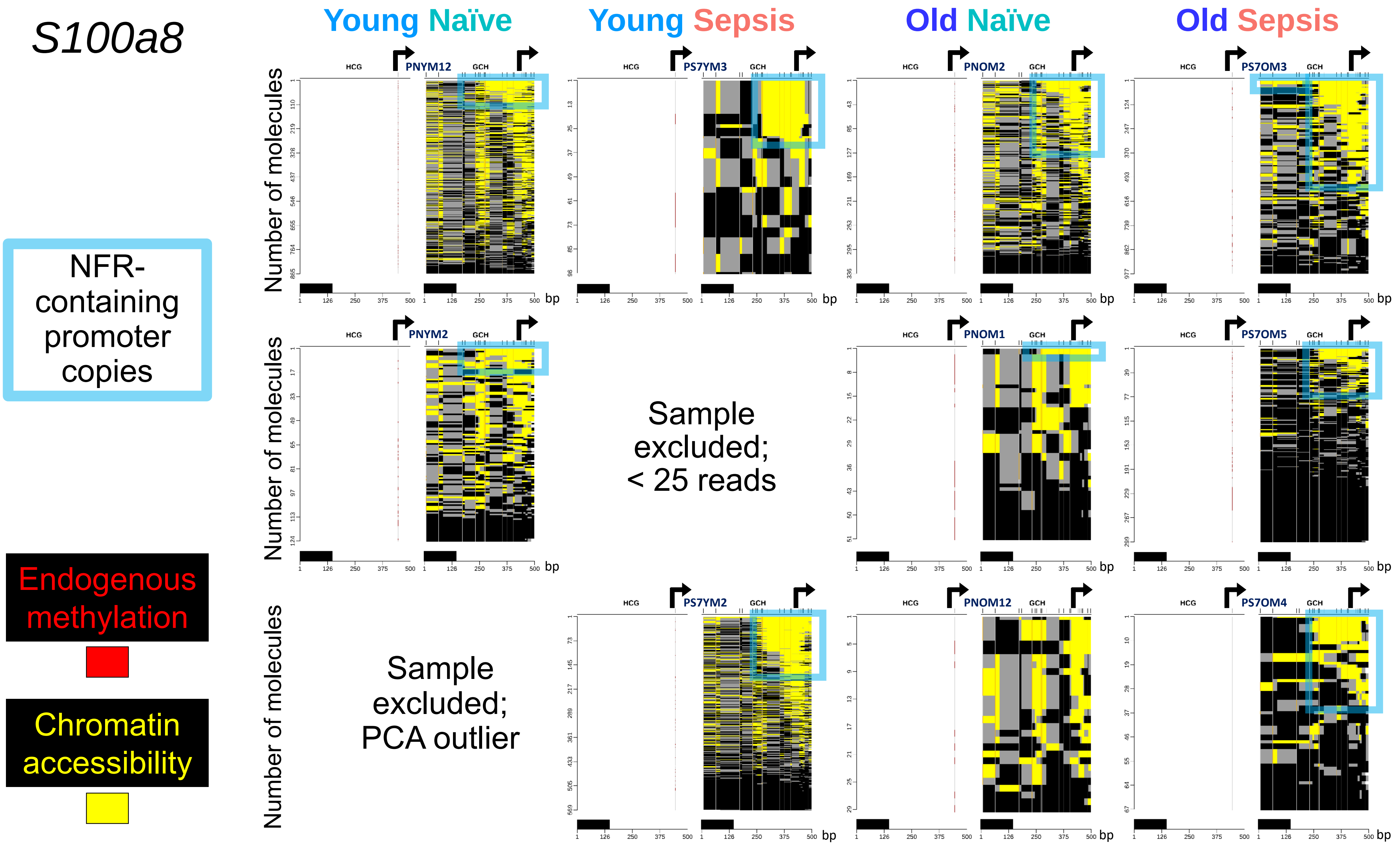
S100a8
Young Naïve
Young Sepsis
Old Naïve
Old Sepsis
NFR-containing promoter copies
Endogenous methylation
Chromatin accessibility
Number of molecules
HCG
GCH
PNYM12
PS7YM3
PNOM2
PS7OM3
PNYM2
PNOM1
PS7OM5
PS7YM2
PNOM12
PS7OM4
Sample excluded; < 25 reads
Sample excluded; PCA outlier
bp

Class 4 promoter: *Fyb*

No response to CLP + DCS in any Male mice (only in Female Septic mice):

- Strong sex divergence
- Nucleosome-free region (NFR) formation consistent with activated transcription
- Decreased accessibility upstream and downstream of NFRs at TSS
- Low, baseline levels of CpG methylation.

Note: Chromatin remodeling in response to CLP + DCS in other classes in male septic mice rules out insufficient sepsis response.

Fyb

NFR-containing promoter copies

Most HCG methylation likely arises from M.CviPI modification of accessible CCG sites

Endogenous methylation

Chromatin accessibility

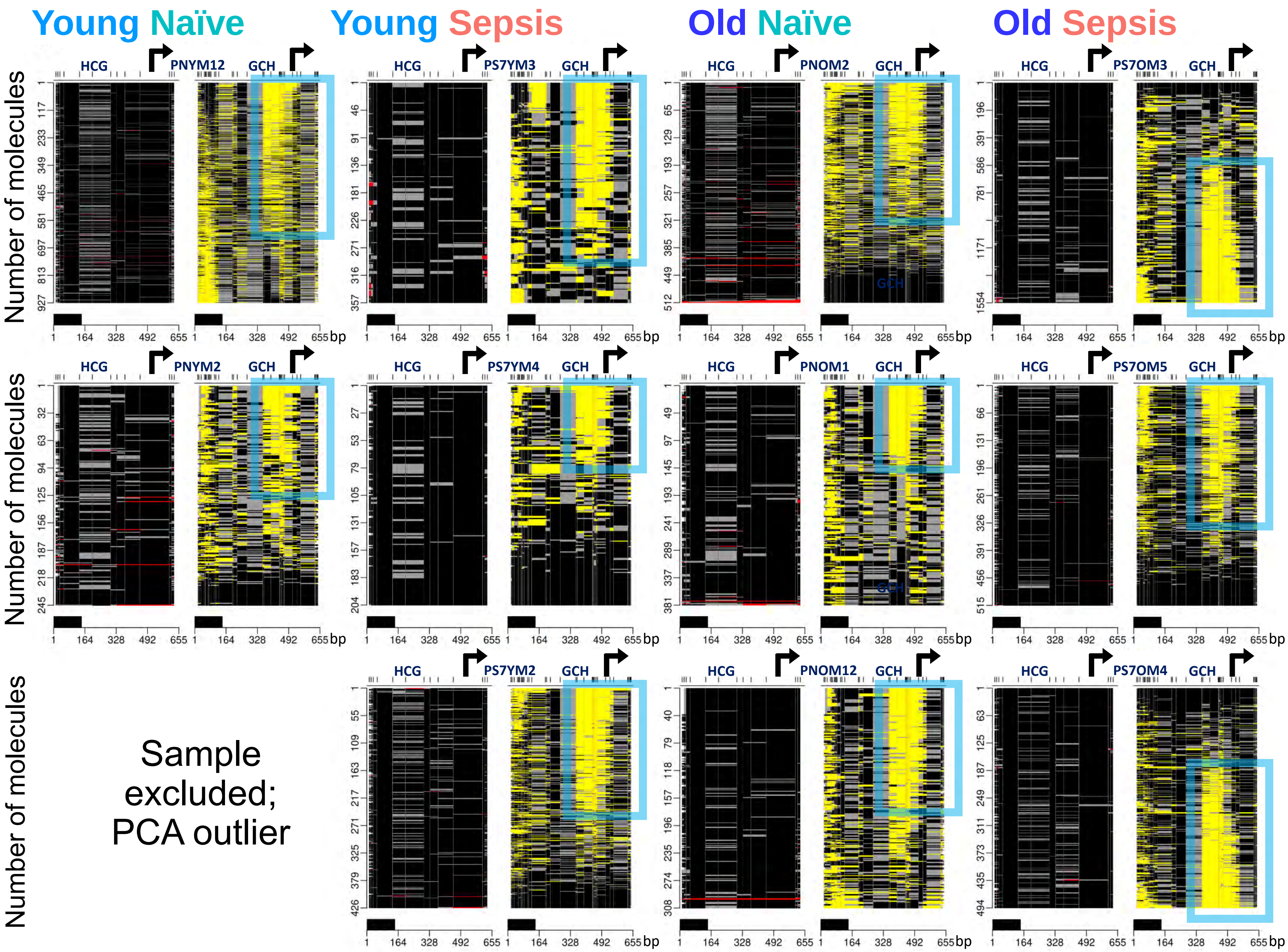

Class 5 promoters: *Lyz1*, *Retn*, *F7*, *Pmp22, Vnn1*, *Serpina1a*, *Emp1*, *Dab2, Vsig4*, *Mmp19*

No NRF formation in response to CLP + DCS across all cohorts:

- High levels of endogenous CpG methylation (at HCGs)
- Accessibility pattern consistent with disorganized or random nucleosome arrays
- Decreased accessibility in old sepsis samples

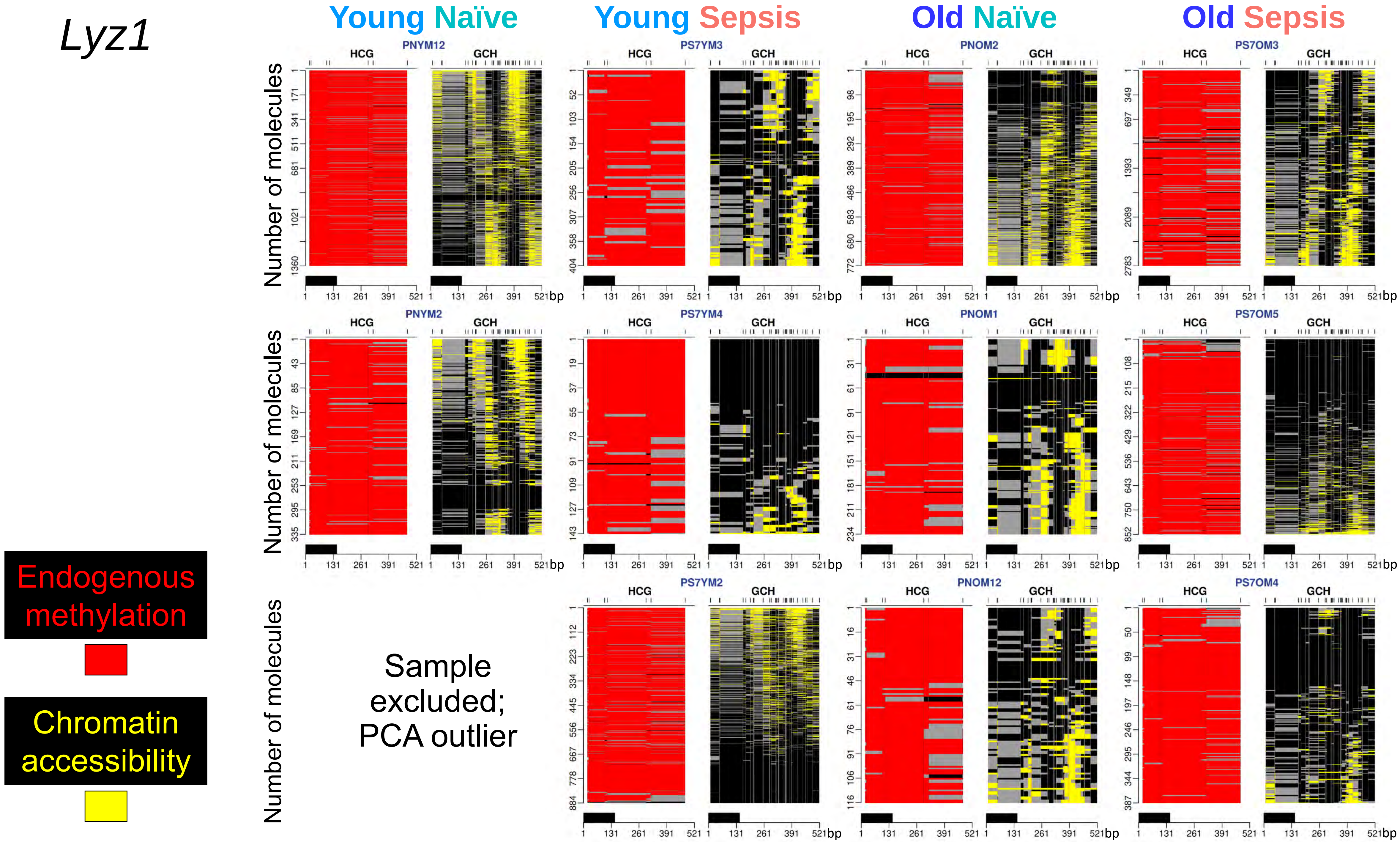
Lyz1
Young Naïve
Young Sepsis
Old Naïve
Old Sepsis
PNYM12
PS7YM3
PNOM2
PS7OM3
PNYM2
PS7YM4
PNOM1
PS7OM5
PS7YM2
PNOM12
PS7OM4
HCG
GCH
Number of molecules
bp
Sample excluded; PCA outlier
Endogenous methylation
Chromatin accessibility

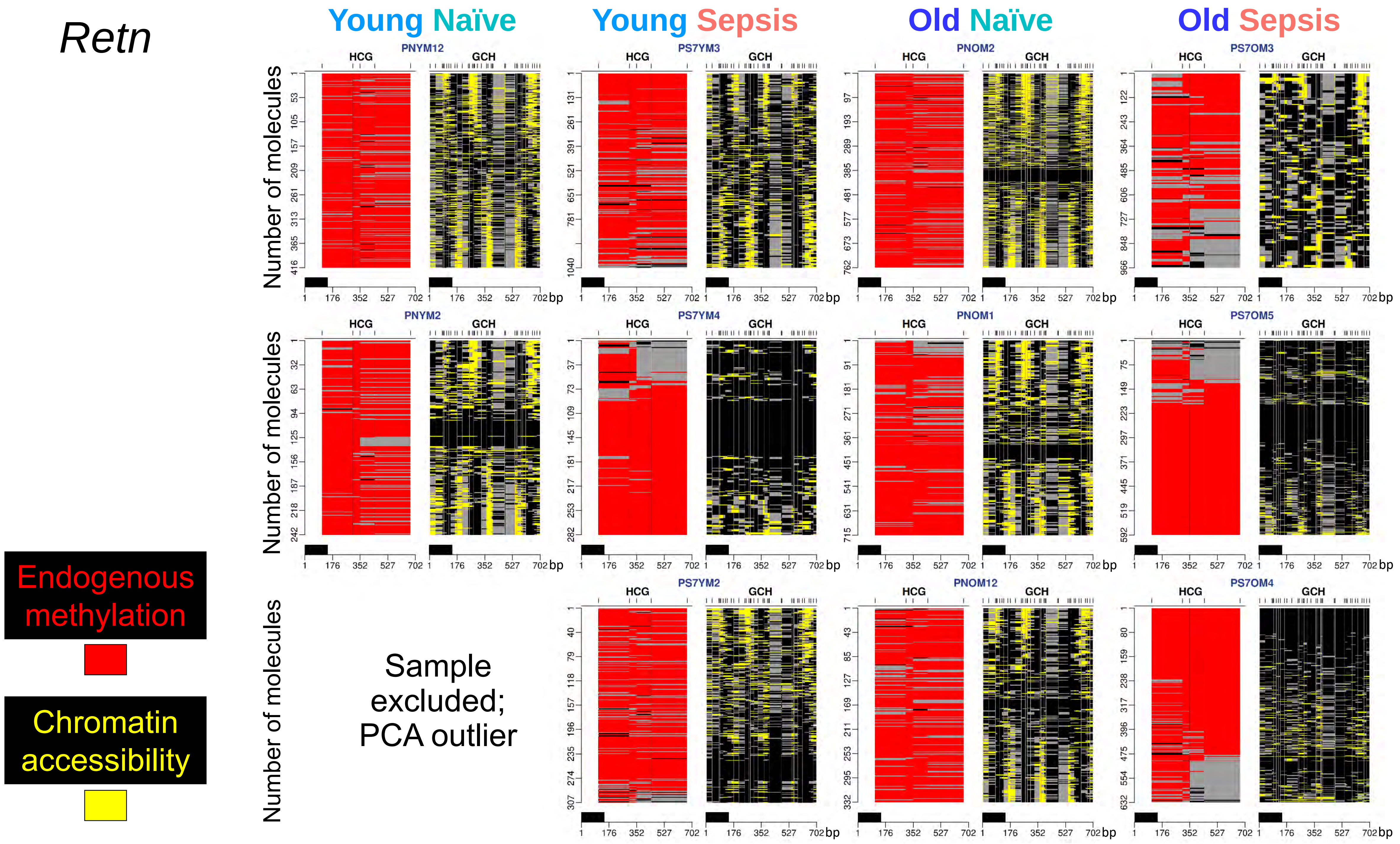

Retn
Young Naïve
Young Sepsis
Old Naïve
Old Sepsis
PNYM12
PS7YM3
PNOM2
PS7OM3
PNYM2
PS7YM4
PNOM1
PS7OM5
PS7YM2
PNOM12
PS7OM4
HCG
GCH
Number of molecules
bp
Sample excluded; PCA outlier
Endogenous methylation
Chromatin accessibility

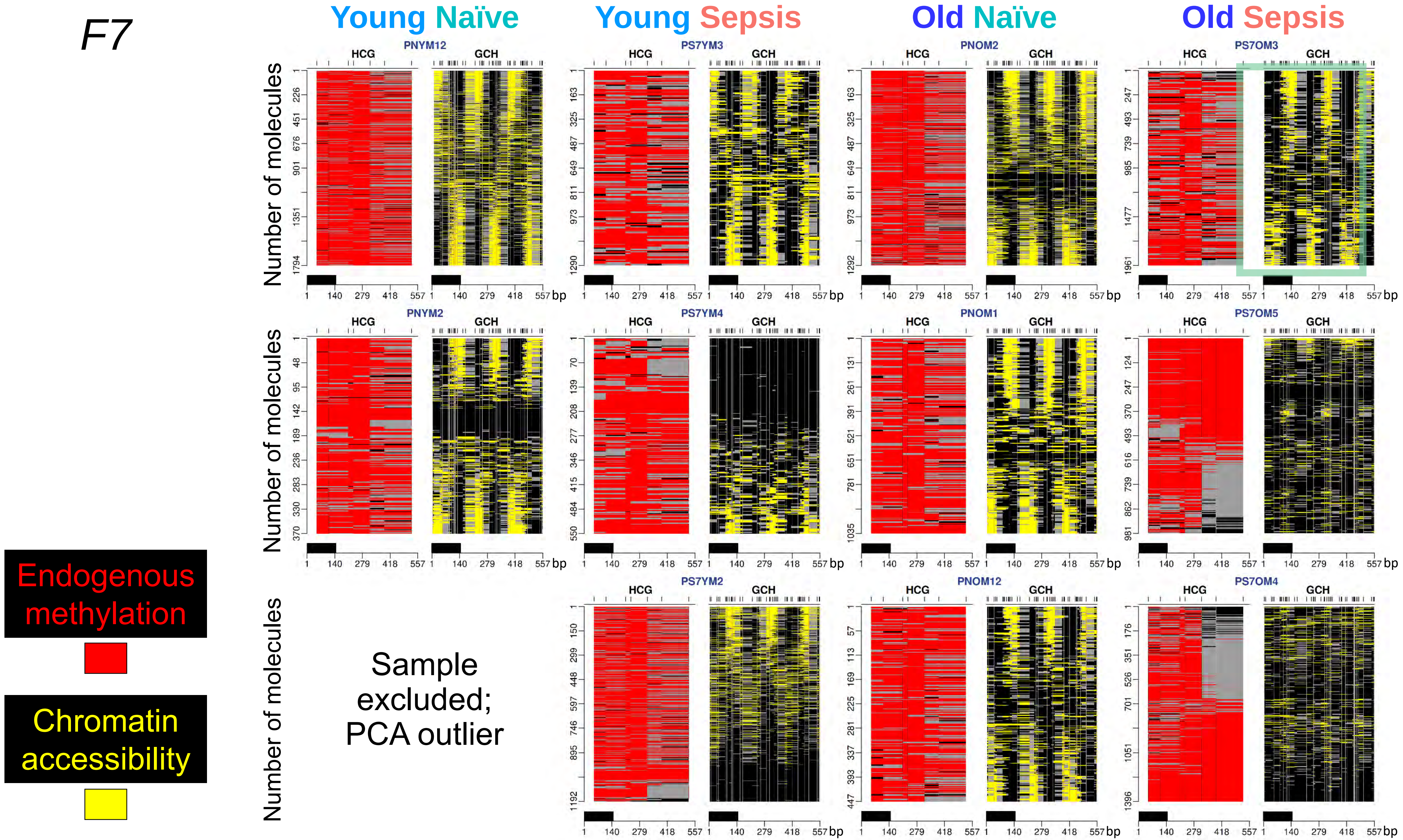
F7
Young Naïve
Young Sepsis
Old Naïve
Old Sepsis
PNYM12
PS7YM3
PNOM2
PS7OM3
PNYM2
PS7YM4
PNOM1
PS7OM5
PS7YM2
PNOM12
PS7OM4
HCG
GCH
Number of molecules
bp
Sample excluded; PCA outlier
Endogenous methylation
Chromatin accessibility

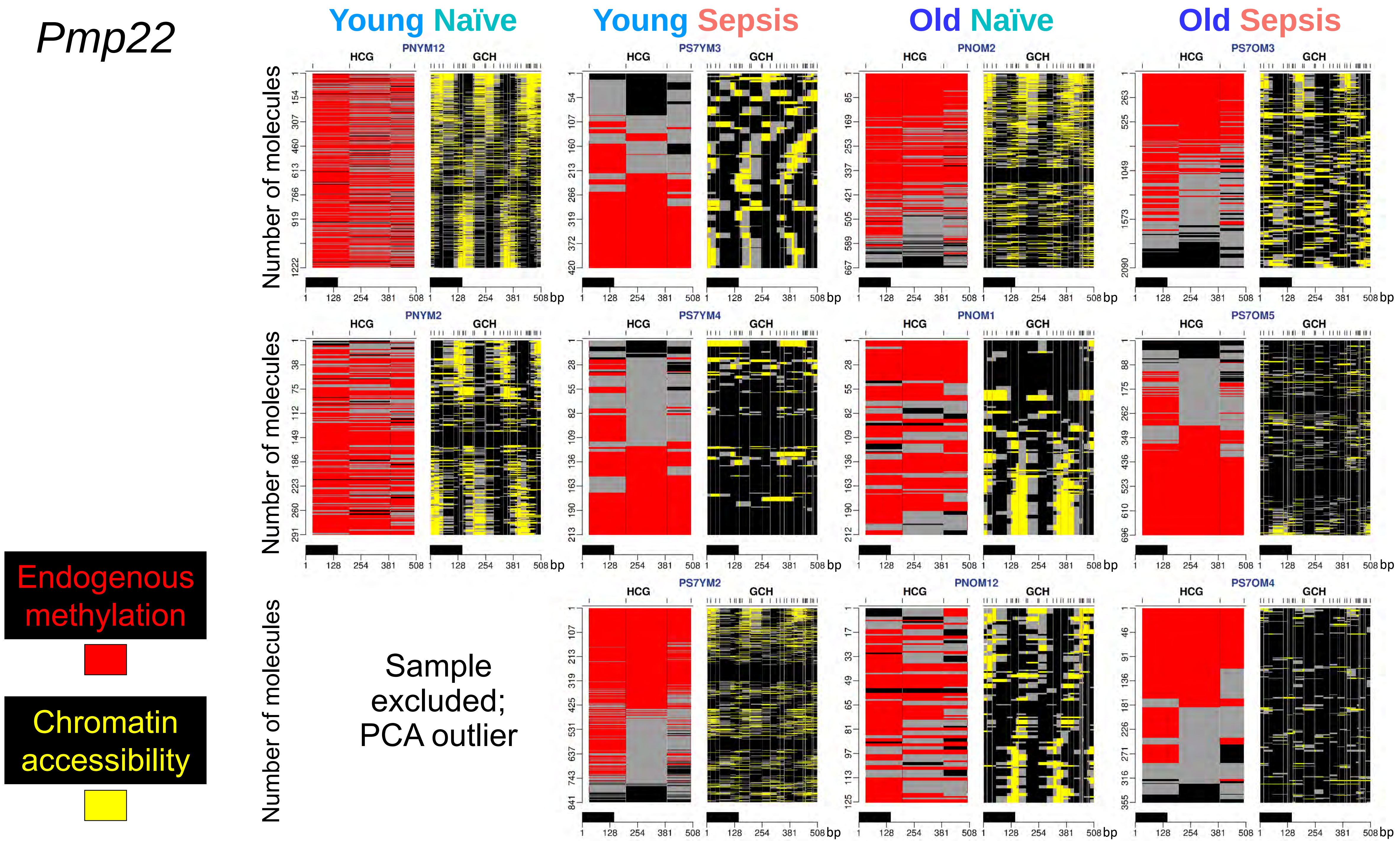
Pmp22
Young Naïve
Young Sepsis
Old Naïve
Old Sepsis
PNYM12
PNYM2
PS7YM3
PS7YM4
PS7YM2
PNOM2
PNOM1
PNOM12
PS7OM3
PS7OM5
PS7OM4
HCG
GCH
Number of molecules
1 128 254 381 508 bp
Sample excluded; PCA outlier
Endogenous methylation
Chromatin accessibility

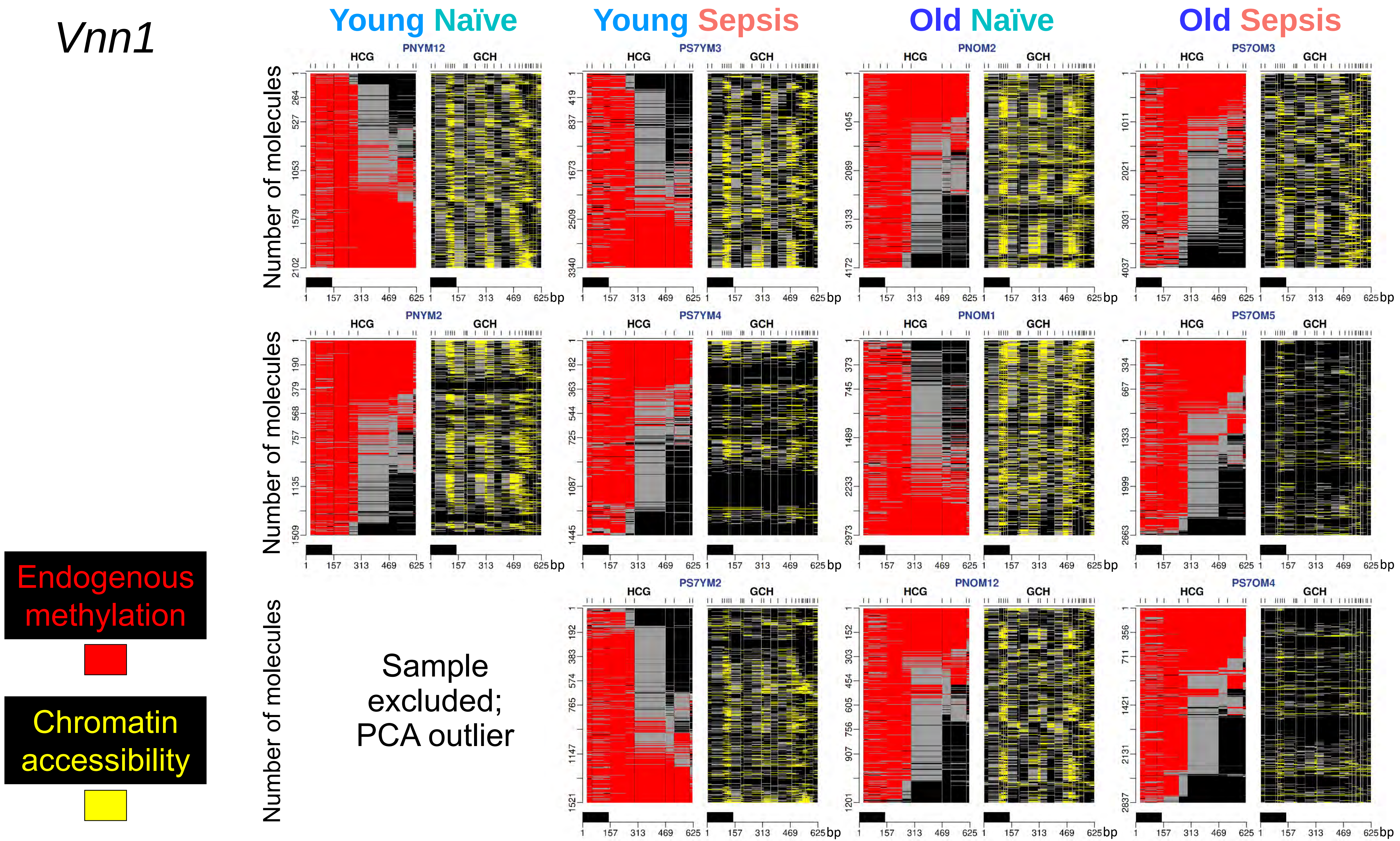

Vnn1
Young Naïve
Young Sepsis
Old Naïve
Old Sepsis
PNYM12
PS7YM3
PNOM2
PS7OM3
PNYM2
PS7YM4
PNOM1
PS7OM5
PS7YM2
PNOM12
PS7OM4
HCG
GCH
Number of molecules
1 157 313 469 625 bp
Sample excluded; PCA outlier
Endogenous methylation
Chromatin accessibility

Serpina1a

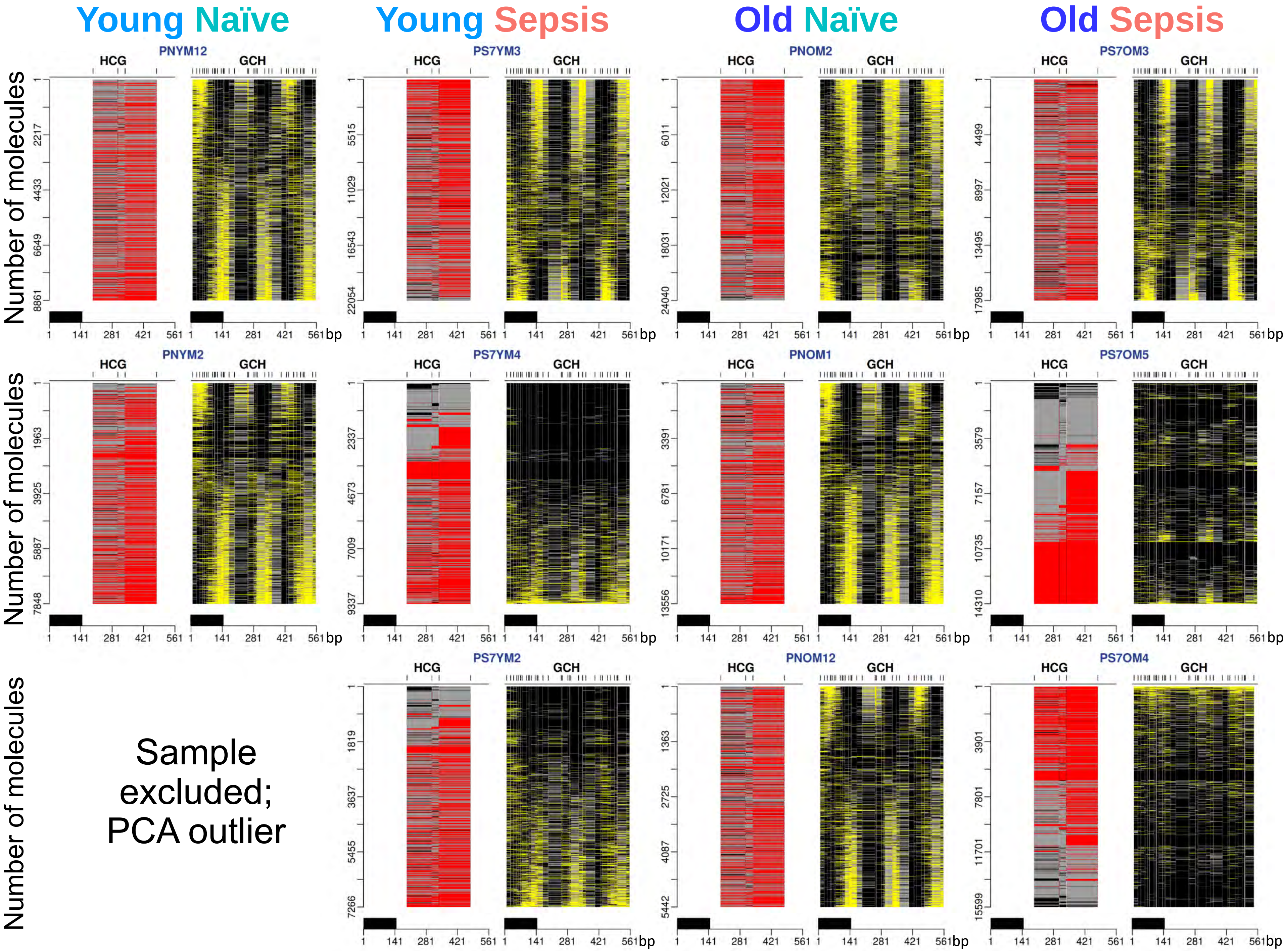

Endogenous methylation

Chromatin accessibility

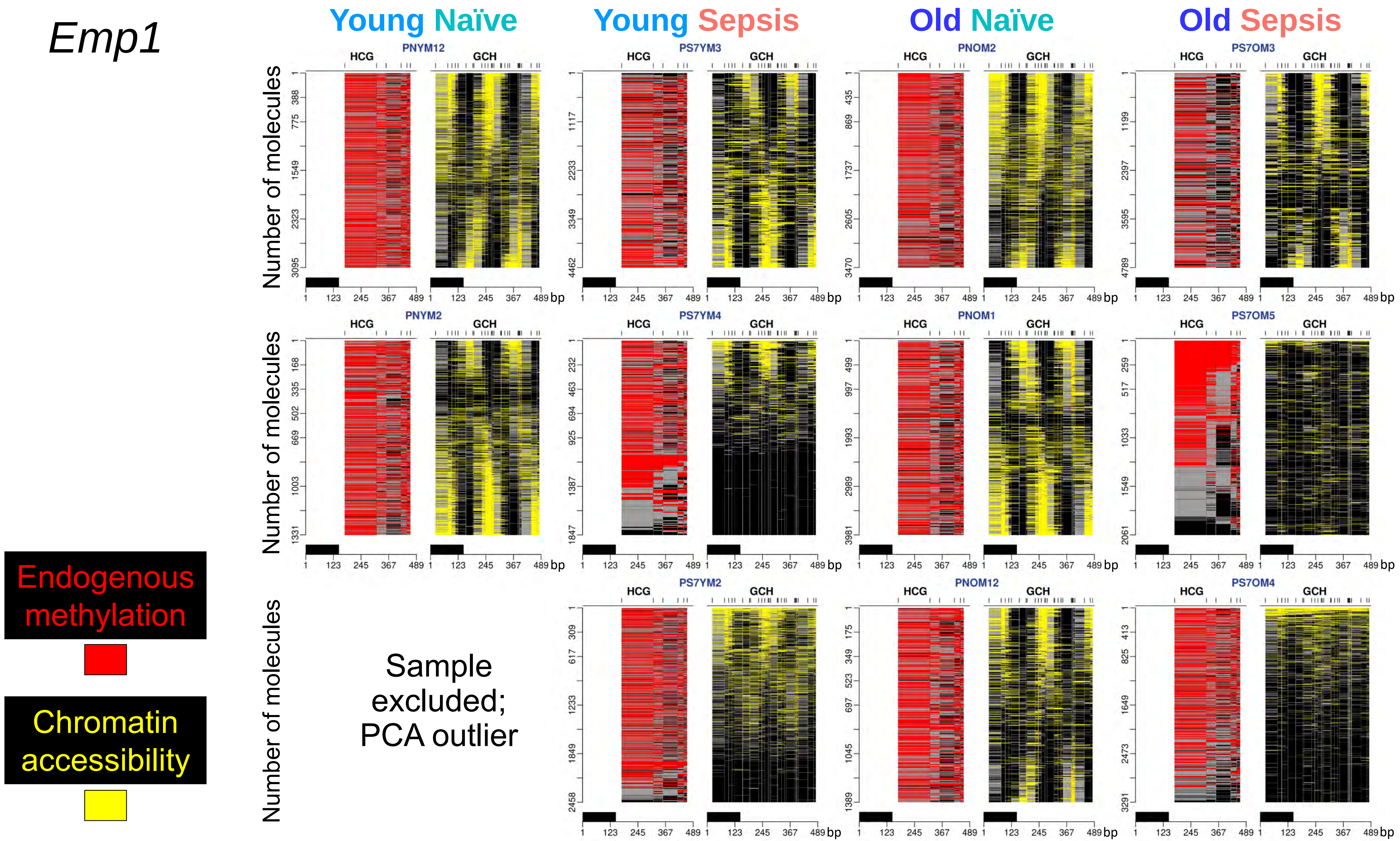

Emp1
Young Naïve
Young Sepsis
Old Naïve
Old Sepsis
PNYM12
PS7YM3
PNOM2
PS7OM3
PNYM2
PS7YM4
PNOM1
PS7OM5
PS7YM2
PNOM12
PS7OM4
HCG
GCH
Number of molecules
bp
Sample excluded; PCA outlier
Endogenous methylation
Chromatin accessibility

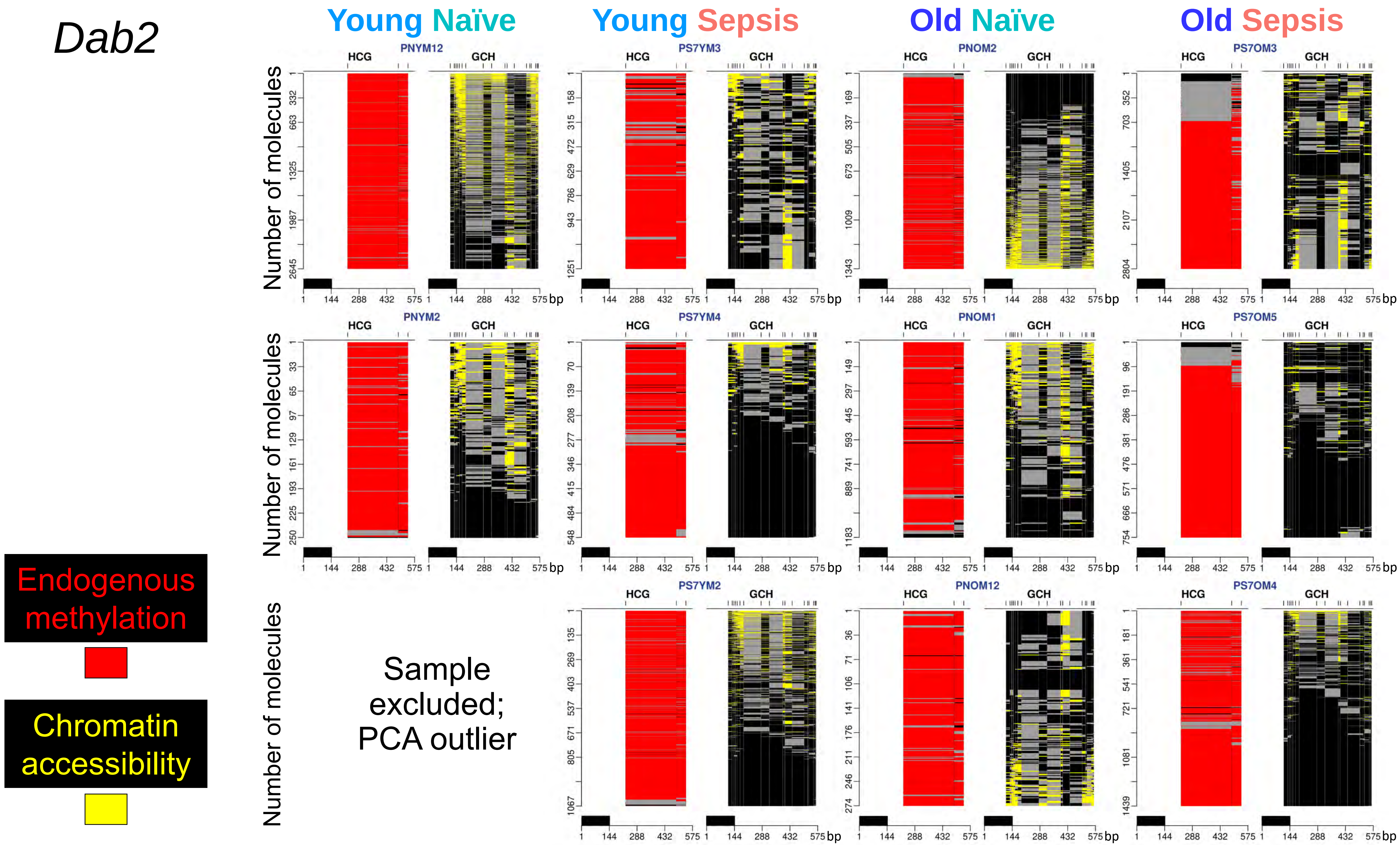

Dab2
Young Naïve
Young Sepsis
Old Naïve
Old Sepsis
PNYM12
PS7YM3
PNOM2
PS7OM3
PNYM2
PS7YM4
PNOM1
PS7OM5
PS7YM2
PNOM12
PS7OM4
HCG
GCH
Number of molecules
bp
1
144
288
432
575
Sample excluded; PCA outlier
Endogenous methylation
Chromatin accessibility

Vsig4

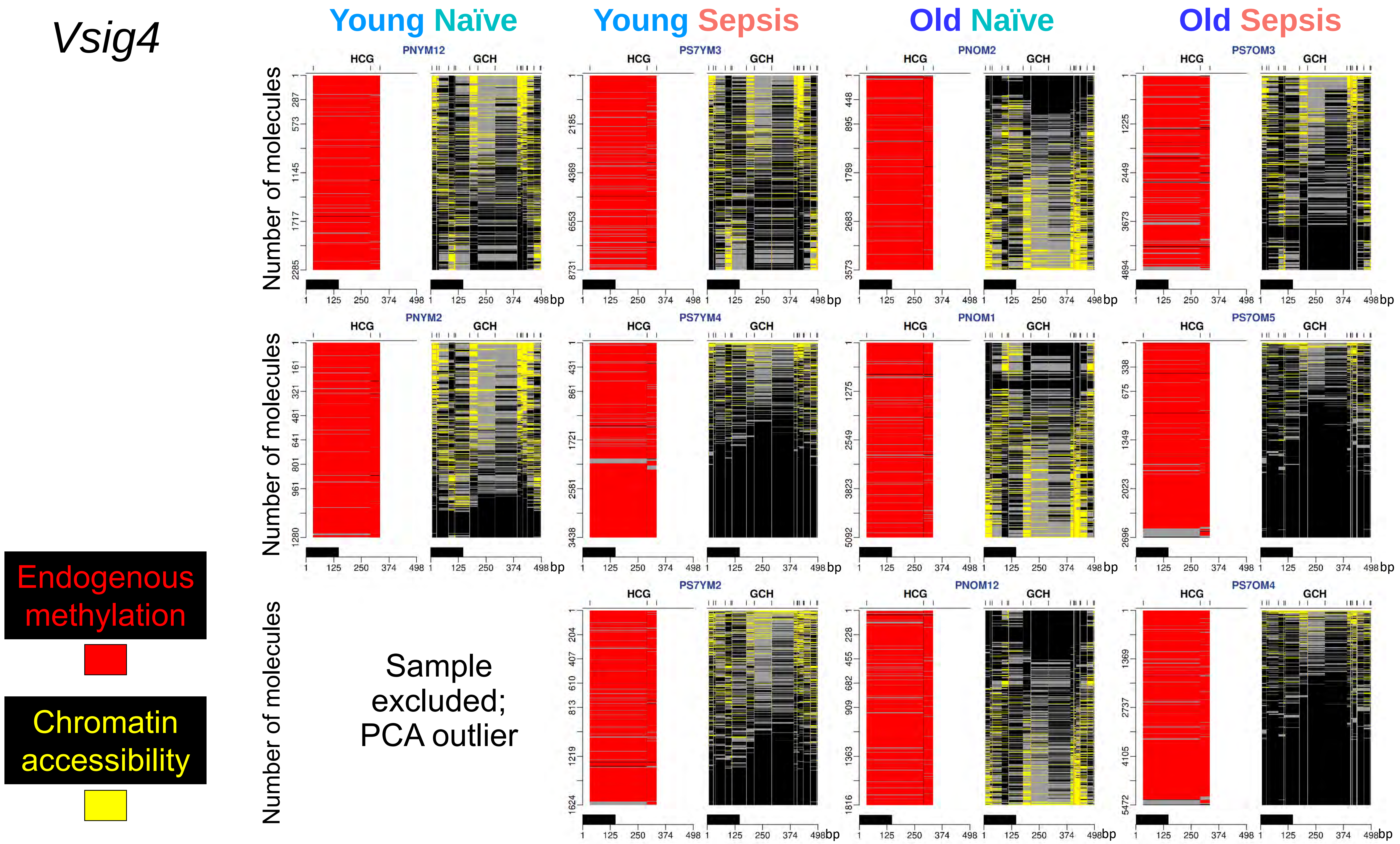
Young Naïve
Young Sepsis
Old Naïve
Old Sepsis
PNYM12
PS7YM3
PNOM2
PS7OM3
PNYM2
PS7YM4
PNOM1
PS7OM5
PS7YM2
PNOM12
PS7OM4
HCG
GCH
Number of molecules
1 125 250 374 498 bp
Sample excluded; PCA outlier
Endogenous methylation
Chromatin accessibility

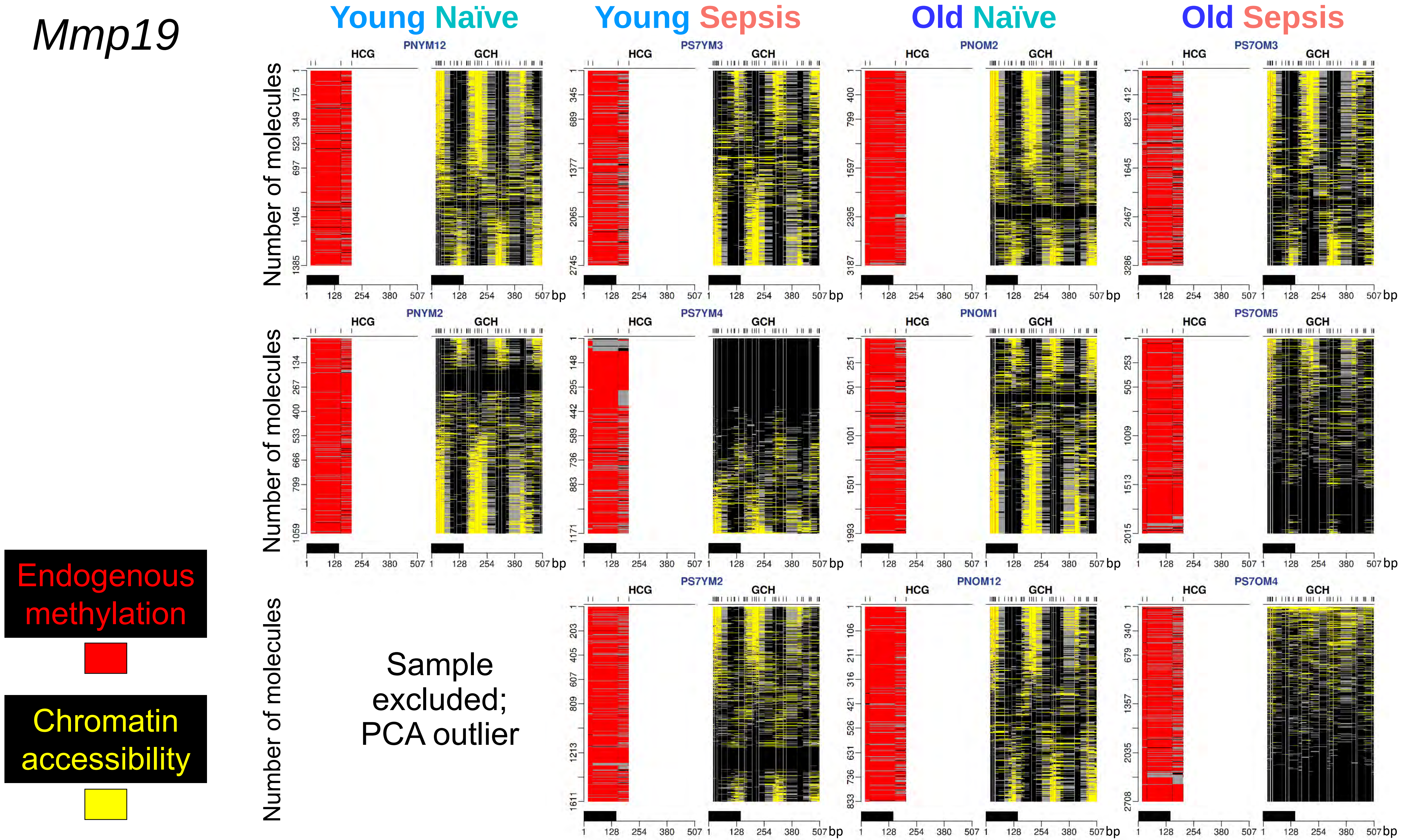
Mmp19
Young Naïve
Young Sepsis
Old Naïve
Old Sepsis
PNYM12
PS7YM3
PNOM2
PS7OM3
PNYM2
PS7YM4
PNOM1
PS7OM5
PS7YM2
PNOM12
PS7OM4
HCG
GCH
Number of molecules
bp
Sample excluded; PCA outlier
Endogenous methylation
Chromatin accessibility

Class 6 promoters: *Serpine1*, *Lgals2*, *Ccl5*, *Fabp7*, *Ambp*, *Kdm6b*, *Fgr*, *Gpnmb*, *Cd9*, *Cxcl3*, *Mmp9*

No NRF formation in response to CLP + DCS across all cohorts:

- Intermediate levels of endogenous CpG methylation (at HCGs)
- Accessibility pattern consistent with disorganized or random nucleosome arrays
- Decreased accessibility in old sepsis samples

Serpine1

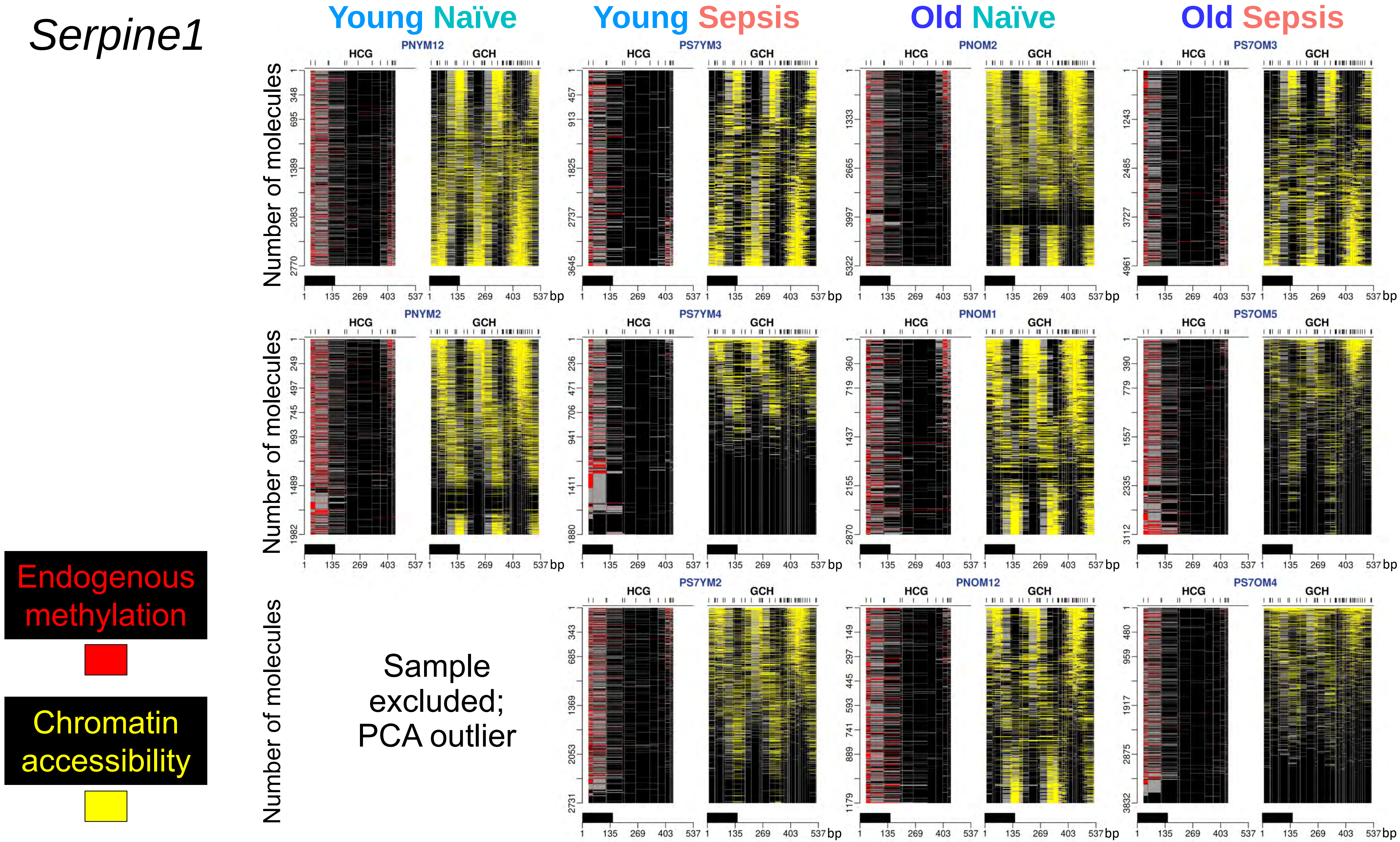

Young Naïve
Young Sepsis
Old Naïve
Old Sepsis
PNYM12
PS7YM3
PNOM2
PS7OM3
PNYM2
PS7YM4
PNOM1
PS7OM5
PS7YM2
PNOM12
PS7OM4
HCG
GCH
Number of molecules
bp
Sample excluded; PCA outlier
Endogenous methylation
Chromatin accessibility

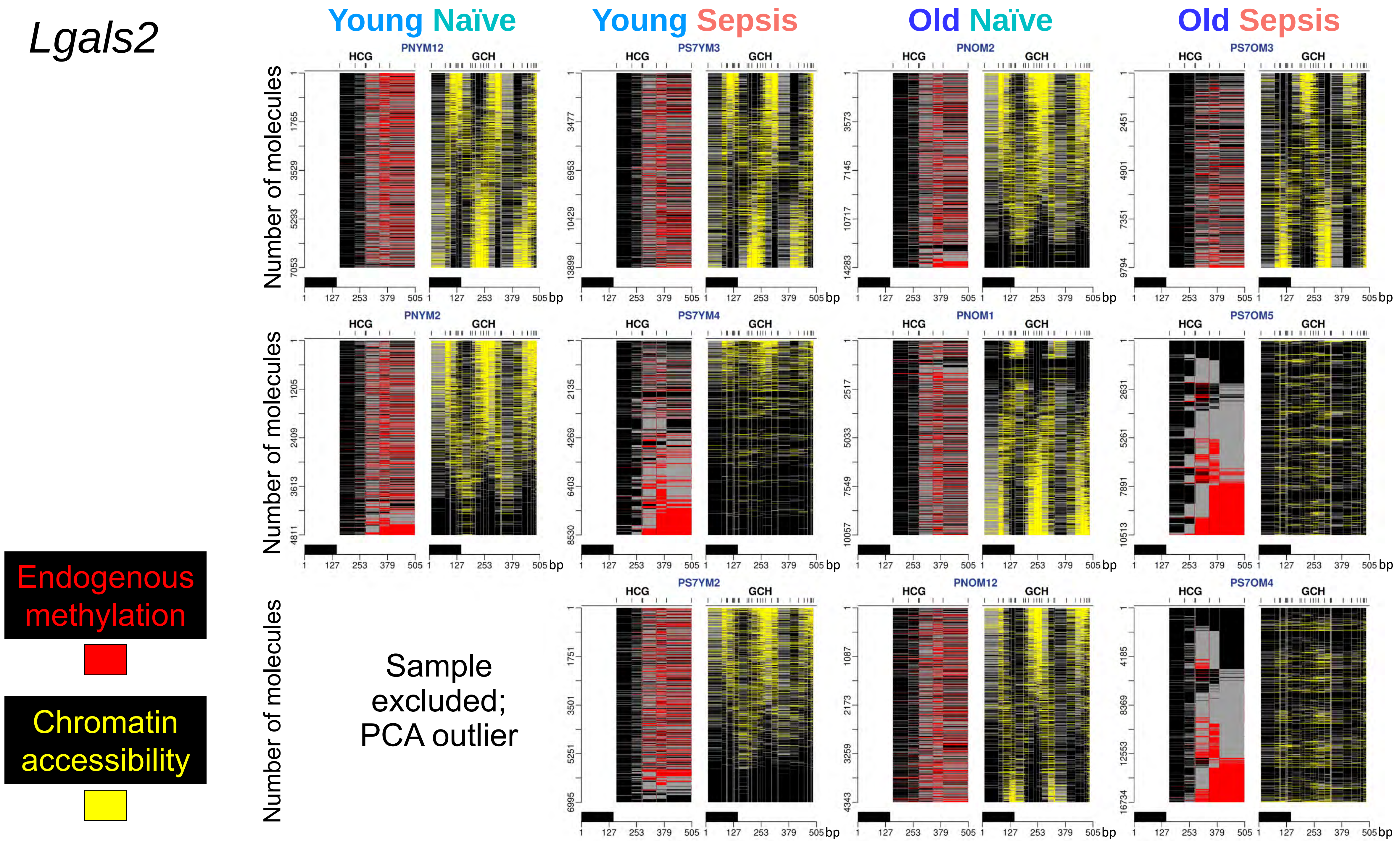

Lgals2
Young Naïve
Young Sepsis
Old Naïve
Old Sepsis
PNYM12
PS7YM3
PNOM2
PS7OM3
PNYM2
PS7YM4
PNOM1
PS7OM5
PS7YM2
PNOM12
PS7OM4
HCG
GCH
Number of molecules
1 127 253 379 505 bp
Sample excluded; PCA outlier
Endogenous methylation
Chromatin accessibility

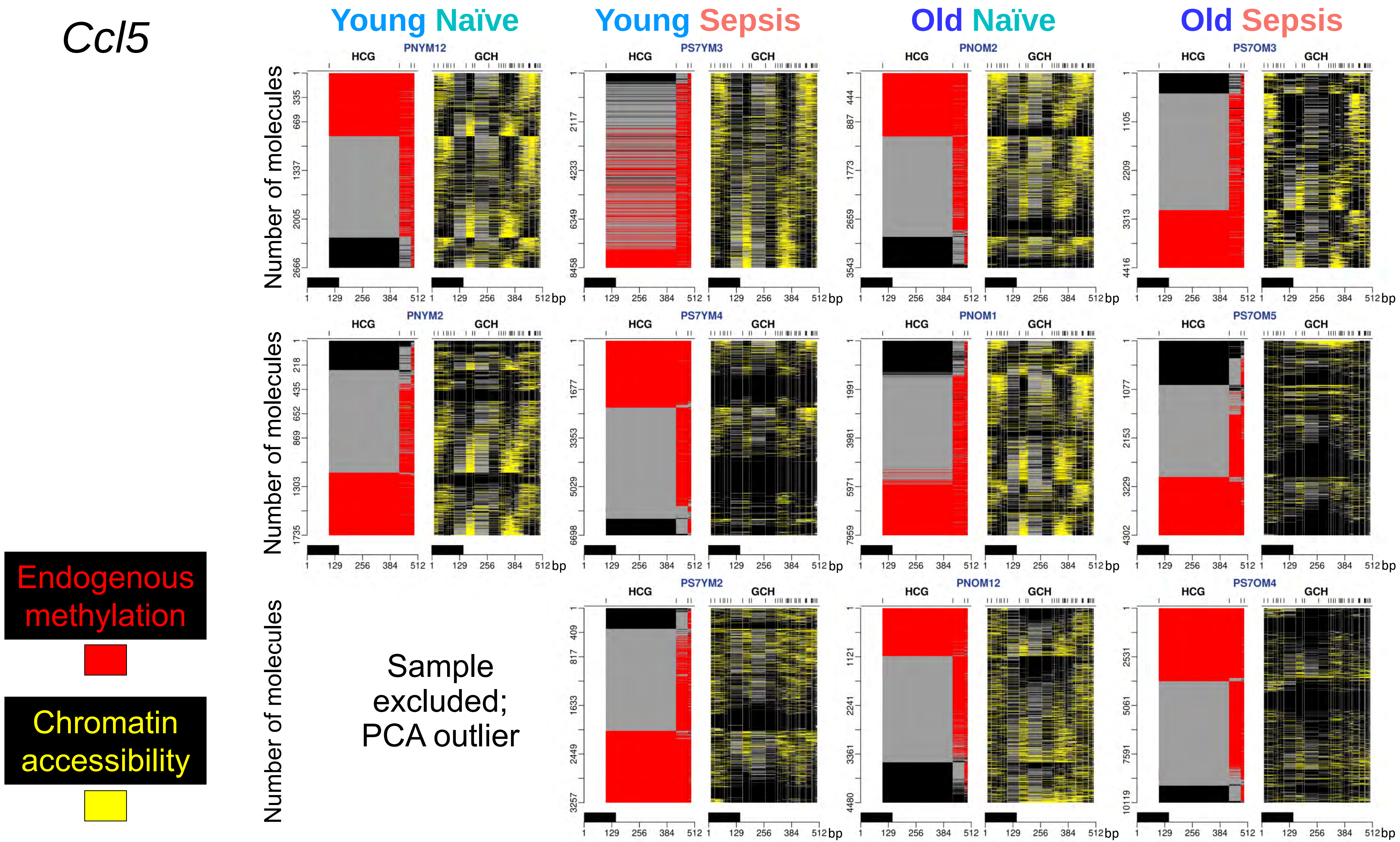
Ccl5
Young Naïve
Young Sepsis
Old Naïve
Old Sepsis
PNYM12
PS7YM3
PNOM2
PS7OM3
PNYM2
PS7YM4
PNOM1
PS7OM5
PS7YM2
PNOM12
PS7OM4
HCG
GCH
Number of molecules
1
129
256
384
512
bp
Sample excluded; PCA outlier
Endogenous methylation
Chromatin accessibility

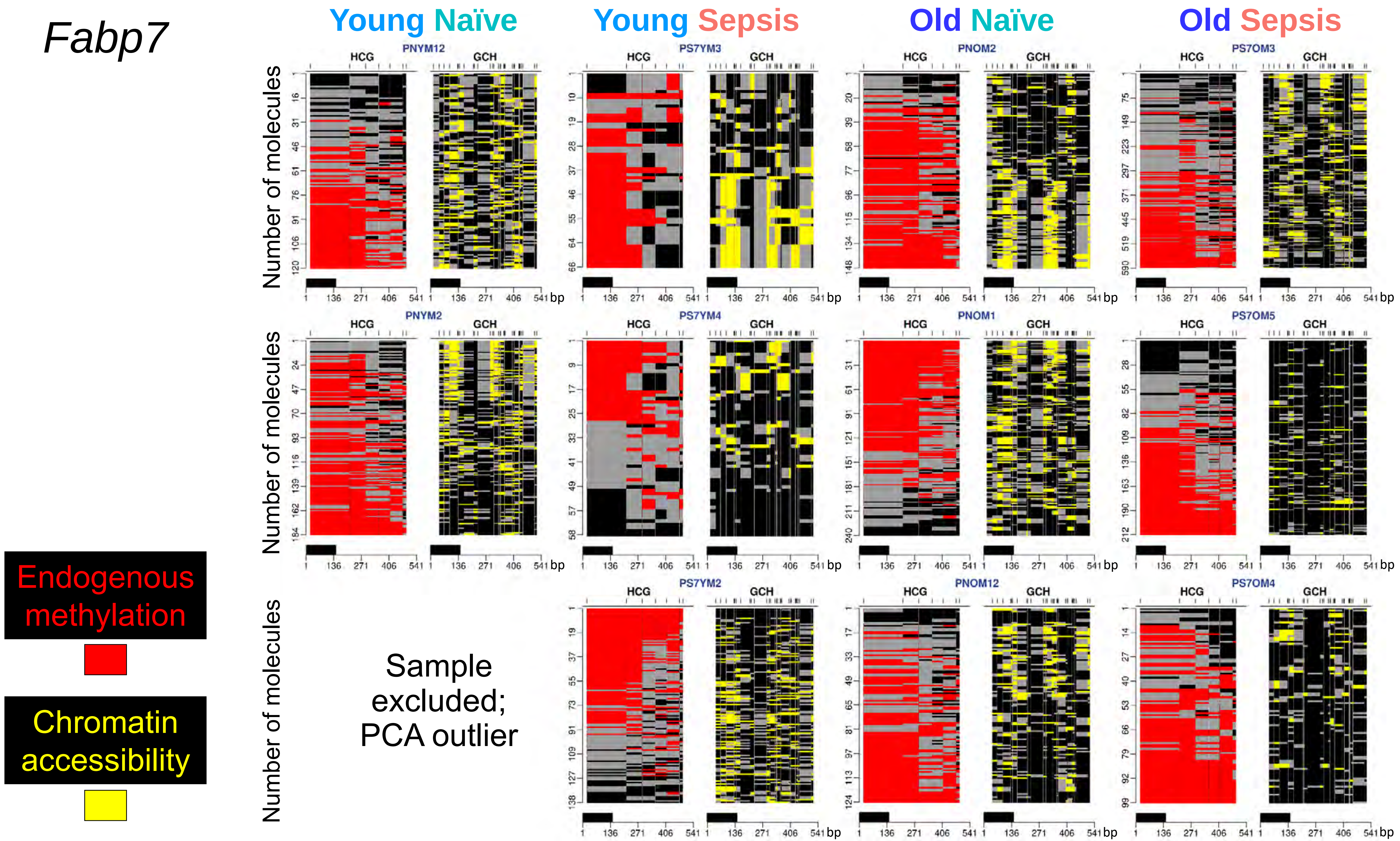

Fabp7
Young Naïve
Young Sepsis
Old Naïve
Old Sepsis
PNYM12
PS7YM3
PNOM2
PS7OM3
PNYM2
PS7YM4
PNOM1
PS7OM5
PS7YM2
PNOM12
PS7OM4
HCG
GCH
Number of molecules
bp
Sample excluded; PCA outlier
Endogenous methylation
Chromatin accessibility

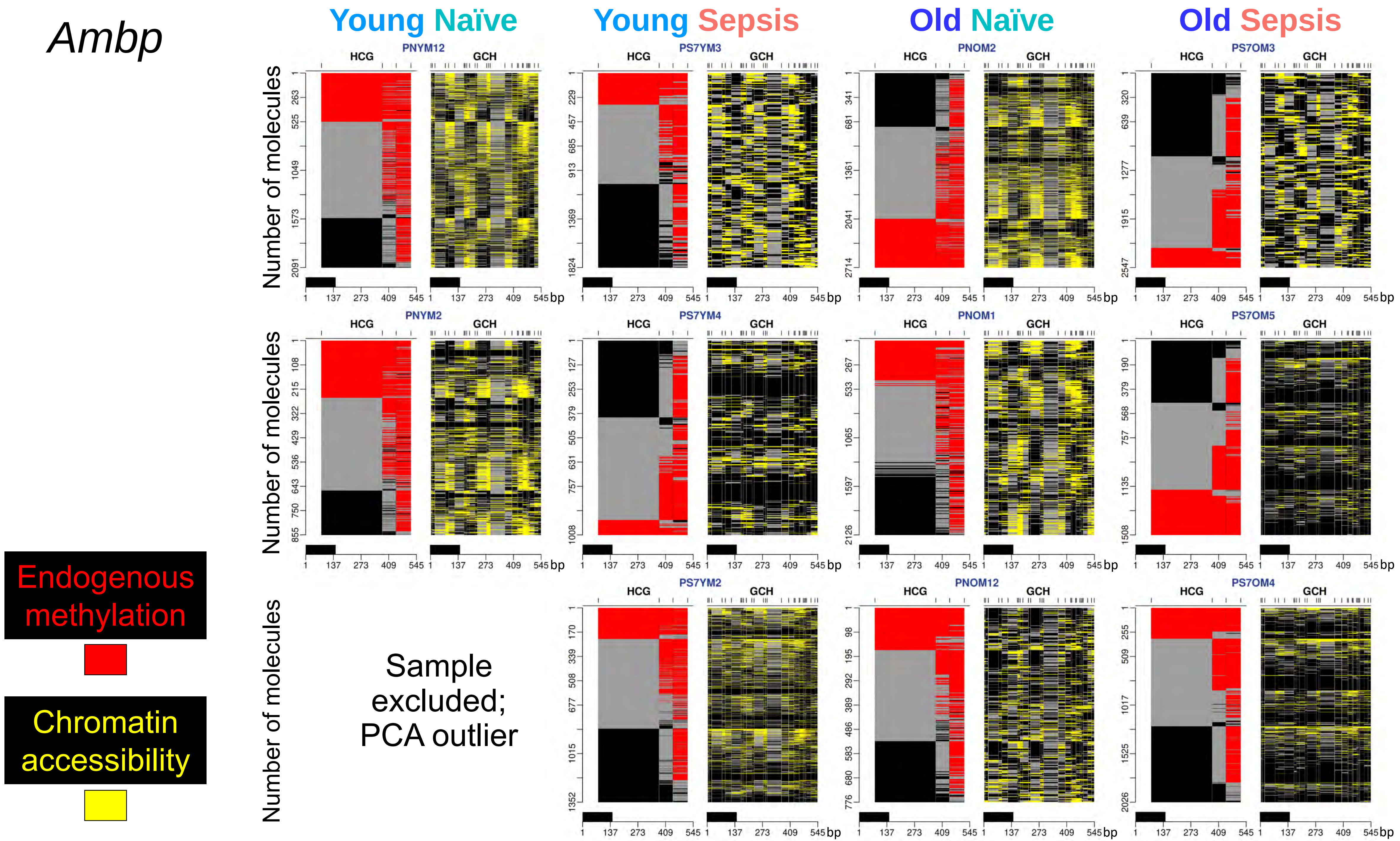

Ambp
Young Naïve
Young Sepsis
Old Naïve
Old Sepsis
PNYM12
PS7YM3
PNOM2
PS7OM3
PNYM2
PS7YM4
PNOM1
PS7OM5
PS7YM2
PNOM12
PS7OM4
HCG
GCH
Number of molecules
bp
Sample excluded; PCA outlier
Endogenous methylation
Chromatin accessibility

Kdm6b

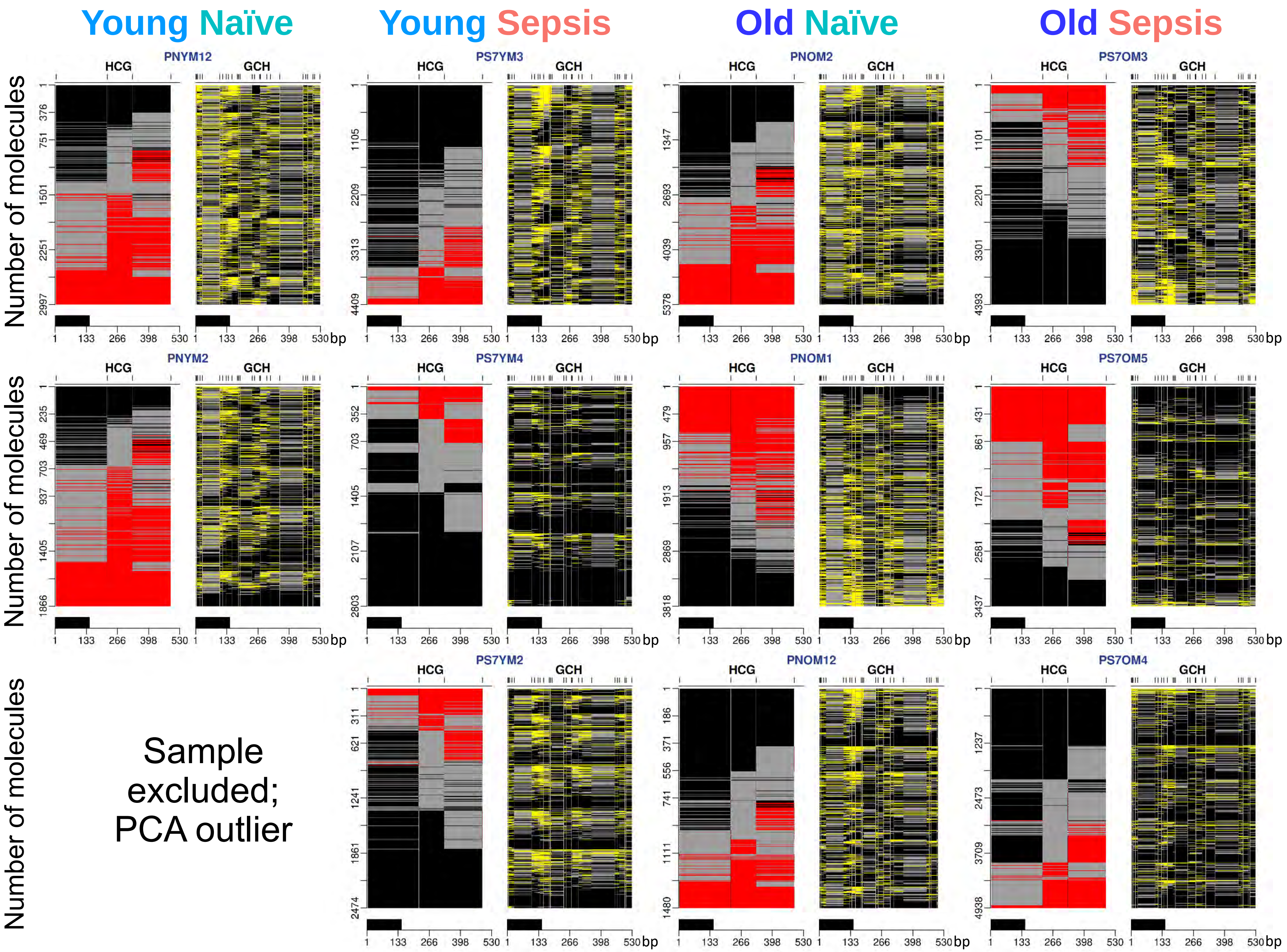

Endogenous methylation

Chromatin accessibility

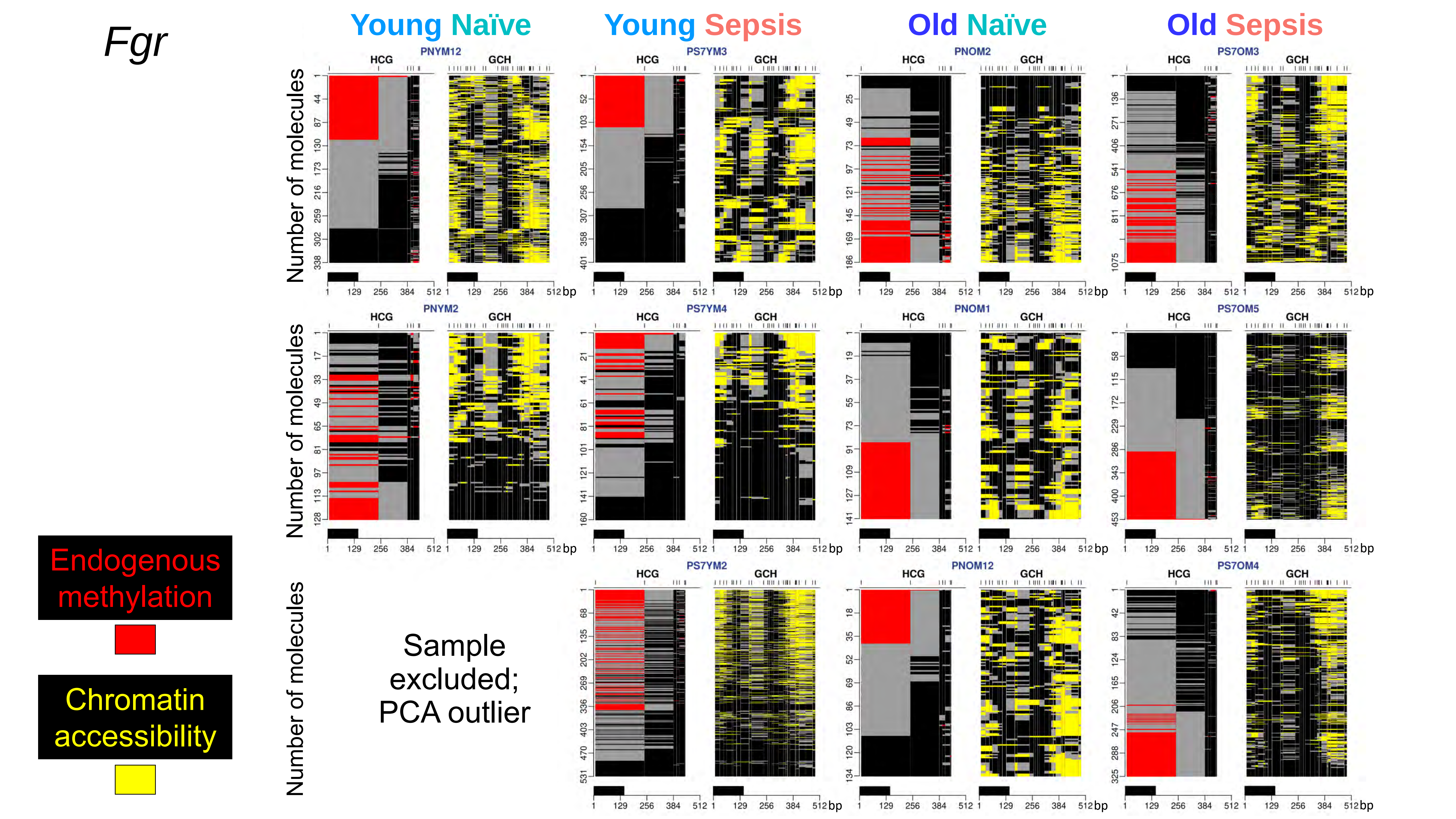
Fgr
Young Naïve
Young Sepsis
Old Naïve
Old Sepsis
PNYM12
PS7YM3
PNOM2
PS7OM3
PNYM2
PS7YM4
PNOM1
PS7OM5
PS7YM2
PNOM12
PS7OM4
HCG
GCH
Number of molecules
1 129 256 384 512 bp
Sample excluded; PCA outlier
Endogenous methylation
Chromatin accessibility

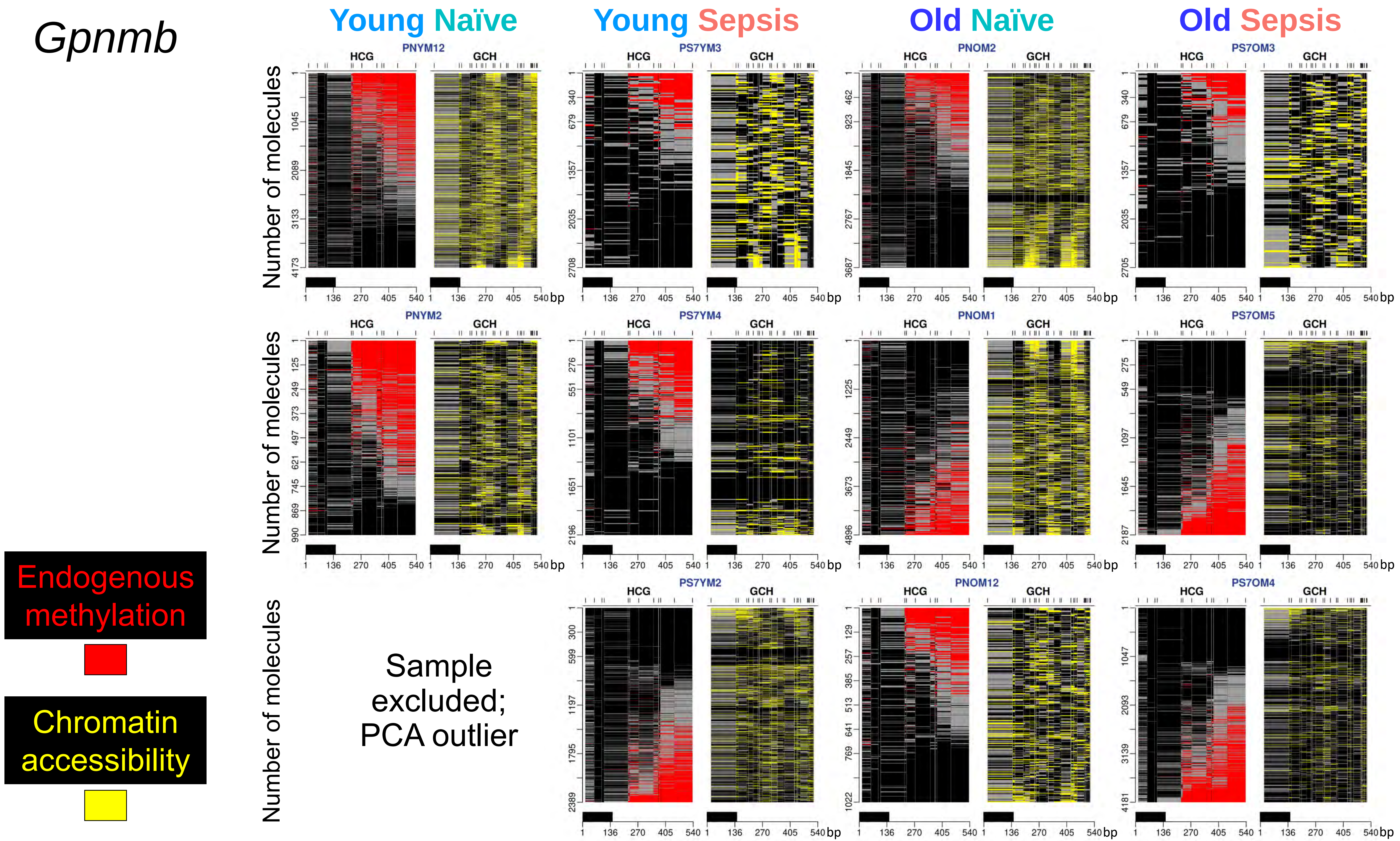

Gpnmb
Young Naïve
Young Sepsis
Old Naïve
Old Sepsis
PNYM12
PS7YM3
PNOM2
PS7OM3
PNYM2
PS7YM4
PNOM1
PS7OM5
PS7YM2
PNOM12
PS7OM4
HCG
GCH
Number of molecules
bp
Sample excluded; PCA outlier
Endogenous methylation
Chromatin accessibility

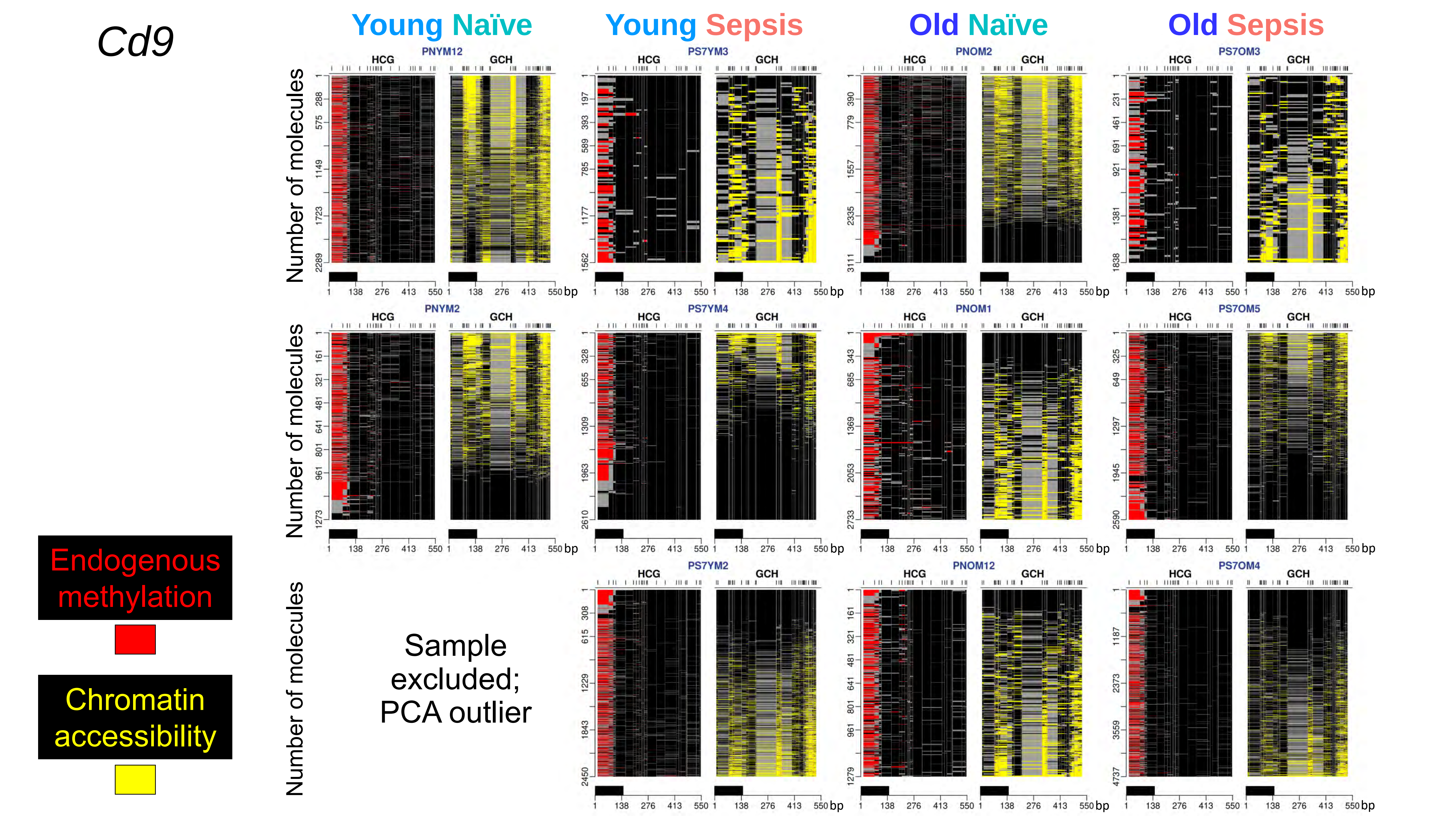
Cd9
Young Naïve
Young Sepsis
Old Naïve
Old Sepsis
PNYM12
PS7YM3
PNOM2
PS7OM3
PNYM2
PS7YM4
PNOM1
PS7OM5
PS7YM2
PNOM12
PS7OM4
HCG
GCH
Number of molecules
1 138 276 413 550 bp
Sample excluded; PCA outlier
Endogenous methylation
Chromatin accessibility

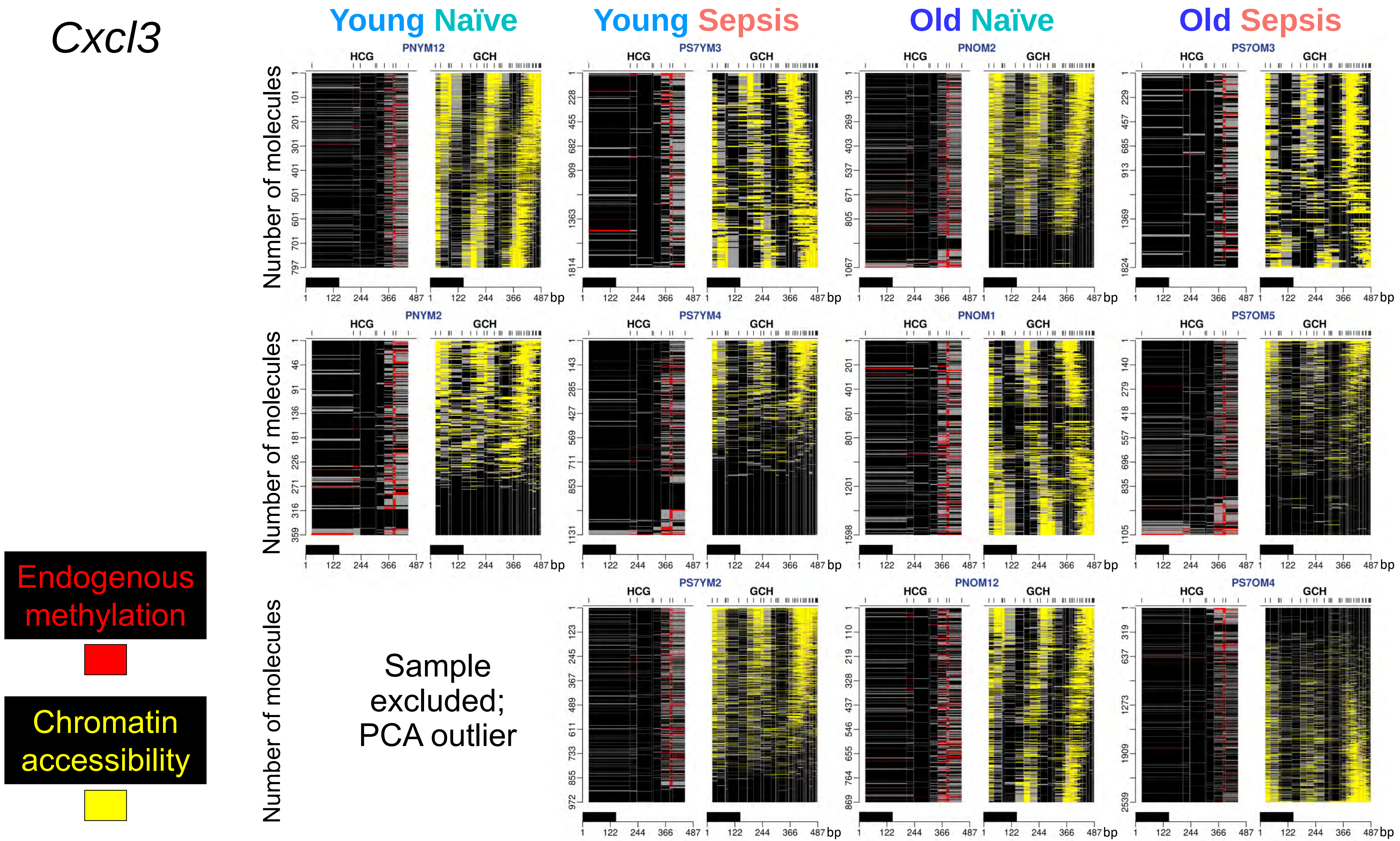

Cxcl3
Young Naïve
Young Sepsis
Old Naïve
Old Sepsis
PNYM12
PS7YM3
PNOM2
PS7OM3
PNYM2
PS7YM4
PNOM1
PS7OM5
PS7YM2
PNOM12
PS7OM4
HCG
GCH
Number of molecules
bp
Sample excluded; PCA outlier
Endogenous methylation
Chromatin accessibility

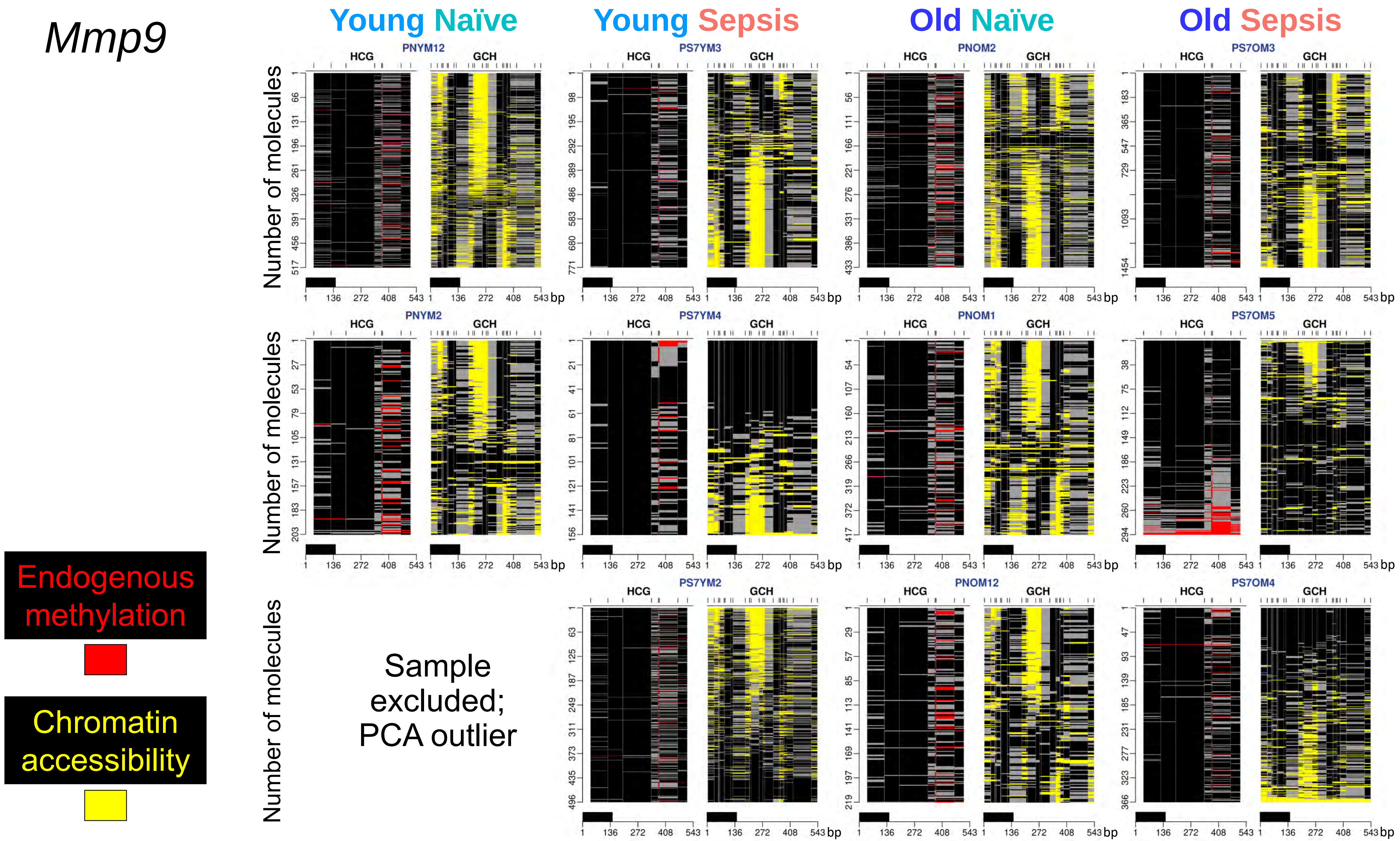
Mmp9
Young Naïve
Young Sepsis
Old Naïve
Old Sepsis
PNYM12
PS7YM3
PNOM2
PS7OM3
PNYM2
PS7YM4
PNOM1
PS7OM5
PS7YM2
PNOM12
PS7OM4
HCG
GCH
Number of molecules
1 136 272 408 543 bp
Sample excluded; PCA outlier
Endogenous methylation
Chromatin accessibility

Class 7 promoters: *Vcan*, *Tmem176*, *Stab1*, *Plac8*, *Il10*, *Gprc5b*, *Htr2a*, *S100a10*, *Car4*, *Mt2*, *Igkv4-69*, *Ccl17*, *Igkv12-44*, *Igkv12-46*

No NRF formation in response to CLP + DCS across all cohorts:

- Low levels of endogenous CpG methylation (at HCGs) or < 3 HCG sites
- Accessibility pattern consistent with disorganized or random nucleosome arrays
- Decreased accessibility in old sepsis cohort compared to other cohorts

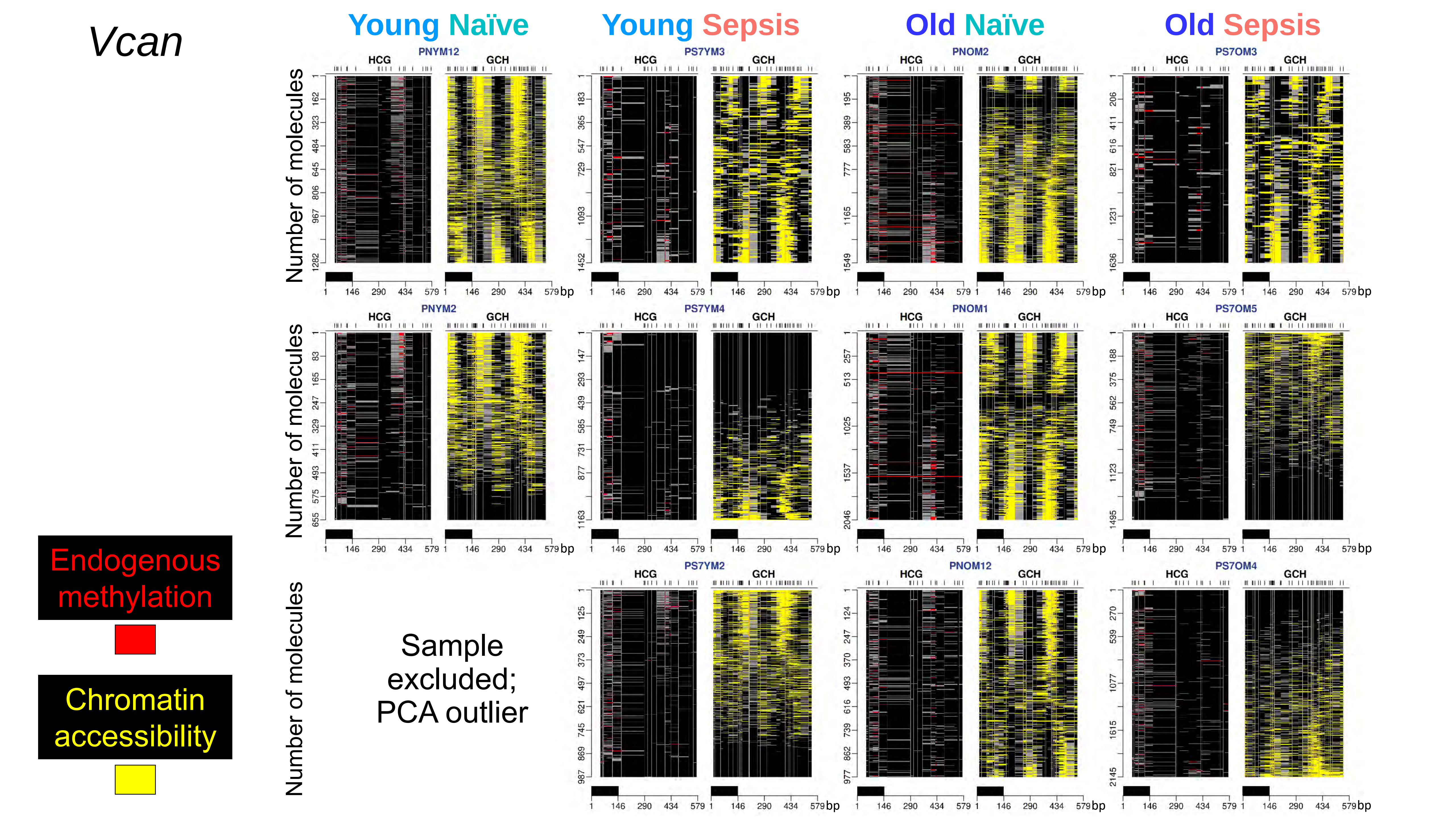
Vcan
Young Naïve
Young Sepsis
Old Naïve
Old Sepsis
PNYM12
PS7YM3
PNOM2
PS7OM3
PNYM2
PS7YM4
PNOM1
PS7OM5
PS7YM2
PNOM12
PS7OM4
HCG
GCH
Number of molecules
bp
Sample excluded; PCA outlier
Endogenous methylation
Chromatin accessibility

Tmem176

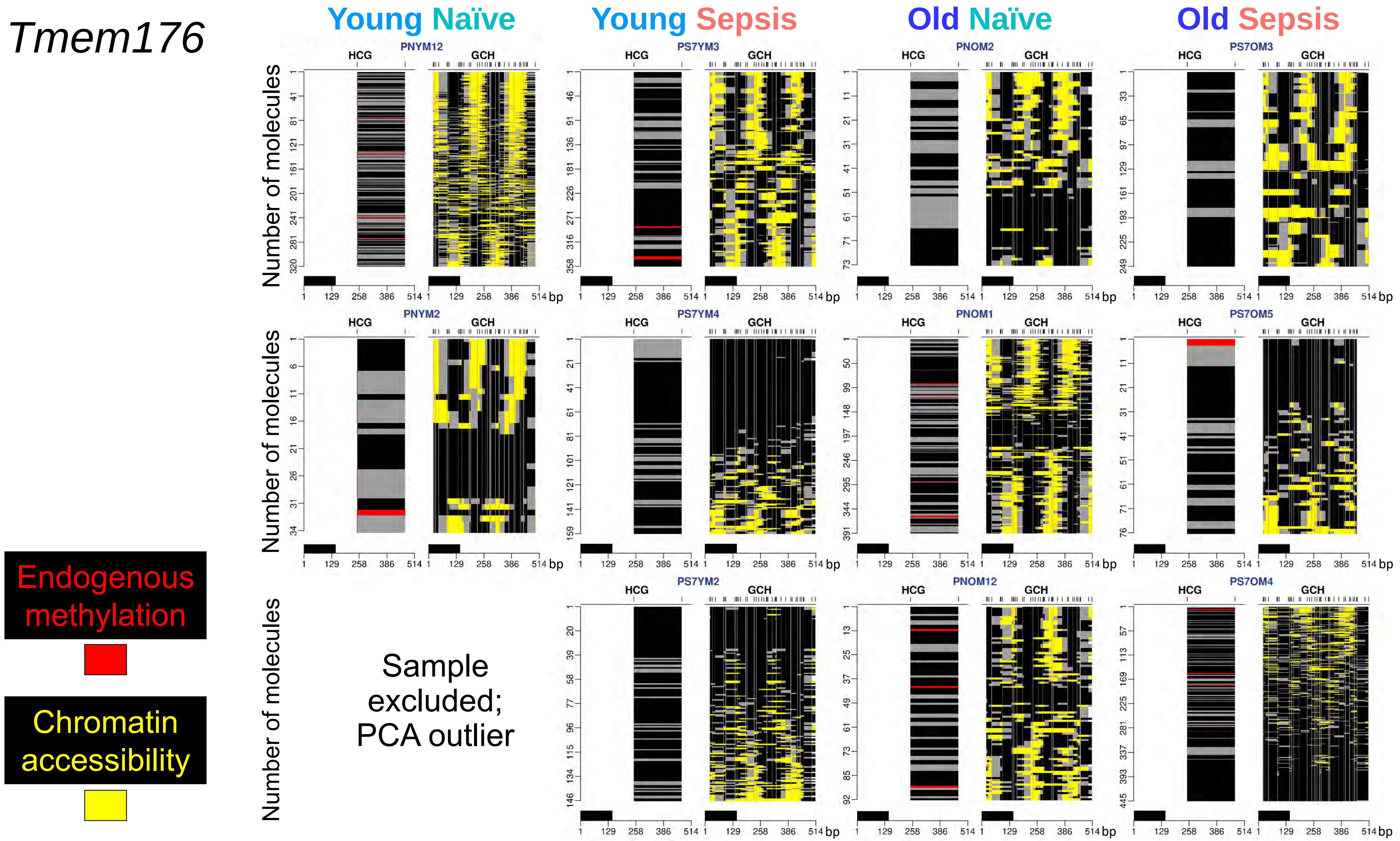

Young Naïve
Young Sepsis
Old Naïve
Old Sepsis
PNYM12
PS7YM3
PNOM2
PS7OM3
PNYM2
PS7YM4
PNOM1
PS7OM5
PS7YM2
PNOM12
PS7OM4
HCG
GCH
Number of molecules
1 129 258 386 514 bp
Sample excluded; PCA outlier
Endogenous methylation
Chromatin accessibility

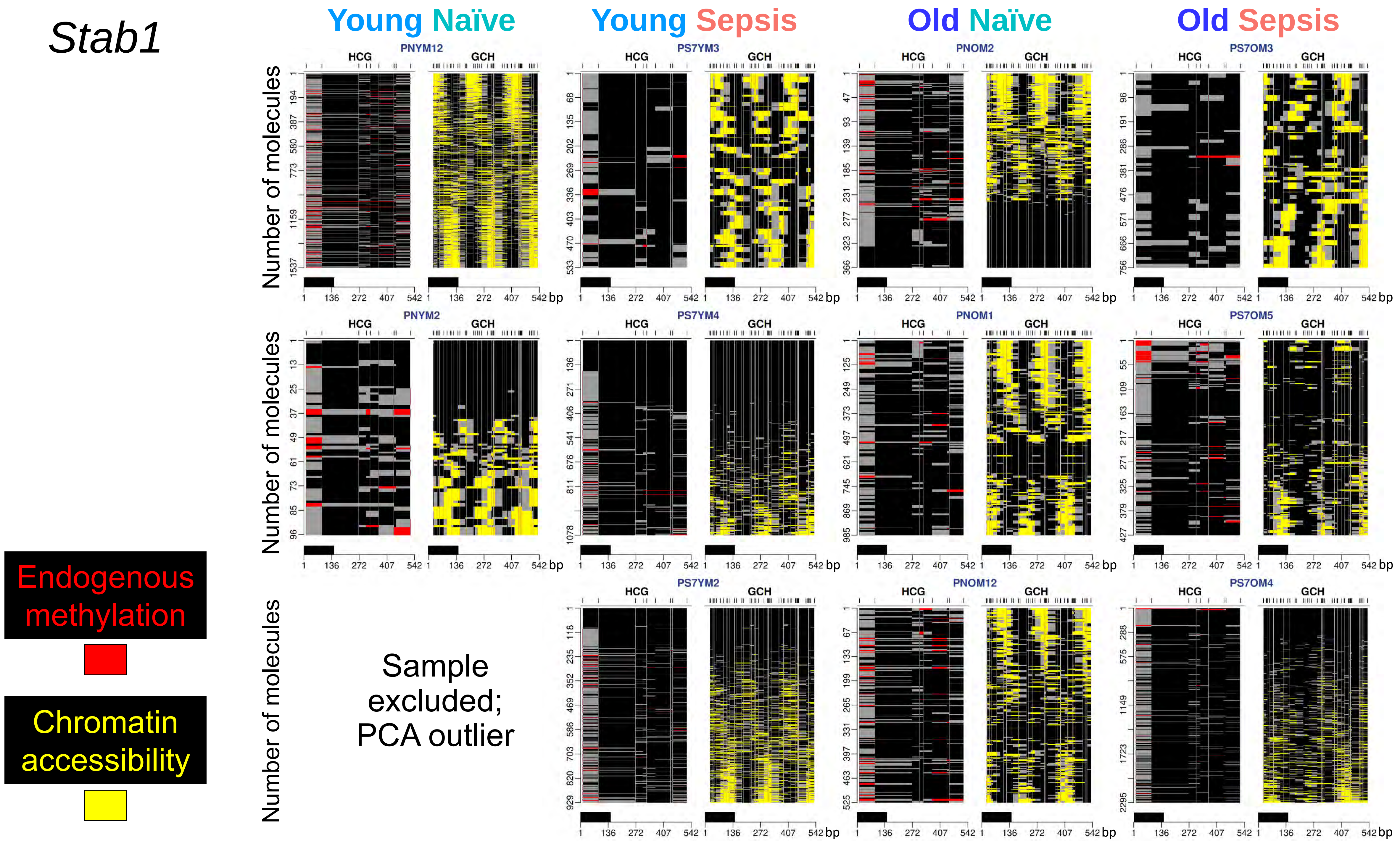

Stab1
Young Naïve
Young Sepsis
Old Naïve
Old Sepsis
PNYM12
PS7YM3
PNOM2
PS7OM3
PNYM2
PS7YM4
PNOM1
PS7OM5
PS7YM2
PNOM12
PS7OM4
HCG
GCH
Number of molecules
bp
Sample excluded; PCA outlier
Endogenous methylation
Chromatin accessibility

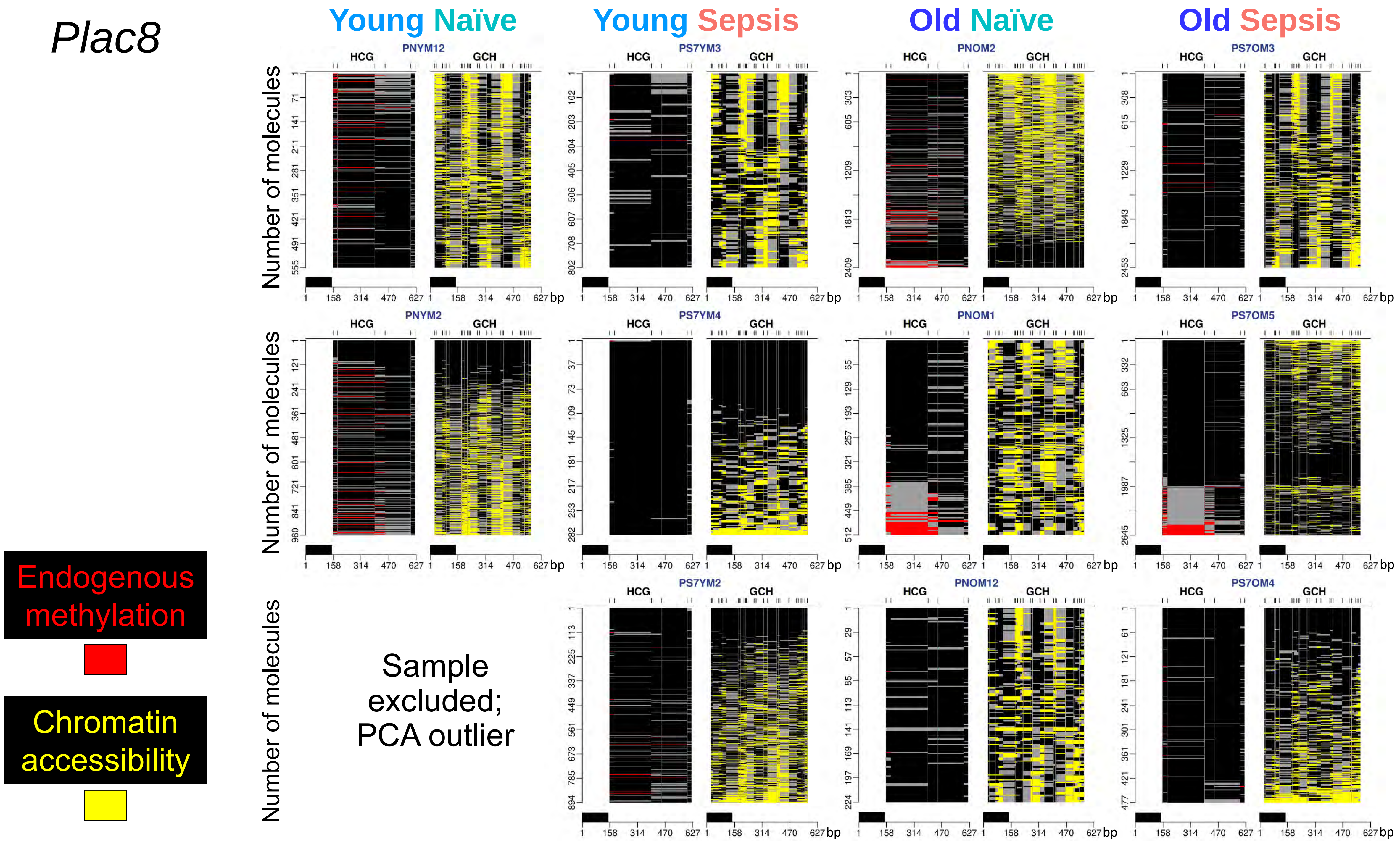

Plac8
Young Naïve
Young Sepsis
Old Naïve
Old Sepsis
PNYM12
PS7YM3
PNOM2
PS7OM3
PNYM2
PS7YM4
PNOM1
PS7OM5
PS7YM2
PNOM12
PS7OM4
HCG
GCH
Number of molecules
1 158 314 470 627 bp
Sample excluded; PCA outlier
Endogenous methylation
Chromatin accessibility

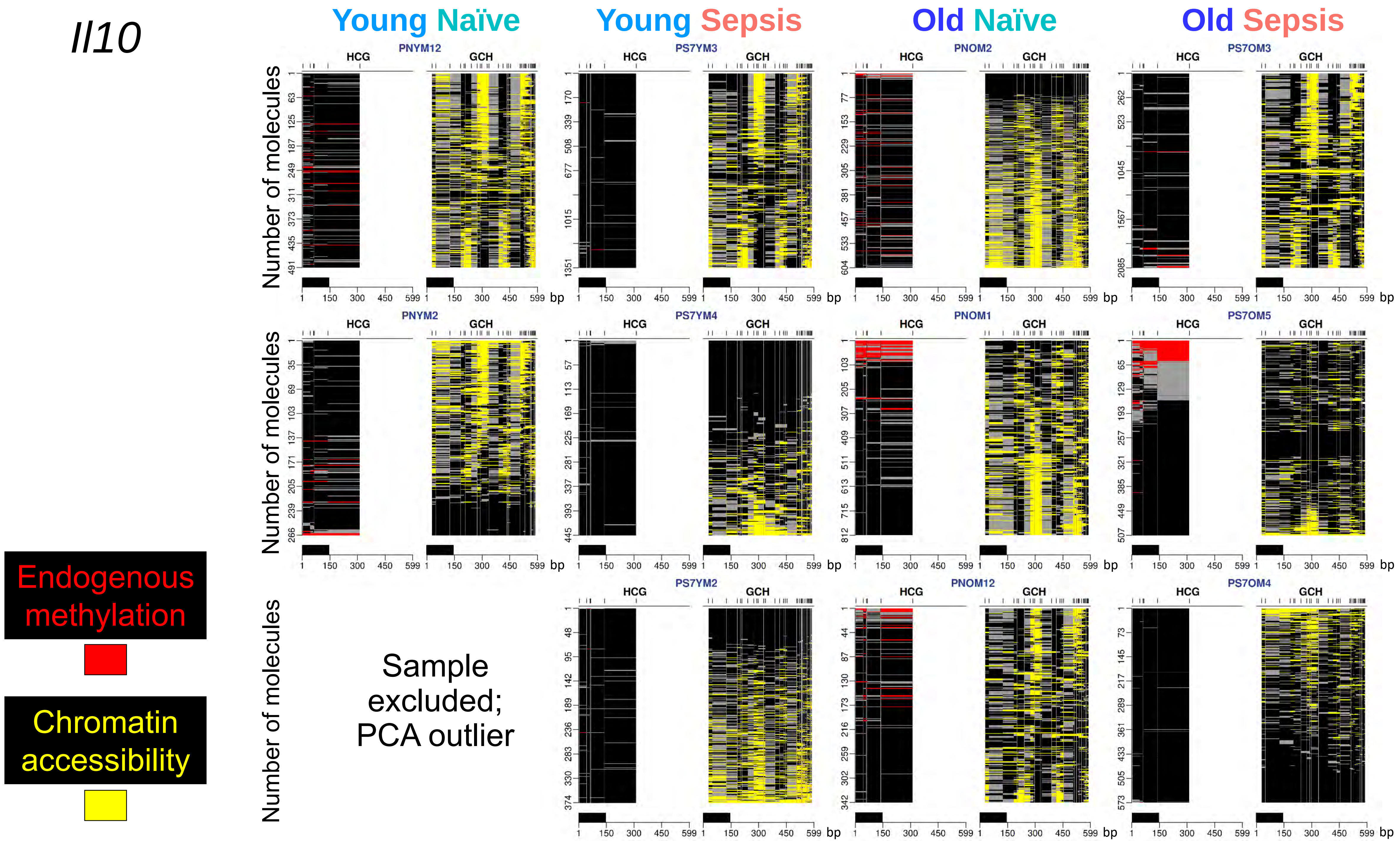

Il10
Young Naïve
Young Sepsis
Old Naïve
Old Sepsis
PNYM12
PS7YM3
PNOM2
PS7OM3
PNYM2
PS7YM4
PNOM1
PS7OM5
PS7YM2
PNOM12
PS7OM4
HCG
GCH
Number of molecules
bp
Sample excluded; PCA outlier
Endogenous methylation
Chromatin accessibility

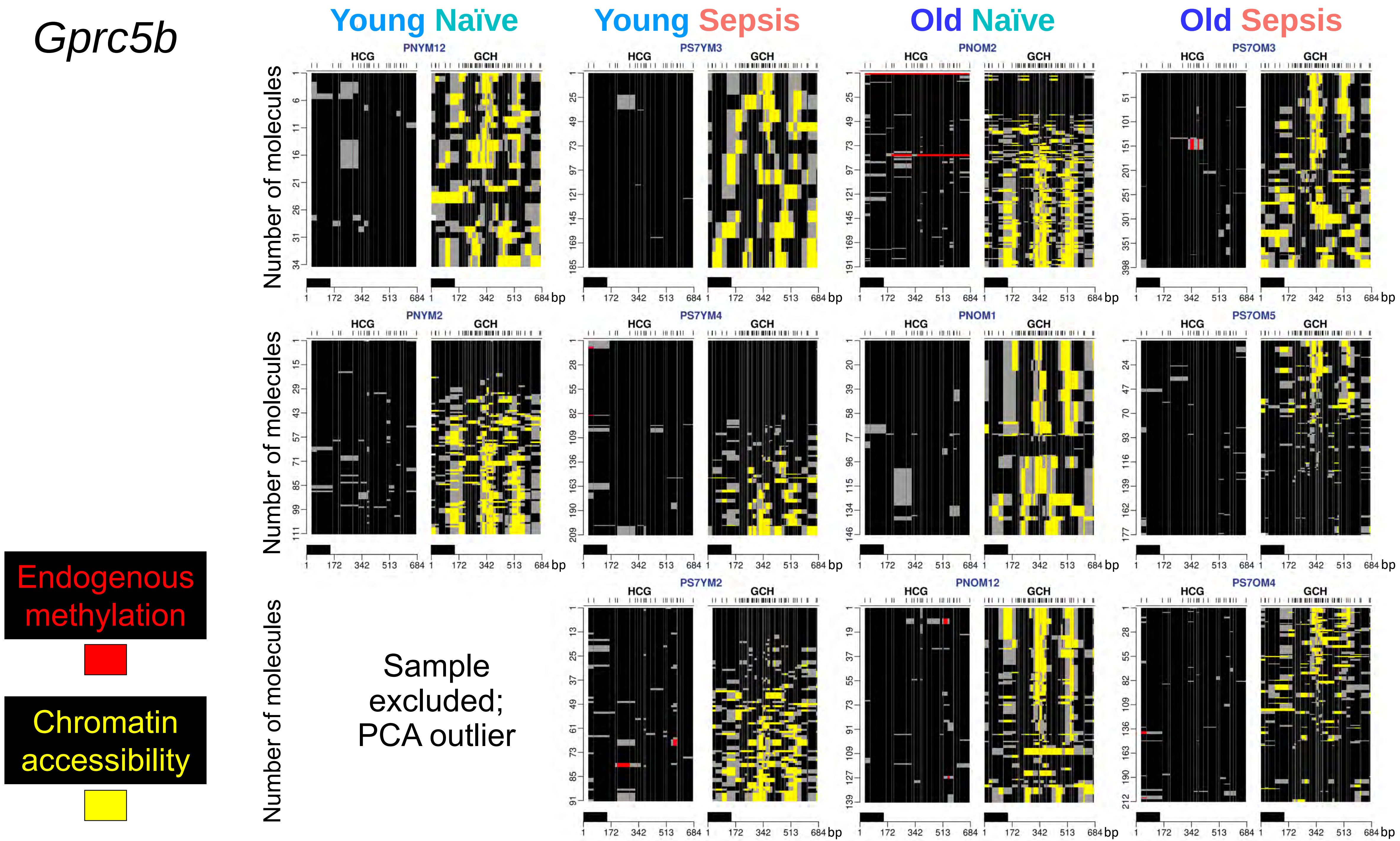
Gprc5b
Young Naïve
Young Sepsis
Old Naïve
Old Sepsis
PNYM12
PS7YM3
PNOM2
PS7OM3
PNYM2
PS7YM4
PNOM1
PS7OM5
PS7YM2
PNOM12
PS7OM4
Sample excluded; PCA outlier
Endogenous methylation
Chromatin accessibility

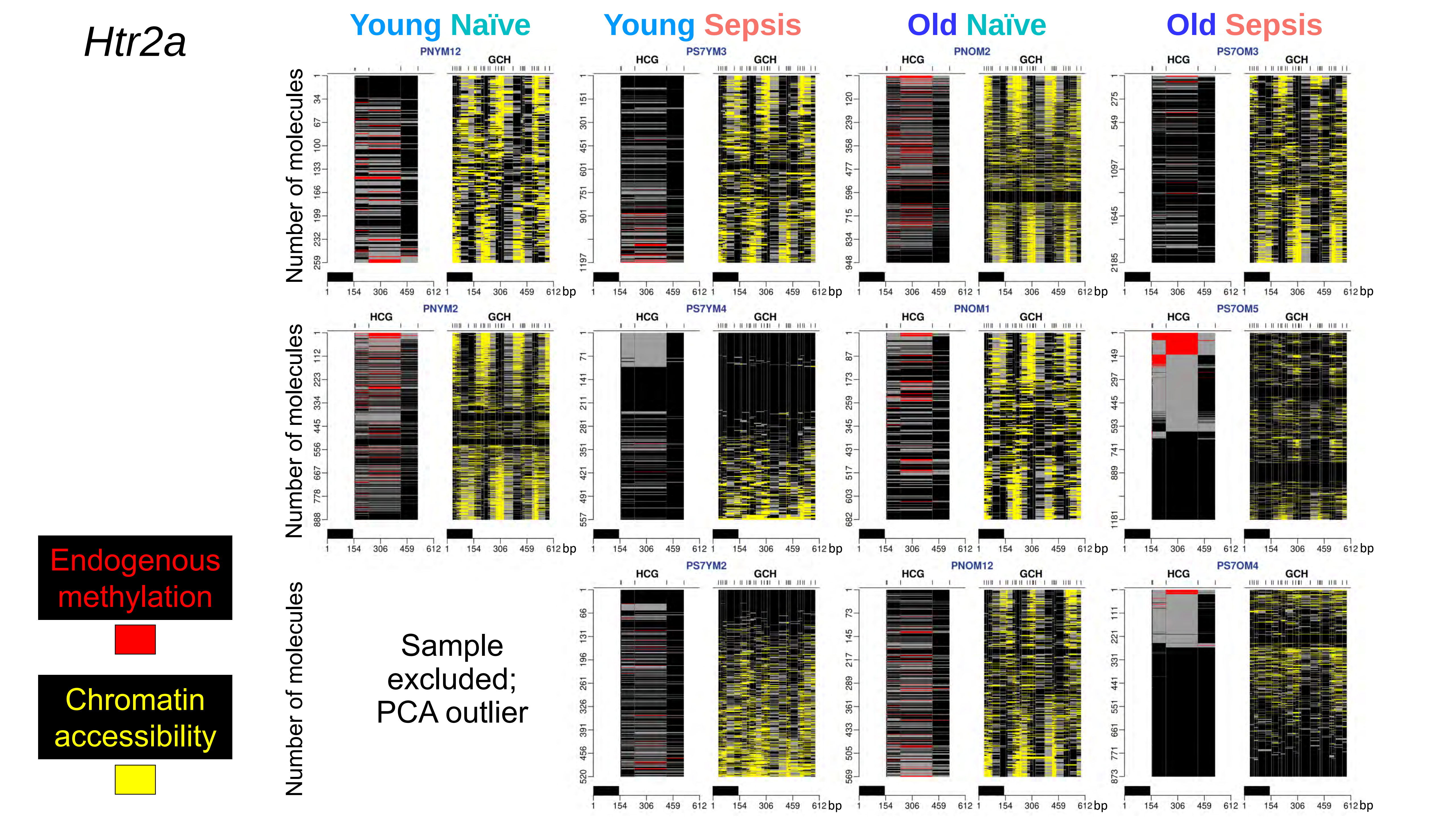
Htr2a
Young Naïve
Young Sepsis
Old Naïve
Old Sepsis
PNYM12
PS7YM3
PNOM2
PS7OM3
PNYM2
PS7YM4
PNOM1
PS7OM5
PS7YM2
PNOM12
PS7OM4
HCG
GCH
Number of molecules
bp
Sample excluded; PCA outlier
Endogenous methylation
Chromatin accessibility

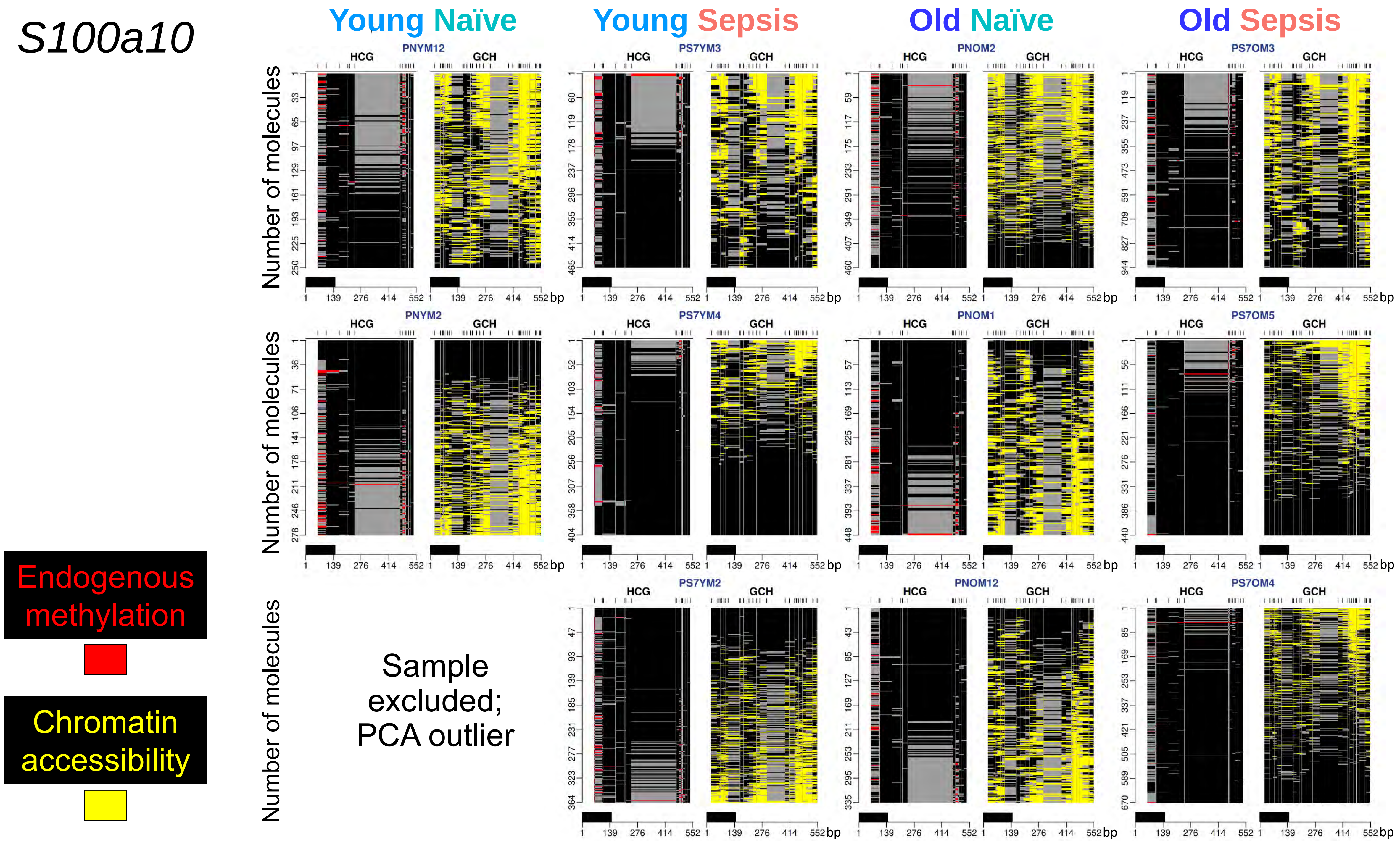
S100a10
Young Naïve
Young Sepsis
Old Naïve
Old Sepsis
PNYM12
PS7YM3
PNOM2
PS7OM3
PNYM2
PS7YM4
PNOM1
PS7OM5
PS7YM2
PNOM12
PS7OM4
HCG
GCH
Number of molecules
1
139
276
414
552
bp
Sample excluded; PCA outlier
Endogenous methylation
Chromatin accessibility

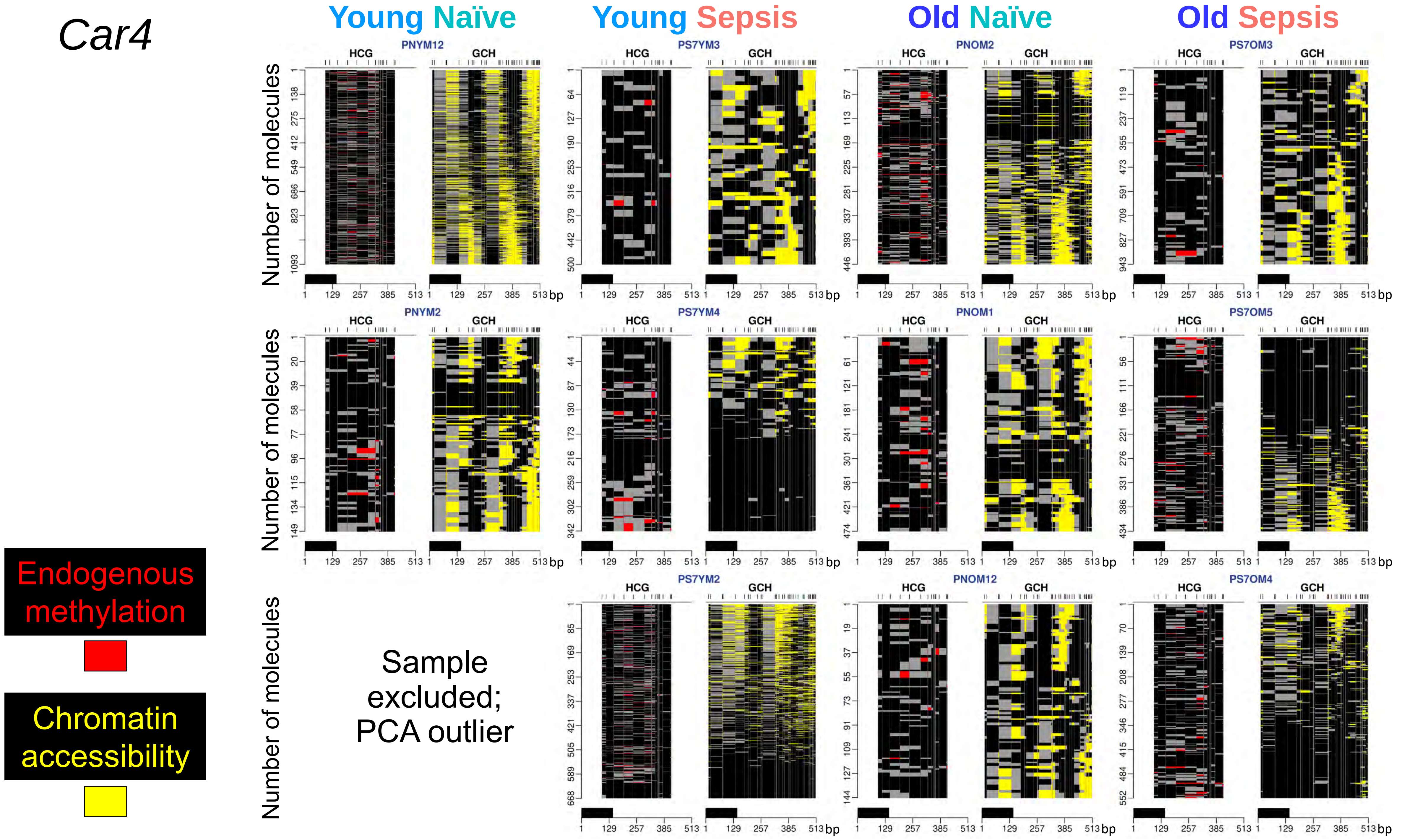

Car4
Young Naïve
Young Sepsis
Old Naïve
Old Sepsis
PNYM12
PS7YM3
PNOM2
PS7OM3
PNYM2
PS7YM4
PNOM1
PS7OM5
PS7YM2
PNOM12
PS7OM4
HCG
GCH
Number of molecules
1 129 257 385 513 bp
Sample excluded; PCA outlier
Endogenous methylation
Chromatin accessibility

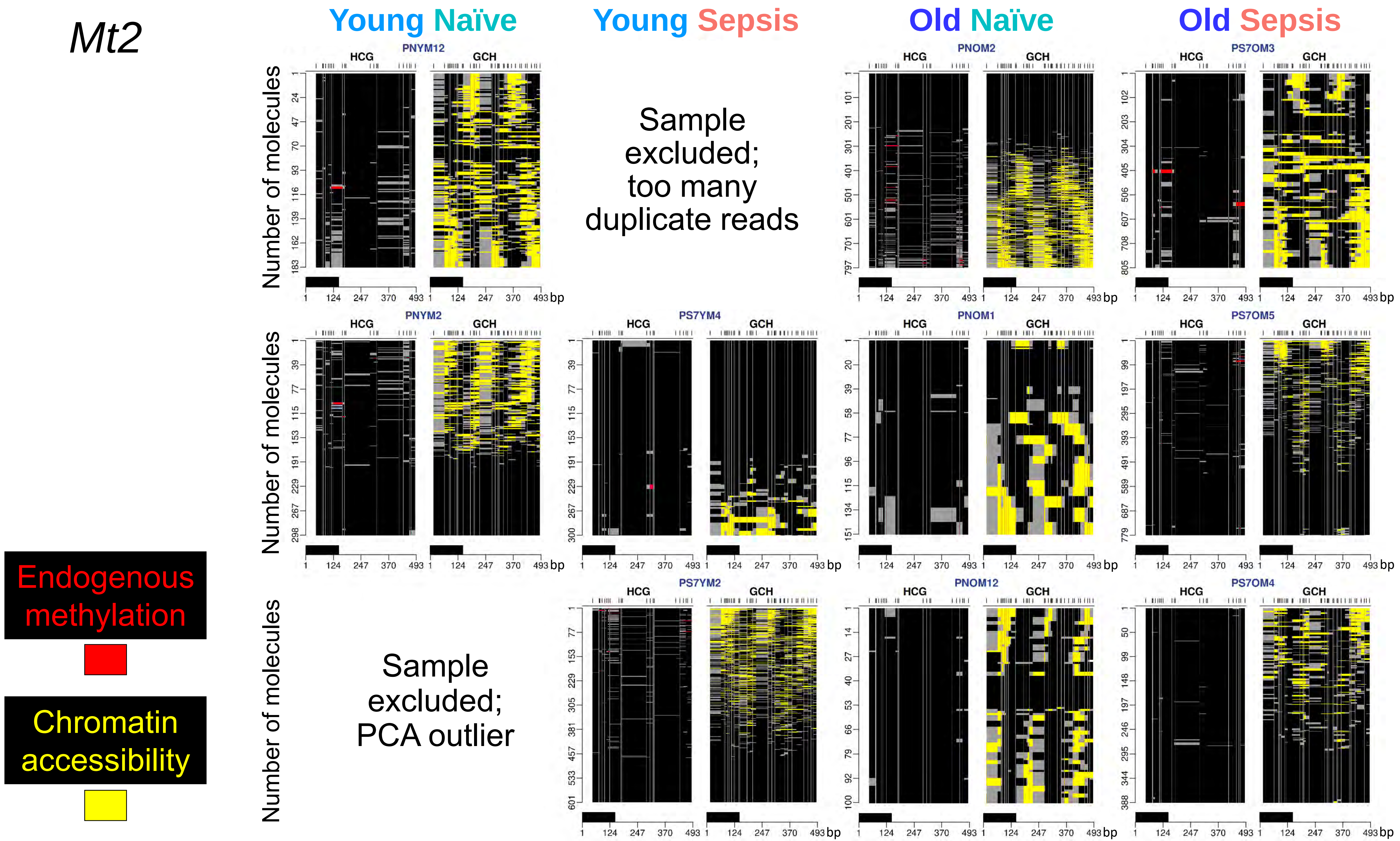
Mt2
Young Naïve
Young Sepsis
Old Naïve
Old Sepsis
PNYM12
PNOM2
PS7OM3
PNYM2
PS7YM4
PNOM1
PS7OM5
PS7YM2
PNOM12
PS7OM4
HCG
GCH
Number of molecules
1
124
247
370
493
bp
Sample excluded; too many duplicate reads
Sample excluded; PCA outlier
Endogenous methylation
Chromatin accessibility

Igkv4-69

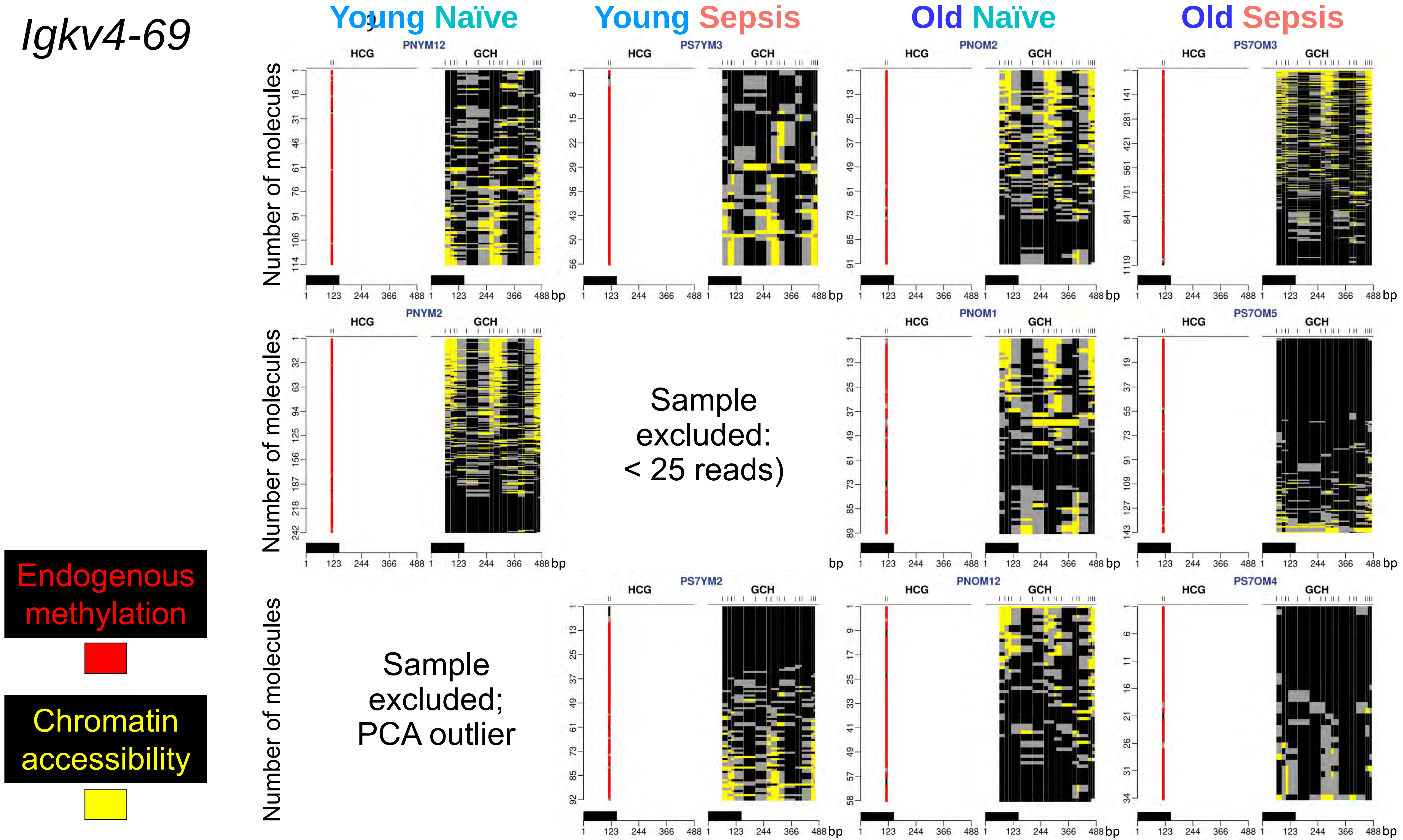

Young Naïve
Young Sepsis
Old Naïve
Old Sepsis
PNYM12
PS7YM3
PNOM2
PS7OM3
PNYM2
PNOM1
PS7OM5
PS7YM2
PNOM12
PS7OM4
HCG
GCH
Number of molecules
bp
Sample excluded: < 25 reads)
Sample excluded; PCA outlier
Endogenous methylation
Chromatin accessibility

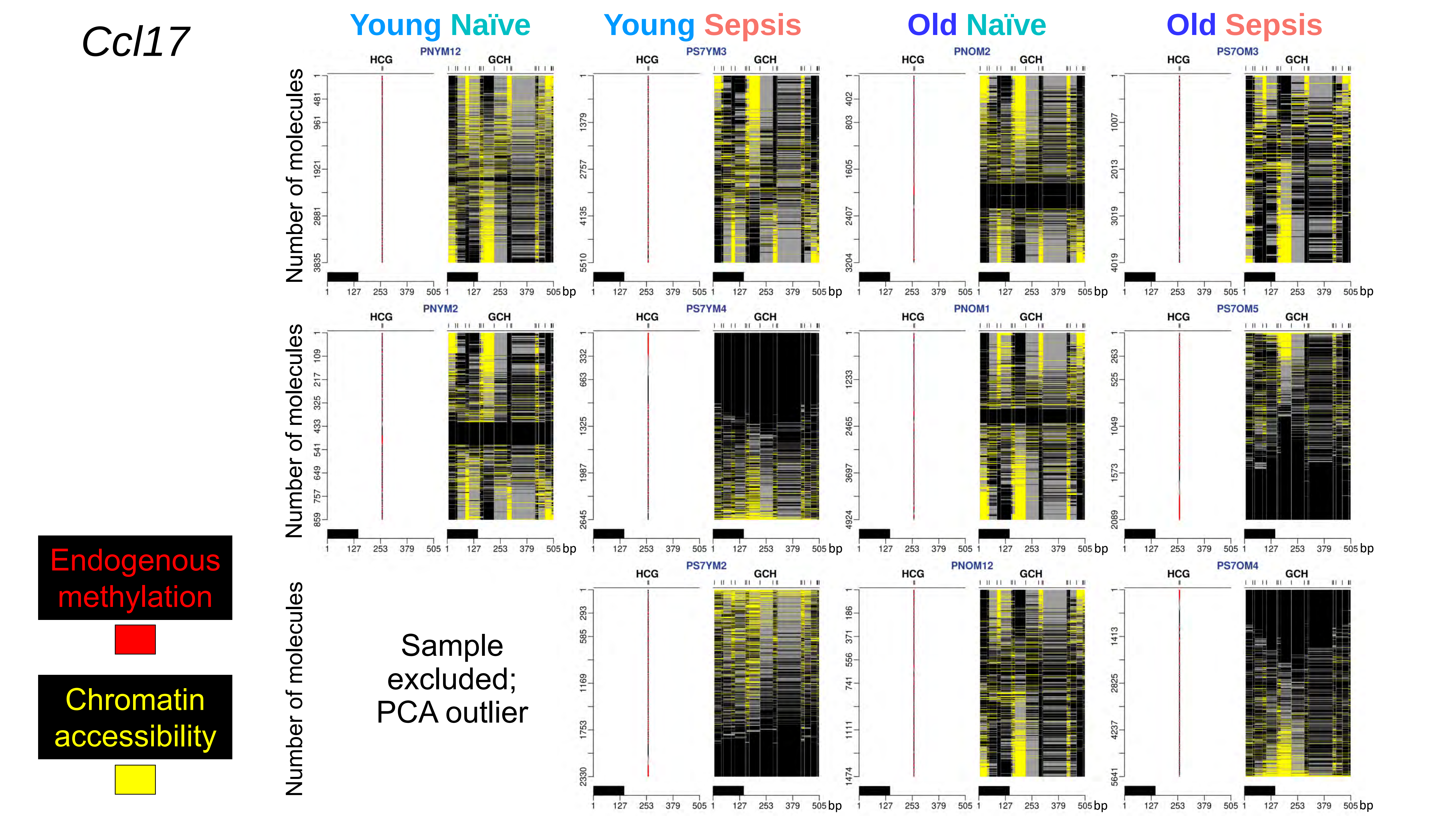
Ccl17
Young Naïve
Young Sepsis
Old Naïve
Old Sepsis
PNYM12
PS7YM3
PNOM2
PS7OM3
PNYM2
PS7YM4
PNOM1
PS7OM5
PS7YM2
PNOM12
PS7OM4
HCG
GCH
Number of molecules
1
127
253
379
505
bp
Sample excluded; PCA outlier
Endogenous methylation
Chromatin accessibility

Igkv12-44

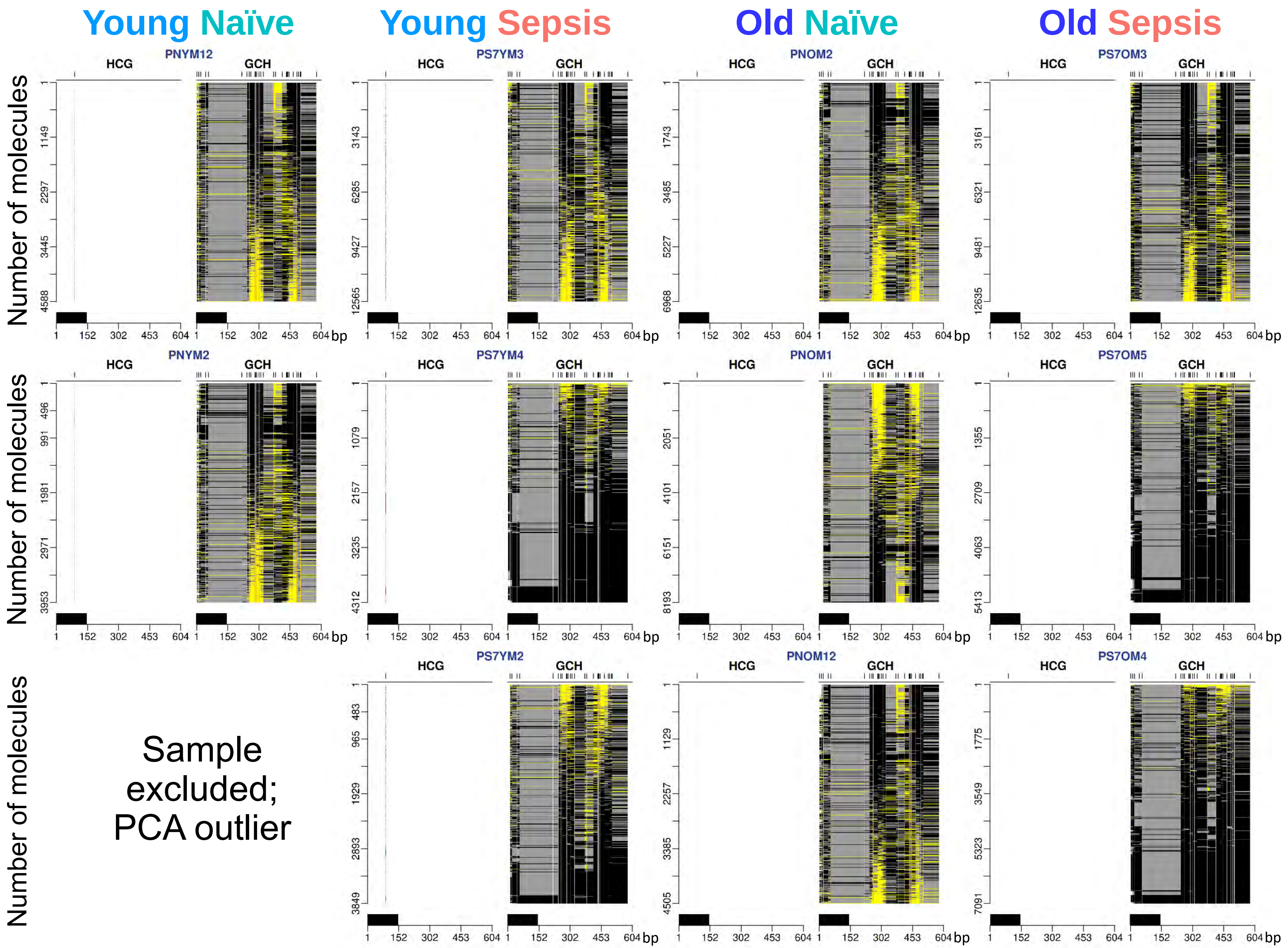

Endogenous methylation

Chromatin accessibility

Class 8 promoters: *Cd274*, *Nfkbiz*, *Hdac3*, *Vdr*, *Il4ra*, *Atf6*, *Il1rl2*

Constitutive NFRs that largely remain refractory to sepsis:

- Decrease in accessibility in old sepsis compared to all other cohorts
- Low, baseline levels of CpG methylation (any observed HCG methylation is likely attributable to M.CviPI methylating CCG sites in highly accessible regions)

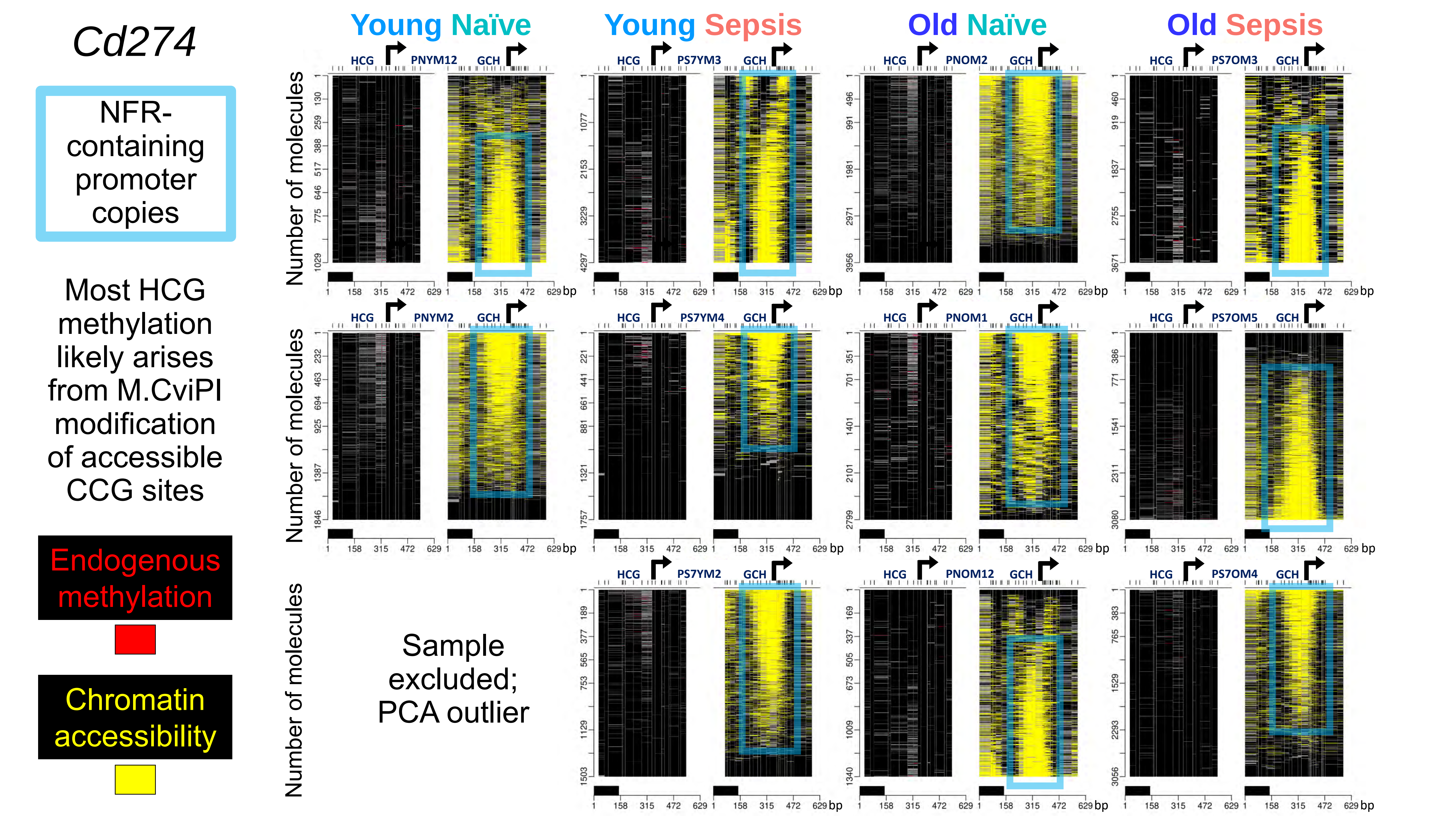
Cd274
NFR-containing promoter copies
Most HCG methylation likely arises from M.CviPI modification of accessible CCG sites
Endogenous methylation
Chromatin accessibility
Young Naïve
Young Sepsis
Old Naïve
Old Sepsis
HCG
GCH
PNYM12
PNYM2
PS7YM3
PS7YM4
PS7YM2
PNOM2
PNOM1
PNOM12
PS7OM3
PS7OM5
PS7OM4
Number of molecules
Sample excluded; PCA outlier
bp

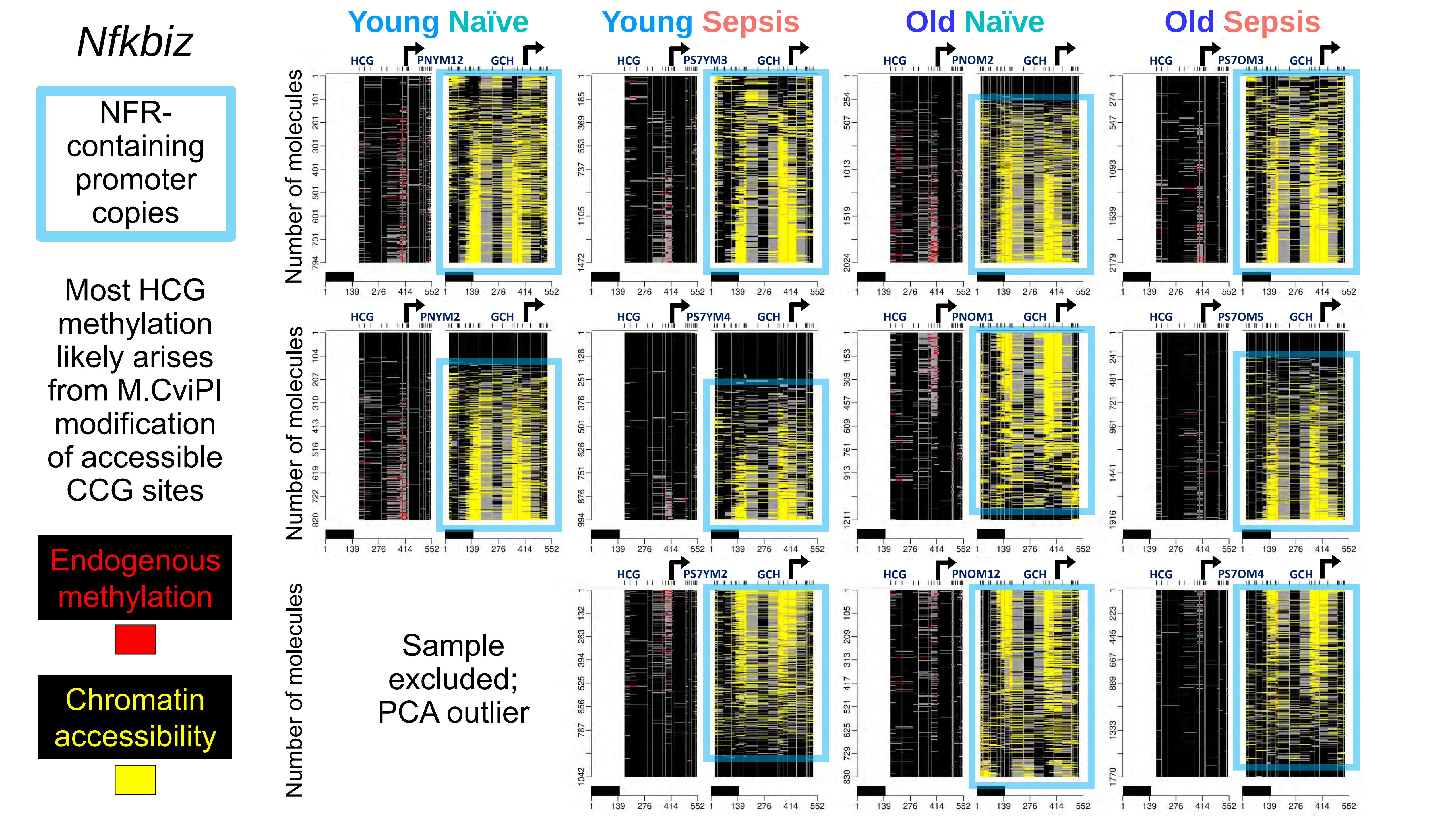

Nfkbiz
NFR-containing promoter copies
Most HCG methylation likely arises from M.CviPI modification of accessible CCG sites
Endogenous methylation
Chromatin accessibility
Young Naïve
Young Sepsis
Old Naïve
Old Sepsis
Number of molecules
HCG
GCH
PNYM12
PS7YM3
PNOM2
PS7OM3
PNYM2
PS7YM4
PNOM1
PS7OM5
PS7YM2
PNOM12
PS7OM4
Sample excluded; PCA outlier
1 139 276 414 552

Hdac3

NFR-containing promoter copies

Most HCG methylation likely arises from M.CviPI modification of accessible CCG sites

Endogenous methylation

Chromatin accessibility

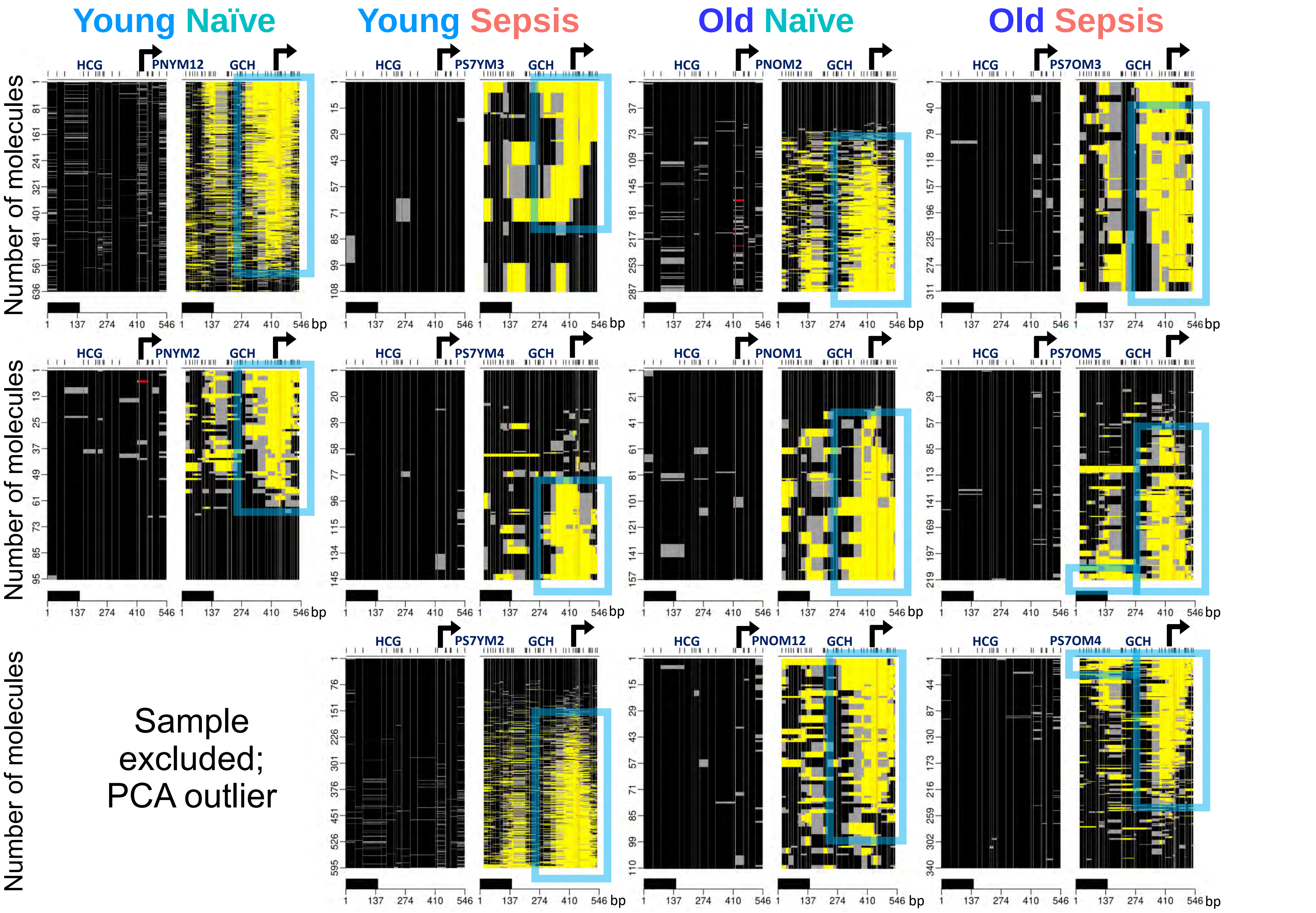

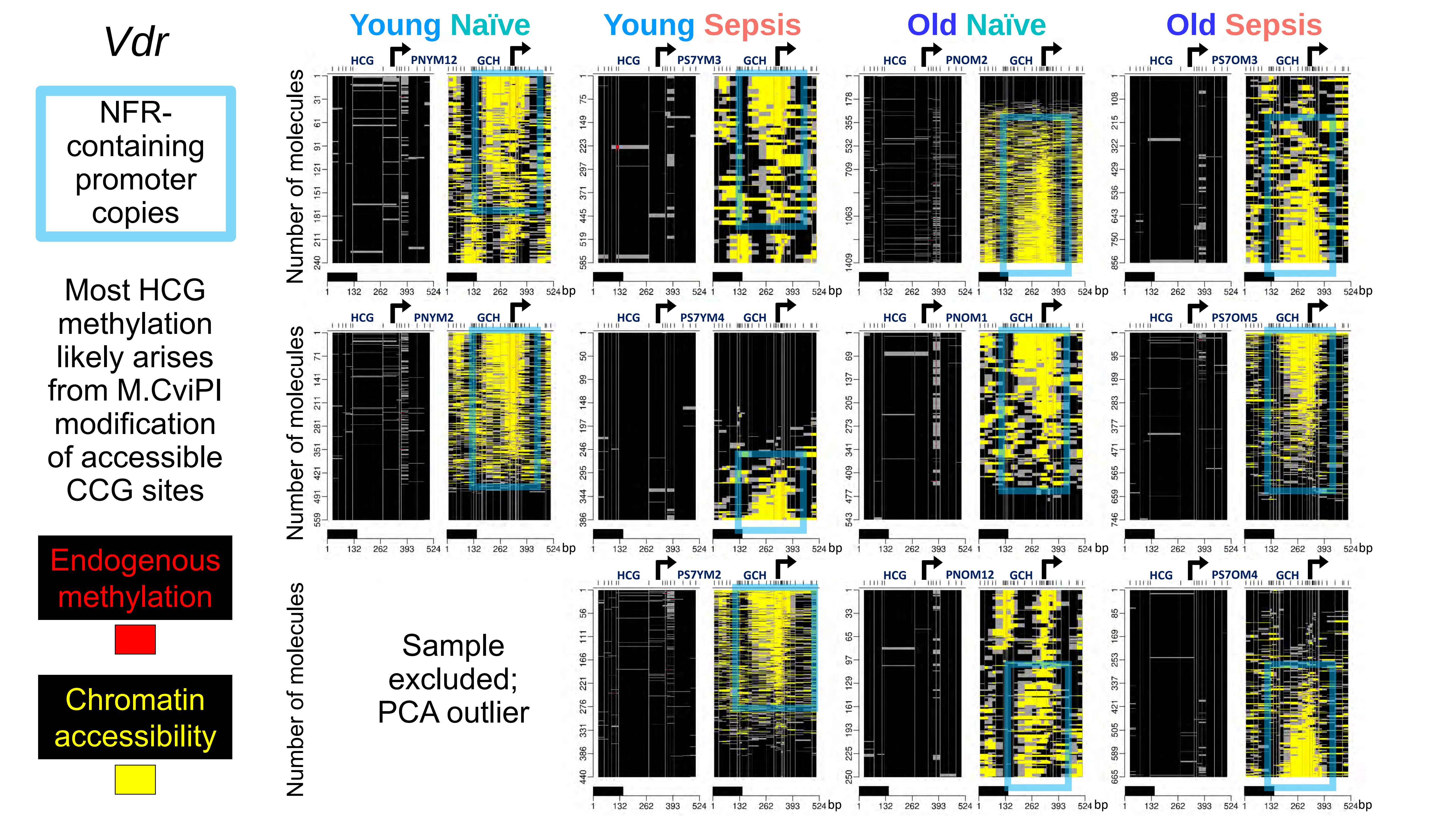

Vdr
NFR-containing promoter copies
Most HCG methylation likely arises from M.CviPI modification of accessible CCG sites
Endogenous methylation
Chromatin accessibility
Young Naïve
Young Sepsis
Old Naïve
Old Sepsis
PNYM12
PNYM2
PS7YM3
PS7YM4
PS7YM2
PNOM2
PNOM1
PNOM12
PS7OM3
PS7OM5
PS7OM4
HCG
GCH
Number of molecules
bp
Sample excluded; PCA outlier

Il4ra

NFR-containing promoter copies

Most HCG methylation likely arises from M.CviPI modification of accessible CCG sites

Endogenous methylation

Chromatin accessibility

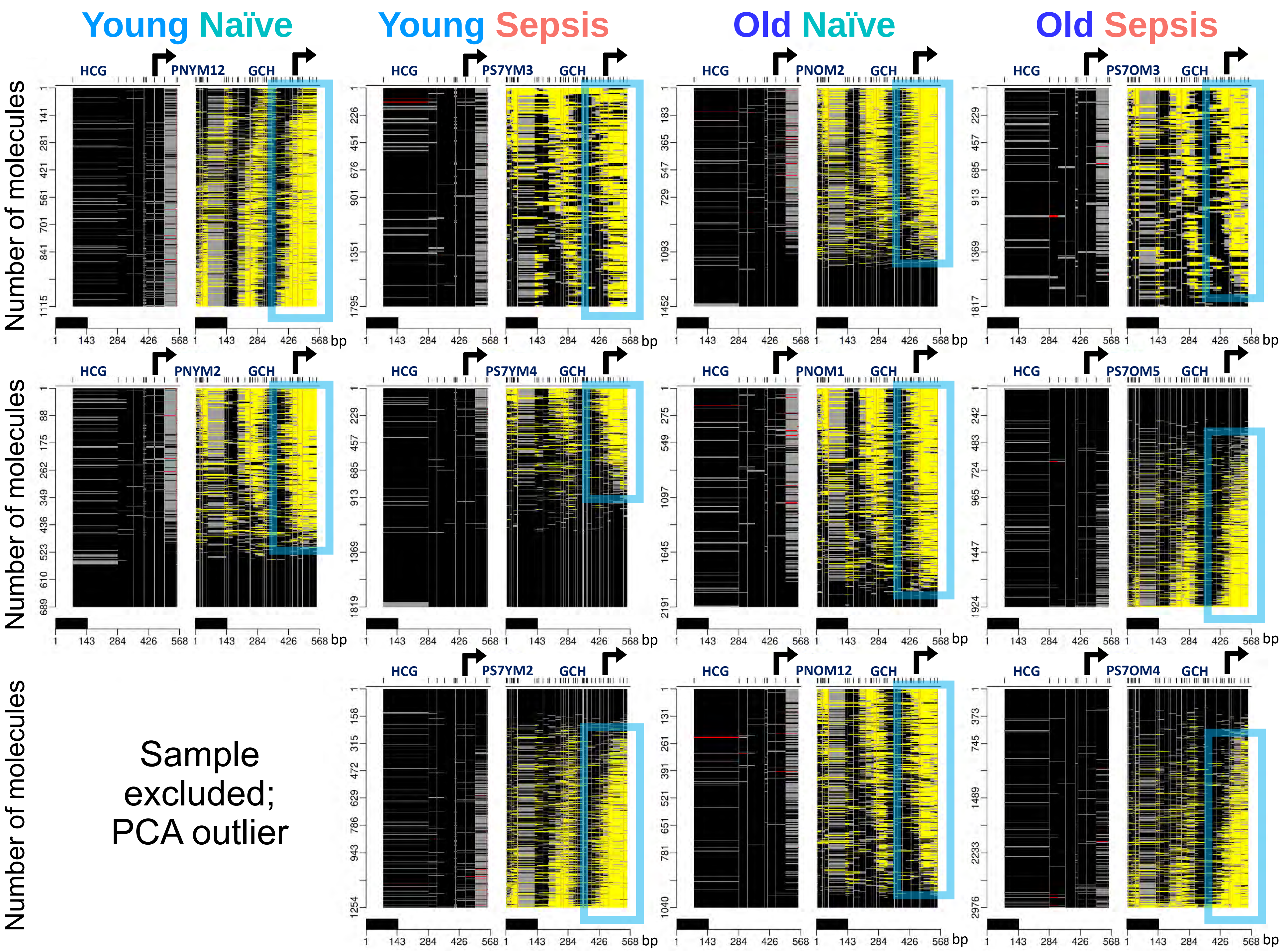

Atf6

NFR-containing promoter copies

Most HCG methylation likely arises from M.CviPI modification of accessible CCG sites

Endogenous methylation

Chromatin accessibility

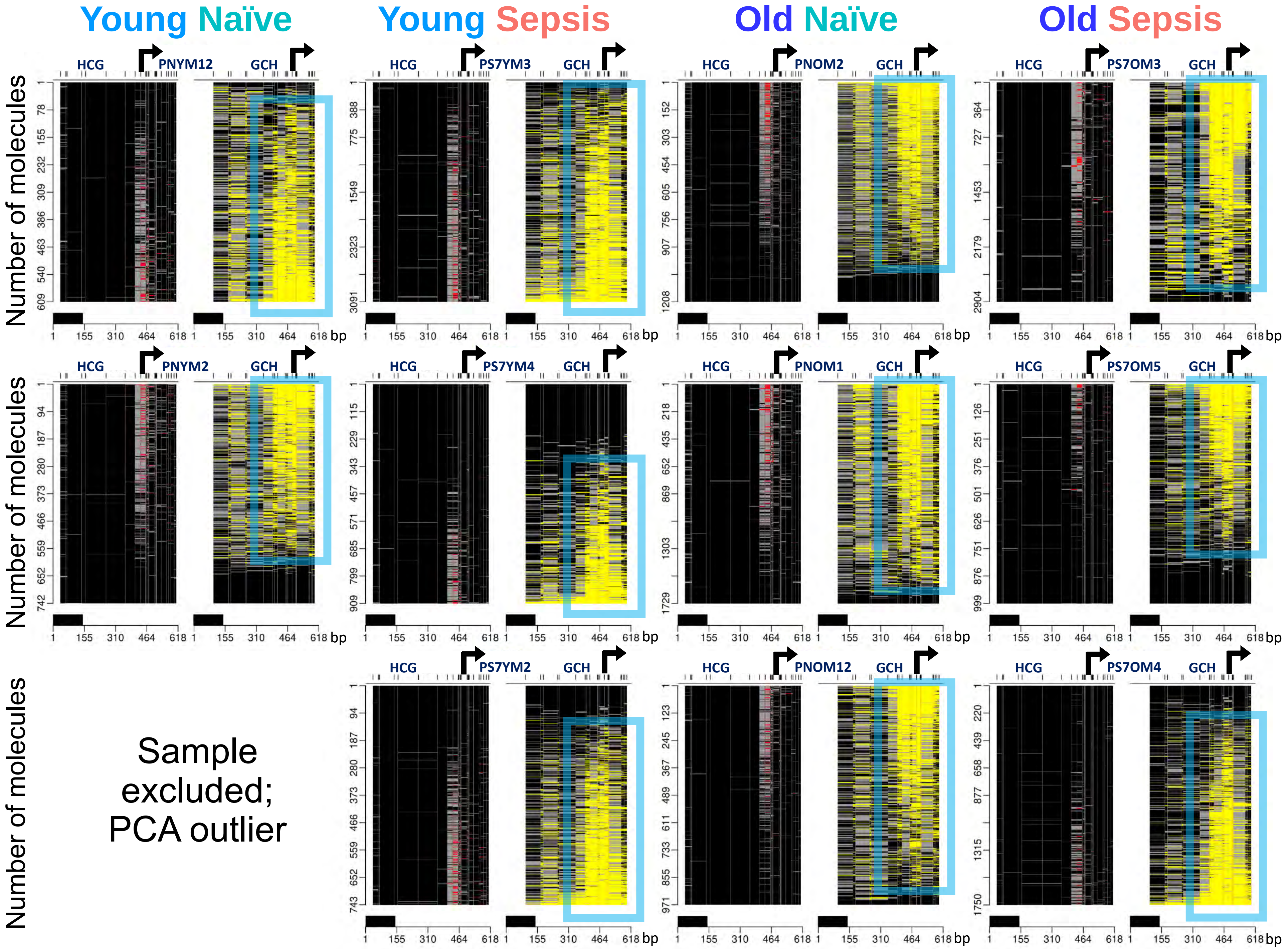

Il1rl2

NFR-containing promoter copies

Most HCG methylation likely arises from M.CviPI modification of accessible CCG sites

Endogenous methylation

Chromatin accessibility

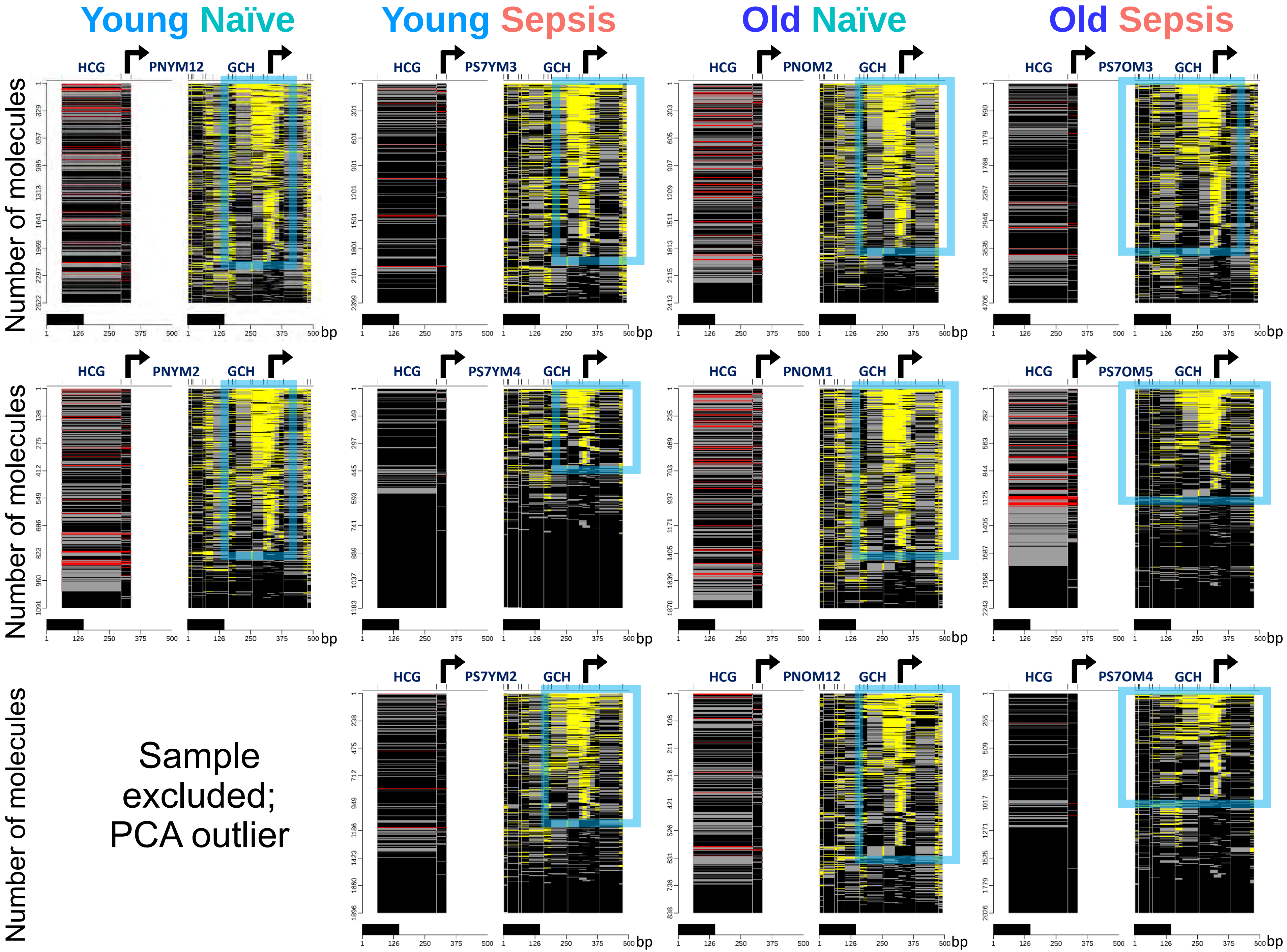

Class 9 promoter: *Tet2*

Constitutive NFR that is maintained under all conditions:

- Low, baseline levels of CpG methylation (any HCG methylation observed above background is due to CCG methylation by M.CviPI in highly accessible regions)

Tet2

NFR-containing promoter copies

Note: All HCG methylation is likely due to modification of accessible CCG sites by M.CviPI

Endogenous methylation

Chromatin accessibility

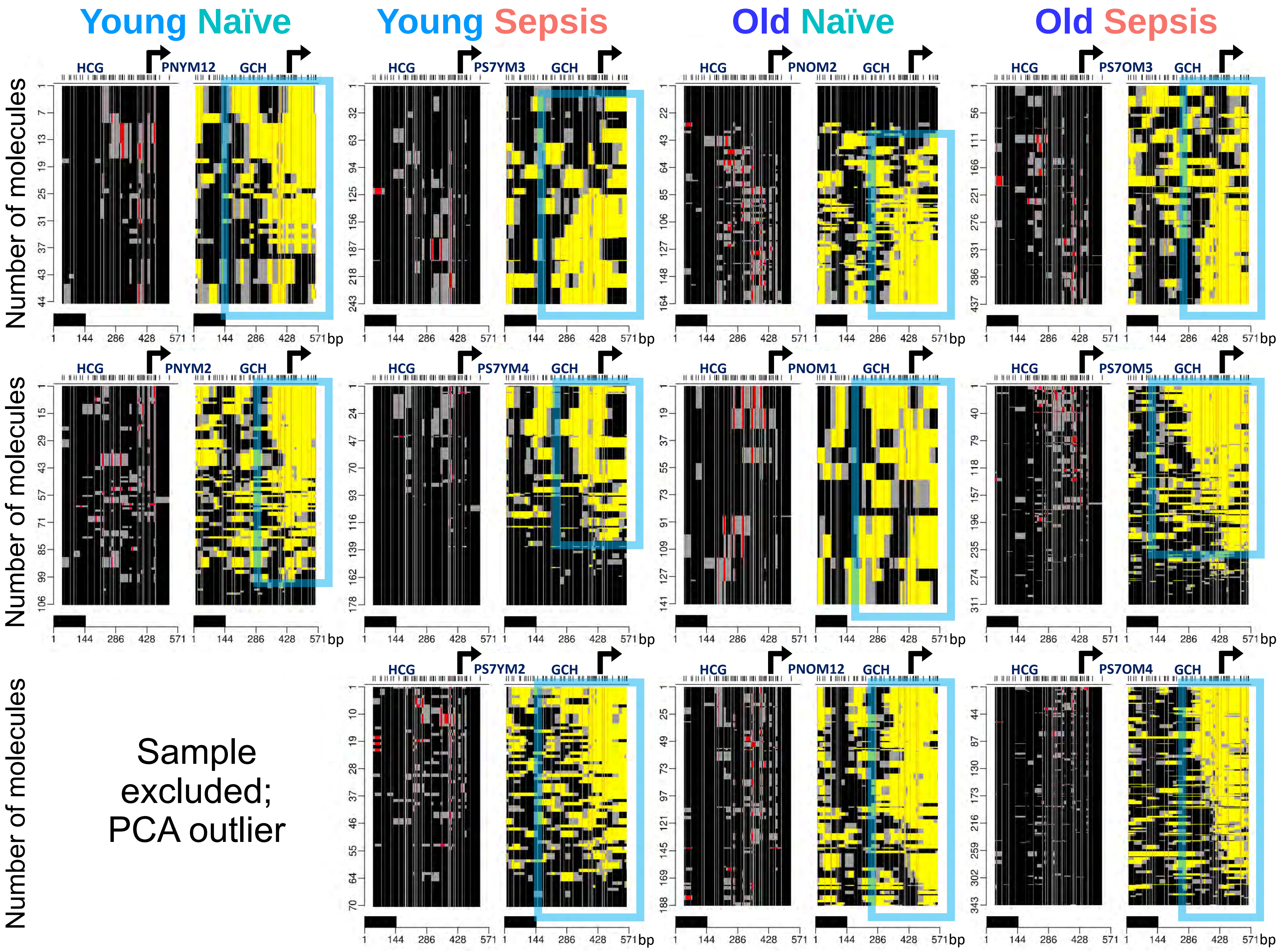

Supplement: Supplementary file 3 [file DataSheet3.pdf]
